# Supplementary material for: Biomimetic α-selective ribosylation enables two-step modular synthesis of biologically important ADP-ribosylated peptides
Source: Nat Commun. 2020 Nov 5;11:5600. doi: 10.1038/s41467-020-19409-1 (PMC7645758; doi:10.1038/s41467-020-19409-1)
Supplement: Supplementary file 1 — Supplementary Information [file 41467_2020_19409_MOESM1_ESM.pdf]

Supplementary Information for

Biomimetic α-Selective Ribosyaltion Enables Two-step Modular Syntheses of Biologically Important ADP-ribosylated Peptides

Content

Supplementary Methods

General information and materials……………………………………….……………………2-3

Abbreviations…………………………………………………………………….……………3

Investigations of the effects of water and temperature on transformations of β-NAD^+ to α/β-ADPr-N_3 with IL[TEOA][Glycolate]……………………………….………….……..……...4

Preparative methods of the ionic liquids……………………………………….……...…4-6

Preparative methods of ADP-ribosyl azide compounds……………...……………..…...…6-7

Investigation of ligands in click reactions of α-ADPr-N_3……………………………...……7-8

Plasmid construction……………………………..…………….……...……………....….…....8

Protein expression and purification……………………….……...…….……………........8-9

Cell culture and preparation of cell powder…………………….……...…….............................9

Preparation of cell lysate……………………………….………………………......…....9-10

Immunoblotting…………………………………………………………….………...…10

Estimation of stability of ADP-ribosylated peptide………….………... ……………………..10

Supplementary Tables

Supplementary Table 1…………………………...………………………………………......11

Supplementary Table 2…………………………...…………………………………………12-14

Supplementary Table 3…………………………...…………………………………………15-16

Supplementary Table 4…………………………...………………………………………..17

Supplementary Table 5-6………………………...……………………………………..…..18

Supplementary Table 7…………………………...………………………………………..19

Supplementary Table 8…………………………...………………………………………..20

Supplementary Table 9…………………………...………………………………………..21

Supplementary Figures

Supplementary Figure 1…….……………………...………………………………………22

Supplementary Figure 2-7…………………………...…………………………………23-24

Supplementary Figure 8…………………………...……………………………...……25-26

Supplementary Figure 9…………………………....………………………………………26

Supplementary Figure 10…………………………...………………………….............27

Supplementary Figure 11…………………………………...…………………………......28

Supplementary Figure 12…………………………...…………………………..................29

Supplementary Figure 13-16…………………....……………….…………………...…...47-48

Compound characterization data and spectra

Characterization of synthesized ionic liquids…….………………………………………30-46

Characterization of synthesized ADP-ribosyl azide compounds……………….…………...46-50

Characterization of synthesized ADP-ribosylated peptide compounds……………………..51-53

^{1}H NMR and ^{31}P NMR spectra of ADP-ribosyl azide compounds………………….......…54-60

^{1}H NMR and ^{13}C NMR spectra of synthesized ionic liquids ……………...………….…...61-111

Supplementary references…………………………....………..……..……...……...111-112

Supplementary Methods

General Information and Materials:

NMR spectra were recorded on 400 or 600 MHz using TMS as internal standard. Chemical shifts were reported in parts per million and coupling constants quoted in Hz. The LC-MS were calibrated by authentic standards. Matrix Assisted Laser Desorption/Ionization time-of-flight mass spectrometry with 2,5- Dihydroxybenzoic acid (DHB) as the matrix. β-NAD^+ were bought from Alfa Aesar. Thermal analysis of ionic liquids was performed on a Netzsch STA 449 F5 using the Proteus Analysis software. Elemental analyses were performed on an Elementar vario EL III elemental analyser. The microanalysis of water contents of ionic liquids was performed by using Karl Fischer Titration method. 1,8-Diazabicyclo[5.4.0]undec-7-ene (Aldrich, 99%); Ambersep®900(OH), ion exchange resin (Sigma-Aldrich); Choline Chloride (Sigma, 99%); N,N-Dimethylethanolamine (Acros, 99%); 1,1,3,3-Tetramethylguanidine (Macklin, 99%); 1-Methylimidazole (Sigma, 99%); Triethanolamine (Macklin, 98%); Diethanolamine (Alfa, 98%); N-Methylmonoethanolamine (aladdin, 99%); Hexanoic acid (Sigma-Aldrich, 99%); Valeric acid (Sigma-Aldrich, 99%); Crotonic acid (Macklin, 98%); Lactic acid (Alfa, 98%); Phosphoric acid (Aladdin, 99%); Propionic Acid (Alfa, 99%); Dichloroacetic acid (Alfa, 99+%); Glycolic acid (Aladdin, 98%); Benzoic acid (Aladdin, 99.5%); Trifluoroacetic Acid (Macklin, 99.5%); Acetate (Aladdin, 99.5%); Butyric acid (Alfa,99+%); 3,5,5-Trimethylhexanoic Acid (Aladdin, 97%); Isovaleric acid (Macklin, 99%). The peptides used for click reactions and control assays were ordered from GL Biochem (Shanghai) Ltd. The ionic liquid pool for screening was built based on the methods reported in our previous work.[1] HPLC analysis was conducted by Thermo Ultimate 3000, with Thermo Hypersil GOLD C18 (250x4.6mm, 5μm). Purifications of ADP-ribosyl molecules and ADP-ribosylated peptides were conducted by semi-preparative HPLC with C-18 column.

Human PARP1 enzyme and biotinylated β-NAD^+ were purchased from Trevigen. Activated DNA was purchased from BPS Bioscience. HeLa S3 cells were purchased from American Type Culture Collection (ATCC). Antibodies used in this study were macroH2A1.1 antibody (1:1000 dilution, D5F6N, Cell Signaling Technology, #12455),

PARP9 antibody (1:1000 dilution, ab53796, Abcam), and anti-poly(ADP-ribose) polymer antibody (1:1000 dilution, ab14459, Abcam). Goat anti-rabbit-HRP conjugated secondary antibody were purchased from Santa Cruz (1:10000 dilution, sc-2004). Donkey anti-mouse-HRP conjugated secondary antibody were purchased from Santa Cruz (1:10000 dilution, sc-2314). Streptavidin-HRP was purchased from Abcam (1:100000 dilution, ab7403). Photo-cross-linking was performed with ENF-260C/FE hand-hang UV lamp (Spectroline). Immunoblotting membranes were visualized by MyECL Imager from Thermo Scientific. All images were processed by Image*J* software (National Institutes of Health), and contrast was adjusted appropriately. IC_{50} and EC_{50} were fit with Origin 7.0 software package (OriginLab).

Abbreviations:

[DBU] = 1,8-Diazabicyclo[5.4.0]undec-7-ene;

[TMG] = N,N'-Tetramethylguanidine;

[TFA] = Trifluoroethanoicacid;

[DCA] = Dichloroacetic acid;

[MEOA] = 2-Methylaminoethanol;

[DEOA]= Diethanolamine;

[DMEOA] = N,N-Dimethylethanolamine;

[TEOA] = Triethanolamine;

[BMIM] = 1-Butyl-3-methylimidazolium;

[HMIM] = 1-Hexyl-3-methylimidazolium;

[OMIM] = 1- Octyl-3-methylimidazolium;

[DodecylMIM] = 1-dodecyl-3-methylimidazolium;

[TetradecylMIM] = 1-tetradecyl-3-methylimidazolium.

Investigations of the effects of water and temperature on transformations of β-NAD+ to α/β-ADPr-N3 with IL[TEOA][Glycolate]

The reaction of β-NAD+ (0.5 mg, 0.75μmol), NaN_3 (2 mg, 0.03 mmol) in the mixture of H_2O and ionic liquid (IL = [TEOA][Glycolate]) were conducted in a PCR instrument under different reaction conditions. The resulted reaction mixtures were analyzed by HPLC. It was found that the selectivity of α/β-ADPr-N_3 greatly depended on the water contents, which ranged from 1.5 to 8.2 under the given reaction temperatures and times. The results of the experiments were listed in Table S1.

Preparative methods of the ionic liquids

All of the ionic liquids prepared in our work have been prepared and purified according to the traditional methods which have been reported in literature and well established in our own lab from 2005. [1-5]

Method-(1) (Supplementary Figure 1A) is used for the preparations of protonic ionic liquids including DBU-based ionic liquids, TEOA-based ionic liquids, DEOA-based ionic liquids, DMEOA-based ionic liquids and TMG-based ionic liquids. In the typical procedure for Method-(1), the two donors of cations and anions were strictly weighed according to the molar ratio of 1:1, and then the anhydrous methanol solution of the anion donor reagent was slowly added dropwise into the anhydrous methanol solution of the cationic donor reagent under ice bath conditions. After the reaction mixture was stirred for 1 day at room temperature, the solvent was removed under reduced pressure to give the ionic liquids containing the given anions and cations. The ionic liquids were further dried at 60°C for 24 hours under reduced pressure. The ionic liquids were characterized by ^1H NMR, ^{13}C NMR, IR and DSC/TGA. Both of solvents residues and unreacted materials were detected by ^1H NMR and ^{13}C NMR spectra, which showed that there are no detectable these kinds of impurities. The water contents of the samples were detected by using Karl Fischer Titration method, indicating less 0.5% (w/w) (Table S9 in the supporting information). Because of no any inorganic salts involved during the preparative procedures of this kind of ionic liquids, no further detections including halide analysis were conducted.

Method-(2) (Supplementary Figure 1B) is used for the preparations of choline-based ionic liquids. In the typical procedure for Method-(2), choline hydroxide was prepared form choline chloride as the starting material through a strong basic anion exchange resin. The aqueous solvent of choline hydroxide could be obtained and then reacted with 1.0 equivalent of carboxylic acid at room temperature for 1 day. After the solvents were removed under reduced press, the choline-based ionic liquids were obtained. The ionic liquids were further dried at 60°C for 24 hours under reduced pressure. The ionic liquids were characterized by ^{1}H NMR, ^{13}C NMR, IR and DSC/TGA. The solvents and unreacted starting materials residues were detected using ^{1}H NMR and ^{13}C NMR, which showed that there are no detectable these kinds of impurities. The possible halides residues coming from the preparation procedures were detected using chloride-selective electrode, the results showed that the chloride content all smaller than 0.05 mol/kg. The water contents of the samples were detected by using Karl Fischer Titration method, indicating less than 0.5% (w/w) (Table S9 in the supporting information).

Method-(3) (Supplementary Figure 1C) is used for the preparations of brominated ionic liquids ([C4-Choline][Br], [C8-Choline][Br] and [C12-Choline][Br]) and imidazole-type ionic liquids. In the typical procedure for Method-(3), 1.1 equivalent of bromo/chloroalkane was slowly added dropwise to 1.0 equivalent of 1-methylimidazole/2-dimethylaminoethanol in ethyl acetate in an ice bath, and refluxed at 75 °C. The aqueous phase was extracted after the completion of the reaction, and ionic liquids could be obtained when the aqueous solvent was removed. The ionic liquids bearing BF_4 as the anions were prepared from the corresponding imidazolium ionic liquids. Briefly, through a strong basic anion exchange resin, the aqueous solvent of hydroxide/imidazolium type ILs could be obtained and then reacted with 1.0 equivalent of tetrafluoroboric acid at room temperature for 1 day. After the solvents were removed, the target ionic liquids were obtained. The ionic liquids were further dried at 60°C for 24 hours under reduced pressure. The brominated ionic liquids ([C12-Choline][Br], [C8-Choline][Br] and [C4-Choline][Br]) that haven't reported before are characterized by ^{1}H NMR, ^{13}C NMR, IR and DSC/TGA. Their purity was detected by ^{1}H NMR and elemental analysis (%CHN), and water contents were detected by using

Karl Fischer Titration method, indicating less than 0.5% (w/w) (Table S9 in the supporting information). The reported imidazole-type ionic liquids are identified by ^1H NMR and compared with the data in literatures. [6-10]

Synthesis of ADP-ribosyl azide compounds

Synthesis of α-ADPr-N_3 (1)

The mixture of 50 mg of β-NAD^+ (0.075 mmol) and 196 mg of sodium azide (3 mmol) in the mixed solvents (0.5 mL) of H_2O and [DEOA][Lactate] (mol/mol = 5:1) was heated at 90 ˚C for 2 h, and then purified by preparative HPLC. The column was eluted using a linear gradient of 0–10% CH_3CN in TEAB buffer (0.1 M, pH 7.5) within 35 min to give α-ADPr-N_3 at 14.7 min (39 mg, 88%); to give β-ADPr-N_3 at 15.9 min. Ratio of α/β-ADPr-N_3 was calculated by the peak area of α/β-ADPr-N_3.

Scale-up synthesis of α-ADPr-N_3 (**1**) was conducted with 0.5 g of β-NAD^+ and 5.0 g sodium azide as the starting materials. The mixture in 5 mL mixed solvent of H_2O and [DEOA][Lactate] (mol/mol = 5:1) were stirred at 90ºC for 2 hours, and then purified by preparative HPLC a linear gradient of 0–10% CH_3CN in TEAB buffer (0.1 M, pH 7.5). The isolated yield was calculated after lyophilization.

Synthesis of α-ADPPr-N_3 (2)

The mixture of 20 mg of NADP (0.03 mmol) and 64 mg of sodium azide (1.0 mmol) in the mixed solvents (0.5 mL) of H_2O and [DEOA][Lactate] (mol/mol = 5:1) was heated at 90 ˚C for 4 h, and then purified by preparative HPLC. The column was eluted using a linear gradient of 0–10% CH_3CN in TEAB buffer (0.1 M, pH 7.5) within 35 min to give α-ADPPr-N_3 at 15.9 min (14 mg, 81%); to give β-NADP-N_3 at 17.2 min. Ratio of α/β-ADPPr-N_3 was calculated by the peak area of α/β-ADPPr-N_3.

Synthesis of α-8-Br-ADPr-N_3 (3)

The mixture of 100 mg of β-NAD^+ (0.15 mmol) and 8.8 g of bromine (2.8 mL) in 2 mL of a pH 4.5 sodium acetate buffer solution was stirred at 0 ˚C for 1 h, extracted with

chloroform to the water phase colorless, and then purified by preparative HPLC. The column was eluted using a linear gradient of 0–10% CH_3CN in TEAB buffer (0.1 M, pH 7.5) within 30 min to give 8-Br-β-NAD^+ at 13.8 min (87 mg, 78%).

The mixture of 20 mg of 8-Br-β-NAD^+ (0.03 mmol) and 70 mg of sodium azide (1.1 mmol) in the mixed solvents (0.5 mL) of H_2O and [DEOA][Lactate] (mol/mol = 5:1) was heated at 90 °C for 4 h, and then purified by preparative HPLC. The column was eluted using a linear gradient of 0–40% CH_3CN in TEAB buffer (0.1 M, pH 7.5) within 35 min to give α-8-Br-ADPr-N_3 at 15.9 min (13 mg, 76%); to give β-8-Br-ADPr-N_3 at 16.7 min. Ratio of α/β-8-Br-ADPr-N_3 was calculated by the peak area of α/β-ADPPr-N_3.

Synthesis of α-ADPr-N_3 from cyclic ADP-ribose.

The mixture of 1 mg of cyclic ADP-ribose (0.002 mmol) and 4.9 mg of sodium azide (0.074 mmol) in the mixed solvents (0.06 mL) of H_2O and [DEOA][Lactate] (mol/mol = 5:1) was stirred at 70 °C for 0.5 h, and then analyzed by analytical HPLC. The column was eluted using a linear gradient of 0–10% CH_3CN in TEAB buffer (0.1 M, pH 7.5) within 35 min to give α-ADPr-N_3 at 14.7 min (86%, calculated by standard curve of α-ADPr-N_3); to give β-ADPr-N_3 at 15.9 min. Ratio of α/β-ADPr-N_3 was calculated by the peak area of α/β-ADPr-N_3

Investigation of ligands in the click reaction of α-ADPr-N_3

The mixture of 0.5 mg of β-ADPr-N_3 (0.75 μmol) and 0.1 mg of phenylacetylene (0.9 μmol), copper sulfate pentahydrate with different concentrations shown in Table S6 and sodium ascorbate (0.25 mM), different ligands (Table S5) (0.4 mM), in the mixed solvents (1 mL) of water: ethanol: tert-butanol = 2:3:5 (v/v/v) stirred at 37 ° C, and the reaction is detected by analytical HPLC every 0.5 hour until the peak of the raw material disappears or not reduces in the HPLC spectrum. The column was eluted using a linear gradient of 0–60% CH_3CN in TEAB buffer (0.1 M, pH 7.5) within 20 min to give triazole product at 11.1 min.

Ligand BBTA, TBTA and THPTA (their chemical structures were shown in Table S5) that used for the click reaction of ADPr-N_3 were prepared based on the reported methods in literatures [11, 12,13].

Plasmid construction

pET28a-mH2A1.1(163-369) and pET28a-mH2A1.2(163-372) was constructed by standard restriction enzyme digestion and ligation cloning. Briefly, mH2A1.1(163-369) and mH2A1.2(163-372) fragment was amplified from complementary DNA by PCR using primer pair macroH2AFY-F and macroH2AFY-R. Then the DNA fragment was digested with NdeI and XhoI restriction enzymes and ligated with pET28a vector predigested with the same restriction enzymes. Sequences of all the constructs were verified by Sanger sequencing.

pET28a-mH2A1.1(163-369)G224E was generated from pET28a-mH2A1.1(163-369) by PCR based mutagenesis using primer pair mH2AFY-G224E-F and mH2AFY-G224E-R.

Primer name	Sequence
macroH2AFY-F	ATCTACCATATGGGTGAAGTCAGTAAGGCAGCCAGCGCCG
macroH2AFY-R	TTAAACTCGAGCTAGTTGGCGTCCAGCTTGGCCATTTCC
mH2AFY-G224E-F	GACAAACACTGACTTCTACATCGGTGAGGAAGTAGGAAACACGCTGGAGA
mH2AFY-G224E-R	TCTCCAGCGTGTTTCCTACTTCCTCACCGATGTAGAAGTCAGTGTTTGTC

Protein expression and purification

Briefly, mH2A1.1, mH2A1.1 (G224E) and mH2A1.2 were expressed in *E.coli* Rosetta (DE3) as N-terminal His_6-tagged fusions. Protein expression was induced by adding isopropyl-β-D-thiogalactopyranoside (IPTG) to a final concentration of 1 mM when

OD600 reached 0.6, and the cultures were further grown for 16–20 h at 16 °C. The cells were harvested and resuspended in lysis buffer (25 mM Tris pH 7.4, 500 mM NaCl, 1 mM $MgCl_2$, 5 mM β-mercaptoethanol (DTT), 10 % glycerol, 1 mM PMSF, and 1×Roche Complete EDTA-free protease inhibitors). Following sonication and centrifugation, the supernatant was loaded onto a nickel column (GE Healthcare) pre-equilibrated with lysis buffer. The column was washed with 5 column volumes of wash buffer (lysis buffer with 30 mM imidazole), and then the target proteins were eluted with elution buffer (lysis buffer with 500 mM imidazole). The eluents were further purified by a Superdex 75 10/300 gel-filtration column (GE Healthcare) in lysis buffer. After concentration, the target proteins were frozen and stored at −80 °C.

Cell culture and preparation of cell powder

HeLa S3 cells were cultured in DMEM (Life Technologies) supplemented with 10% fetal bovine serum (FBS, Life Technologies), 100 U/mL penicillin, and 100 μg/mL streptomycin. The cells were maintained in a humidified 37 °C incubator with 5% CO_2. Harvested cell pellets were washed with ice-cold phosphate-buffered saline (PBS) and frozen with liquid N_2. The pellets were grinded with a Ball Mill (Retch MM301) into powder and stored at −80 °C until use.

Preparation of cell lysate

The frozen cell powder was first resuspended in a hypotonic buffer (10 mM HEPES, pH 7.5, 2 mM $MgCl_2$, 0.1% tween-20, 20% glycerol, 2 mM PMSF, and 1×Roche Complete EDTA-free protease inhibitors) and incubated for 10 min on ice. The suspension was centrifuged at 16,000×*g* for 15 min at 4 ºC and the supernatant was kept for use later. The pellet was resuspended in a high-salt buffer (50 mM HEPES, pH 7.5, 420 mM NaCl, 2 mM $MgCl_2$, 0.1% tween-20, 20% glycerol, 2 mM PMSF, and 1×Roche Complete EDTA-free protease inhibitors) and incubated for 30 min on ice. The suspension was centrifuged at 16,000×*g* for 15 min at 4 ºC and the supernatant was combined with the soluble fraction in hypotonic buffer and filtered with a 0.45 μm filter to give the whole-cell lysates. Protein concentration was measured by Bradford (Bio-

Rad).

Immunoblotting

The protein samples in 1×LDS sample loading buffer (Invitrogen) with 50 mM DTT were heated at 80 °C for 8 min. After SDS-PAGE, the proteins were transferred onto a PVDF membrane (GE Healthcare). The membrane was blocked by 5% non-fat milk in Tris-buffered saline with 0.1% tween-20 (TBST) for 1 h at room temperature, and was incubated overnight with primary antibody or streptavidin-HRP in buffer (5% bovine serum albumin in TBST) at 4 °C. For samples incubated with primary antibodies, the membranes were washed by TBST for three times, and then incubated with secondary antibody in TBST for 1 h at room temperature. For all the membranes, after a final washing by TBST for three times, the proteins were visualized with SuperSignal™ west dura extended duration substrate using MyECL Imager system (Thermo Fisher Scientific).

Estimation of stability of ADP-ribosylated peptide

Peptide-3 (50 mM) was incubated in the following buffer, respectively: i) phosphate buffer (pH 7.5) with 0.5 M HN_2OH; ii) phosphate buffer (pH 3.0); iii) phosphate buffer (pH 5.0); iii) phosphate buffer (pH 5.0); iv) phosphate buffer (pH 9.0); v) phosphate buffer (pH 11.0) at 37 ºC for indicated time. The mixtures were analyzed by HPLC with a Vydac 218TP C18 column (4.6 mm×250 mm, 5 µm; Grace Davison, Columbia, MD). Mobile phases used were 0.05% TFA in water (buffer A) and 0.05% TFA in ACN (buffer B). The flow rate for LC was 0.2 mL/min. The reaction mixtures were eluted by buffer A for 5 min and then 5–85% buffer B over 10 min. UV detection was at 220 nm.

Supplementary Table 1 Effects of water and temperature on the transformation of β-NAD$^+$ to α/β-ADPr-N_3.

Entry	H_2O:IL (mol/mol)	Time	Temp	α Yield	β Yield	Ratio (α/β)
1	Neat IL	0.5 h	90 ℃	7.4%	5.1%	1.5
2	10:1	0.5 h	90 ℃	32.4%	4.5%	7.2
3	5:1	0.5 h	90 ℃	42.0%	5.1%	8.2
4	1:1	0.5 h	90 ℃	33.0%	10.8%	3.1
5	1:10	0.5 h	90 ℃	23.3%	6.8%	3.4
6	Water	0.5 h	90 ℃	8.5%	4.0%	2.1
7	5:1	4 h	90 ℃	57.3%	7.9%	7.4
8	5:1	2 h	90 ℃	54.0%	7.4%	7.3
9	5:1	1 h	90 ℃	46.6%	6.3%	7.4
10	5:1	0.5 h	90 ℃	42.0%	5.1%	8.2
11	5:1	10min	90 ℃	13.6%	1.7%	8.0
12	5:1	0.5 h	30 ℃	0%	0%	0
13	5:1	0.5 h	70 ℃	15.3%	6.3%	2.4
14	5:1	0.5 h	75 ℃	24.4%	8.5%	2.9
15	5:1	0.5 h	80 ℃	29.5%	9.1%	3.2
16	5:1	0.5 h	85 ℃	38.1%	7.4%	5.1
17	5:1	0.5 h	90 ℃	42.0%	5.1%	8.2
18	5:1	0.5 h	95 ℃	44.3%	5.7%	7.7
19	5:1	0.5 h	100 ℃	53.9%	7.4%	7.3
20	5:1	0.5 h	105 ℃	55.1%	9.1%	6.1

Supplementary Table 2 Chemical structures of ionic liquids.

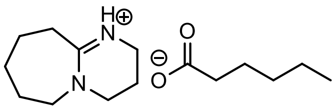

1. [DBU][Hexanoate]

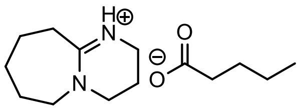

2. [DBU][Valerate]

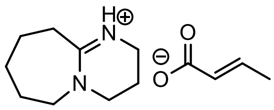

3. [DBU][Crotonate]

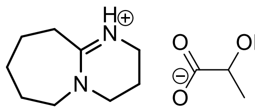

4. [DBU][Lactate]

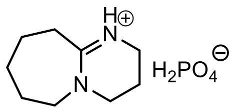

5. [DBU][H_2PO_4]

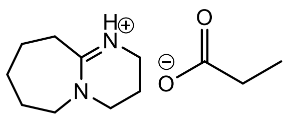

6. [DBU][Propionate]

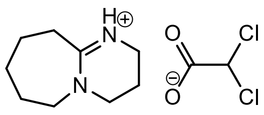

7. [DBU][DCA]

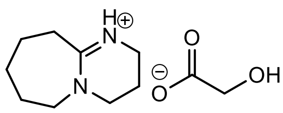

8. [DBU][Glycolate]

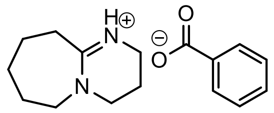

9. [DBU][Benzoate]

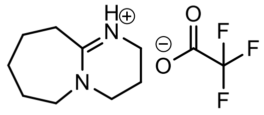

10. [DBU][TFA]

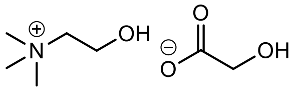

11. [Choline][Glycolate]

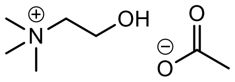

12. [Choline][Acetate]

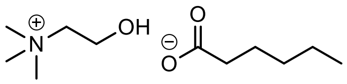

13. [Choline][Hexanoate]

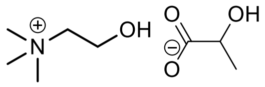

14. [Choline][Lactate]

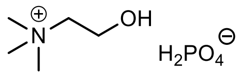

15. [Choline][H_2PO_4]

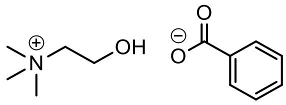

16. [Choline][Benzoate]

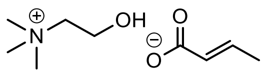

17. [Choline][Crotonate]

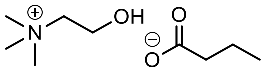

18. [Choline][Butyrate]

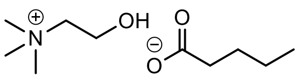

19. [Choline][Valerate]

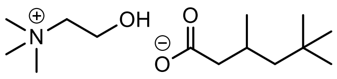

20. [Choline] [3,5,5-Trimethylhexanoate]

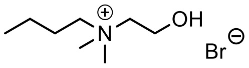

21. [C4-Choline][Br]

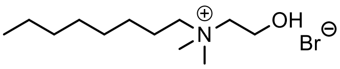

22. [C8-Choline][Br]

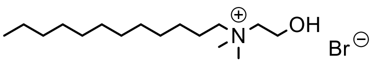

23. [C12-Choline][Br]

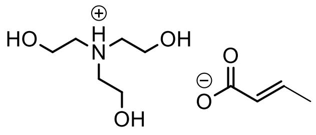

24. [TEOA][Crotonate]

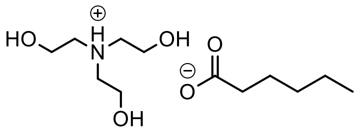

25. [TEOA][Hexanoate]

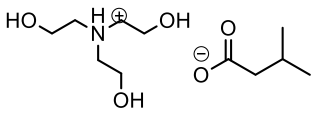

26. [TEOA][Isovalerate]

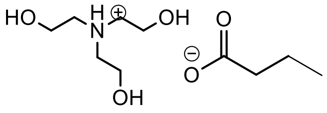

27. [TEOA][Butyrate]

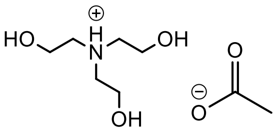

28. [TEOA][Acetate]

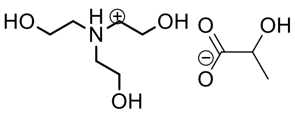

29. [TEOA][Lactate]

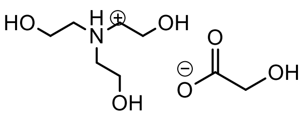

30. [TEOA][Glycolate]

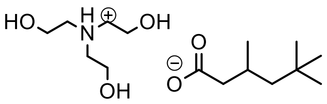

31. [TEOA][3,5,5-Trimethylhexanoate]

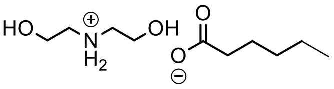

32. [DEOA][Hexanoate]

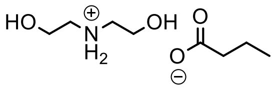

33. [DEOA][Butyrate]

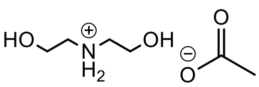

34. [DEOA][Acetate]

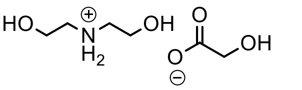

35. [DEOA][Glycolate]

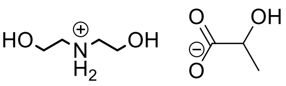

36. [DEOA][Lactate]

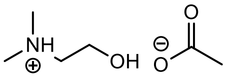

37. [DMEOA][Acetate]

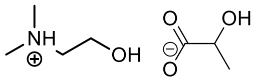

38. [DMEOA][Lactate]

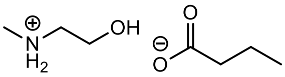

39. [MEOA][Butyrate]

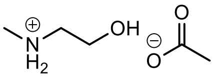

40. [MEOA][Acetate]

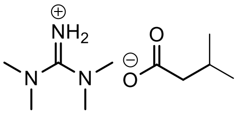

41. [TMG][Isovalerate]

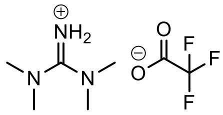

42. [TMG][TFA]

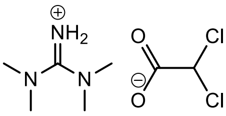

43. [TMG][DCA]

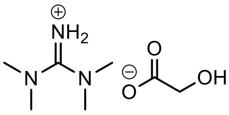

44. [TMG][Glycolate]

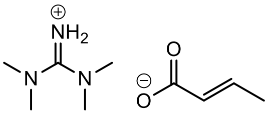

45. [TMG][Crotonate]

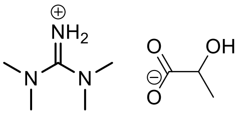

46. [TMG][Lactate]

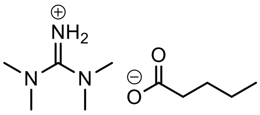

47. [TMG][Valerate]

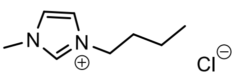

48. [BMIM][Cl]

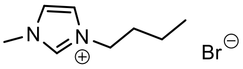

49. [BMIM][Br]

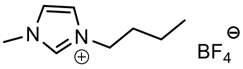

50. [BMIM][BF_4]

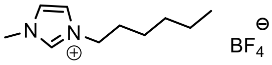

51. [HMIM][BF_4]

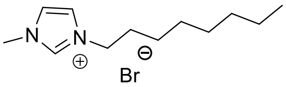

52. [OMIM][Br]

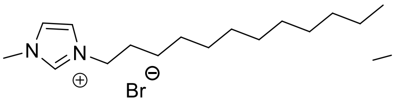

53. [DodecylMIM][Br]

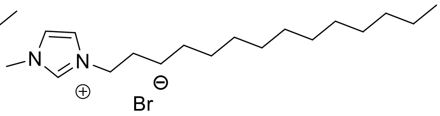

54. [TetradecylMIM][Br]

Supplementary Table 3 Screening results on the 54 kinds of water/IL systems [a].

Entry	Name of ILs	α Yield	β Yield	Ratio α/β
1	[DBU][Hexanoate]	24.0%	24.0%	1.0
2	[DBU][Valerate]	20.5%	26.1%	0.79
3	[DBU][Crotonate]	17.0%	30.7%	0.55
4	[DBU][Lactate]	35.2%	4.5%	7.8
5	[DBU][H_2PO_4]	27.3%	5.7%	4.8
6	[DBU][Propionate]	20.5%	14.8%	1.4
7	[DBU][DCA]	29.5%	5.7%	5.2
8	[DBU][Glycolate]	29.4%	4.0%	7.4
9	[DBU][Benzoate]	27.8%	8.9%	3.1
10	[DBU][TFA]	36.8%	6.0%	6.1
11	[Choline][Glycolate]	31.8%	4.5%	7.1
12	[Choline][Acetate]	28.4%	12.5%	2.3
13	[Choline][Hexanoate]	17.0%	6.8%	2.5
14	[Choline][Lactate]	36.4%	3.4%	10.7
15	[Choline][H_2PO_4]	29.5%	4.5%	6.6
16	[Choline][Benzoate]	51.1%	7.9%	6.5
17	[Choline][Crotonate]	27.3%	10.3%	2.7
18	[Choline][Butyrate]	10.7%	24.2%	0.4
19	[Choline][Valerate]	20.2%	27.4%	0.7
20	[Choline][3,5,5-Trimethylhexanoate]	10.1%	80.7%	0.1
21	[C4-Choline][Br]	28.4%	11.4%	2.5
22	[C8-Choline][Br]	29.5%	20.5%	1.4
23	[C12-Choline][Br]	34.0%	12.5%	2.7
24	[TEOA][Crotonate]	35.2%	5.7%	6.2
25	[TEOA][Hexanoate]	22.7%	5.7%	4.0
26	[TEOA][Isovalerate]	29.5%	9.1%	3.2
27	[TEOA][Butyrate]	25.0%	9.1%	2.7

28	[TEOA][Acetate]	37.5%	11.4%	3.3
29	[TEOA][Lactate]	23.9%	1.6%	14.9
30	[TEOA][Glycolate]	42.0%	5.1%	8.2
31	[TEOA][3,5,5-Trimethylhexanoate]	28.4%	12.5%	2.3
32	[DEOA][Hexanoate]	44.4%	20.9%	2.1
33	[DEOA][Butyrate]	53.3%	14.7%	3.6
34	[DEOA][Acetate]	61.9%	28.4%	2.2
35	[DEOA][Glycolate]	59.5%	5.1%	11.7
36	[DEOA][Lactate]	48.2%	2.0%	24.7
37	[DMEOA][Acetate]	13.1%	1.1%	11.7
38	[DMEOA][Lactate]	30.6%	1.3%	23.5
39	[MEOA][Butyrate]	16.9%	14.5%	1.2
40	[MEOA][Acetate]	18.9%	5.9%	3.2
41	[TMG][Isovalerate]	25.5%	5.4%	4.7
42	[TMG][TFA]	8.9%	2.8%	3.2
43	[TMG][DCA]	27.0%	5.9%	4.6
44	[TMG][Glycolate]	21.5%	1.8%	12.2
45	[TMG][Crotonate]	8.6%	22.8%	0.4
46	[TMG][Lactate]	11.4%	1.0%	11.7
47	[TMG][Valerate]	24.0%	14.2%	1.7
48	[BMIM][Cl]	31.5%	32.4%	1.0
49	[BMIM][Br]	15.1%	7.6%	2.0
50	[BMIM][BF_4]	10.6%	1.0%	10.6
51	[HMIM][BF_4]	42.4%	20.0%	2.1
52	[OMIM][Br]	22.0%	8.1%	2.7
53	[DodecylMIM][Br]	22.1%	33.5%	0.7
54	[TetradecylMIM][Br]	22.1%	25.8%	0.9

[a] α refers to α-ADPr-N_3, and β refers to β-ADPr-N_3, and the yield was determined by HPLC, and was calculated from a standard curve of α- and β-ADPr-N_3, the ratio of α/β was calculated based on the yields of α- and β-ADPr-N_3.

Supplementary Table 4 Improvements for the preparative reaction conditions of α-ADPr-N_3.

Entry[a]	H_2O:IL	Time	Temperature	substrate concentration	α Yield	β Yield	Ratio (α/β)
1	5:1	0.5 h	75 ℃	16 mM	24.4%	8.5%	2.9
2	5:1	0.5 h	80 ℃	16 mM	29.5%	9.1%	3.2
3	5:1	0.5 h	85 ℃	16 mM	38.1%	7.4%	5.1
4	5:1	0.5 h	90 ℃	16 mM	42.0%	5.1%	8.2
5	5:1	0.5 h	95 ℃	16 mM	44.3%	5.7%	7.7
6	5:1	0.5 h	100 ℃	16 mM	53.9%	7.4%	7.3
7	5:1	0.5 h	105 ℃	16 mM	55.1%	9.1%	6.1
8	5:1	0.5 h	110 ℃	16 mM	54.5%	10.2%	5.3
9	5:1	0.5 h	90 ℃	8 mM	47.7%	8.9%	5.4
10	5:1	0.5 h	90 ℃	16 mM	42.0%	5.1%	8.2
11	5:1	0.5 h	90 ℃	32 mM	86.6%	10.8%	8.0
12	5:1	0.5 h	90 ℃	48 mM	83.3%	9.8%	8.5
13 [b]	5:1	0.5 h	90 ℃	8 mM	32.0%	1.4%	22.9
14 [b]	5:1	0.5 h	90 ℃	16 mM	49.9%	2.0%	24.9
15 [b]	5:1	0.5 h	90 ℃	32 mM	84.1%	3.8%	22.6
16 [b]	5:1	0.5 h	90 ℃	48 mM	89.4%	3.5%	25.4

[a] IL = [TEOA][Glycolate], [b] IL = [DEOA][Lactate]

Supplementary Table 5 Chemical structure of the click reaction ligands.

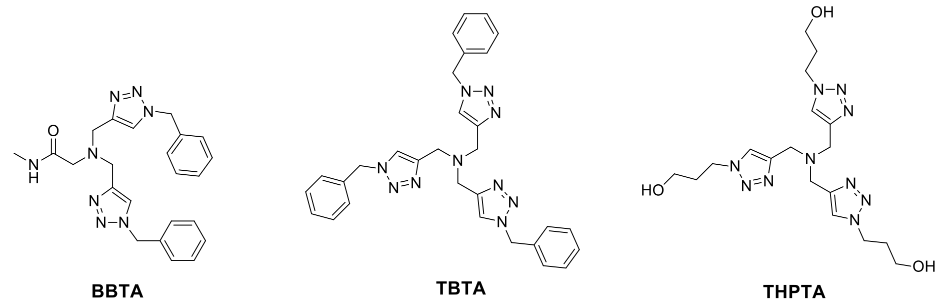

Supplementary Table 6 Results of ligand-promoted click reactions of α-ADPr-N_3.

Entry	Copper concentration	Ligand [a]	Time	Yield [b]
1	0.05 mM	--	6 h	0 %
2	0.05 mM	THPTA	6 h	54 %
3	0.05 mM	TBTA	6 h	0 %
4	0.05 mM	BBTA	6 h	0 %
5	0.09 mM	--	6 h	11 %
6	0.09 mM	THPTA	4 h	95 %
7	0.09 mM	TBTA	4 h	20 %
8	0.09 mM	BBTA	4 h	19 %
9	0.25 mM	--	4h	17 %
10	0.25 mM	THPTA	0.5 h	98 %
11	0.25 mM	TBTA	4 h	72 %
12	0.25 mM	BBTA	4 h	47 %

[a] 4.0 equivalence of copper sulfate. [b] Calculated based on the peak areas and the standard curve of the target compound.

Supplementary Table 7 Results of click reactions for preparations of ADP-ribosylated peptides.

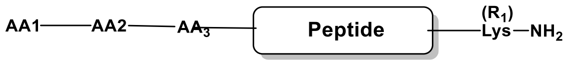

Compound	AA1	AA2	AA3	Peptide	R1	t_R	Time	Yield[a]
Peptide-1	10	Pro	9α	H2B(3-19)	Biotin	14.9min	4h	96%
Peptide-2	10	Pro	9β	H2B(3-19)	Biotin	16.2min	4h	97%
Peptide-3	--	Pro	9α	H2B(3-19)	--	9.3min	2h	98%
Peptide-4	--	Pro	9β	H2B(3-19)	--	9.4min	2h	98%
Peptide-5	10	Pro	9α	H2B(3-19)[b]	Biotin	14.9min	4h	97%

[a] Isolated yields; [b] Ser 7 and Ser 15 were mutated to Ala residues.

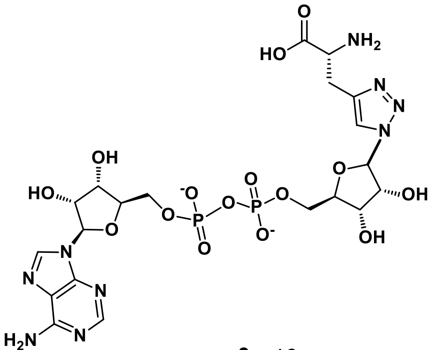

9 α / β

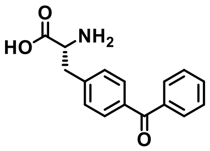

10

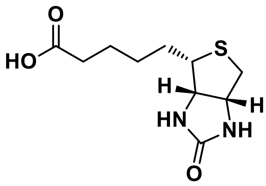

Biotin

H2B (3-19): PAK SAPAP KKGSK KAVT

Supplementary Table 8 Thermogravimetric analysis results and melting points of ionic liquids.

Name of IL	T_{onset} / T_{mp} (°C)	Name of IL	T_{onset} / T_{mp} (°C)
[DBU][Hexanoate]	169.7 / -[a]	[TEOA][Hexanoate]	158.0 / -[a]
[DBU][Valerate]	237.9 / -[a]	[TEOA][Isovalerate]	166.5 / -[a]
[DBU][Crotonate]	233.2 / -[a]	[TEOA][Butyrate]	152.5 / -[a]
[DBU][Lactate]	215.7 / -[a]	[TEOA][Acetate]	175.4 / -[a]
[DBU][H_2PO_4]	192.8 / -[a]	[TEOA][Lactate]	230.3 / -[a]
[DBU][Propionate]	227.9 / -[a]	[TEOA][Glycolate]	227.8 / -[a]
[DBU][DCA]	208.0 / -[a]	[TEOA][3,5,5-Trimethylhexanoate]	172.2 / -[a]
[DBU][Glycolate]	209.0 / -[a]	[DEOA][Hexanoate]	169.8 / -[a]
[DBU][Benzoate]	217.6 / -[a]	[DEOA][Butyrate]	149.6 / -[a]
[DBU][TFA]	189.3 / -[a]	[DEOA][Acetate]	169.2 / -[a]
[Choline][Glycolate]	192.4 / -[a]	[DEOA][Glycolate]	169.3 / -[a]
[Choline][Acetate]	187.0 / -[a]	[DEOA][Lactate]	234.3 / -[a]
[Choline][Hexanoate]	190.5 / -[a]	[DMEOA][Acetate]	201.0 / -[a]
[Choline][Lactate]	219.8/ -[a]	[DMEOA][Lactate]	231.8 / -[a]
[Choline][H_2PO_4]	166.0 / -[a]	[MEOA][Butyrate]	172.3 / -[a]
[Choline][Benzoate]	204.7 / -[a]	[MEOA][Acetate]	116.7 / -[a]
[Choline][Crotonate]	200.1 / -[a]	[TMG][Isovalerate]	138.7 / -[a]
[Choline][Butyrate]	192.8 / -[a]	[TMG][TFA]	185.3 / -[a]
[Choline][Valerate]	190.5 / -[a]	[TMG][DCA]	177.1 / -[a]
[Choline][3,5,5-Trimethylhexanoate]	186.3/ -[a]	[TMG][Glycolate]	190.5 / -[a]
[C4-Choline][Br]	244.6 / 115.7	[TMG][Crotonate]	192.6 / -[a]
[C8-Choline][Br]	237.5 / 124.9	[TMG][Lactate]	210.9 / -[a]
[C12-Choline][Br]	232.3 / 80.2	[TMG][Valerate]	200.7 / -[a]
[TEOA][Crotonate]			

[a] liquid at room temperature.

Supplementary Table 9 Microanalysis of water contents of ionic liquids.

**Name of IL**	**Water content (ppm)**	**Name of IL**	**Water content (ppm)**
[DBU][Hexanoate]	2654	[TEOA][Hexanoate]	2877
[DBU][Valerate]	2906	[TEOA][Isovalerate]	3356
[DBU][Crotonate]	3982	[TEOA][Butyrate]	3177
[DBU][Lactate]	2430	[TEOA][Acetate]	3558
[DBU][H_2PO_4]	1033	[TEOA][Lactate]	3979
[DBU][Propionate]	2917	[TEOA][Glycolate]	3559
[DBU][DCA]	1853	[TEOA][3,5,5-Trimethylhexanoate]	3288
[DBU][Glycolate]	2521	[DEOA][Hexanoate]	3011
[DBU][Benzoate]	1556	[DEOA][Butyrate]	2926
[DBU][TFA]	585	[DEOA][Acetate]	3352
[Choline][Glycolate]	4113	[DEOA][Glycolate]	3955
[Choline][Acetate]	3269	[DEOA][Lactate]	3960
[Choline][Hexanoate]	4881	[DMEOA][Acetate]	3775
[Choline][Lactate]	2777	[DMEOA][Lactate]	3879
[Choline][H_2PO_4]	3904	[MEOA][Butyrate]	2405
[Choline][Benzoate]	3075	[MEOA][Acetate]	3249
[Choline][Crotonate]	2669	[TMG][Isovalerate]	3588
[Choline][Butyrate]	955	[TMG][TFA]	836
[Choline][Valerate]	1278	[TMG][DCA]	1778
[Choline][3,5,5-Trimethylhexanoate]	3488	[TMG][Glycolate]	1093
[C4-Choline][Br]	1552	[TMG][Crotonate]	943
[C8-Choline][Br]	978	[TMG][Lactate]	1273
[C12-Choline][Br]	821	[TMG][Valerate]	1454
[TEOA][Crotonate]	2997		

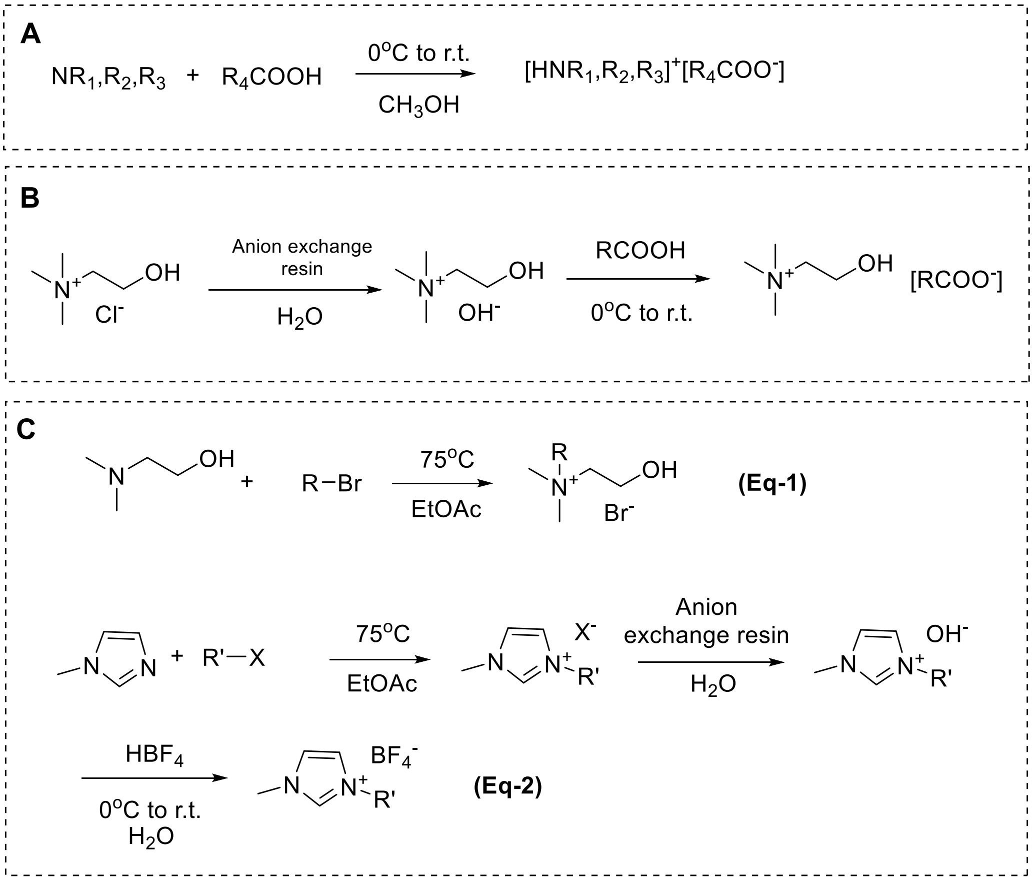

Supplementary Figure 1 Synthesis routes of ionic liquids. (A) Method-(1) for preparations of protonic ionic liquids. (B) Method-(2) for preparations of choline-based ionic liquids. (C) Method-(3) for preparations of brominated ionic liquids and imidazole-type ionic liquids.

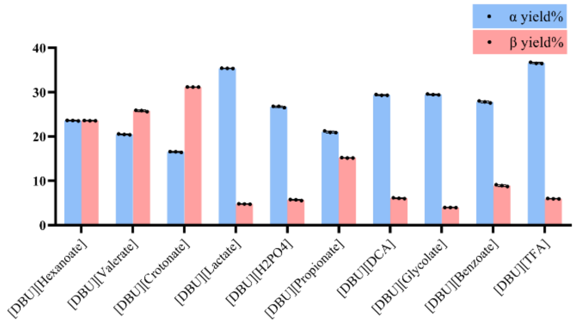

Supplementary Figure 2 Yields of α/β-ADPr-N_3 with DBU-based ionic liquids. Experiments were performed in triplicates. Data are reported as mean ± s.d. (n = 3), and black dots indicate value of each sample. Raw data for the figure are available at doi:10.6084/m9.figshare.12375032.

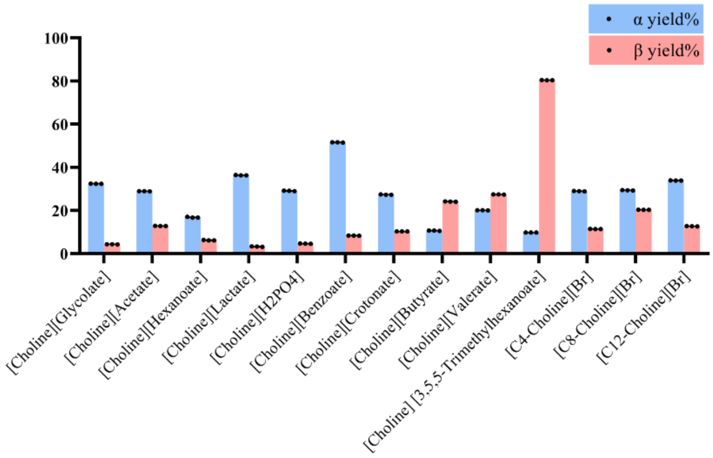

Supplementary Figure 3 Yields of α/β-ADPr-N_3 with Choline-based ionic liquids. Data are reported as mean ± s.d. (n = 3), and black dots indicate value of each sample. Raw data for the figure are available at doi:10.6084/m9.figshare.12375032.

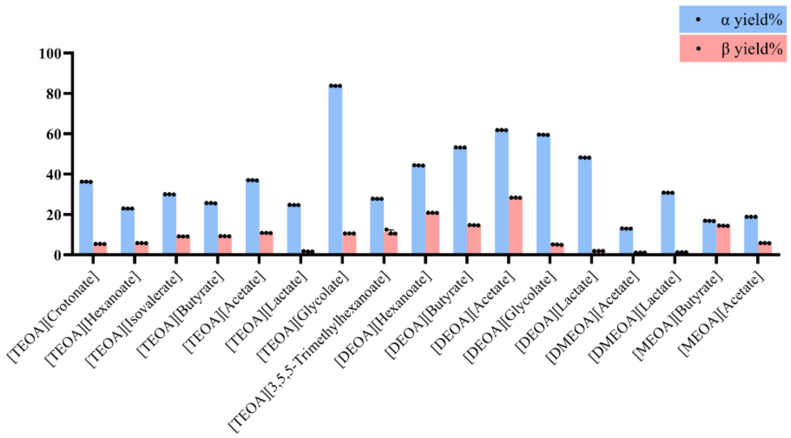

Supplementary Figure 4 Yields of α/β-ADPr-N_3 with alkylolamine-based ionic liquids. Data are reported as mean ± s.d. (n = 3), and black dots indicate value of each sample. Raw data for the figure are available at doi:10.6084/m9.figshare.12375032.

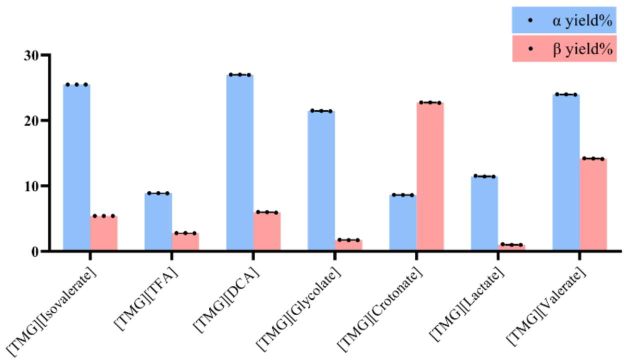

Supplementary Figure 5 Yields of α/β-ADPr-N_3 with TMG-based ionic liquids. Data are reported as mean ± s.d. (n = 3), and black dots indicate value of each sample. Raw data for the figure are available at doi:10.6084/m9.figshare.12375032.

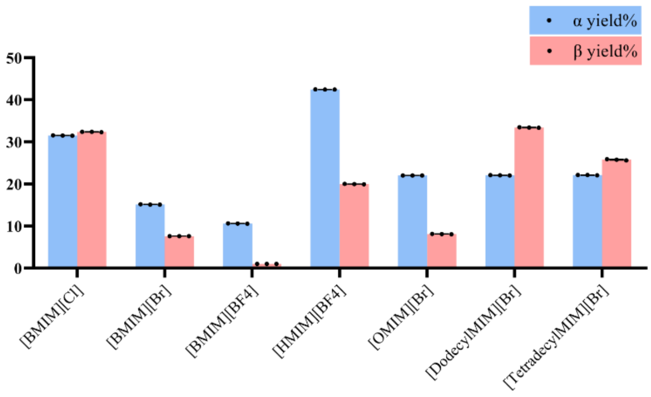

Supplementary Figure 6 Yields of α/β-ADPr-N_3 with of Imidazole-based ionic liquids. Data are reported as mean ± s.d. (n = 3), and black dots indicate value of each sample. Raw data for the figure are available at doi:10.6084/m9.figshare.12375032.

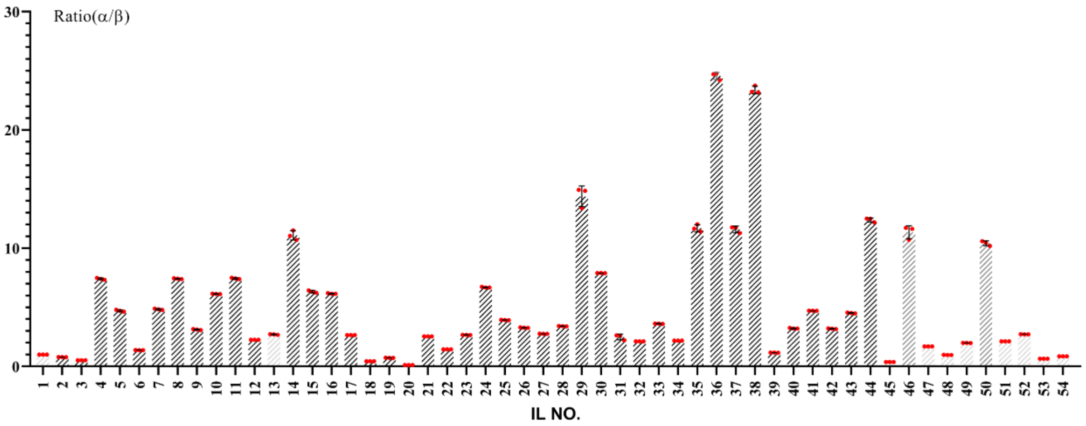

Supplementary Figure 7 Ratio of α/β-ADPr-N_3 in different ionic liquid/water reaction systems. Data are reported as mean ± s.d. (n = 3), and black dots indicate value of each sample. Raw data for the figure are available at doi:10.6084/m9.figshare.12375032.

Peptide-1:

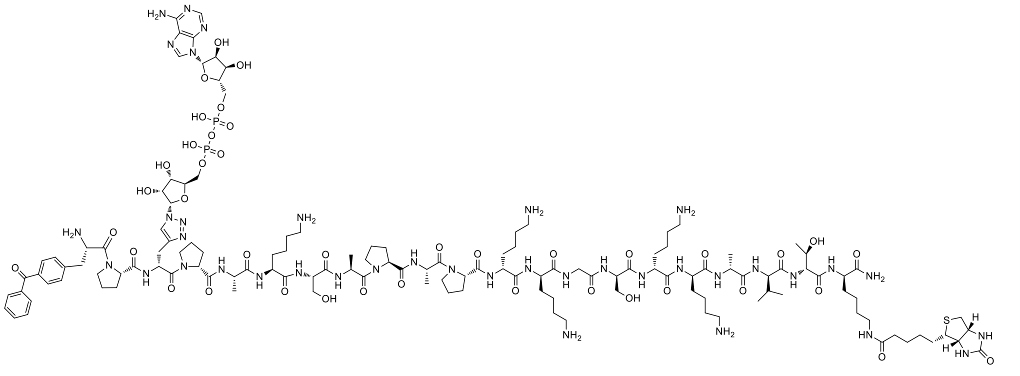

Peptide-2:

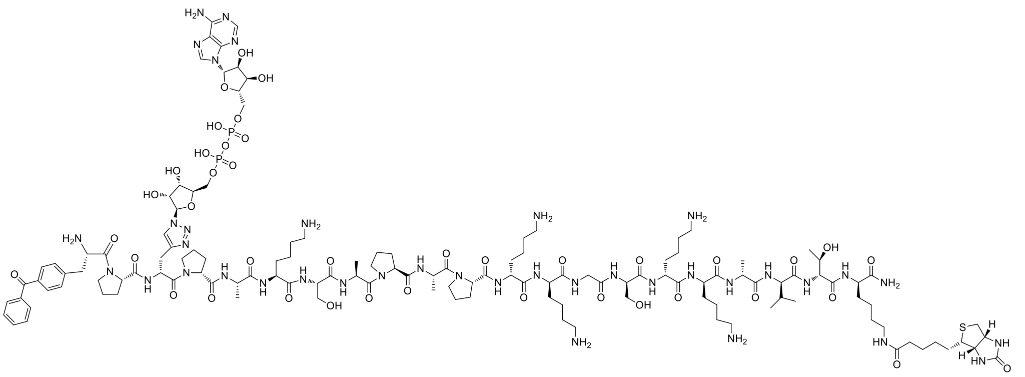

Peptide-3:

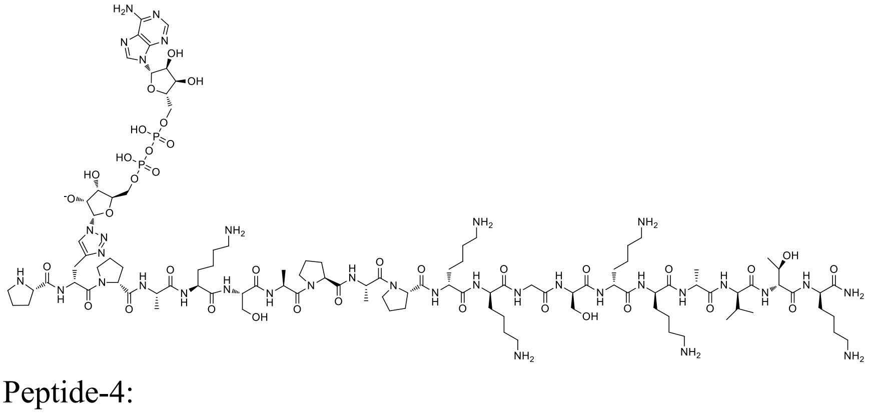

Peptide-4:

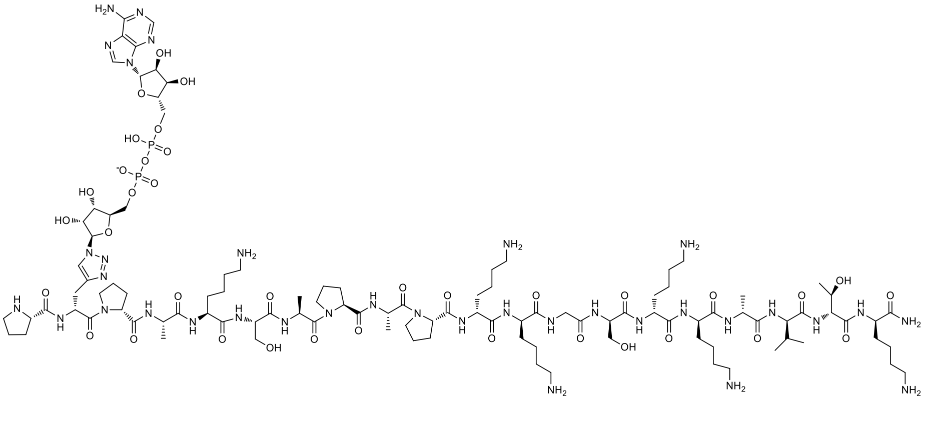

Peptide-5:

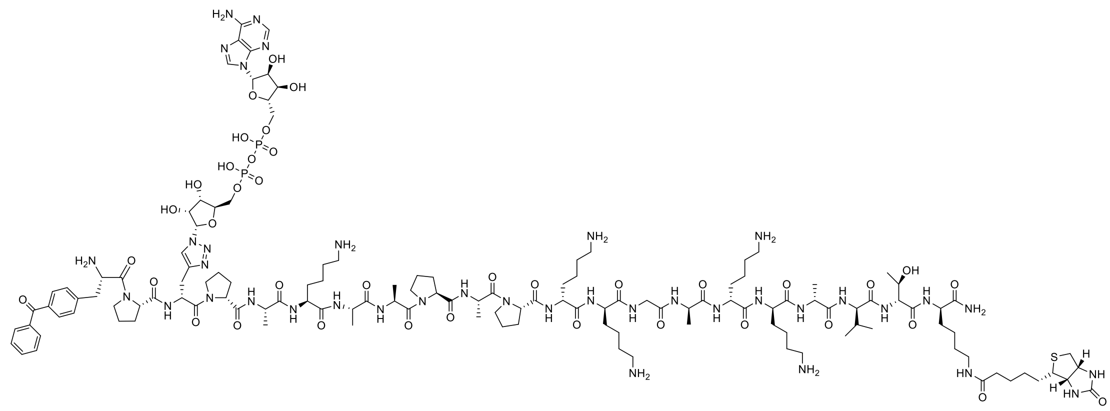

Supplementary Figure 8 Chemical structures of ADP-ribosylated peptides.

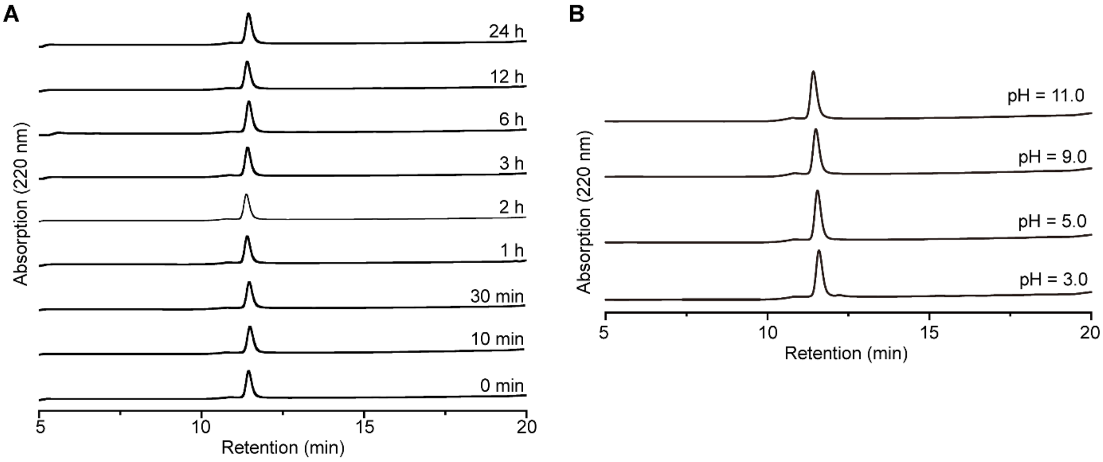

Supplementary Figure 9 Estimation of the stability of ADP-ribosylated peptide. (A) Peptide-3 was stable toward NH_2OH treatment (0.5 M NH_2OH in phosphate buffer, pH = 7.5, 37 ºC). (B) Peptide-3 was stable under acidic and basic conditions (37 ºC, 24 h).

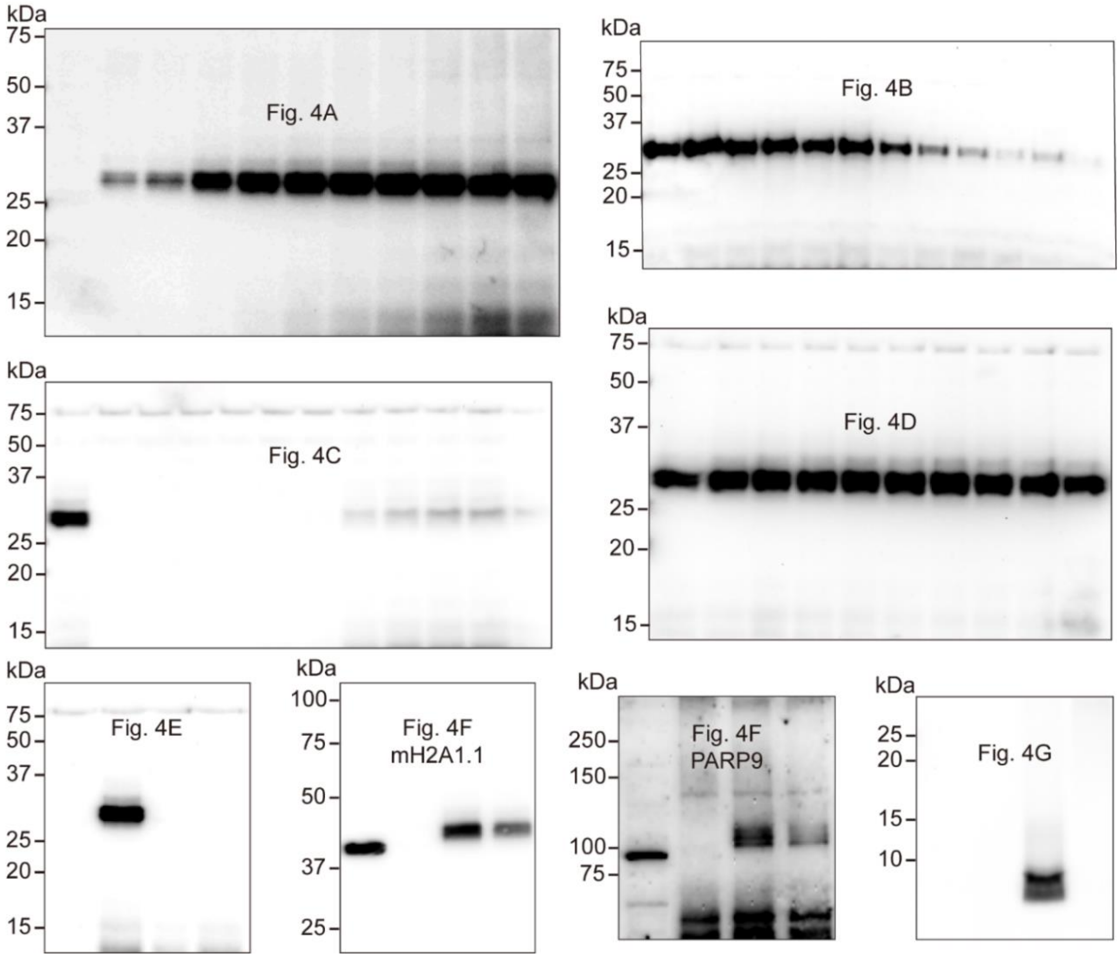

Supplementary Figure 10 Uncropped blots of Fig. 4.

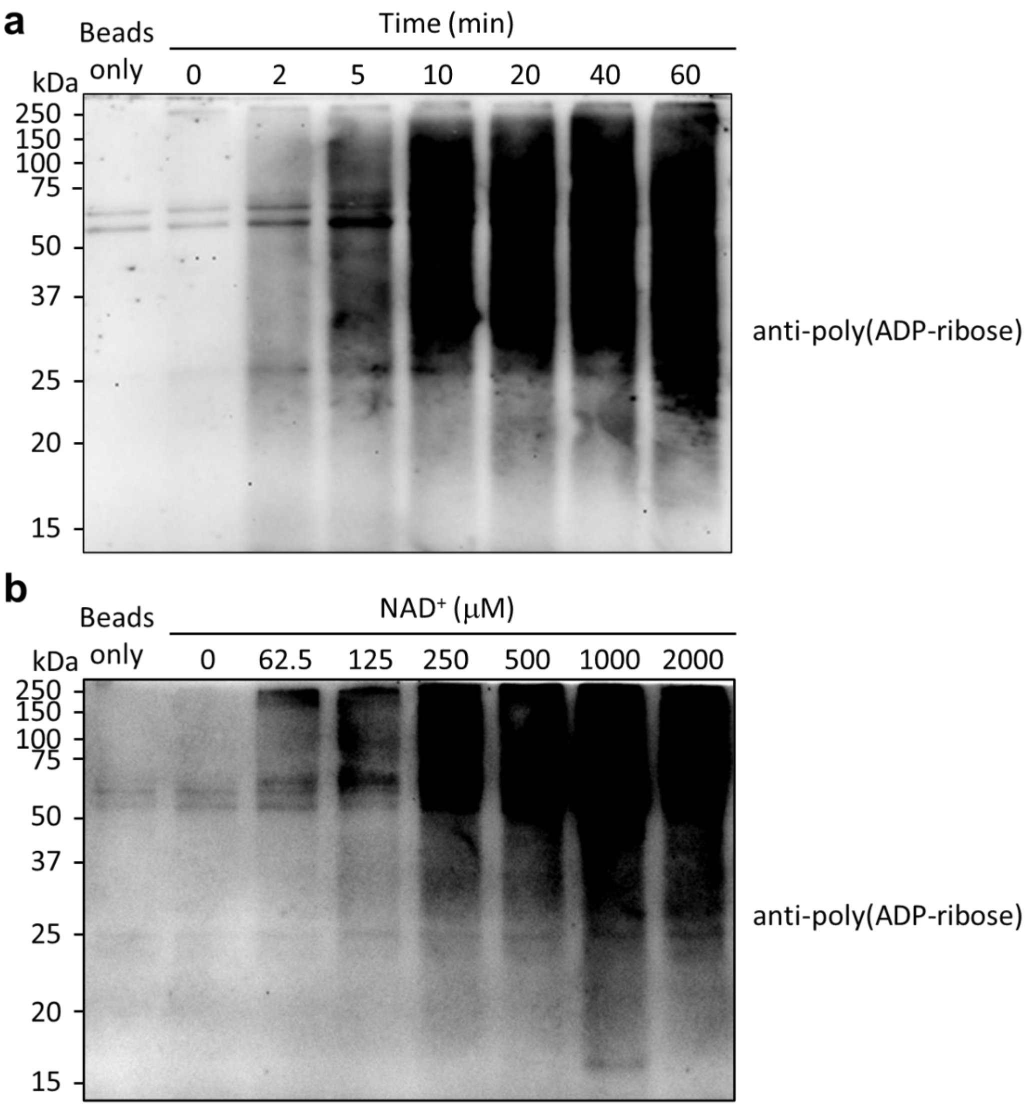

Supplementary Figure 11 The poly-ADP-ribosylation of peptide-1. **a** The poly-ADP-ribosylation of peptide-1 by PARP1 is time-dependent. **b** The poly-ADP-ribosylation of peptide-1 by PARP1 is NAD^+ concentration-dependent. In **a** and **b**, peptide-1 (15 μM) in reaction buffer (50 mM Tris pH 8.0, 10 mM $MgCl_2$, 1 mM DTT) containing 1× activated DNA was added NAD^+ and PARP1 (1 μL, 10 units/μL). Total volume of each reaction was 20 mL. For samples in **a**, the NAD^+ concentration was 2 mM, and the reactions were incubated for indicated time points. For samples in **a**, the reactions were incubated for 60 min with indicated concentrations of NAD^+. After the reaction, the mixtures were subjected to Dynabeads enrichment prior to immunoblotting analysis against anti-poly(ADP-ribose) polymer antibody. Samples with Dynabeads only were prepared as control. The blottings represent three independent experiments.

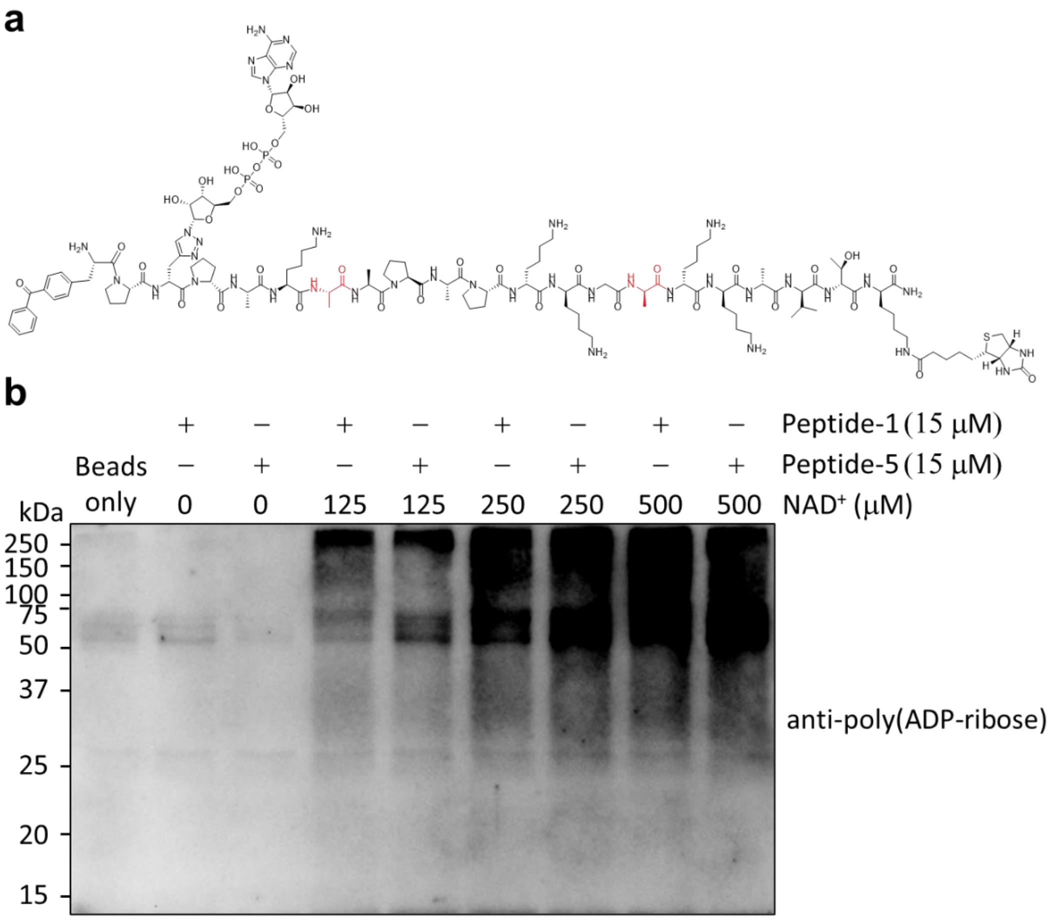

Supplementary Figure 12 The preexisting triazolyl-ADPr mark is the preferred modification site of the PARP1-catalyzed poly-ADP-ribosylation. **a** Structure of peptide-5. Highlighted in red are the original Ser 7 and Ser 15 residues mutated to Ala. **b** Comparison of peptide-1 and peptide-5 in poly-ADP-ribosylation reactions. The peptides (15 μM) in reaction buffer (50 mM Tris pH 8.0, 10 mM $MgCl_2$, 1 mM DTT) containing 1× activated DNA was added indicated concentrations of NAD^+ and PARP1 (1 μL, 10 units/μL). Total volume of each reaction was 20 μL. The reactions were incubated for 60 min. After the reaction, the mixtures were subjected to Dynabeads enrichment prior to immunoblotting analysis against anti-poly(ADP-ribose) polymer antibody. Sample with Dynabeads only was prepared as control. The blotting represents three independent experiments.

Characterization of synthesized ionic liquids

IL-1: [DBU][Hexanoate]

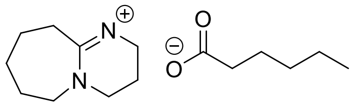

^{1}H NMR (600 MHz, D_2O) δ 3.57 - 3.55 (m, 2H), 3.53 - 3.51 (m, 2H), 3.32 - 3.31 (m, 2H), 2.63 - 2.61 (m, 2H), 2.16 (t, *J* = 7.2 Hz, 2H), 2.02 - 2.00 (m, 2H), 1.75 - 1.68 (m, 6H), 1.57 - 1.52 (m, 2H), 1.32 - 1.25 (m, 4H), 0.87 (t, *J* = 7.2 Hz, 3H) ppm; ^{13}C NMR (100 MHz, D_2O) δ 182.8, 165.9, 54.1, 48.2, 38.0, 37.8, 32.6, 31.3, 28.5, 26.0, 25.8, 23.5, 22.0, 19.0, 13.6 ppm.

IR: ν 3242, 2928, 2858, 1644, 1558, 1447, 1386, 1322, 1205, 1158, 1099, 1052, 884, 882 cm^{-1}.

IL-2: [DBU][Valerate]

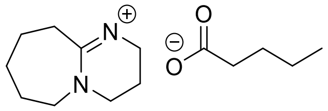

^{1}H NMR (600 MHz, D_2O) δ 3.57 - 3.56 (m, 2H), 3.53 - 3.51 (m, 2H), 3.33 - 3.31 (m, 2H), 2.63 - 2.61 (m, 2H), 2.17 (t, *J* = 7.4 Hz, 2H), 2.02 - 2.00 (m, 2H), 1.75 - 1.73 (m, 6H), 1.55 - 1.49 (m, 2H), 1.33 - 1.27 (m, 2H), 0.89 (t, *J* = 7.4 Hz, 3H) ppm; ^{13}C NMR (100 MHz, D_2O) δ 183.2, 165.8, 54.0, 48.1, 37.9, 37.5, 32.6, 28.4, 28.2, 25.9, 23.4, 22.1, 18.9, 13.3 ppm.

IR: ν 2929, 2859, 1642, 1554, 1446, 1384, 1322, 1202, 1158, 1107, 689 cm^{-1}.

IL-3: [DBU][Crotonate]

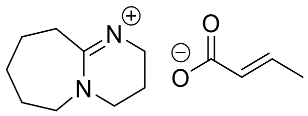

^{1}H NMR (600 MHz, D_2O) δ 6.61 - 6.55 (m, 1H), 5.79 (d, *J* = 15.6 Hz, 1H), 3.51 - 3.49 (m, 2H), 3.46 (t, *J* = 5.8 Hz, 2H)2, 3.26 (t, *J* = 5.6 Hz, 2H), 2.57 - 2.56 (m, 2H), 1.96 - 1.95 (m, 2H), 1.76 (d, *J* = 6.8 Hz, 3H), 1.69 - 1.62 (m, 6H) ppm; ^{13}C NMR (100 MHz,

D_2O) δ 174.4, 165.8, 140.8, 127.6, 54.0, 48.1, 37.9, 32.6, 28.4, 25.9, 23.4, 18.9, 17.1 ppm.

IR: ν 2925, 2861, 2466, 1894, 1637, 1545, 1378, 1350, 1319, 1279, 1157, 974, 897, 715, 685, 635, 529 cm^{-1}.

IL-4: [DBU][Lactate]

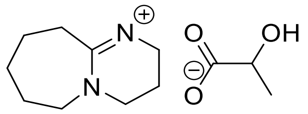

^1H NMR (600 MHz, D_2O) δ 4.06 - 4.03 (m, 1H), 3.54 - 3.52 (m, 2H), 3.50 - 3.48 (m, 2H), 3.29 - 3.27 (m, 2H), 2.60 - 2.58 (m, 2H), 1.98 - 1.96 (m, 2H), 1.71 - 1.65 (m, 6H), 1.29 (d, J = 7.2 Hz, 3H) ppm; ^{13}C NMR (100 MHz, D_2O) δ 181.6, 165.8, 68.3, 54.0, 48.1, 37.9, 32.5, 28.4, 25.9, 23.3, 20.3, 18.9 ppm.

IR: ν 2930, 2862, 1741, 1644, 1596, 1447, 1369, 1321, 1205, 1111, 1032, 842, 691,529 cm^{-1}.

IL-5: [DBU][H_2PO_4]

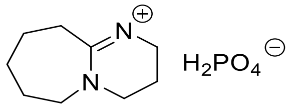

^1H NMR (600 MHz, D_2O) δ 3.58 - 3.57 (m, 2H), 3.54 - 3.52 (m, 2H), 3.34 - 3.32 (m, 2H), 2.64 - 2.62 (m, 2H), 2.04 - 2.00 (m, 2H), 1.76 - 1.69 (m, 6H) ppm; ^{13}C NMR (100 MHz, D_2O) δ 165.9, 54.1, 48.2, 37.9, 32.8, 28.4, 25.8, 23.3, 18.9 ppm. ^{31}P NMR (162 MHz, D_2O) δ -0.08.

IR: ν 2925, 2856, 2326, 1644, 1604, 1348, 1252, 1203, 1063, 939, 865, 688,516 cm^{-1}.

IL-6: [DBU][Propionate]

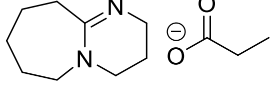

^1H NMR (600 MHz, D_2O) δ 3.58 - 3.56 (m, 2H), 3.54 - 3.52 (m, 2H), 3.33 - 3.32 (m, 2H), 2.64 - 2.62 (m, 2H), 2.19 - 2.18 (m, 2H), 2.04 - 2.00 (m, 2H), 1.76 - 1.74 (m, 6H),

1.06 (t, J = 7.6 Hz, 3H) ppm; ^{13}C NMR (100 MHz, D_2O) δ 184.0, 165.8, 54.0, 48.1, 37.9, 32.6, 30.7, 28.4, 25.8, 23.3, 18.9, 10.2 ppm.

IR: ν 2929, 2861, 1641, 1556, 1460, 1387, 1359, 1322, 1284, 1203, 863, 689, 528 cm^{-1}.

IL-7: [DBU][DCA]

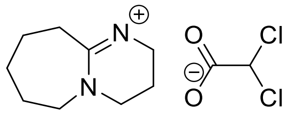

^{1}H NMR (600 MHz, D_2O) δ 6.05 (s, 1H), 3.59 - 3.57 (m, 2H), 3.55 - 3.53 (m, 2H), 3.35 - 3.33 (m, 2H), 2.65 - 2.63 (m, 2H), 2.04 - 2.02 (m, 2H), 1.76 - 1.70 (m, 6H) ppm; ^{13}C NMR (100 MHz, D_2O) δ 169.5, 165.9, 68.7, 54.2, 48.3, 38.1, 32.8, 28.6, 26.1, 23.5, 19.1 ppm.

IR: ν 2931, 1639, 1446, 1350, 1322, 1205, 1106, 984,821, 764, 690 cm^{-1}.

IL-8: [DBU][Glycolate]

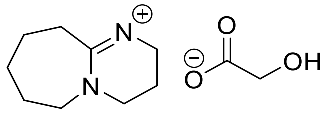

^{1}H NMR (600 MHz, D_2O) δ 3.93 (s, 2H), 3.56 - 3.55 (m, 2H), 3.52 - 3.50 (m, 2H), 3.32 - 3.30 (m, 2H), 2.62 - 2.60 (m, 2H), 2.02 - 1.98 (m, 2H), 1.73 - 1.67 (m, 6H) ppm; ^{13}C NMR (100 MHz, D_2O) δ 178.9, 165.8, 61.1, 54.1, 48.2, 37.9, 32.6, 28.4, 25.9, 23.3, 18.9 ppm.

IR: ν 2927, 1643, 1604, 1355, 1321, 1205, 1107, 1057, 985, 895, 689, 589, 505 cm^{-1}.

IL-9: [DBU][Benzoate]：

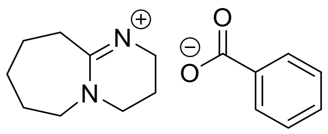

^{1}H NMR (600 MHz, D_2O) δ 7.88 - 7.86 (m, 2H), 7.49 - 7.48 (m, 1H), 7.45 - 7.42 (m, 2H), 3.34 - 3.33 (m, 2H), 3.29 - 3.28 (m, 2H), 3.14 - 3.13 (m, 2H), 2.42 - 2.40 (m, 2H),

1.83 - 1.81 (m, 2H), 1.59 - 1.50 (m, 6H) ppm; ^{13}C NMR (100 MHz, D_2O) δ 173.8, 165.4, 135.9, 131.2, 129.0, 128.2, 53.8, 47.8, 37.7, 32.4, 28.2, 25.7, 23.1, 18.7 ppm.

IR: ν 2938, 2855, 1647, 1596, 1553, 1354, 1325, 1207, 1159, 1060, 820, 717, 691, 669, 528 cm^{-1}.

IL-10: [DBU][TFA]：

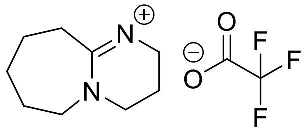

^{1}H NMR (600 MHz, D_2O) δ 3.57 (s, 2H), 3.54 (s, 2H), 3.33 (s, 2H), 2.63 (s, 2H), 2.03 (s, 2H), 1.74 - 1.70 (m, 6H) ppm; ^{13}C NMR (100 MHz, D_2O) δ 165.9, 162.3 (q), 116.5 (q), 54.1, 48.2, 37.9, 32.7, 28.4, 25.9, 23.3, 18.9 ppm. ^{19}F NMR (376 MHz, D_2O) δ -75.7.

IR: ν 2934, 1687, 1641, 1448, 1324, 1195, 1162, 1108, 985, 821, 797, 717 cm^{-1}.

IL-11: [Choline][Glycolate]：

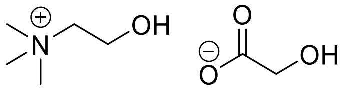

^{1}H NMR (600 MHz, D2O) δ 4.07 (d, J = 2.8 Hz, 2H), 3.95 (s, 2H), 3.53 - 3.52 (m, 2H), 3.21 (s, 9H) ppm; ^{13}C NMR (100 MHz, D2O) δ 178.4, 67.4, 60.6, 55.6, 53.8 ppm.

IR: ν 3191, 2853, 1590, 1480, 1358, 1079, 1006, 954, 900, 685 cm^{-1}.

IL-12: [Choline][Acetate]：

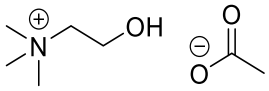

^{1}H NMR (600 MHz, D_2O) δ 4.07 - 4.07 (m, 2H), 3.53 - 3.52 (m, 2H), 3.21 (s, 9H), 1.93 (s, 3H) ppm; ^{13}C NMR (100 MHz, D_2O) δ 178.5, 67.3, 55.5, 53.8, 21.6 ppm.

IR: ν 3144, 1568, 1481, 1384, 1330, 1139, 1089, 1008, 954, 915, 867, 640, 463 cm^{-1}.

IL-13: [Choline][Hexanoate]：

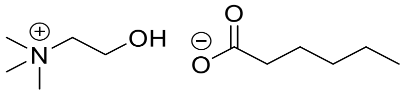

^1H NMR (600 MHz, D_2O) δ 4.07 (s, 2H), 3.53 - 3.52 (m, 2H), 3.21 (s, 9H), 2.17 (t, *J* = 7.4 Hz, 2H), 1.58 - 1.53 (m, 2H), 1.33 - 1.25 (m, 4H), 0.88 (t, *J* = 7.0 Hz, 3H) ppm; ^{13}C NMR (100 MHz, D_2O) δ 183.6, 67.4, 55.6, 53.8, 37.3, 31.0, 25.4, 21.8, 13.3 ppm.

IR: ν 2928, 2858, 1567, 1481, 1384, 1304, 1091, 955, 867, 614, 555, 454 cm^{-1}.

IL-14: [Choline][Lactate]：

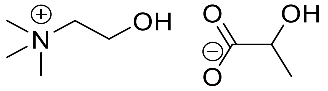

^1H NMR (600 MHz, D_2O) δ 4.14 - 4.11 (m, 1H), 4.08 (s, 2H), 3.54 - 3.53 (m, 2H), 3.21 (s, 9H), 1.34 (d, *J* = 6.8 Hz, 3H) ppm; ^{13}C NMR (100 MHz, D_2O) δ 181.9, 68.5, 68.3, 55.6, 53.5, 20.1 ppm.

IR: ν 3204, 1592, 1479, 1402, 1341, 1119, 1087, 1032, 954, 865, 772, 645, 531 cm^{-1}.

IL-15: [Choline][H_2PO_4]：

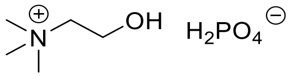

^1H NMR (600 MHz, D_2O) δ 4.07 (d, *J* = 2.4 Hz, 2H), 3.53 (d, *J* = 4.8 Hz, 2H), 3.21 (s, 9H) ppm; ^{13}C NMR (100 MHz, D_2O) δ 67.3, 55.5, 53.8 ppm. ^{31}P NMR (162 MHz, D_2O) δ -0.13.

IR: ν 3037, 2852, 2338, 1596, 1480, 1239, 1077, 936, 865, 507 cm^{-1}.

IL-16: [Choline][Benzoate]：

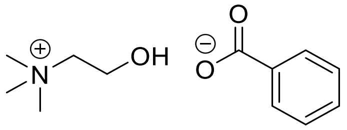

^1H NMR (600 MHz, D_2O) δ 7.90 - 7.89 (m, 2H), 7.57 (t, *J* = 7.2 Hz, 1H), 7.51 - 7.48 (m, 2H), 4.06 - 4.05 (m, 2H), 3.51 - 3.50 (m, 2H), 3.19 (s, 9H) ppm; ^{13}C NMR (100 MHz, D_2O) δ 175.0, 136.1, 131.3, 128.9, 128.4, 67.2, 55.5, 53.7 ppm.

IR: ν 3024, 1597, 1558, 1479, 1359, 1137, 1089, 953, 826, 719, 669 cm^{-1}.

IL-17: [Choline][Crotonate]：

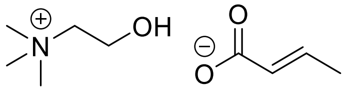

^{1}H NMR (600 MHz, D_2O) δ 6.71 - 6.66 (m, 1H), 5.85 (d, *J* = 15.6 Hz, 1H), 4.07 - 4.06 (m, 2H), 3.53 - 3.51 (m, 2H), 3.21 (s, 9H), 1.83 (d, *J* = 6.8 Hz, 3H) ppm; ^{13}C NMR (100 MHz, D_2O) δ 173.3, 144.2, 124.8, 67.4, 55.5, 53.8, 17.2 ppm.

IR: ν 3024, 2854, 1656,1558, 1480, 1358, 1286, 1242, 1090, 954, 862, 716, 555 cm^{-1}.

IL-18: [Choline][Butyrate]：

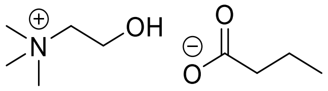

^{1}H NMR (600 MHz, D_2O) δ 4.08 (d, *J* = 3.8 Hz, 2H), 3.54 - 3.52 (m, 2H), 3.21 (s, 9H), 2.18 (t, *J* = 7.2 Hz, 2H), 1.59 - 1.56 (m, 2H), 0.91 (t, *J* = 7.4 Hz, 3H) ppm; ^{13}C NMR (100 MHz, D_2O) δ 183.1, 67.4, 55.5, 53.8, 39.2, 19.2, 13.2 ppm.

IR: ν 3174, 1563, 1481, 1387, 1305, 1090, 955, 868, 611 cm^{-1}.

IL-19: [Choline][Valerate]：

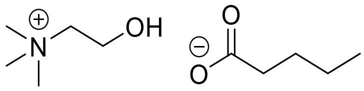

^{1}H NMR (600 MHz, D_2O) δ 4.07 (s, 2H), 3.53 - 3.52 (m, 2H), 3.21 (s, 9H), 2.19 (t, *J* = 7.0 Hz, 2H), 1.55 - 1.52 (m, 2H), 1.33 - 1.29 (m, 2H), 0.91 - 0.89 (m, 3H) ppm; ^{13}C NMR (100 MHz, D_2O) δ 183.8, 67.4, 55.5, 53.8, 37.3, 28.1, 22.0, 13.2 ppm. IR: ν 3175, 2956, 2861, 1563, 1480, 1388, 1309, 1090, 954, 867, 646 cm^{-1}.

IL-20: [Choline][3,5,5-Trimethylhexanoate]：

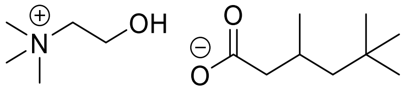

^{1}H NMR (600 MHz, D_2O) δ 4.07 – 4.06 (m, 2H), 3.54 - 3.52 (m, 2H), 3.21 (s, 9H), 2.24 - 2.20 (m, 1H), 1.97 - 1.95 (m, 2H), 1.24 (dd, *J* = 13.8, 3.0 Hz, 1H), 1.12 (dd, *J* = 13.9,

5.9 Hz, 1H) 0.95 - 0.91 (m, 12H) ppm; ^{13}C NMR (150 MHz, D_2O) δ 183.4, 67.4, 55.6, 53.8, 50.3, 47.9, 30.0, 29.4, 27.5, 22.2 ppm.

IR: ν 3021, 2949, 1566, 1464, 1379, 1363, 1107. 1075, 966, 954, 896, 631, 454 cm^{-1}.

IL-21: [C4-Choline][Br]：

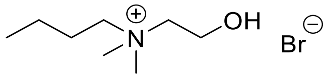

^{1}H NMR (400 MHz, D_2O) δ 4.07 - 4.06 (m, 2H), 3.52 - 3.50 (m, 2H), 3.42 - 3.38 (m, 2H), 3.15 (s, 6H), 1.83 - 1.75 (m, 2H), 1.43 - 1.38 (m, 2H), 0.97 (t, J = 7.4 Hz, 3H) ppm; ^{13}C NMR (150 MHz, D_2O) δ 65.4, 64.9, 55.4, 51.4, 24.0, 19.1, 12.9 ppm.

IR: ν 3248, 3011, 2964, 2873, 1487, 1459, 1047, 1027, 991, 960, 936, 918, 903, 807, 735, 583, 548, 498, 456 cm^{-1}.

Elem. Anal. Calcd for C16H36BrNO: C, 42.49%; H, 8.19%; N, 6.19%. Found: C, 42.17%; H, 9.15%; N, 5.98%.

IL-22: [C8-Choline][Br]：

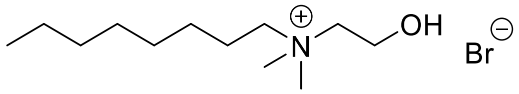

^{1}H NMR (400 MHz, D_2O) δ 4.09 (s, 2H), 3.57 - 3.54 (m, 2H), 3.46 - 3.42 (m, 2H), 3.20 (s, 6H), 1.86 - 1.82 (m, 2H), 1.42 - 1.34 (m, 10H), 0.93 (t, J = 6.6 Hz, 3H) ppm; ^{13}C NMR (150 MHz, D_2O) δ 65.5, 65.0, 55.5, 51.6, 31.3, 28.6, 28.5, 25.8, 22.3, 22.2, 13.8 ppm.

IR: ν 3339, 3245, 3017, 2948, 2918, 2852, 1491, 1479, 1468, 1415, 1376, 1341, 1092, 1060, 1047, 1001, 979, 964, 939, 926, 853, 753, 722, 603, 562, 510, 464 cm^{-1}.

Elem. Anal. Calcd for C16H36BrNO: C, 51.06%; H, 10.00%; N, 4.96%. Found: C, 50.88%; H, 10.03%; N, 4.65%.

IL-23: [C12-Choline][Br]：

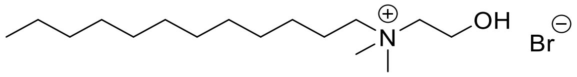

^{1}H NMR (400 MHz, D_2O) δ 4.06 (s, 2H), 3.54 - 3.52 (m, 2H), 3.45 - 3.40 (m, 2H), 3.18 (s, 6H), 1.81 (s, 2H), 1.39 -1.32 (m, 18H), 0.90 (t, *J* = 6.2 Hz, 3H) ppm; ^{13}C NMR (100 MHz, D_2O) δ 65.2, 65.1, 55.4, 51.6, 31.9, 29.8, 29.7, 29.6, 29.5, 29.4, 29.1, 26.1, 22.6, 22.5, 13.9 ppm.

IR: ν 3240, 3007, 2952, 2916, 2849, 1469, 1376, 1257, 1218, 1132, 1097, 1079, 1057, 1010, 992, 963, 918, 856, 840, 763, 719, 599, 568, 517, 500, 461, 435 cm^{-1}.

Elem. Anal. Calcd for C16H36BrNO: C, 56.79%; H, 10.72%; N, 4.14%. Found: C, 56.70%; H, 10.75%; N, 4.03%.

IL-24: [TEOA][Crotonate]：

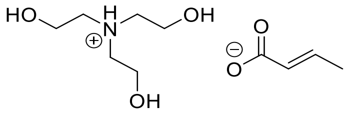

^{1}H NMR (400 MHz, D_2O) δ 6.49 - 6.40 (m, 1H), C16H36BrNO 5.63 - 5.59 (m, 1H), 3.72 - 3.69 (m, 6H), 3.24 - 3.21 (m, 6H), 1.59 - 1.57 (m, 3H) ppm; ^{13}C NMR (100 MHz, D_2O) δ 175.8, 141.3, 127.2, 55.3, 55.0, 16.9 ppm.

IR: ν 3096, 2853, 1655, 1553, 1475, 1442, 1365, 1289, 1246, 1207, 1101, 1062, 1035, 1011, 982, 913, 862, 831, 708, 578, 517 cm^{-1}.

IL-25: [TEOA][Hexanoate]：

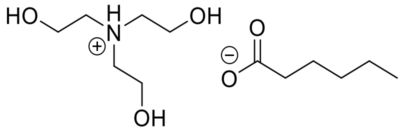

^{1}H NMR (400 MHz, D_2O) δ 3.90 - 3.88 (m, 6H), 3.42 - 3.39 (m, 6H), 2.11 (t, *J* = 7.6 Hz, 2H), 1.53 - 1.46 (m, 2H), 1.25 - 1.21 (m, 4H), 0.82 (t, *J* = 6.8 Hz, 3H) ppm; ^{13}C NMR (100 MHz, D_2O) δ 183.9, 55.2, 55.0, 37.5, 31.0, 25.5, 21.8, 13.3 ppm.

IR: ν 3267, 2930, 2871, 1730, 1562, 1400, 1248, 1174, 1097, 1067, 1036, 910, 881, 732, 649 cm^{-1}.

IL-26: [TEOA][Isovalerate]：

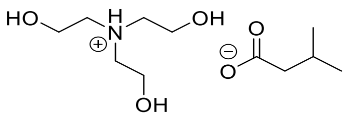

^{1}H NMR (400 MHz, D_2O) δ 3.92 - 3.89 (m, 6H), 3.38 - 3.35 (m, 6H), 2.05 - 2.03 (m, 2H), 1.99 - 1.88 (m, 1H), 0.90 (d, J = 6.6 Hz, 6H) ppm; ^{13}C NMR (100 MHz, D_2O) δ 183.4, 55.5, 55.3, 47.2, 26.1, 21.9 ppm.

IR: ν 3234, 2954, 2869, 1709, 1561, 1397, 1260, 1213, 1167, 1097, 1067, 1034, 913, 881, 709, 646, 433 cm^{-1}.

IL-27: [TEOA][Butyrate]：

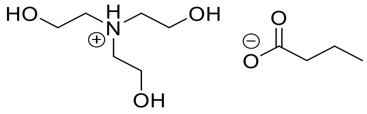

^{1}H NMR (600 MHz, D_2O) δ 3.82 - 3.80 (m, 6H), 3.33 – 3.31 (m, 6H), 2.02 (t, J = 7.4 Hz, 2H), 1.45 - 1.39 (m, 2H), 0.76 (t, J = 7.4 Hz, 3H) ppm; ^{13}C NMR (100 MHz, D_2O) δ 183.5, 55.2, 55.1, 39.5, 19.3, 13.3 ppm.

IR: ν 3227, 2960, 2872, 1721, 1561, 1399, 1307, 1256, 1206, 1066, 1034, 881. 672, 619, 521 cm^{-1}.

IL-28: [TEOA][Acetate]：

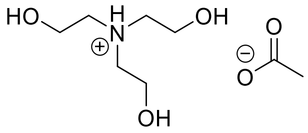

^{1}H NMR (400 MHz, D_2O) δ 3.84 - 3.81 (m, 6H), 3.22 - 3.20 (m, 6H), 1.86 (s, 3H) ppm; ^{13}C NMR (100 MHz, D_2O) δ 181.2, 56.1, 55.3, 23.3 ppm.

IR: ν 3226, 2871, 1567, 1400, 1154, 1064, 1032, 913, 881, 648, 616 cm^{-1}.

IL-29: [TEOA][Lactate]：

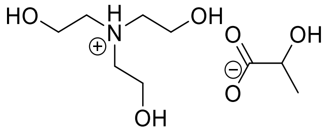

^{1}H NMR (400 MHz, D_2O) δ 4.13 - 4.07 (m, 1H), 3.96 - 3.93 (m, 6H), 3.49 - 3.46 (m, 6H), 1.31 (d, *J* = 6.8 Hz, 3H) ppm; ^{13}C NMR (100 MHz, D_2O) δ 181.6, 68.0, 55.2, 54.9, 20.0 ppm.

IR: ν 3266, 2973, 2878, 1721, 1583, 1450, 1410, 1344. 1121. 1095, 1065, 1033, 916, 850, 647, 533 cm^{-1}.

IL-30: [TEOA][Glycolate]：

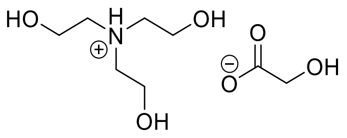

^{1}H NMR (400 MHz, D_2O) δ 3.95 - 3.93 (m, 8H), 3.48 - 3.45 (m, 6H) ppm; ^{13}C NMR (100 MHz, D_2O) δ 179.8, 61.2, 55.3, 55.1 ppm.

IR: ν 3230, 2881, 1584, 1399, 1318, 1062, 1005, 912, 686, 579, 515 cm^{-1}.

IL-31: [TEOA][3,5,5-Trimethylhexanoate]：

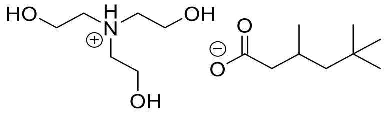

^{1}H NMR (400 MHz, D_2O) δ 3.95 - 3.93 (m, 6H), 3.46 - 3.43 (m, 6H), 2.22 - 2.17 (m, 1H), 1.98 - 1.93 (m, 2H), 1.24 - 1.20 (m, 1H), 1.12 - 1.07 (m, 1H), 0.94 - 0.84 (m, 12H) ppm; ^{13}C NMR (150 MHz, D_2O) δ 181.6, 55.5, 55.3, 50.8, 47.5, 30.6, 29.6, 27.4, 22.4 ppm.

IR: ν 3251, 2952, 2868, 1711, 1561, 1394, 1364, 1267, 1212, 1164, 1098, 1072, 1035, 912, 882, 672, 654, 452 cm^{-1}.

IL-32: [DEOA][Hexanoate]：

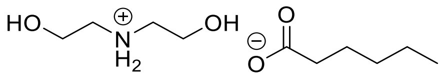

^{1}H NMR (400 MHz, D_2O) δ 3.76 - 3.73 (m, 4H), 3.12 - 3.09 (m, 4H), 2.07 - 2.03 (m, 2H), 1.47 - 1.40 (m, 2H), 1.19 - 1.17 (m, 4H), 0.77 (t, *J* = 7.0 Hz, 3H) ppm; ^{13}C NMR (100 MHz, D_2O) δ 183.9, 56.5, 48.8, 37.5, 31.0, 25.5, 21.7, 13.3 ppm.

IR: ν 2954, 2928, 2859, 1552, 1399, 1305, 1099, 1068, 962, 733, 655, 518 cm^{-1}.

IL-33: [DEOA][Butyrate]：

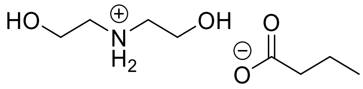

^{1}H NMR (400 MHz, D_2O) δ 3.80 - 3.77 (m, 4H), 3.16 - 3.14 (m, 4H), 2.09 - 2.05 (m, 2H), 1.52 - 1.45 (m, 2H), 0.83 - 0.79 (m, 3H) ppm; ^{13}C NMR (100 MHz, D_2O) δ 183.8, 56.4, 48.8, 39.5, 19.3, 13.2 ppm.

IR: ν 2959, 2872, 1551, 1398, 1306, 1255, 1210, 1068, 955, 894, 791, 699, 819, 513 cm^{-1}.

IL-34: [DEOA][Acetate]：

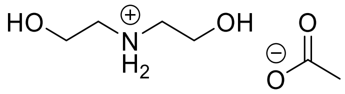

^{1}H NMR (400 MHz, D_2O) δ 3.78 - 3.76 (m, 4H), 3.12 - 3.09 (m, 4H), 1.84 (s, 3H) ppm; ^{13}C NMR (100 MHz, D_2O) δ 181.0, 56.3, 48.7, 23.2 ppm.

IR: ν 2843, 1554, 1397, 1335, 1066, 1016, 959, 923, 648, 616, 471 cm^{-1}.

IL-35: [DEOA][Glycolate]：

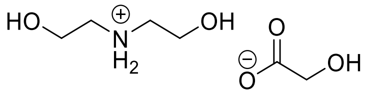

^{1}H NMR (400 MHz, D_2O) δ 3.92 (s, 2H), 3.86 - 3.84 (m, 4H), 3.24 - 3.21 (m, 4H) ppm; ^{13}C NMR (100 MHz, D_2O) δ 179.6, 61.1, 56.4, 48.8 ppm.

IR: ν 3187, 2847, 1572, 1401, 1317, 1065, 948, 912, 687, 580, 512 cm^{-1}.

IL-36: [DEOA][Lactate]：

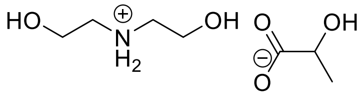

^{1}H NMR (400 MHz, D_2O) δ 4.16 - 4.11 (m, 1H), 3.89 - 3.86 (m, 4H), 3.26 - 3.24 (m, 4H), 1.34 (d, J = 6.8 Hz, 3H) ppm; ^{13}C NMR (100 MHz, D_2O) δ 181.9, 68.1, 57.3, 48.9, 20.1 ppm.

IR: ν 3226, 2971, 2871, 1735, 1573, 1449, 1408, 1122, 1069, 1035, 944, 852, 773, 656, 536 cm^{-1}.

IL-37: [DMEOA][Acetate]：

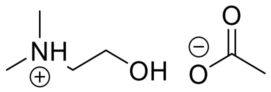

^{1}H NMR (400 MHz, D_2O) δ 3.89 - 3.86 (m, 2H), 3.28 - 3.26 (m, 2H), 2.90 (s, 6H), 1.98 (s, 3H) ppm; ^{13}C NMR (100 MHz, D_2O) δ 178.9, 58.6, 55.0, 42.6, 21.8 ppm.

IR: ν 3268, 3028, 1572, 1474, 1397, 1258, 1080, 1013, 956, 919, 858, 799, 649, 613, 452 cm^{-1}.

IL-38: [DMEOA][Lactate]：

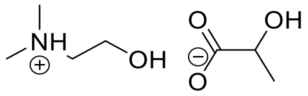

^{1}H NMR (400 MHz, D_2O) δ 4.17 - 4.12 (m, 1H), 3.87 - 3.85 (m, 2H), 3.27 - 3.24 (m, 2H), 2.89 (s, 6H), 1.31 (d, J = 7.0 Hz, 3H) ppm; ^{13}C NMR (100 MHz, D_2O) δ 181.4, 67.9, 58.7, 55.1, 42.6, 19.9 ppm.

IR: ν 3275, 2973, 2872, 2349, 1721, 1587, 1458, 1343, 1232, 1121, 1081, 1034, 992, 922, 849, 822, 773, 648, 436 cm^{-1}.

IL-39: [MEOA][Butyrate]：

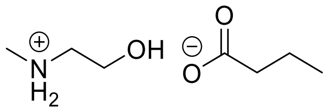

^{1}H NMR (400 MHz, D_2O) δ 3.82 - 3.79 (m, 2H), 3.15 - 3.13 (m, 2H), 2.71 (s, 3H), 2.12 (t, J = 7.2 Hz, 2H), 1.56 - 1.47 (m, 2H), 0.85 (t, J = 7.4 Hz, 3H) ppm; ^{13}C NMR (100 MHz, D_2O) δ 183.5, 56.3, 50.3, 39.4, 32.4, 19.2, 13.2 ppm.

IR: ν 2960, 2872, 2439, 1553, 1458, 1394, 1337, 1305, 1254, 1210, 1150, 1085, 1040, 1003, 945, 893, 791, 700, 620, 532 cm^{-1}.

IL-40: [MEOA][Acetate]：

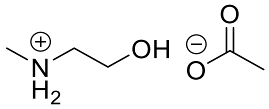

^{1}H NMR (400 MHz, D_2O) δ 3.58 - 3.55 (m, 2H), 2.91 - 2.89 (m, 2H), 2.48 (s, 3H), 1.66 (s, 3H) ppm; ^{13}C NMR (100 MHz, D_2O) δ 180.8, 56.2, 50.3, 32.4, 23.1 ppm.

IR: ν 2731, 2441, 1556, 1392, 1334, 1149, 1082, 1039, 1012, 918, 648, 616, 533, 470 cm^{-1}.

IL-41: [TMG][Isovalerate]：

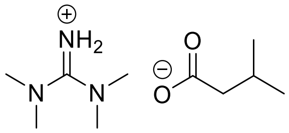

^{1}H NMR (600 MHz, D_2O) δ 2.96 (s, 12H), 2.07 (d, *J* = 7.4 Hz, 2H), 2.00 - 1.93 (m, 1H), 0.92 (d, *J* = 6.6 Hz, 6H) ppm; ^{13}C NMR (100 MHz, D_2O) δ 182.7, 161.3, 46.9, 38.8, 26.1, 21.9 ppm.

IR: ν 2951, 1547, 1453, 1386, 1322, 1257, 1168, 1098, 1064, 1035, 883, 726, 639, 543, 477 cm^{-1}.

IL-42: [TMG][TFA]：

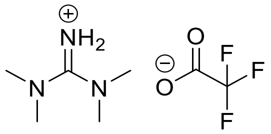

^{1}H NMR (400 MHz, D_2O) δ 2.96 (s, 12H) ppm; ^{13}C NMR (100 MHz, D_2O) δ 162.5 (q), 161.3, 116.5 (q), 38.8 ppm. ^{19}F NMR (376 MHz, D_2O) δ -75.7.

IR: ν 3097, 1669, 1600, 1567, 1410, 1197, 1169, 1180, 1063, 1037, 879, 827,799, 717 cm^{-1}.

IL-43: [TMG][DCA]：

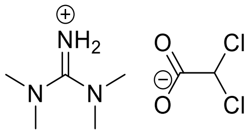

^{1}H NMR (600 MHz, D_2O) δ 6.11 (s, 1H), 2.96 (s, 12H) ppm; ^{13}C NMR (100 MHz, D_2O) δ 170.3, 161.3, 68.7, 39.0 ppm.

IR: ν 3013, 1643, 1596, 1564, 1453, 1408, 1347, 1212, 1145, 1097, 1063, 1036, 909, 881 cm^{-1}.

IL-44: [TMG][Glycolate]：

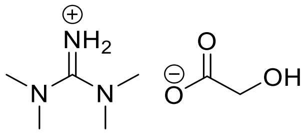

^{1}H NMR (600 MHz, D_2O) δ 3.94 (s, 2H), 2.97 (s, 12H) ppm; ^{13}C NMR (100 MHz, D_2O) δ 178.4, 161.3, 60.6, 38.8 ppm.

IR: ν 3219, 3006, 2929, 2879, 1684, 1600, 1561, 1482, 1454, 1428, 1410, 1390, 1311, 1235 cm^{-1}.

IL-45: [TMG][Crotonate]：

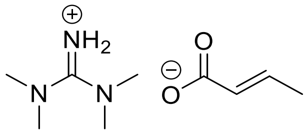

^{1}H NMR (600 MHz, D_2O) δ 6.69 - 6.63 (m, 1H), 5.84 (d, J = 15.6 Hz, 1H), 2.96 (s, 12H), 1.82 (d, J = 6.8 Hz, 3H) ppm; ^{13}C NMR (100 MHz, D_2O) δ 175.6, 161.3, 141.1, 127.4, 38.8, 17.0 ppm.

IR: ν 3014, 2821, 1655, 1593, 1536, 1481, 1438, 1409, 1360, 1291, 1245, 1213, 1156, 1107 cm^{-1}.

IL-46: [TMG][Lactate]：

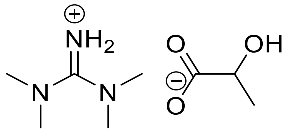

^{1}H NMR (600 MHz, D_2O) δ 4.14 - 4.10 (m, 1H), 2.97 (s, 12H), 1.34 (d, J = 6.8 Hz, 3H) ppm; ^{13}C NMR (100 MHz, D_2O) δ 181.9, 161.3, 68.4, 38.8, 20.2 ppm.

IR: ν 2967, 1595, 1563, 1451, 1407, 1372, 1342, 1238, 1115, 1091, 1064, 1033, 880 cm^{-1}.

IL-47: [TMG] [Valerate]：

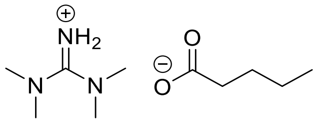

^{1}H NMR (600 MHz, D_2O) δ 2.97 (s, 12H), 2.22 (t, J = 7.4 Hz, 2H), 1.56 - 1.53 (m, 2H), 1.32 - 1.31 (m, 2H), 0.90 (t, J = 7.4 Hz, 3H) ppm; ^{13}C NMR (150 MHz, D_2O) δ 183.6, 161.3, 38.9, 37.1, 27.9, 22.0, 13.2 ppm.

IR: ν 2954, 1547, 1454, 1389, 1237, 1096, 1065, 1035, 883, 727, 652, 542 cm^{-1}.

IL-48: [BMIM][Cl]:

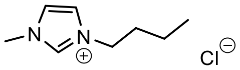

^{1}H NMR (400 MHz, D_2O) δ 8.78 (s, 1H), 7.52 (d, J = 21.0 Hz, 2H), 4.25 (t, J = 7.2 Hz, 2H), 3.95 (s, 3H), 1.94 - 1.86 (m, 2H), 1.39 - 1.32 (m, 2H), 0.96 (t, J = 7.4 Hz, 3H). ^{13}C NMR (150 MHz, D_2O) δ 139.2, 125.9, 124.1, 50.2, 36.1, 33.5, 19.5, 13.3. [2]

IL-49: [BMIM][Br]:

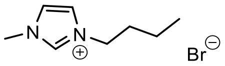

^{1}H NMR (600 MHz, D_2O) δ 8.65 (s, 1H), 7.41 (s, 1H), 7.36 (s, 1H), 4.13 (t, J = 7.2 Hz, 2H), 3.82 (s, 3H), 1.81 - 1.76 (m, 2H), 1.27 - 1.23 (m, 2H), 0.86 (t, J = 7.4 Hz, 3H). ^{13}C NMR (150 MHz, D_2O) δ 136.5, 124.1, 122.9, 49.7, 36.1, 31.7, 17.9, 13.1. [3]

IL-50: [BMIM][BF_4] [2]:

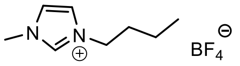

^{1}H NMR (400 MHz, D_2O) δ 8.72 (s, 1H), 7.52 - 7.48 (m, 2H), 4.24 (t, J = 7.2 Hz, 2H), 3.95 (s, 3H), 1.90 (dd, J_1 = 14.8 Hz, J_2 = 7.2 Hz, 2H), 1.38 (dd, J_1 = 15.0 Hz, J_2 = 7.5 Hz, 2H), 0.98 (t, J = 7.4 Hz, 3H). ^{13}C NMR (150 MHz, D_2O) δ135.3, 123.2, 122.5, 50.3,

35.7, 31.9, 18.1, 12.7. ^{19}F NMR (376 MHz, D_2O) δ -150.3. ^{11}B NMR (193 MHz, D_2O) δ -1.4.

IL-51: [HMIM][BF_4]:

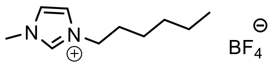

^1H NMR (400 MHz, D_2O) δ 8.71 - 8.66 (m, 1H), 7.48 (d, *J* = 16.6 Hz, 2H), 4.24 - 4.18 (m, 2H), 3.93 (s, 2H), 3.89 (s, 1H), 1.91 - 1.88 (m, 2H), 1.33 - 1.22 (m, 6H), 0.90 - 0.87 (m, 2H), 0.79 - 0.78 (m, 1H). ^{13}C NMR (150 MHz, D_2O) δ 138.7, 126.1, 120.5, 50.1, 36.6, 30.7, 29.1, 23.5, 21.1, 13.0. ^{19}F NMR (376 MHz, D_2O) δ -150.2. ^{11}B NMR (193 MHz, D_2O) δ -1.3. [4]

IL-52: [OMIM][Br]:

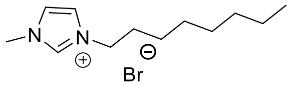

^1H NMR (400 MHz, D_2O) δ 9.05 (s, 1H), 7.66 (d, *J* = 11.8 Hz, 2H), 4.35 (d, *J* = 14.4 Hz, 2H), 4.03 (s, 3H), 1.95 (m, 2H), 1.38 - 1.30 (m, 10H), 0.89 - 0.87 (m, 3H).[5]
^{13}C NMR (150 MHz, D_2O) δ 136.3, 125.1, 121.9, 49.2, 35.6, 31.3, 29.4, 28.5, 28.3, 24.9, 22.7, 13.8.

IL-53: [DodecylMIM][Br]:

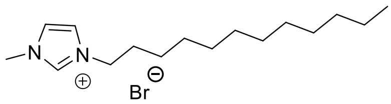

^1H NMR (400 MHz, D_2O) δ 8.99 (s, 1H), 7.59 - 7.55 (m, 2H), 4.29 - 4.26 (m, 2H), 3.96 (s, 3H), 1.91 - 1.89 (m, 2H), 1.34 - 1.25 (m, 18H), 0.84 (t, *J* = 6.6 Hz, 3H).[5]
^{13}C NMR (150 MHz, D_2O) δ 137.5, 124.1, 122.3, 50.7, 37.5, 32.1, 31.4, 30.4, 30.1, 29.9, 29.3, 28.3, 27.7, 25.8, 23.5, 13.9.

IL-54: [TetradecylMIM][Br]:

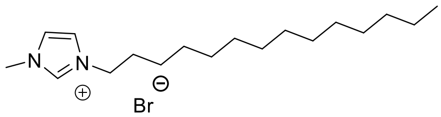

^{1}H NMR (400 MHz, D_2O) δ 9.00 (s, 1H), 7.59 (s, 1H), 7.55 (s, 1H), 4.29 - 4.26 (m, 2H), 3.96 (s, 3H), 1.91 - 1.90 (m, 2H), 1.35 - 1.25 (m, 20H), 0.87 - 0.83 (m, 3H).[6]

^{13}C NMR (150 MHz, D_2O) δ 136.8, 123.1, 121.7, 49,7, 37.3, 32.1, 30.5, 29.7, 29.6, 29.4, 29.1, 29.0, 28.9, 28.7, 28.5, 25.5, 22.9, 14.1.

Characterization of synthesized ADP-ribosyl azide compounds

α-ADPr-N_3 (1):

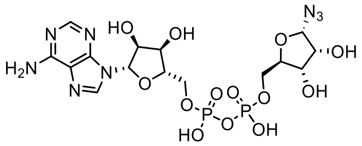

^{1}H NMR (600 MHz, D_2O) δ 8.64 (s, 1H), 8.44 (s, 1H), 6.18 (d, J = 5.4 Hz, 1H), 5.46 (d, J = 4.8 Hz, 1H), 4.76 (d, J = 5.2 Hz, 1H), 4.55 - 4.54 (m, 1H), 4.41 (s, 1H), 4.30 - 4.22 (m, 5H), 4.09 - 4.05 (m, 2H). ^{31}P NMR (162 MHz, D_2O) δ -11.31 (s)，-11.40 (s). MALDI-TOF-MS: *m/z* $C_{15}H_{22}N_8O_{13}P_2$ $[M+H]^+$ 585.34 , found 585.35.

β-ADPr-N_3 (1'):

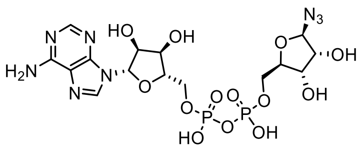

^{1}H NMR (600 MHz, D_2O) δ 8.66 (s, 1H), 8.45 (s, 1H), 6.19 (d, J = 5.4 Hz, 1H), 5.32 (d, J = 2.2 Hz, 1H), 4.57 - 4.55 (m, 1H), 4.42 (s, 1H), 4.32 - 4.27 (m, 3H), 4.17 - 4.16 (m, 2H), 4.08 - 4.03 (m, 2H), 3.99 - 3.98 (m, 1H). ^{31}P NMR (162 MHz, D_2O) δ -11.38 (s). MALDI-TOF-MS: *m/z* $C_{15}H_{22}N_8O_{13}P_2$ $[M+H]^+$ 585.34 , found 585.20.

To further identify the configuration of α/β-ADPr-N_3, we run the H-H COSY, 1D-TOCSY and 1D-NOESY spectra below (Figure S11 to Figure S14). Figure S12 is 1D-TOCSY and 1D-NOESY spectra of α-ADPr-N_3. The 1D-NOESY data of α-ADPr-N_3 established the cis relationship between H-1 and H-3 on the distal ribose (the peak was marked with *red asterisk* in Figure 12(D)) beside of the relationship between H-1 and

H-2 (the peak was marked with *green asterisk* in Figure 12(D)), which suggested the same side of H-3 and H-1 in this molecule. While in the Figure S14 which is the 1D-TOCSY and 1D-NOESY spectra of β-ADPr-N_3, only the relationship between H-1 and H-2 could be detected (the peak was marked with *purple asterisk* in Figure 14(C)), suggesting the different side of H-3 and H-1 in this molecule. The configuration of the β-ADPr-N_3 could also be confirmed by comparison its ^{1}H NMR spectrum with reported ^{1}H NMR spectrum of β-ADPr-N_3.[14]

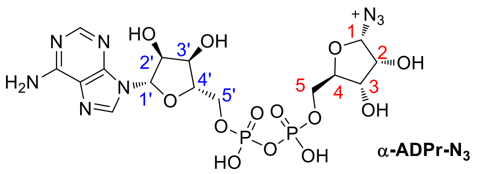

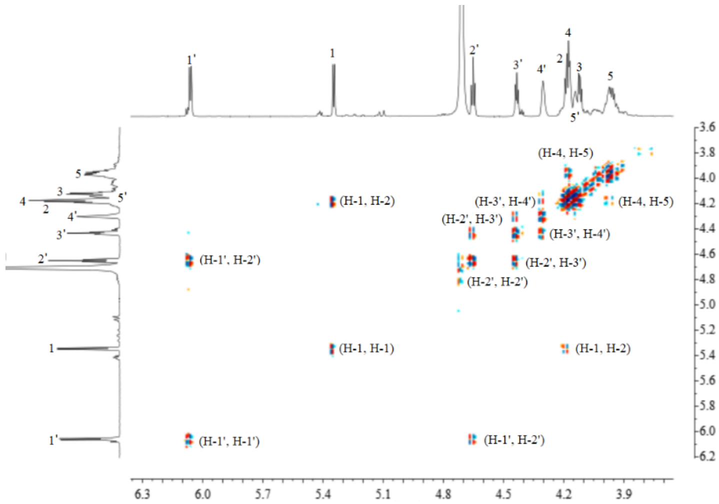

Supplementary Figure 13 H-H COSY spectrum of α-ADPr-N_3

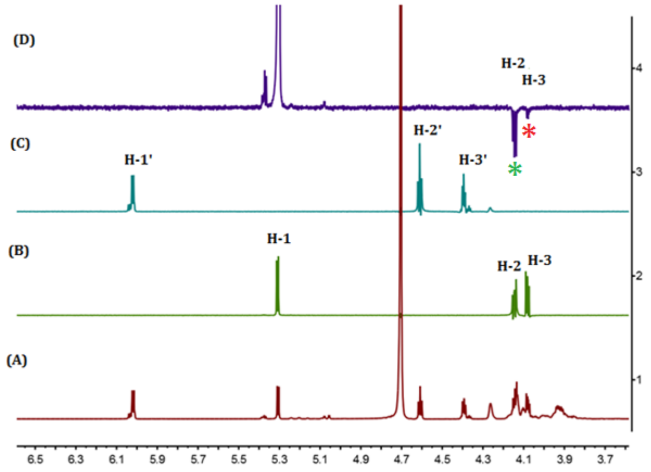

Supplementary Figure 14 Hydrogen spectrum of α-ADPr-N_3, 1D-TOCSY and 1D-

NOESY spectra. Note: (A) is ^{1}H NMR; (B) is the 1D-TOCSY spectrum with selective excitation of the H-1, (C) is the 1D-TOCSY spectrum with selective excitation of the H-1’; (D) is 1D-NOESY spectrum with selective excitation of the H-1.

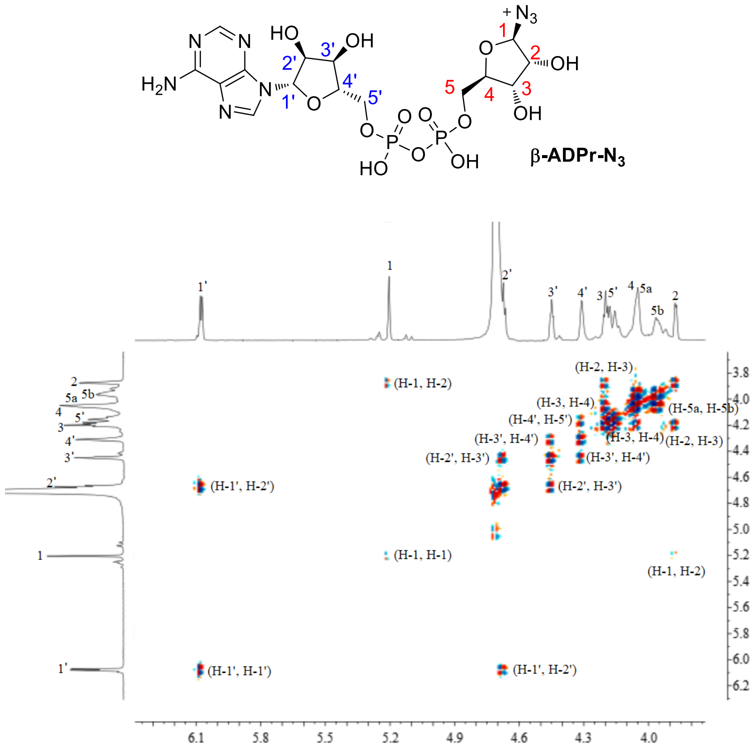

Supplementary Figure 15 H-H COSY spectrum of β-ADPr-N$_3$

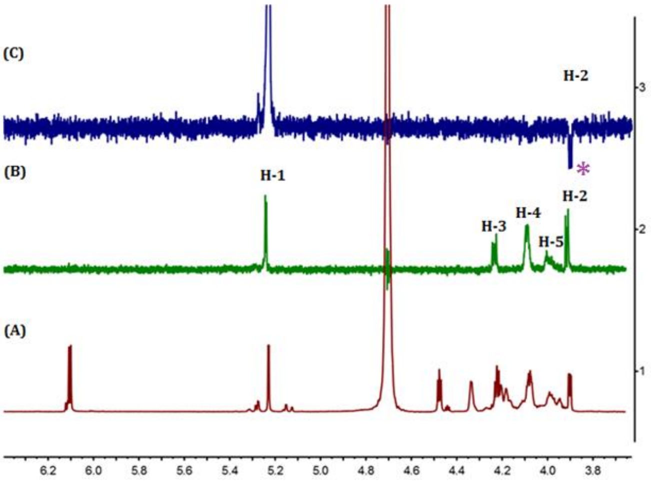

Supplementary Figure 16 Hydrogen spectrum of β-ADPr-N$_3$, 1D-TOCSY and 1D-NOESY spectra. Note: (A) is ^{1}H NMR; (B) is the 1D-TOCSY spectrum with selective excitation of the H-1; (C) is 1D-NOESY spectrum with selective excitation of the H-1.

α-ADPPr-N_3 (**2**):

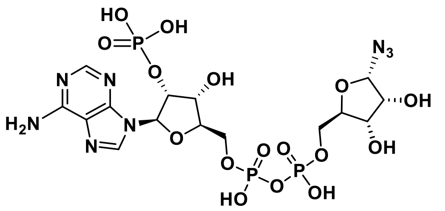

^{1}H NMR (400 MHz, D_2O) δ 8.51 (s, 1H), 8.27 (s, 1H), 6.22 (d, J = 4.8 Hz, 1H), 5.39 (d, J = 4.8 Hz, 1H), 5.00 - 4.96 (m, 1H), 4.61 (t, J = 5.0 Hz, 1H), 4.38 (s, 1H), 4.26 - 4.23 (m, 1H), 4.19 (d, J = 5.6 Hz, 2H), 3.98 - 3.96 (m, 2H). ^{31}P NMR (162 MHz, D_2O) δ 3.53 (d, J = 6.0 Hz), -11.41 (d, J = 19.6 Hz). MALDI-TOF-MS: *m/z* $C_{15}H_{23}N_8O_{16}P_3$ $[M-H]^-$ 663.04 , Found 663.04. IR: -N_3: 2110.56cm^{-1}.

β-ADPPr-N_3 (**2'**) :

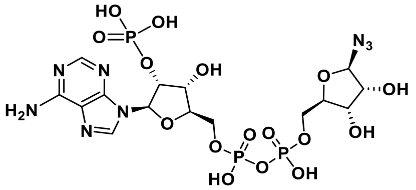

^{1}H NMR (600 MHz, D_2O) δ 8.51 (s, 1H), 8.28 (s, 1H), 6.27 (s, 1H), 5.28 (s, 1H),5.06 (d, J = 3.2 Hz, 1H), 4.65 (s, 1H), 4.41 (s, 1H), 4.29 - 4.22 (m, 3H), 4.13 - 4.08 (m, 2H), 4.00 - 3.99 (m, 1H), 3.95 (s, 1H). ^{31}P NMR (243 MHz, D_2O) δ 1.68 (s), -11.38 (s). MALDI-TOF-MS: *m/z* $C_{15}H_{23}N_8O_{16}P_3$ $[M-H]^-$ 663.03 , Found 663.04. IR: -N_3: 2110.06cm^{-1}.

8-Br-β-NAD^+:

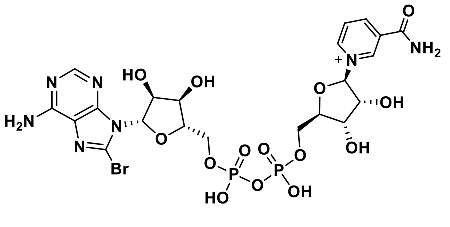

^{1}H NMR (400 MHz, D_2O) δ 9.31 (s, 1H), 9.14 (d, J = 6.2 Hz, 1H), 8.85 (d, J = 8.0 Hz, 1H), 8.24 - 8.21 (m, 1H), 8.17 (s, 1H), 6.00 (t, J = 5.0 Hz, 2H), 5.25 (t, J = 5.9 Hz, 1H), 4.63 - 4.60 (m, 1H), 4.43 - 4.41 (m, 2H), 4.33 - 4.18 (m, 6H). ^{31}P NMR (162 MHz, D_2O)

δ -11.48 (s). MALDI-TOF-MS: *m/z* $C_{21}H_{27}N_7O_{14}P_2Br$ $[M-H]^-$ 742.03 , Found 742.04.

α-8-Br-ADPr-N3 (**3**):

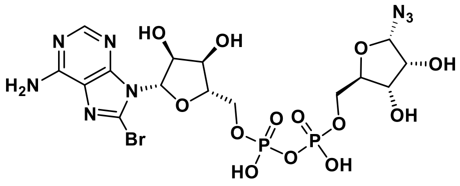

^{1}H NMR (400 MHz, D_2O) δ 8.03 (s, 1H), 6.04 (d, *J* = 7.7 Hz, 1H), 5.43 (d, *J* = 4.7 Hz, 1H), 4.74 - 4.72 (m, 1H), 4.48 - 4.46 (m, 1H), 4.34 - 4.33 (m, 1H), 4.28 - 4.24 (m, 3H), 4.23 - 4.19 (m, 2H), 4.04 (dd, J_1 = 9.2 Hz, J_2 = 4.9 Hz, 2H).^{31}P NMR (162 MHz, D_2O) δ -11.31 (s), -11.81 (s). MALDI-TOF-MS: *m/z* $C_{15}H_{21}N_8O_{13}P_2Br$ $[M+2Na-H]^+$ 706.96, Found 706.87. IR: $-N_3$: 2110.22 cm^{-1}.

β-8-Br-ADPr-N3 (**3'**):

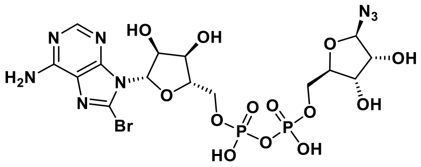

^{1}H NMR (600 MHz, D_2O) δ 8.21 (s, 1H),6.01 (d, *J* = 6.0 Hz, 1H), 5.30 (d, *J* = 2.3 Hz, 1H), 5.10 (t, *J* = 5.9 Hz, 1H), 4.59 - 4.58 (m, 1H), 4.30 - 4.26 (m, 4H), 4.22 - 4.20 (m, 1H), 4.14 (d, *J* = 5.0 Hz, 1H), 4.04 - 4.02 (m, 1H), 3.97 - 3.96 (m, 1H). ^{31}P NMR (162 MHz, D_2O) δ -11.35 (s), -11.86 (s).IR: $-N_3$: 2114.70 cm^{-1}. MALDI-TOF-MS: *m/z* $C_{15}H_{21}N_8O_{13}P_2Br$ $[M+2Na-H]^+$ 706.96, Found 706.82. IR: $-N_3$: 2110.51 cm^{-1}.

Characterization of Synthesized ADP-ribosylated peptide Compounds

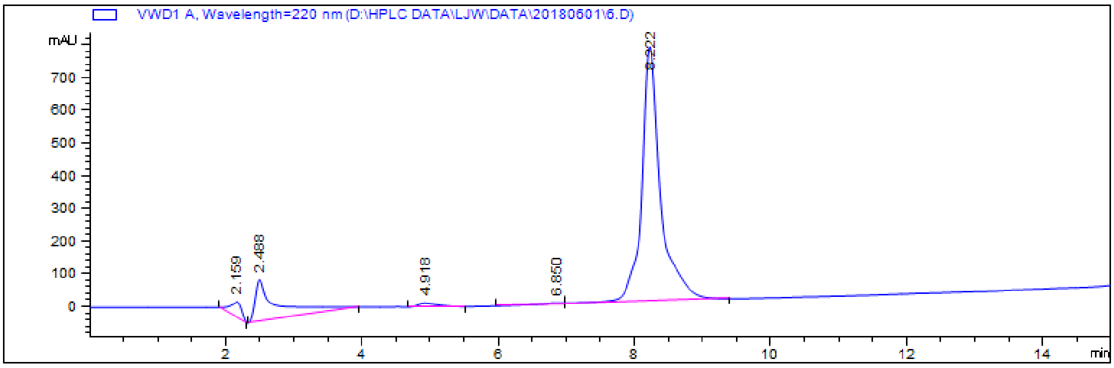

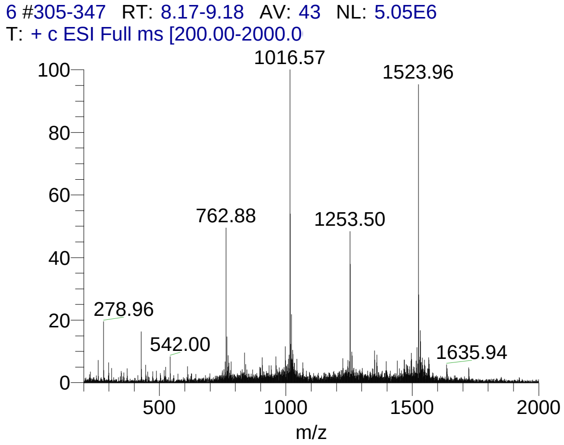

Peptide-1: LC-MS: *m/z* $C_{131}H_{206}N_{38}O_{40}S_{1}P_{2}$ $[M+2H]^{2+}$ 1523.73 , found 1523.96

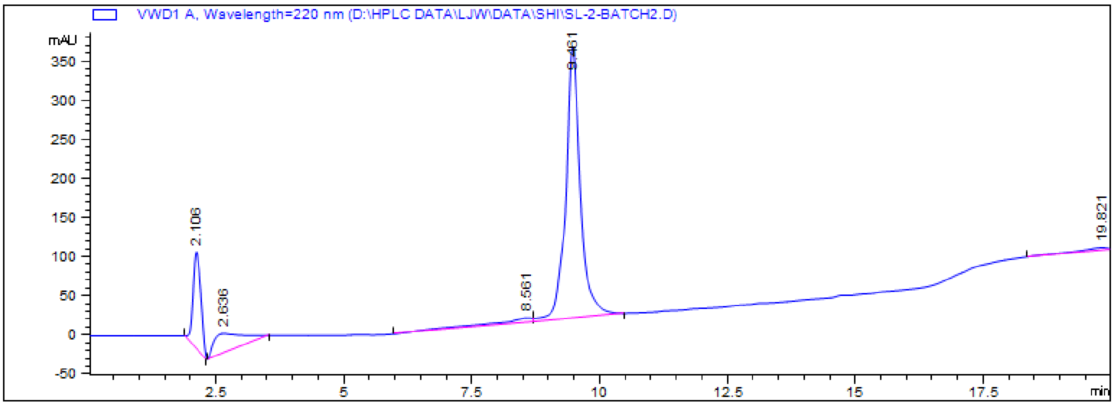

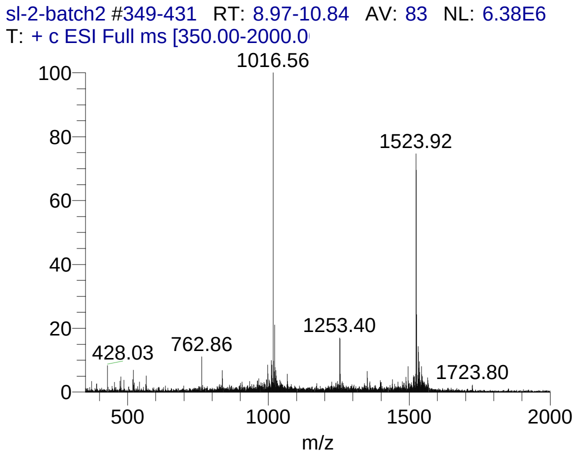

Peptide-2: LC-MS: *m/z* $C_{131}H_{206}N_{38}O_{40}S_1P_2$ $[M+2H]^{2+}$ 1523.73 , found 1523.92

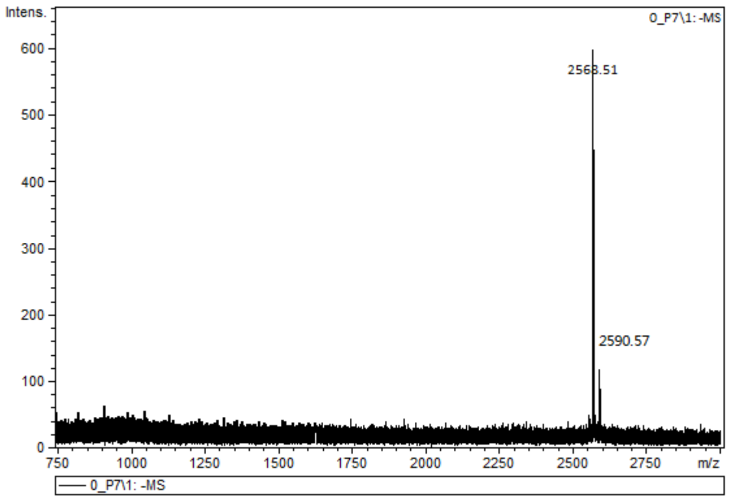

Peptide-3: MALDI-TOF-MS: *m/z* $C_{105}H_{179}N_{35}O_{36}P_2$ $[M-H]^-$ 2568.27, found 2568.51

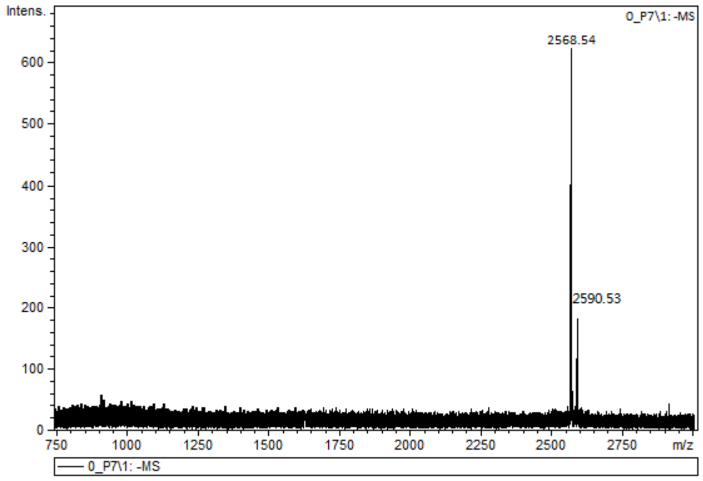

Peptide-4: MALDI-TOF-MS: *m/z* $C_{105}H_{178}N_{35}O_{36}P_2$ $[M-H]^-$ 2568.27, found 2568.54

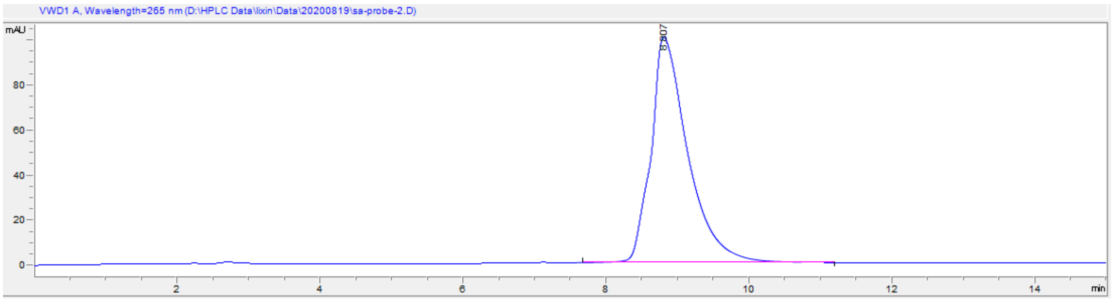

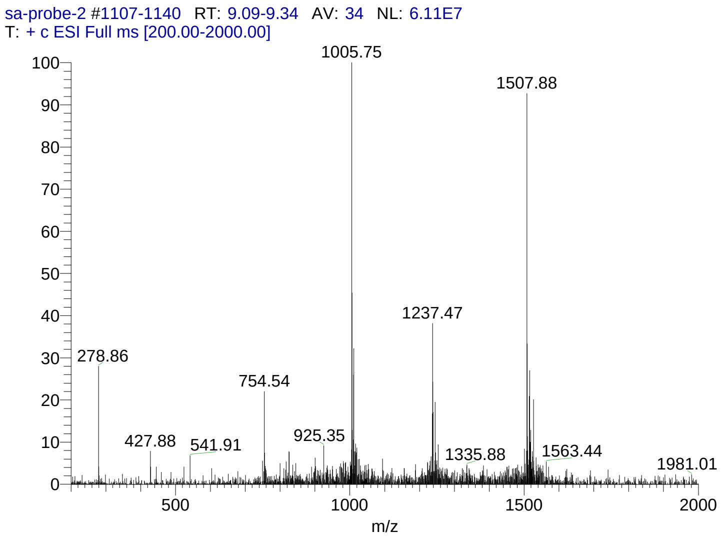

Peptide-5: LC-MS: *m/z* $C_{131}H_{206}N_{38}O_{38}S_{1}P_{2}$ $[M+2H]^{2+}$ 1508.24 , found 1507.88

^{1}H NMR and ^{31}P NMR spectra of ADP-ribosyl azide compounds

α-ADPr-N_3 (1)

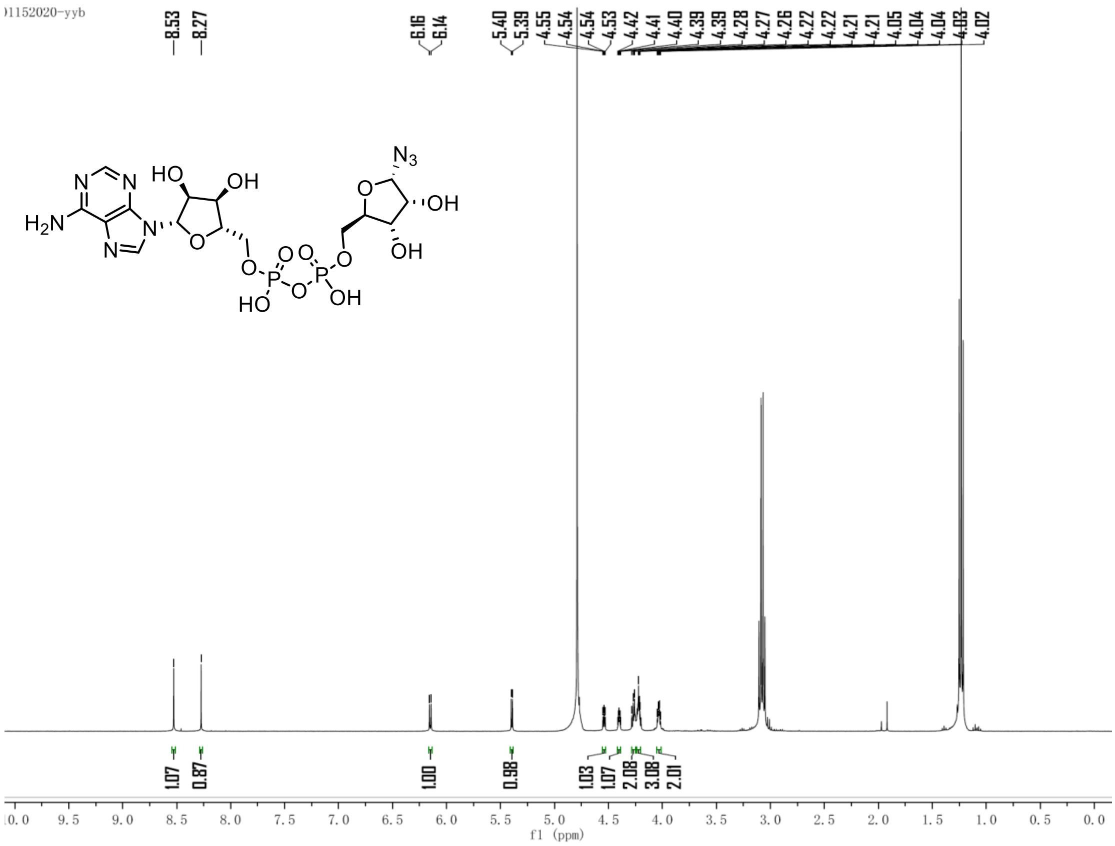

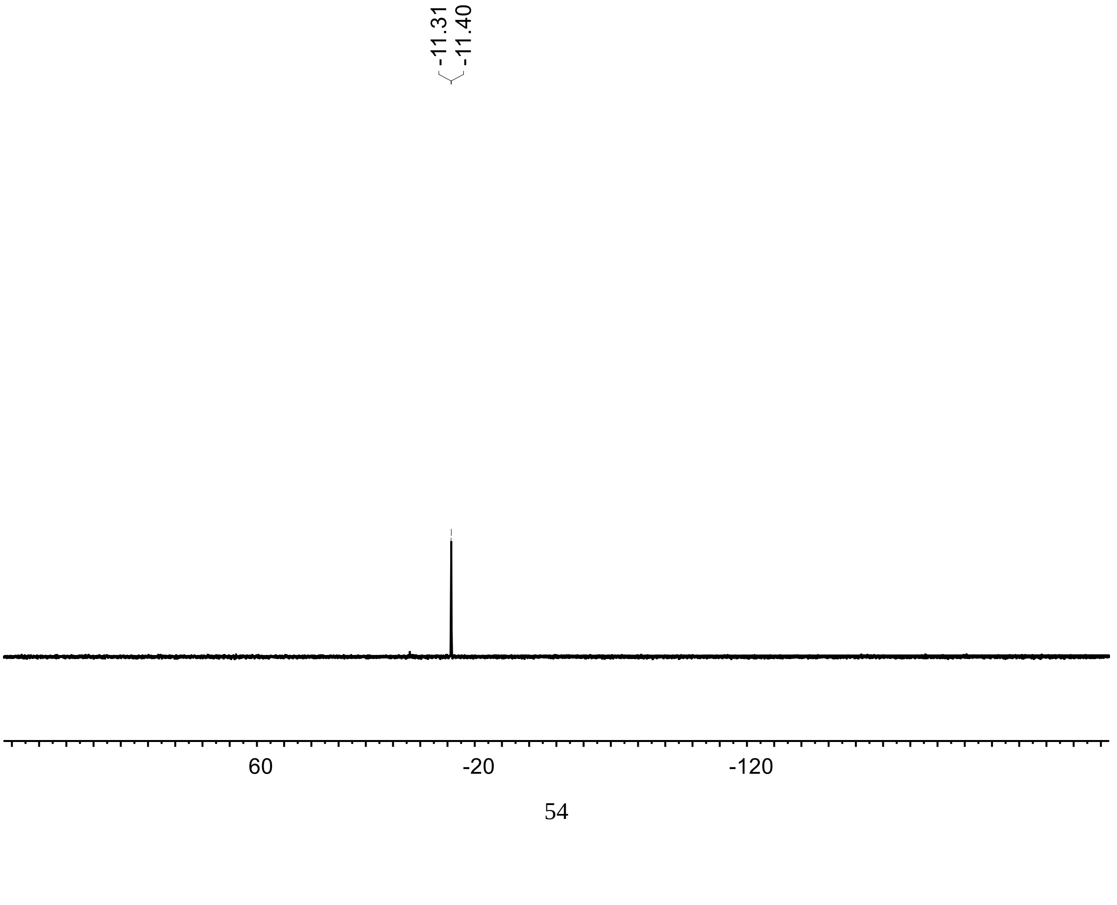

β-ADPr-N_3 (**1'**):

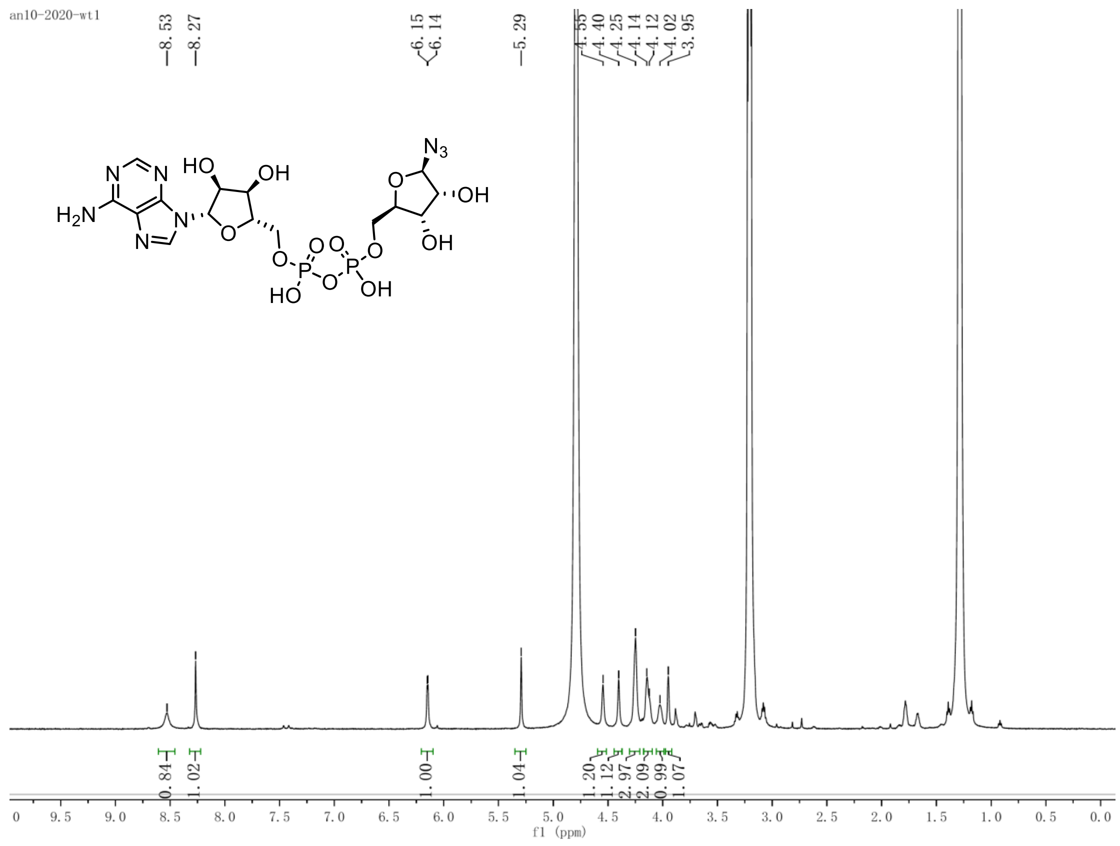

an10-2020-wt1
8.53
8.27
6.15
6.14
5.29
4.55
4.40
4.25
4.14
4.12
4.02
3.95
0.84
1.02
1.00
1.04
1.20
1.12
2.97
2.09
0.99
1.07
f1 (ppm)

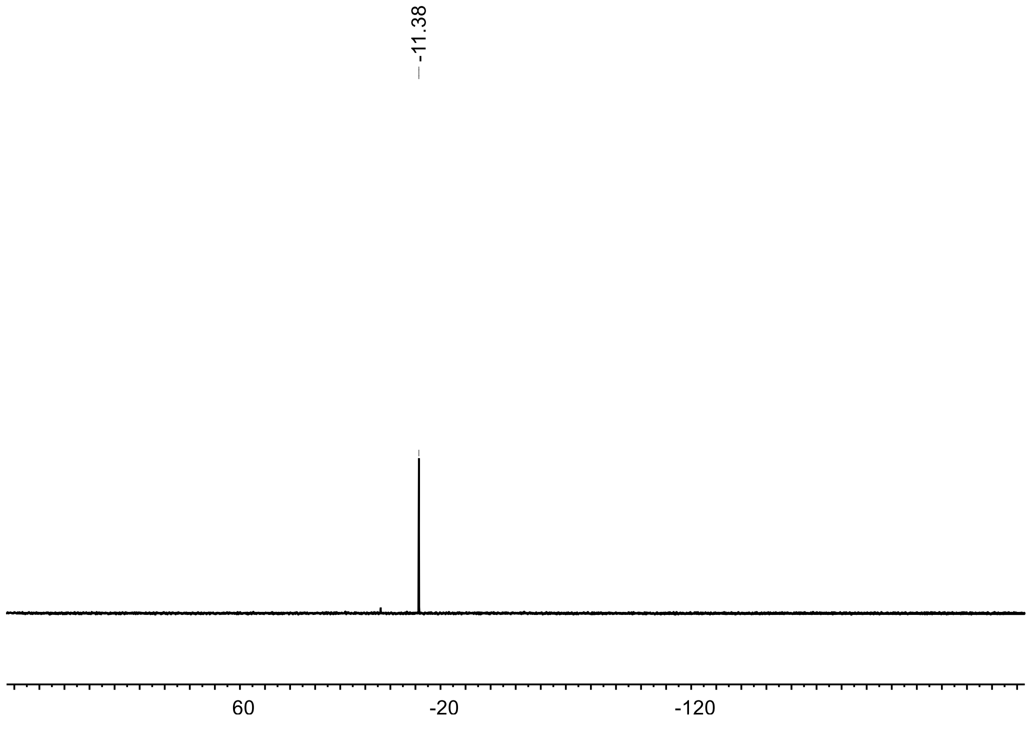

-11.38
60
-20
-120

α-ADPPr-N_3 (**2**):

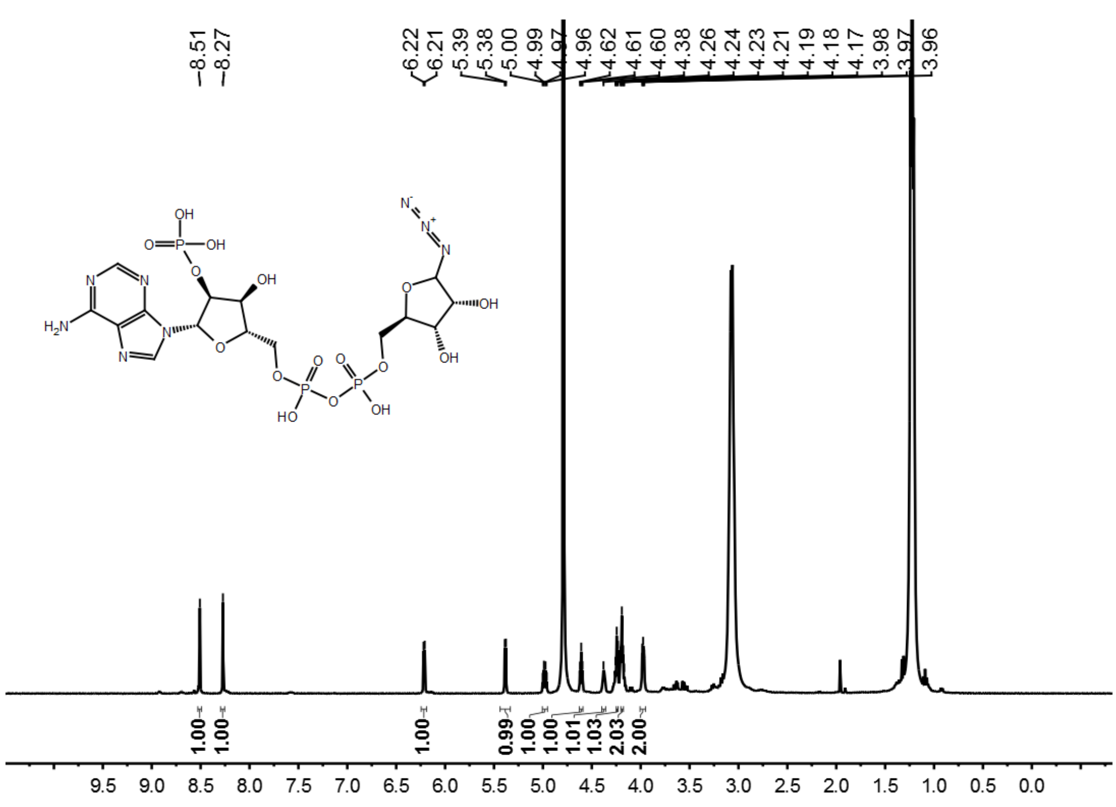

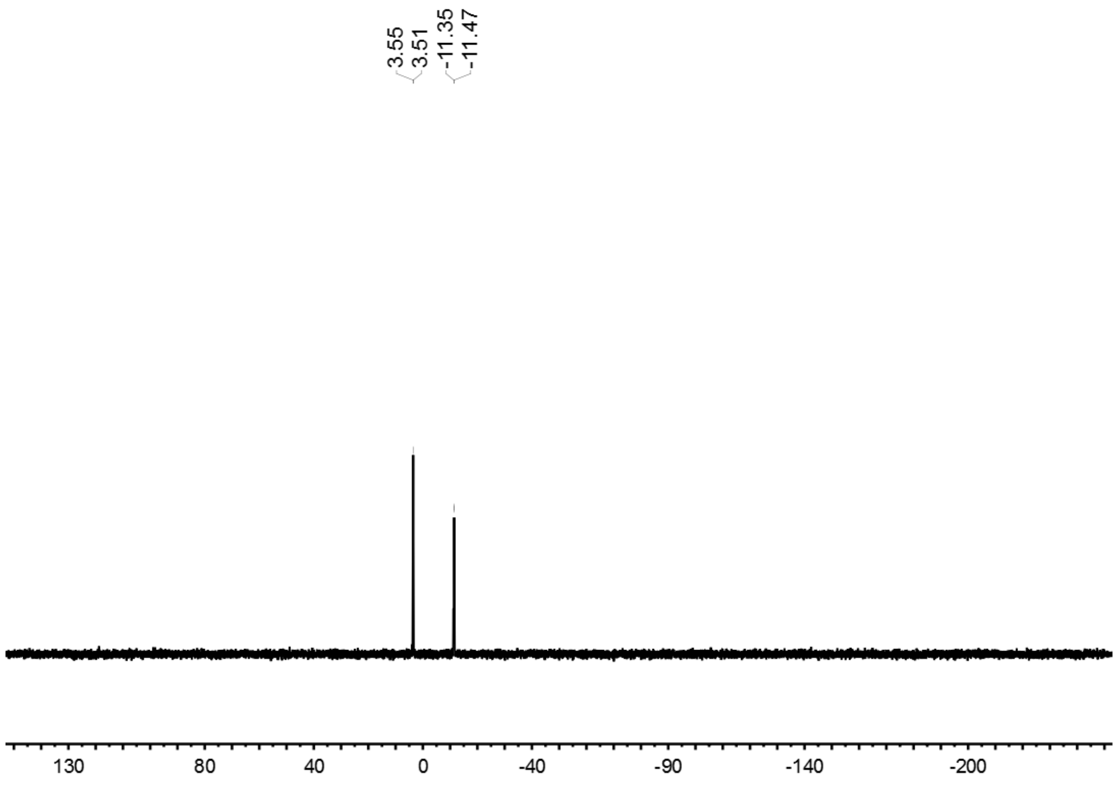

β-ADPPr-N_3 (**2’**):

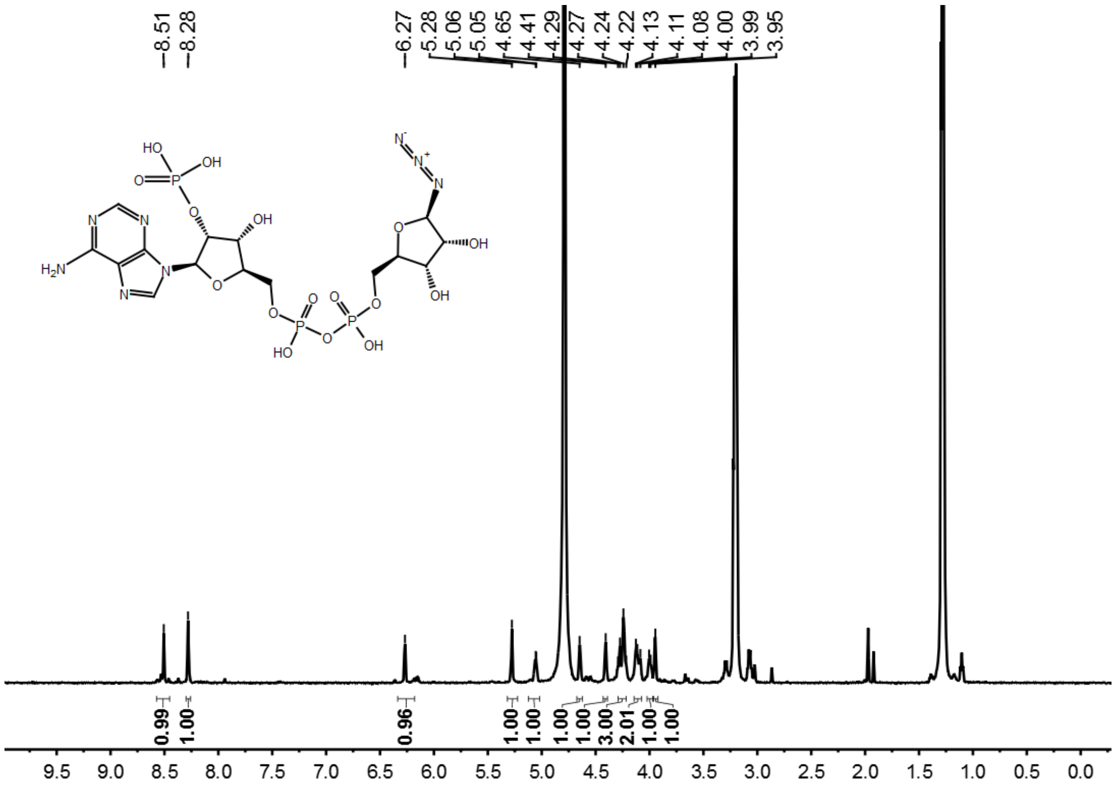
8.51
8.28
6.27
5.28
5.06
5.05
4.65
4.41
4.29
4.27
4.24
4.22
4.13
4.11
4.08
4.00
3.99
3.95
0.99
1.00
0.96
1.00
1.00
1.00
1.00
3.00
2.01
1.00
1.00
9.5 9.0 8.5 8.0 7.5 7.0 6.5 6.0 5.5 5.0 4.5 4.0 3.5 3.0 2.5 2.0 1.5 1.0 0.5 0.0

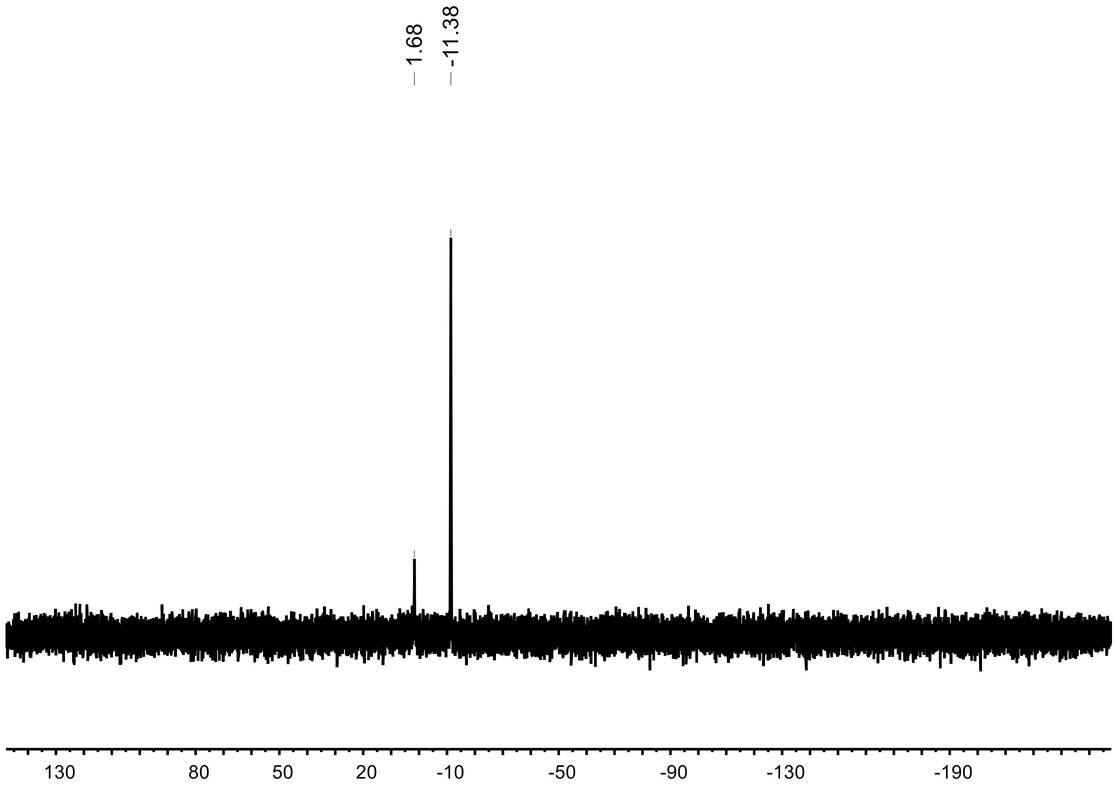
1.68
-11.38
130 80 50 20 -10 -50 -90 -130 -190

8-Br-β-NAD^+

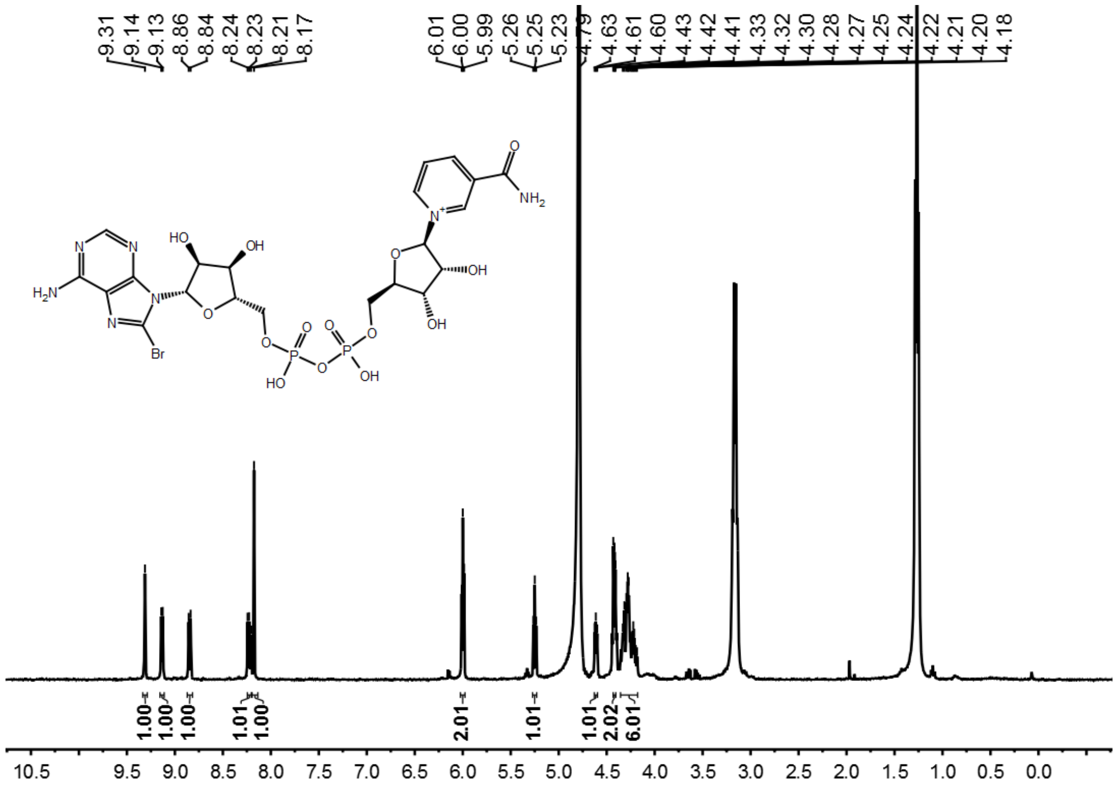
9.31
9.14
9.13
8.86
8.84
8.24
8.23
8.21
8.17
6.01
6.00
5.99
5.26
5.25
5.23
4.79
4.63
4.61
4.60
4.43
4.42
4.41
4.33
4.32
4.30
4.28
4.27
4.25
4.24
4.22
4.21
4.20
4.18
1.00
1.00
1.00
1.01
1.00
2.01
1.01
1.01
2.02
6.01
10.5 9.5 9.0 8.5 8.0 7.5 7.0 6.5 6.0 5.5 5.0 4.5 4.0 3.5 3.0 2.5 2.0 1.5 1.0 0.5 0.0

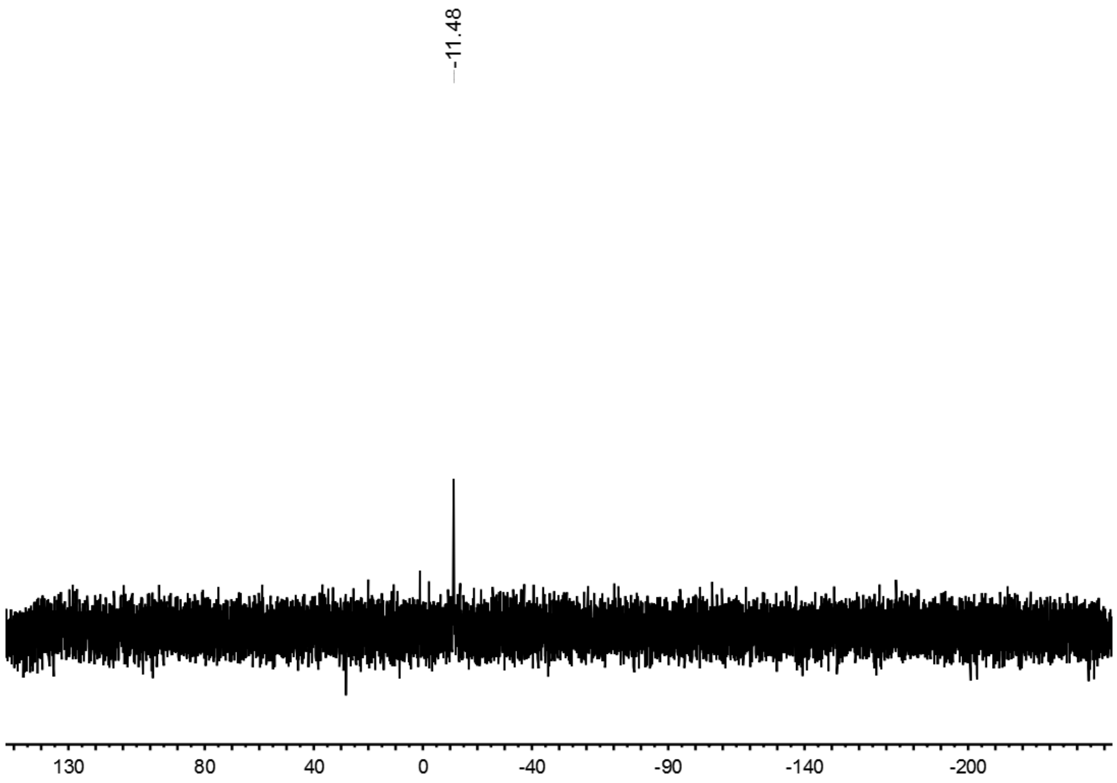
-11.48
130 80 40 0 -40 -90 -140 -200

α-8-Br-ADPr-N_3 (**3**):

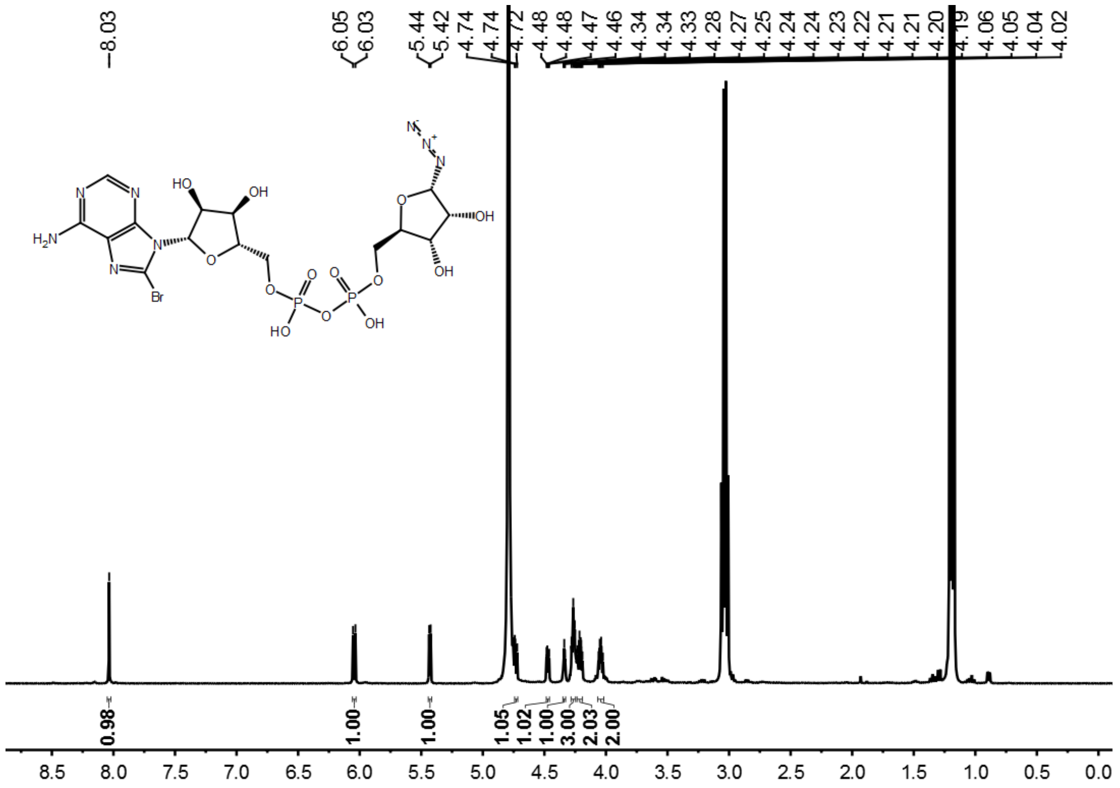

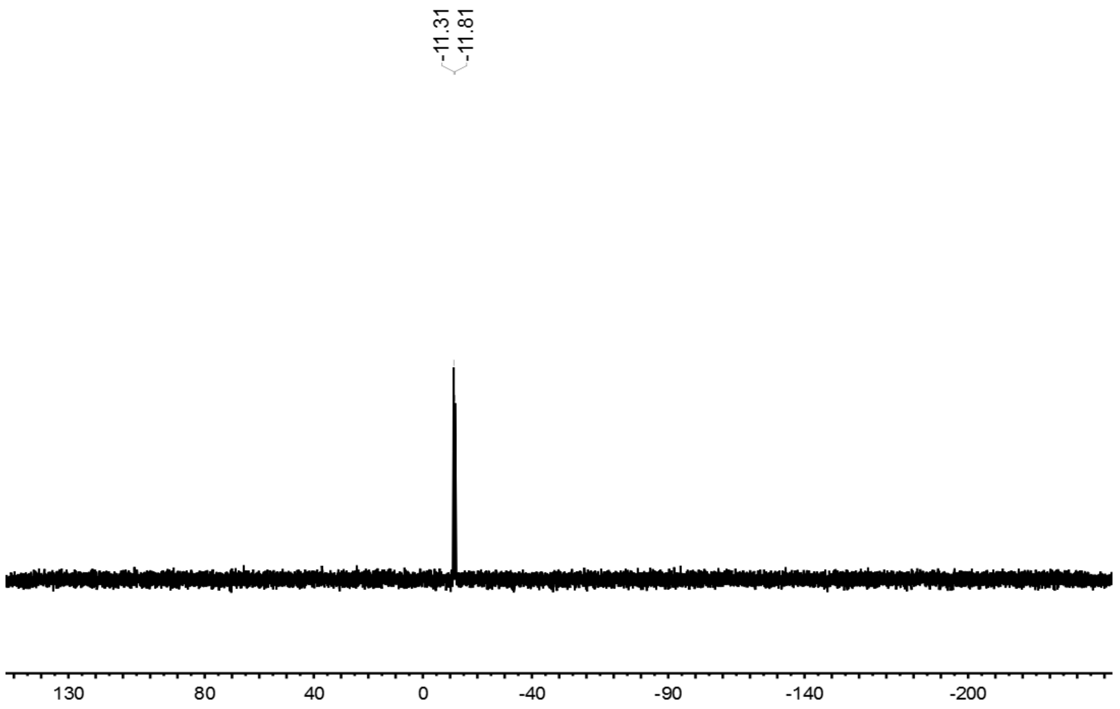

β-8-Br-ADPr-N_3 (**3'**)

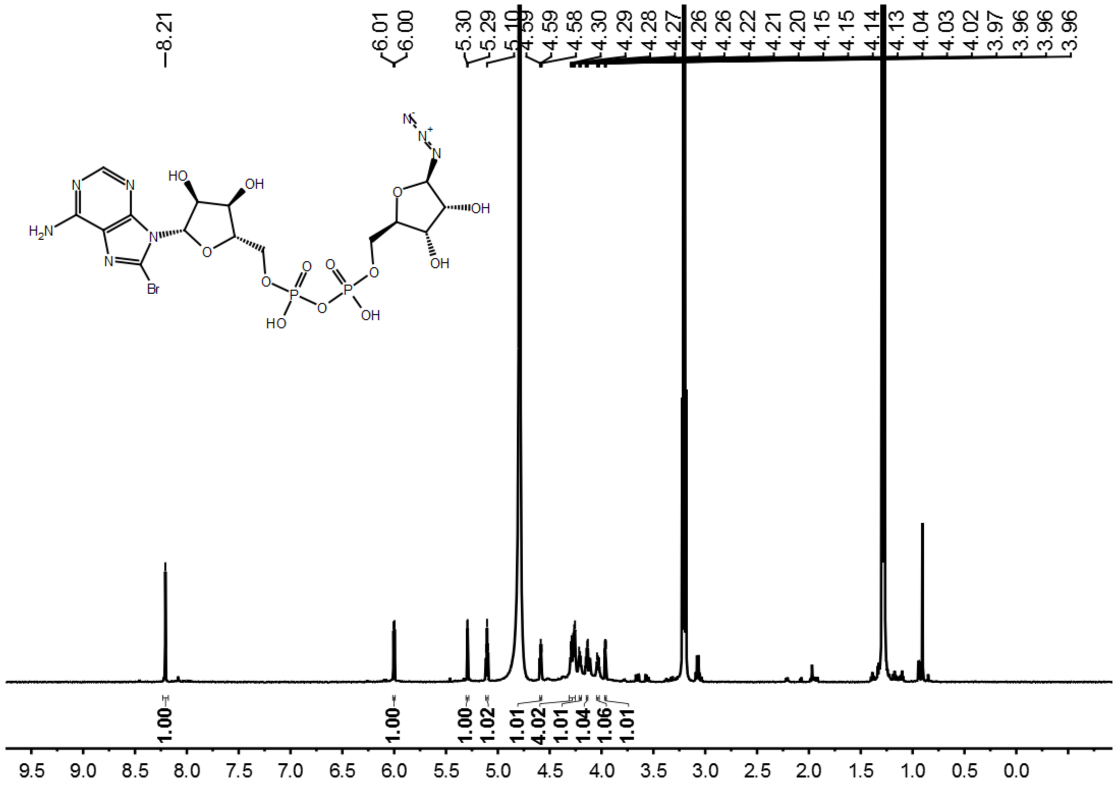

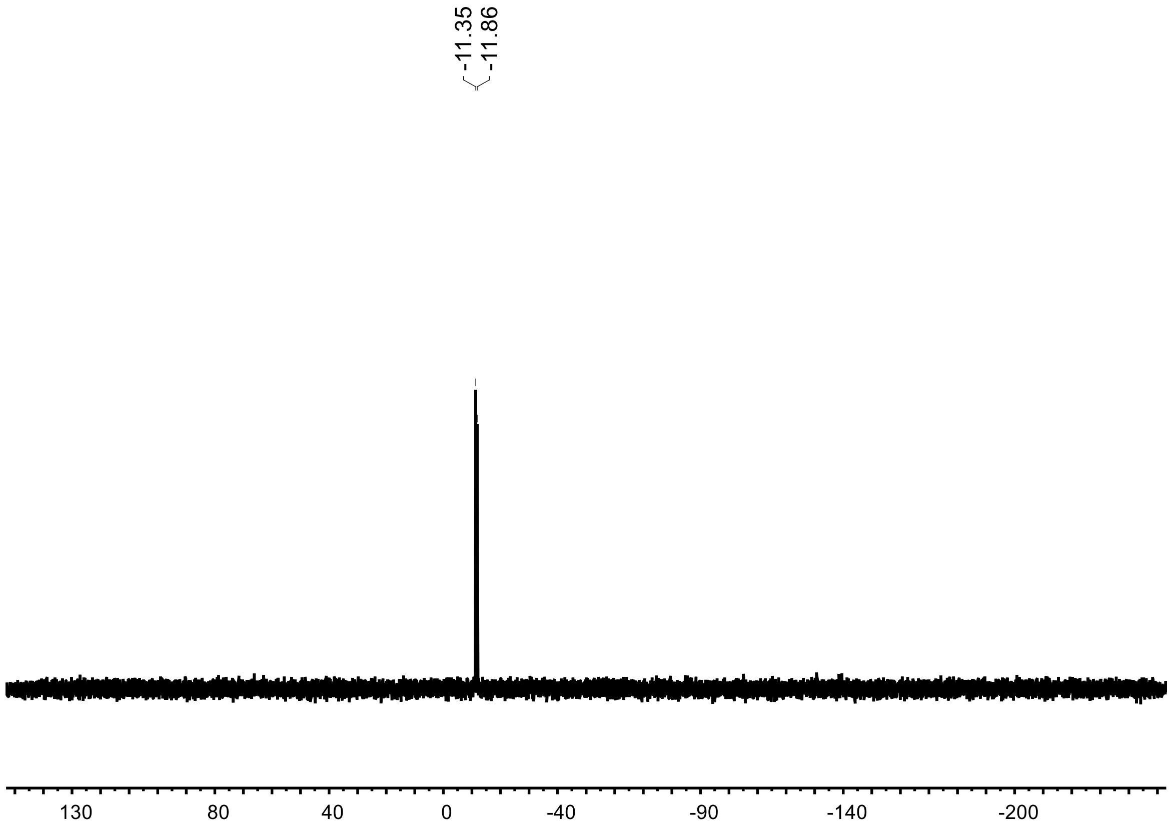

Copies of NMR spectra of the synthesized ionic liquids

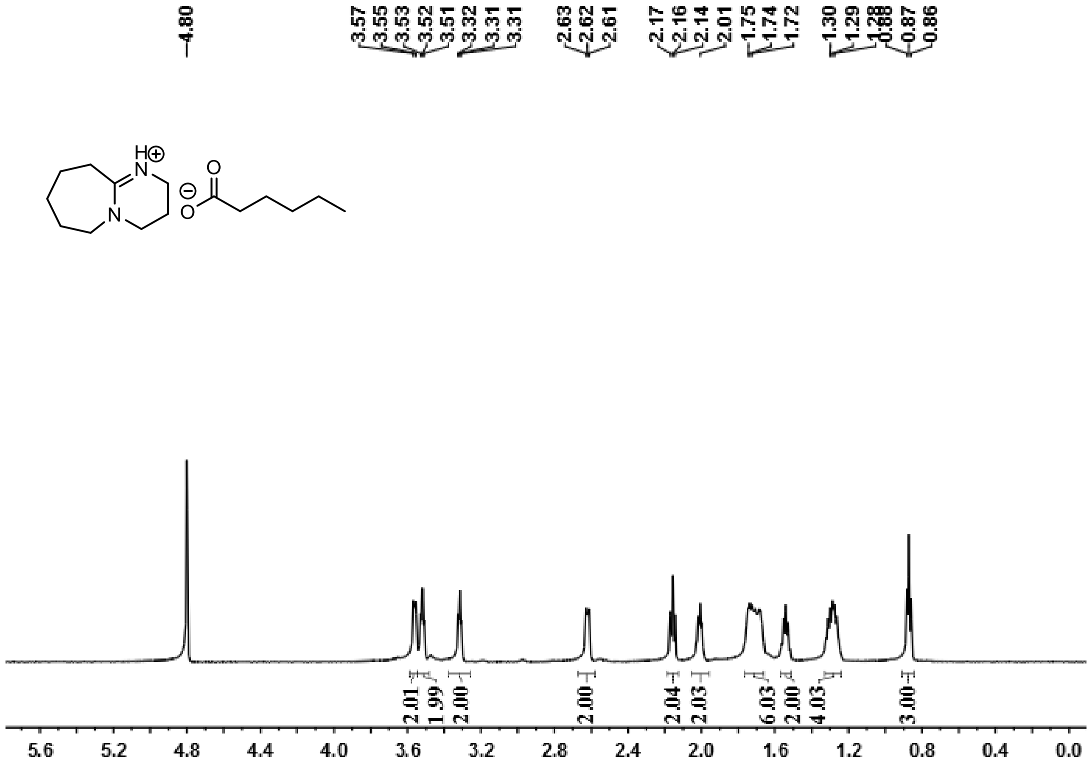

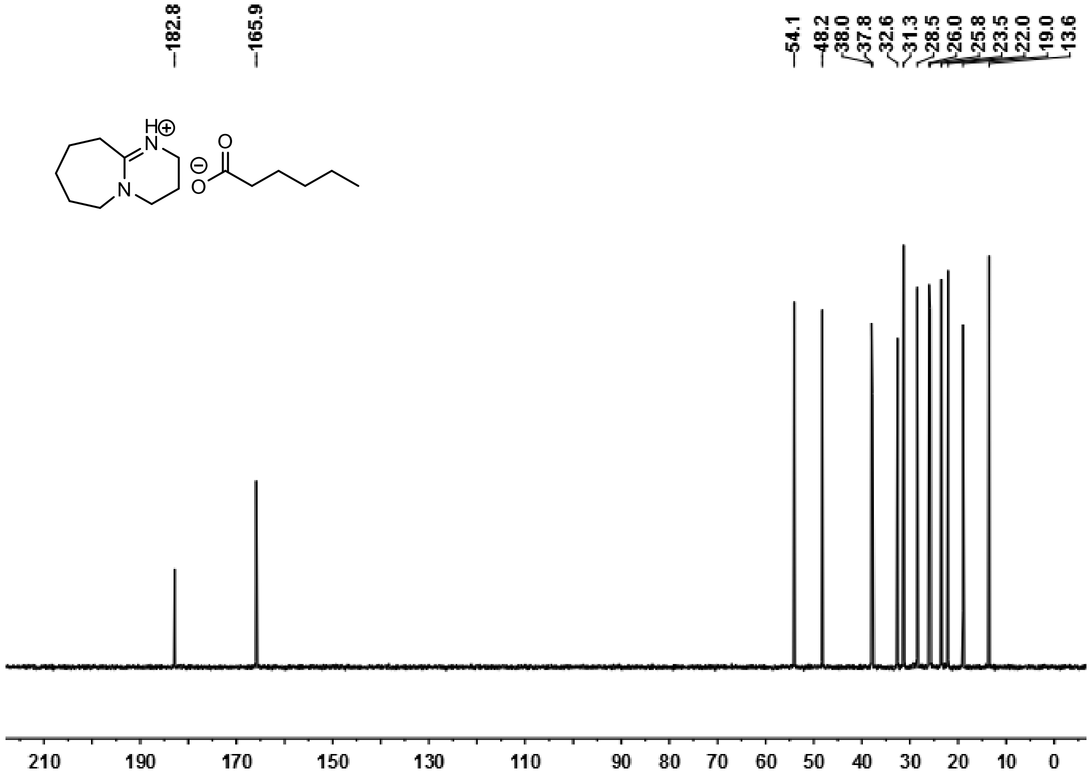

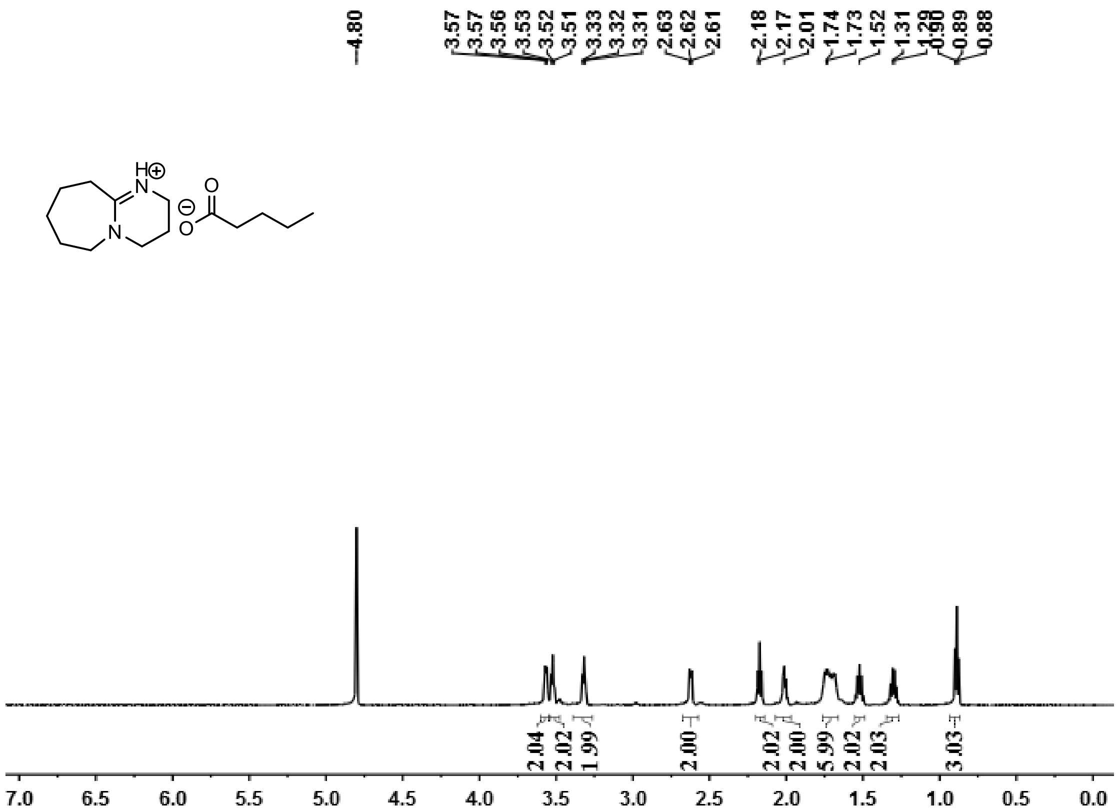
4.80
3.57
3.57
3.56
3.53
3.52
3.51
3.33
3.32
3.31
2.63
2.62
2.61
2.18
2.17
2.01
1.74
1.73
1.52
1.31
1.29
0.90
0.89
0.88
H⊕
N
N
O
O
⊖
2.04
2.02
1.99
2.00
2.02
2.00
5.99
2.02
2.03
3.03
7.0 6.5 6.0 5.5 5.0 4.5 4.0 3.5 3.0 2.5 2.0 1.5 1.0 0.5 0.0

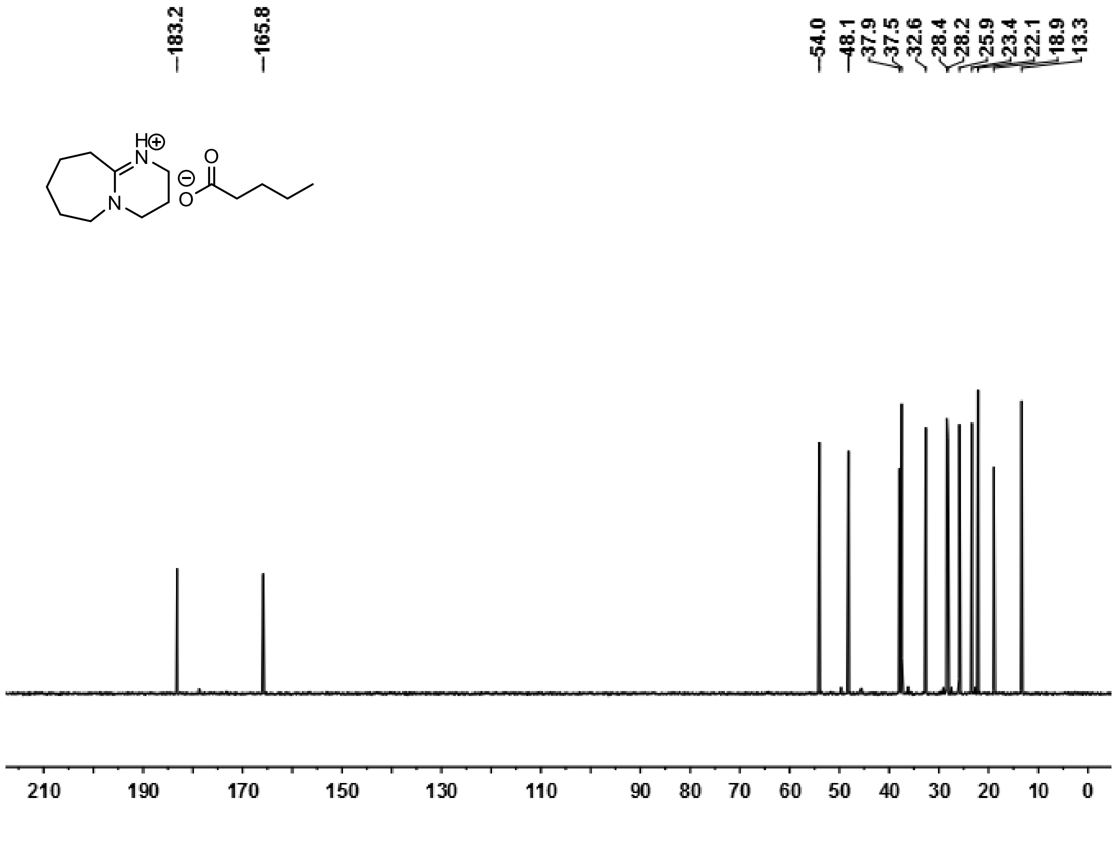
183.2
165.8
54.0
48.1
37.9
37.5
32.6
28.4
28.2
25.9
23.4
22.1
18.9
13.3
H⊕
N
N
O
O
⊖
210 190 170 150 130 110 90 80 70 60 50 40 30 20 10 0

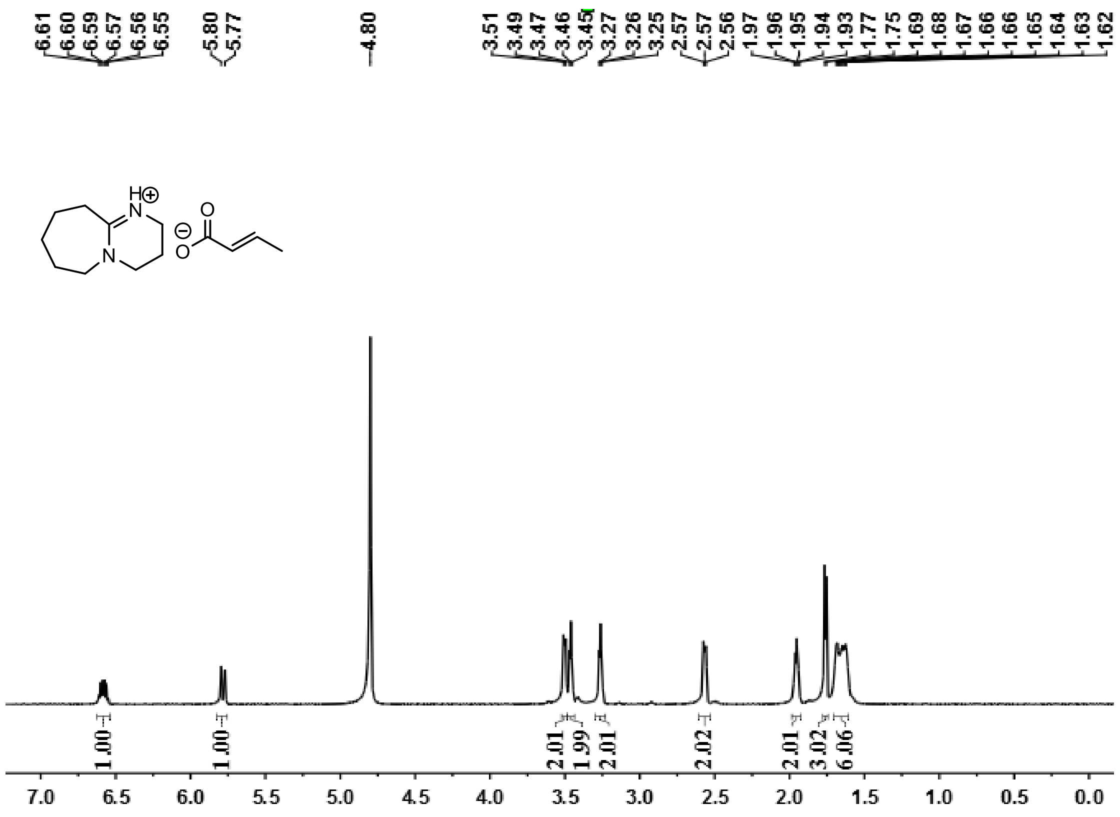
6.61
6.60
6.59
6.57
6.56
6.55
5.80
5.77
4.80
3.51
3.49
3.47
3.46
3.45
3.27
3.26
3.25
2.57
2.57
2.56
1.97
1.96
1.95
1.94
1.93
1.77
1.75
1.69
1.68
1.67
1.66
1.66
1.65
1.64
1.63
1.62
1.00
1.00
2.01
1.99
2.01
2.02
2.01
3.02
6.06
7.0 6.5 6.0 5.5 5.0 4.5 4.0 3.5 3.0 2.5 2.0 1.5 1.0 0.5 0.0

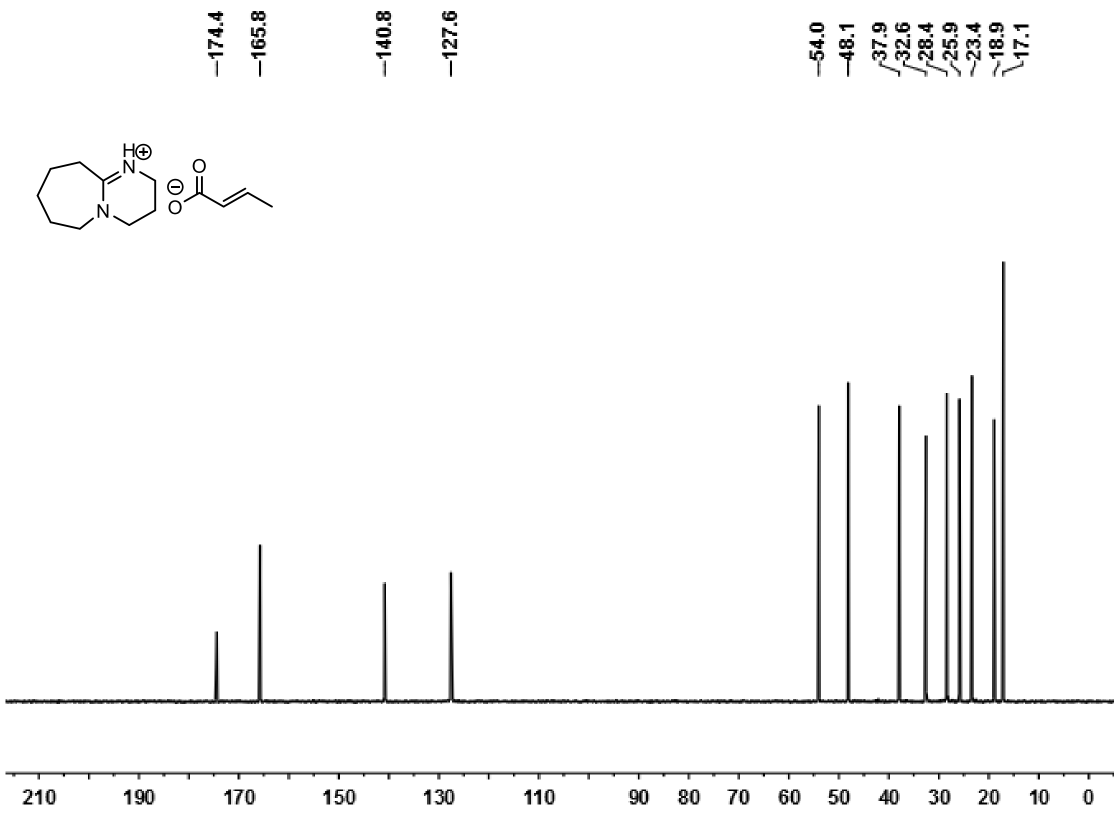
174.4
165.8
140.8
127.6
54.0
48.1
37.9
32.6
28.4
25.9
23.4
18.9
17.1
210 190 170 150 130 110 90 80 70 60 50 40 30 20 10 0

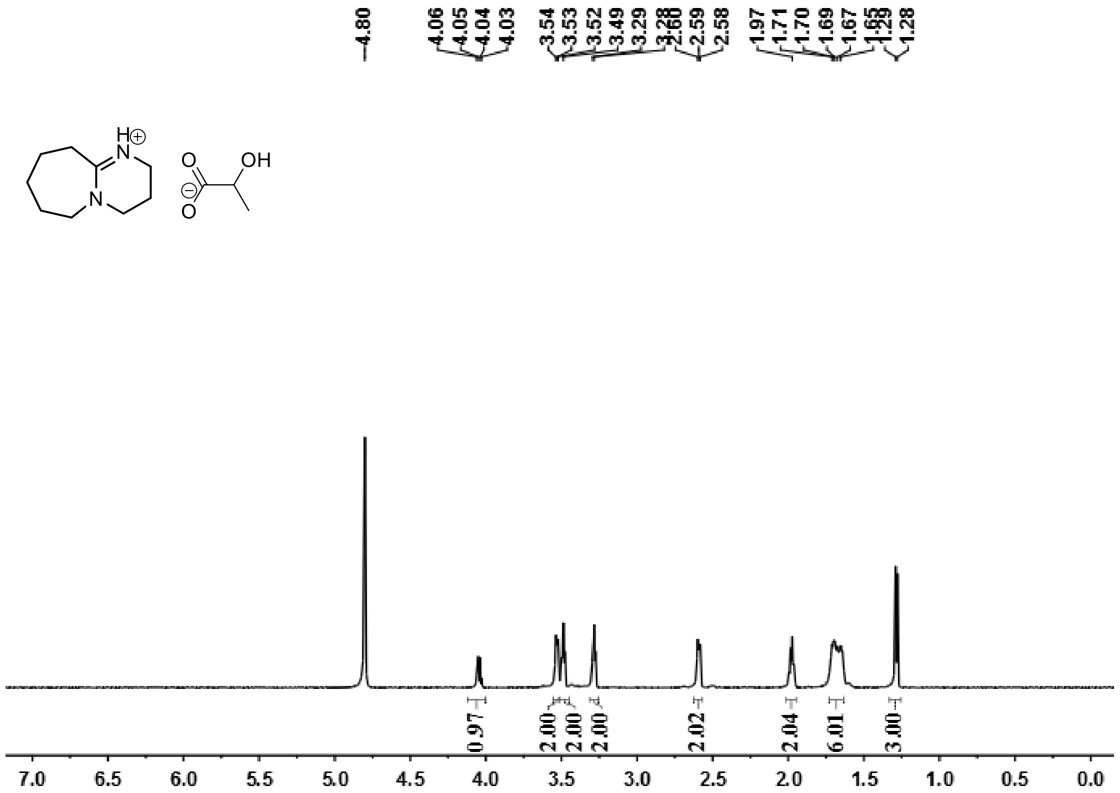
4.80
4.06
4.05
4.04
4.03
3.54
3.53
3.52
3.49
3.29
3.28
2.60
2.59
2.58
1.97
1.71
1.70
1.69
1.67
1.65
1.29
1.28
0.97
2.00
2.00
2.00
2.02
2.04
6.01
3.00
7.0
6.5
6.0
5.5
5.0
4.5
4.0
3.5
3.0
2.5
2.0
1.5
1.0
0.5
0.0

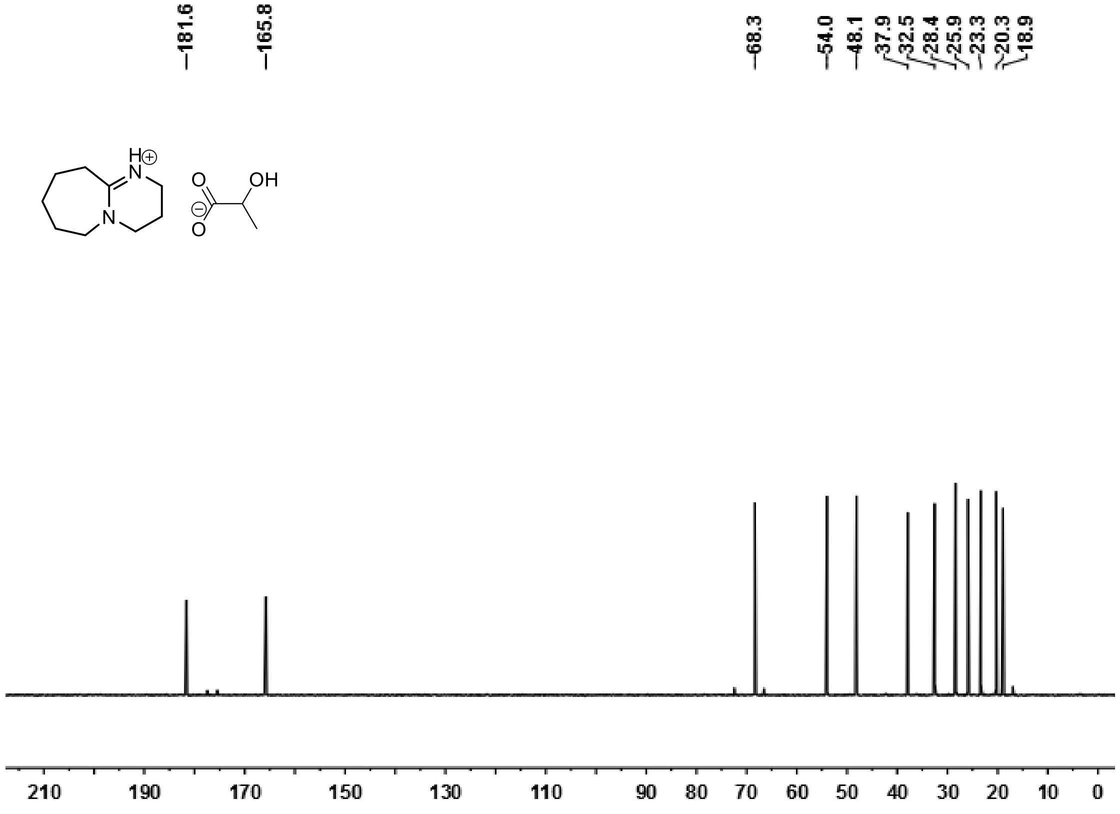
181.6
165.8
68.3
54.0
48.1
37.9
32.5
28.4
25.9
23.3
20.3
18.9
210
190
170
150
130
110
90
80
70
60
50
40
30
20
10
0

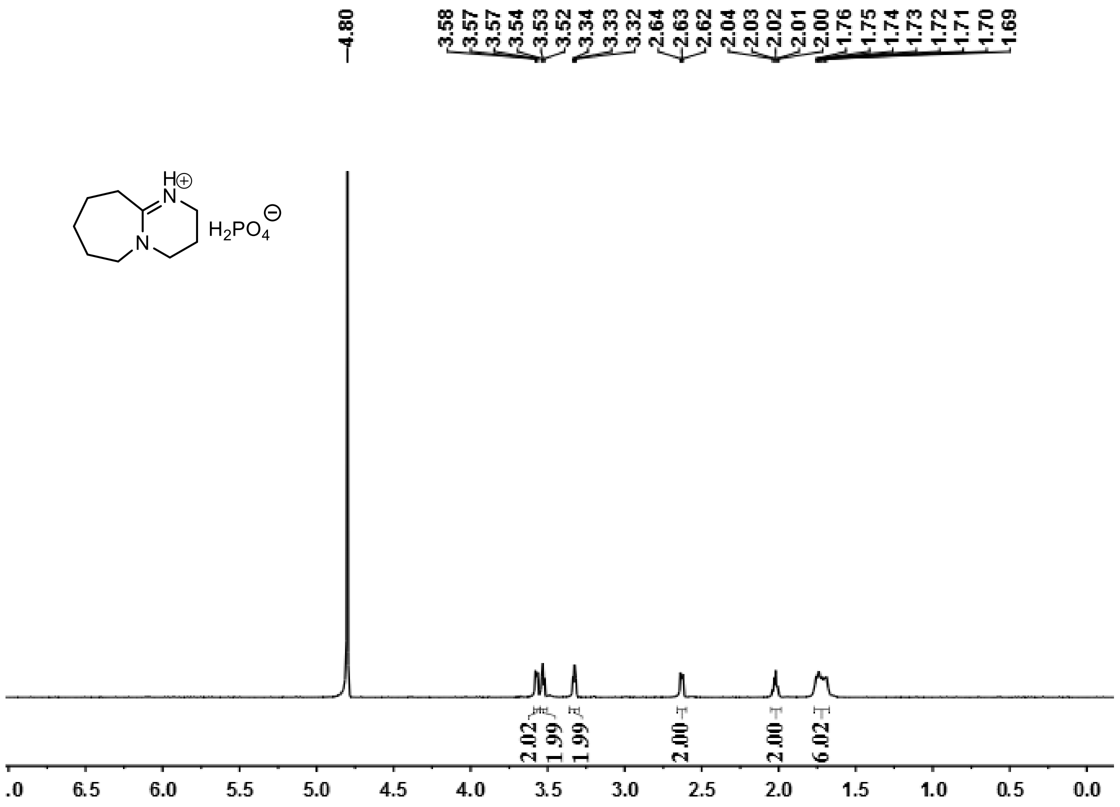
4.80
3.58
3.57
3.57
3.54
3.53
3.52
3.34
3.33
3.32
2.64
2.63
2.62
2.04
2.03
2.02
2.01
2.00
1.76
1.75
1.74
1.73
1.72
1.71
1.70
1.69
2.02
1.99
1.99
2.00
2.00
6.02
$H_2PO_4^{\ominus}$
N
N
H⊕

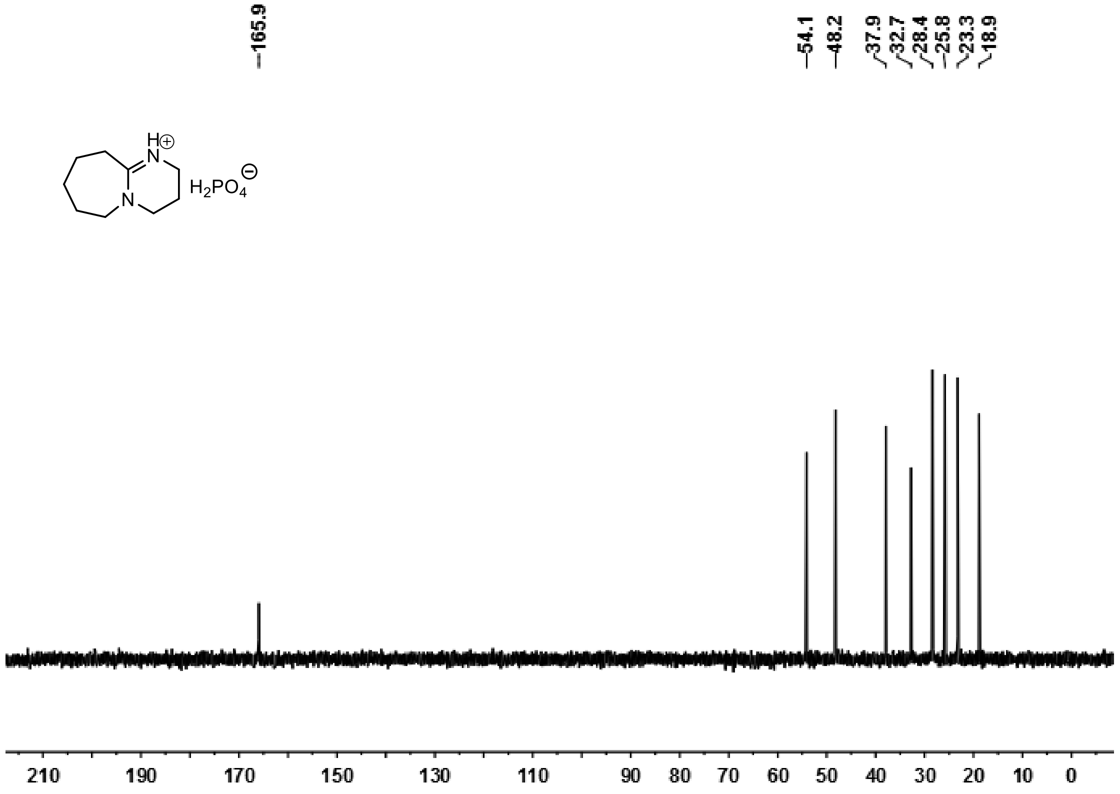
165.9
54.1
48.2
37.9
32.7
28.4
25.8
23.3
18.9
$H_2PO_4^{\ominus}$
N
N
H⊕

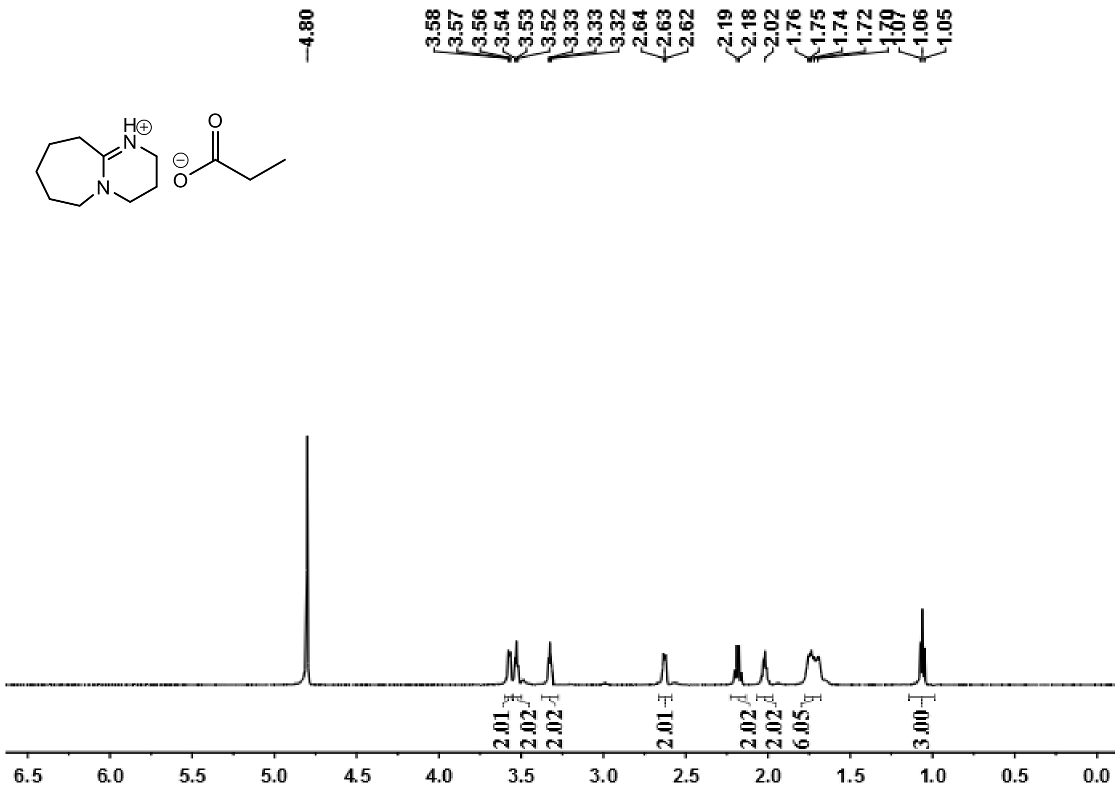
4.80
3.58 3.57 3.56 3.54 3.53 3.52 3.33 3.33 3.32 2.64 2.63 2.62 2.19 2.18 2.02 1.76 1.75 1.74 1.72 1.70 1.07 1.06 1.05
2.01 2.02 2.02 2.01 2.02 2.02 6.05 3.00
6.5 6.0 5.5 5.0 4.5 4.0 3.5 3.0 2.5 2.0 1.5 1.0 0.5 0.0

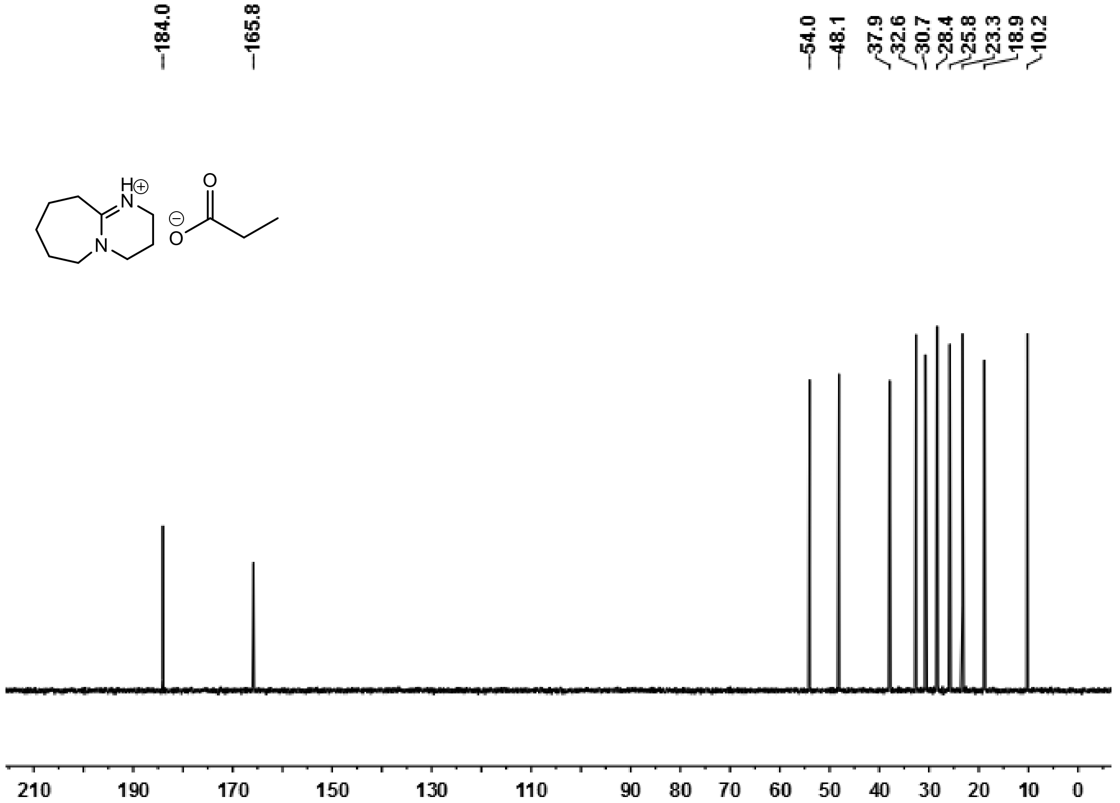
184.0
165.8
54.0 48.1 37.9 32.6 30.7 28.4 25.8 23.3 18.9 10.2
210 190 170 150 130 110 90 80 70 60 50 40 30 20 10 0

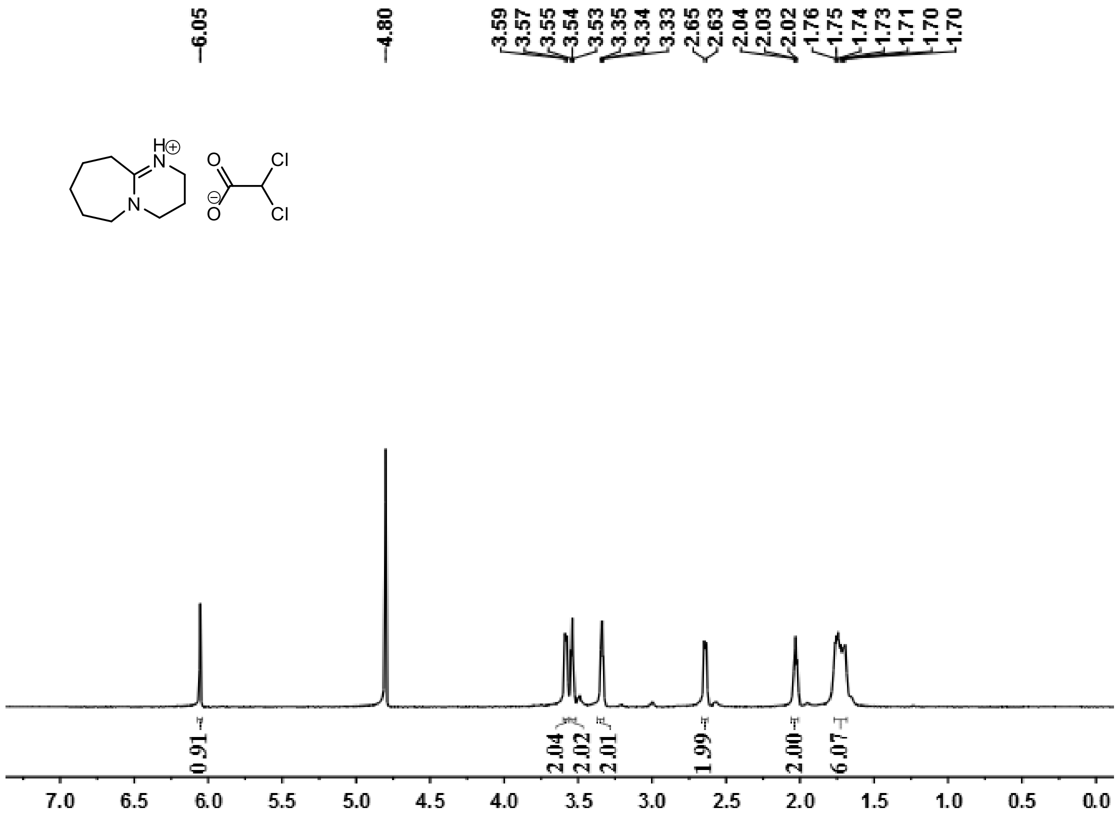
6.05
4.80
3.59 3.57 3.55 3.54 3.53 3.35 3.34 3.33 2.65 2.63 2.04 2.03 2.02 1.76 1.75 1.74 1.73 1.71 1.70 1.70
H⊕
N
O
Cl
O⊖
0.91
2.04
2.02
2.01
1.99
2.00
6.07
7.0 6.5 6.0 5.5 5.0 4.5 4.0 3.5 3.0 2.5 2.0 1.5 1.0 0.5 0.0

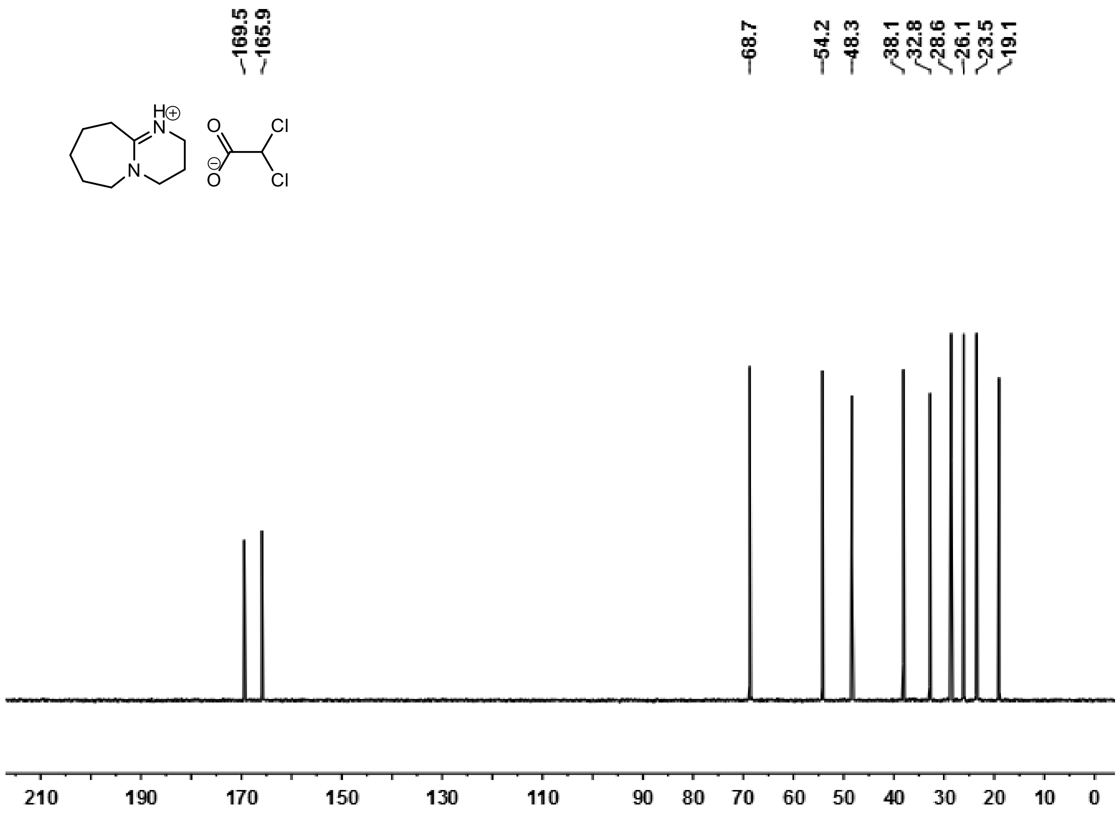
169.5
165.9
68.7
54.2
48.3
38.1 32.8 28.6 26.1 23.5 19.1
H⊕
N
O
Cl
O⊖
210 190 170 150 130 110 90 80 70 60 50 40 30 20 10 0

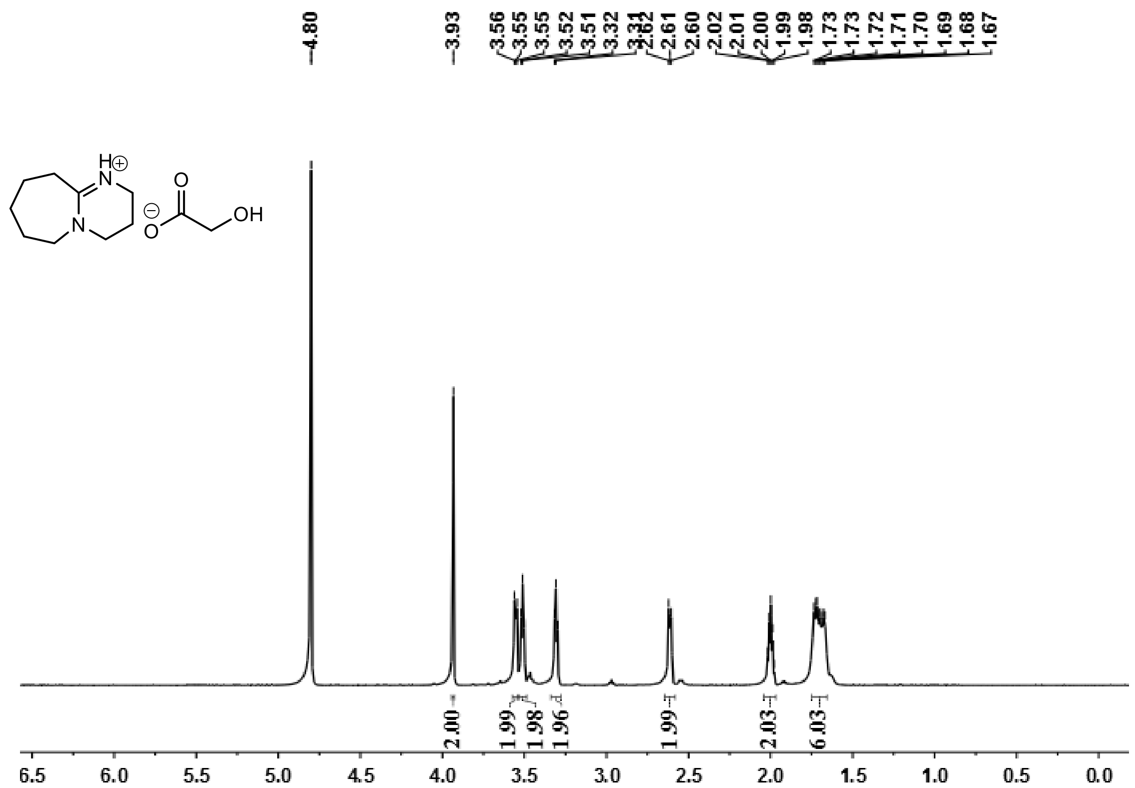

4.80
3.93
3.56
3.55
3.55
3.52
3.51
3.32
3.31
2.62
2.61
2.60
2.02
2.01
2.00
1.99
1.98
1.73
1.73
1.72
1.71
1.70
1.69
1.68
1.67
2.00
1.99
1.98
1.96
1.99
2.03
6.03
6.5
6.0
5.5
5.0
4.5
4.0
3.5
3.0
2.5
2.0
1.5
1.0
0.5
0.0

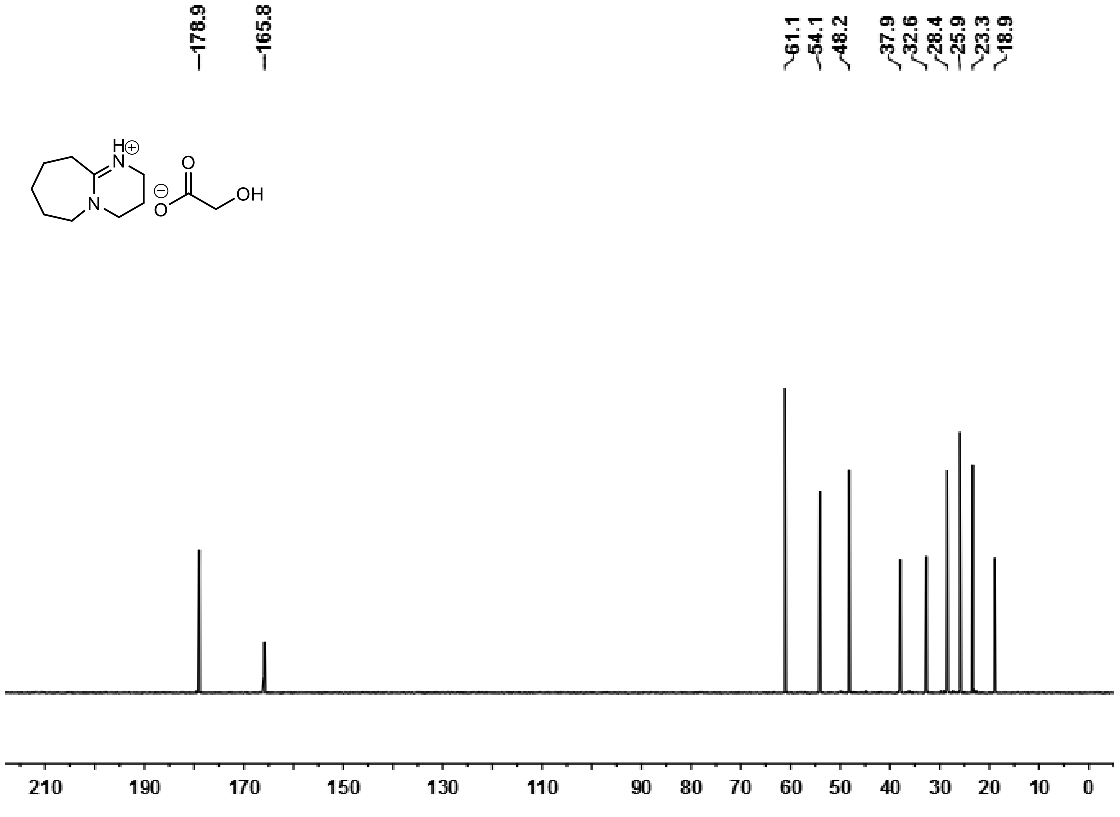

178.9
165.8
61.1
54.1
48.2
37.9
32.6
28.4
25.9
23.3
18.9
210
190
170
150
130
110
90
80
70
60
50
40
30
20
10
0

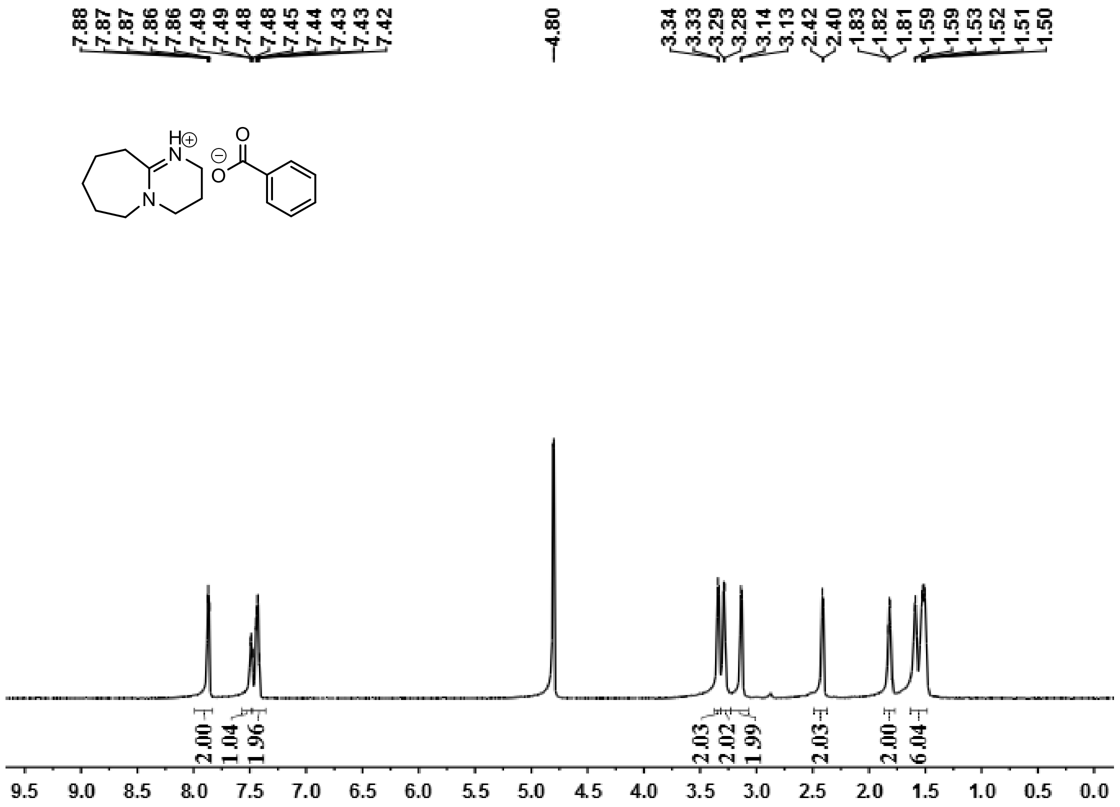
7.88 7.87 7.87 7.86 7.86 7.49 7.49 7.48 7.48 7.45 7.44 7.43 7.43 7.42
4.80
3.34 3.33 3.29 3.28 3.14 3.13 2.42 2.40 1.83 1.82 1.81 1.59 1.59 1.53 1.52 1.51 1.50
H⊕
N
O
⊖
O
2.00 1.04 1.96 2.03 2.02 1.99 2.03 2.00 6.04
9.5 9.0 8.5 8.0 7.5 7.0 6.5 6.0 5.5 5.0 4.5 4.0 3.5 3.0 2.5 2.0 1.5 1.0 0.5 0.0

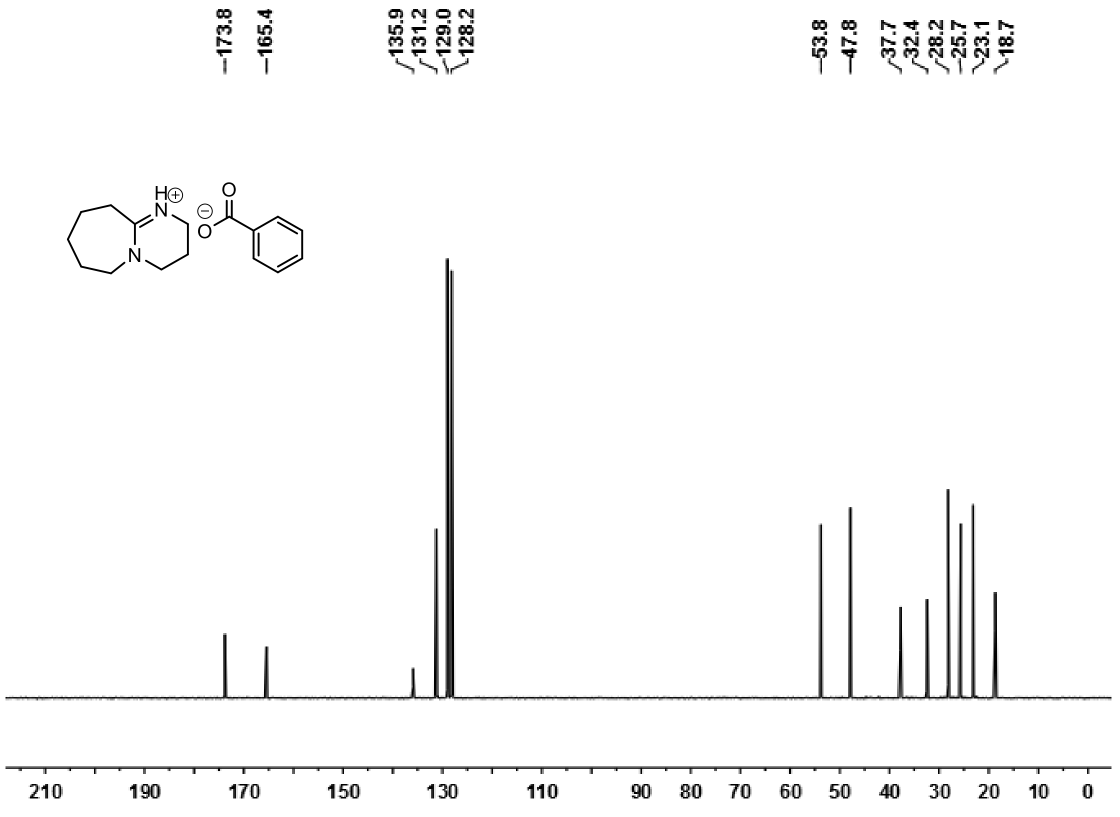
173.8 165.4 135.9 131.2 129.0 128.2 53.8 47.8 37.7 32.4 28.2 25.7 23.1 18.7
H⊕
N
O
⊖
O
210 190 170 150 130 110 90 80 70 60 50 40 30 20 10 0

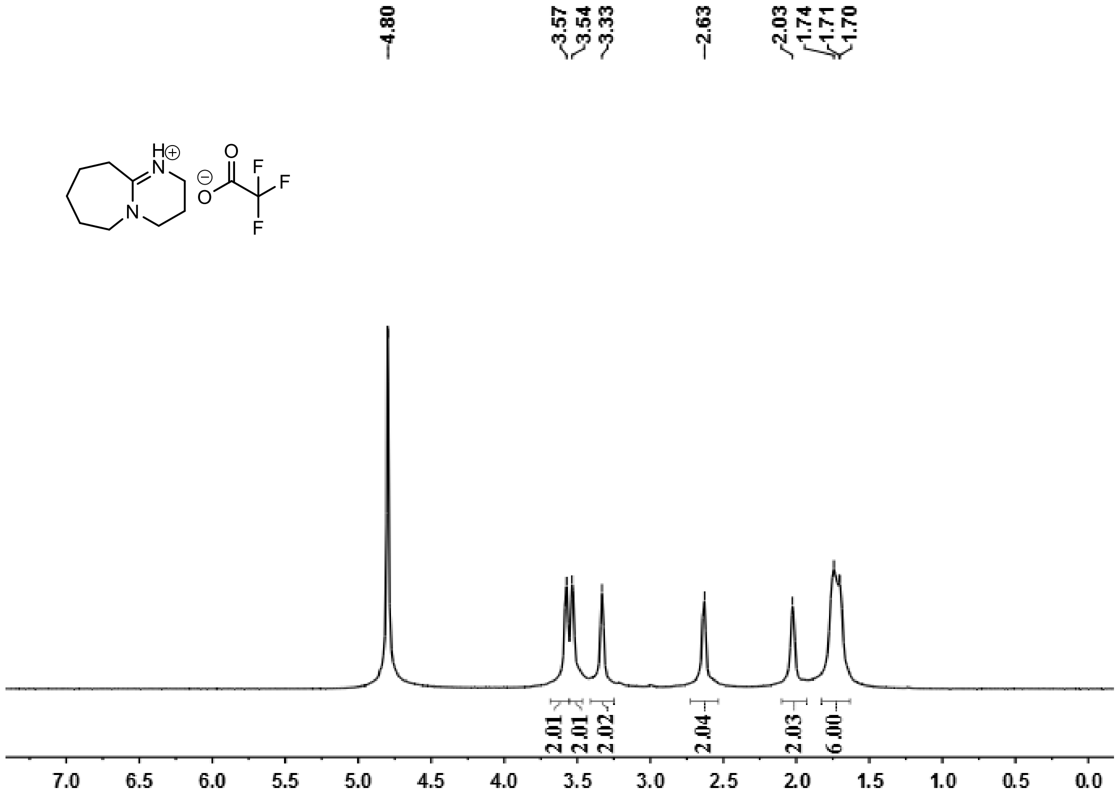
4.80
3.57
3.54
3.33
2.63
2.03
1.74
1.71
1.70
2.01
2.01
2.02
2.04
2.03
6.00
7.0 6.5 6.0 5.5 5.0 4.5 4.0 3.5 3.0 2.5 2.0 1.5 1.0 0.5 0.0

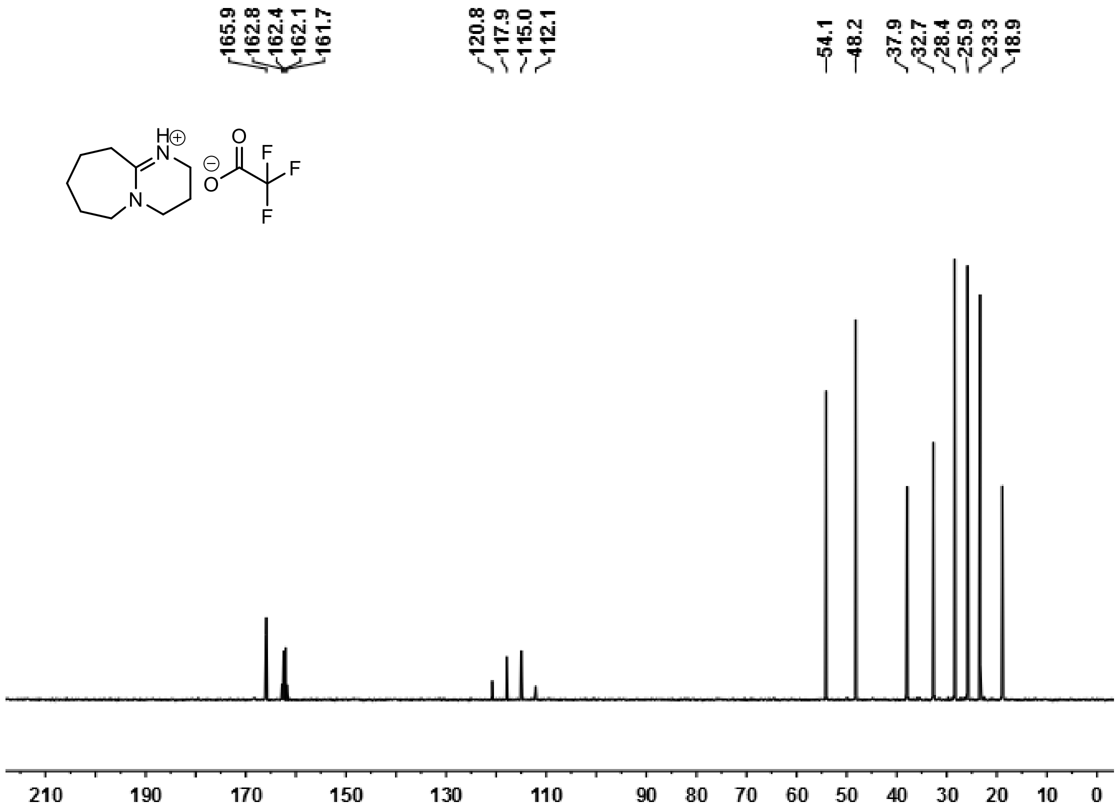
165.9
162.8
162.4
162.1
161.7
120.8
117.9
115.0
112.1
54.1
48.2
37.9
32.7
28.4
25.9
23.3
18.9
210 190 170 150 130 110 90 80 70 60 50 40 30 20 10 0

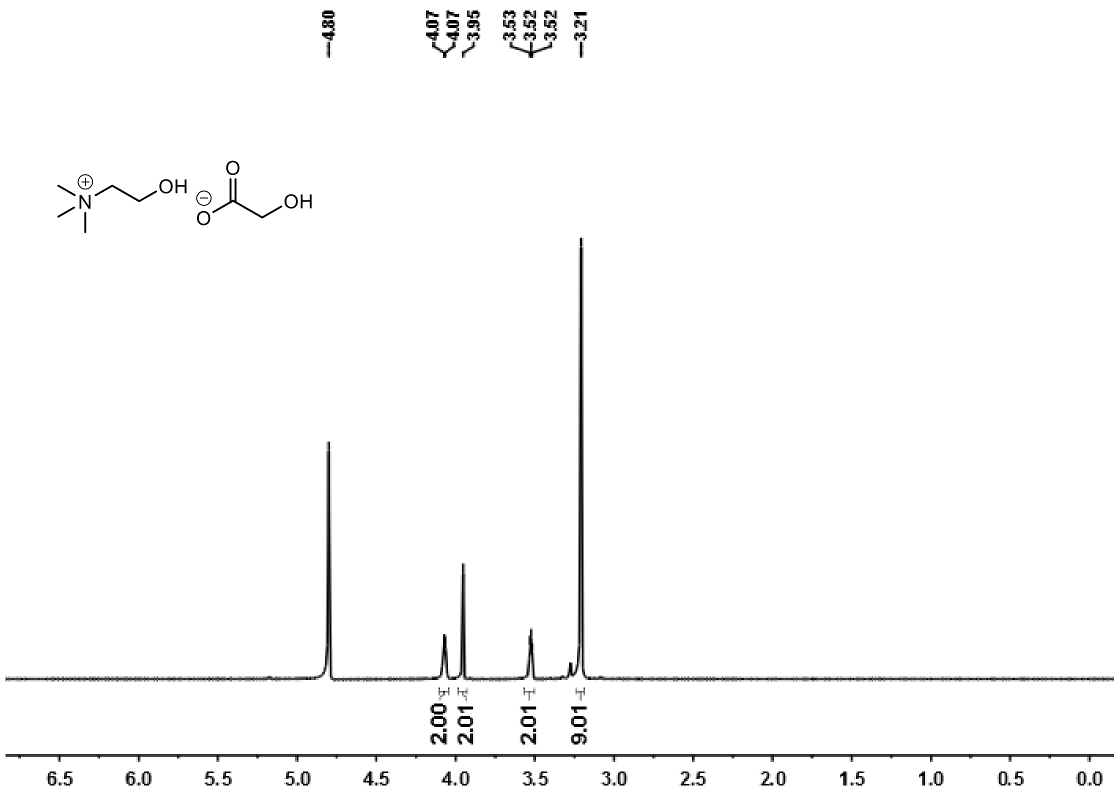
4.80
4.07
4.07
3.95
3.53
3.52
3.52
3.21
2.00
2.01
2.01
9.01
6.5 6.0 5.5 5.0 4.5 4.0 3.5 3.0 2.5 2.0 1.5 1.0 0.5 0.0

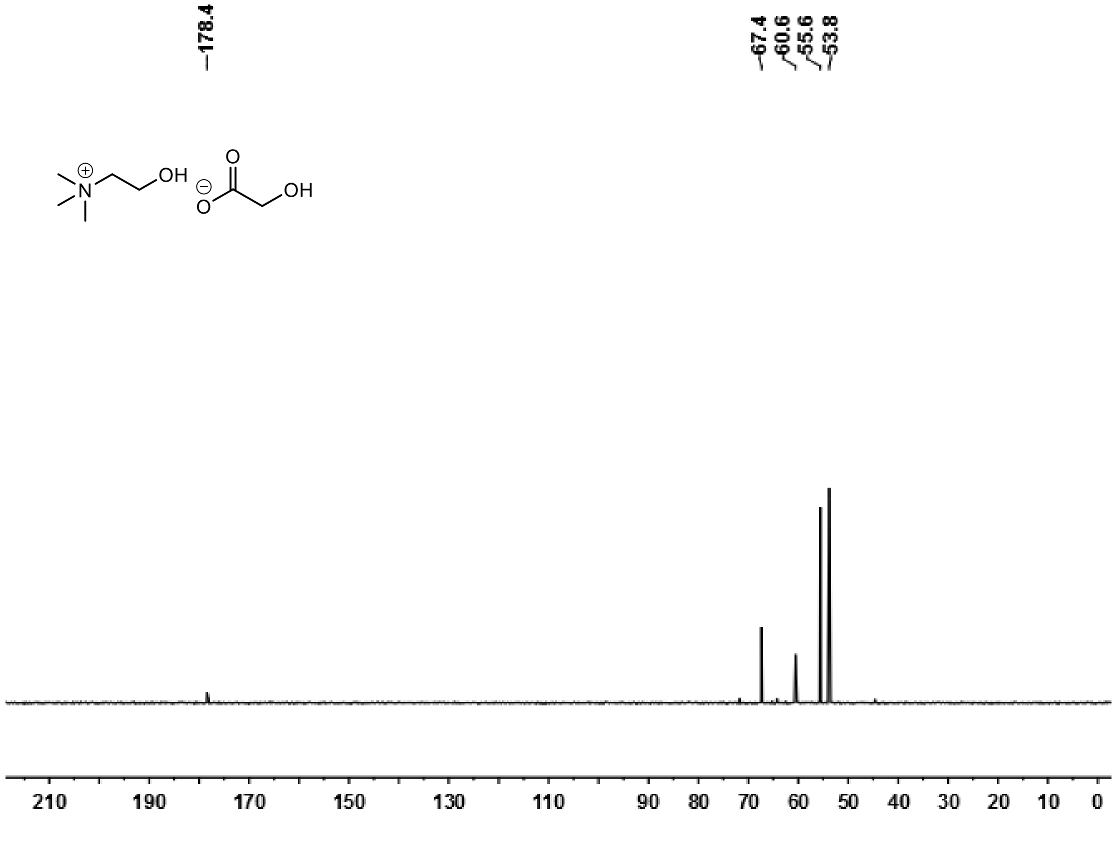
178.4
67.4
60.6
55.6
53.8
210 190 170 150 130 110 90 80 70 60 50 40 30 20 10 0

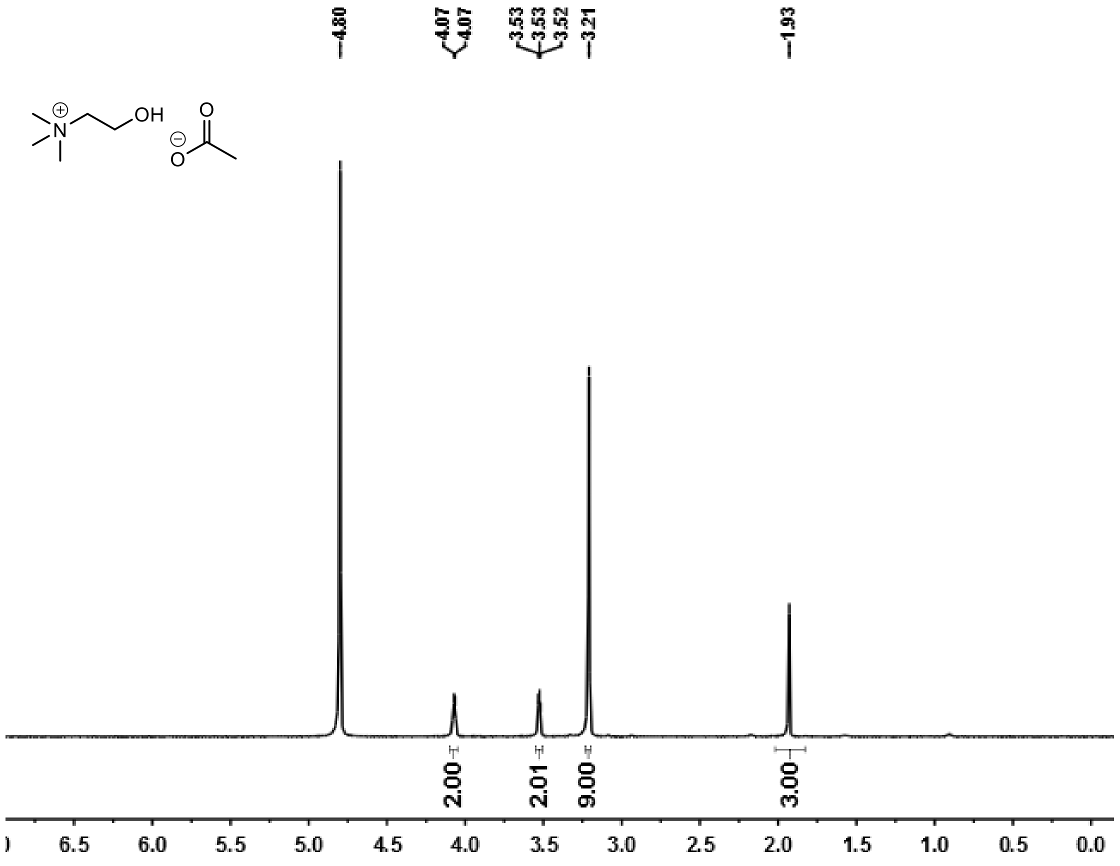
4.80
4.07
4.07
3.53
3.53
3.52
3.21
1.93
OH
O
O
2.00
2.01
9.00
3.00
6.5 6.0 5.5 5.0 4.5 4.0 3.5 3.0 2.5 2.0 1.5 1.0 0.5 0.0

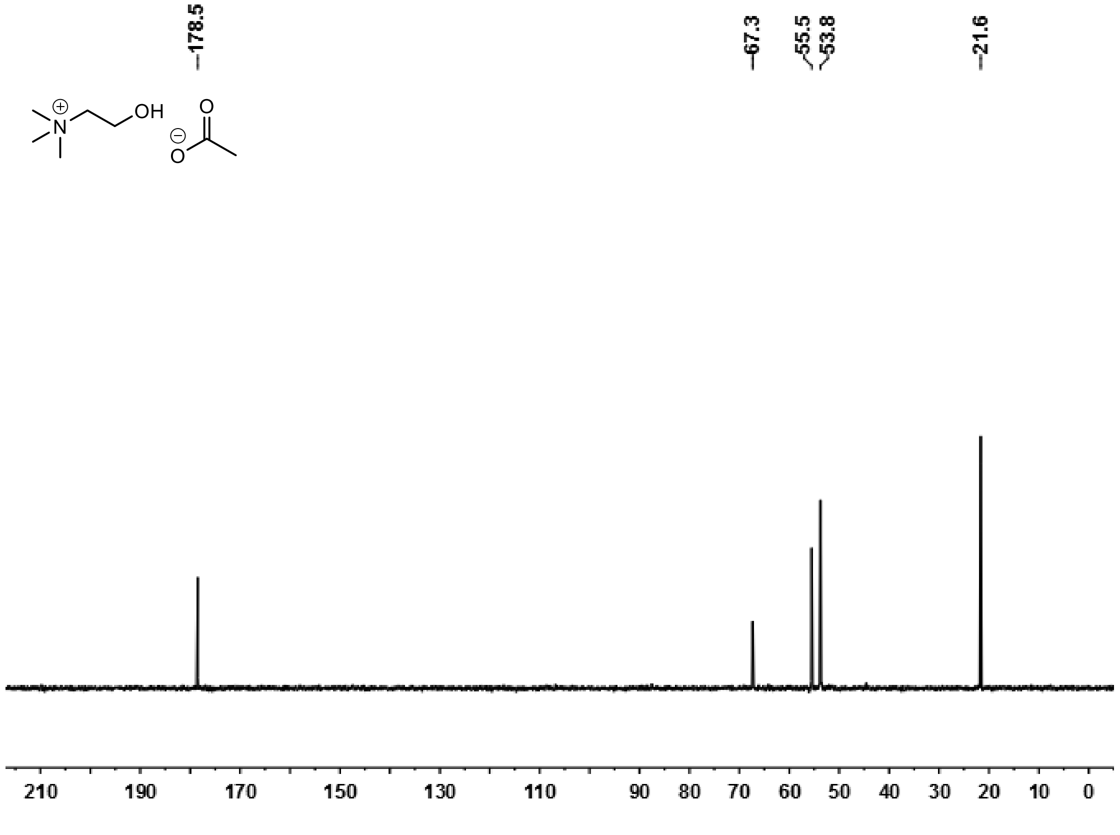
178.5
67.3
55.5
53.8
21.6
OH
O
O
210 190 170 150 130 110 90 80 70 60 50 40 30 20 10 0

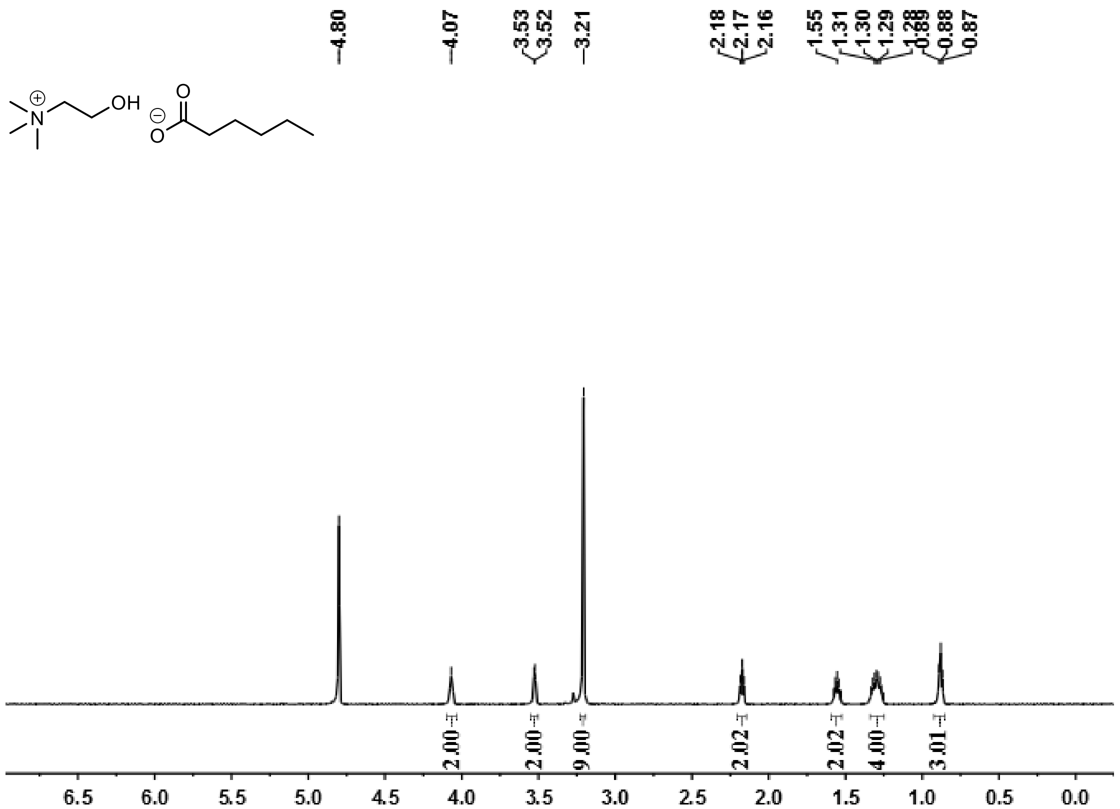

4.80
4.07
3.53
3.52
3.21
2.18
2.17
2.16
1.55
1.31
1.30
1.29
1.28
0.89
0.88
0.87
OH
O
2.00
2.00
9.00
2.02
2.02
4.00
3.01
6.5 6.0 5.5 5.0 4.5 4.0 3.5 3.0 2.5 2.0 1.5 1.0 0.5 0.0

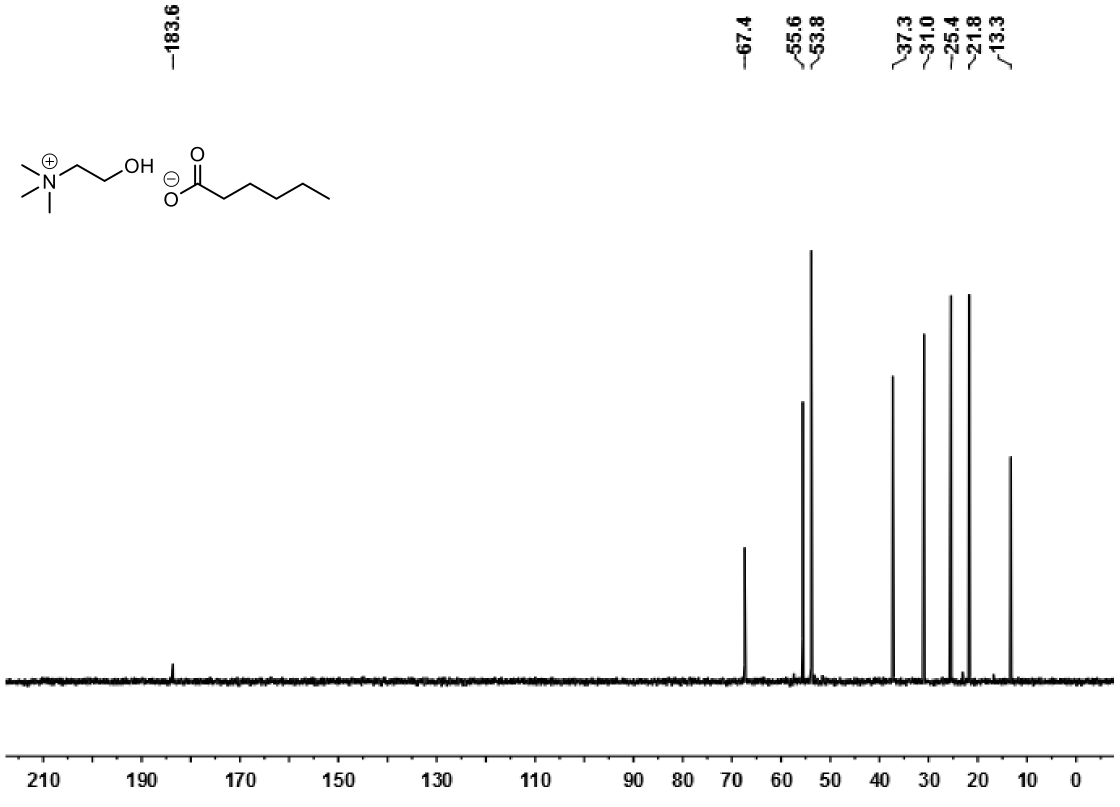

183.6
67.4
55.6
53.8
37.3
31.0
25.4
21.8
13.3
OH
O
210 190 170 150 130 110 90 80 70 60 50 40 30 20 10 0

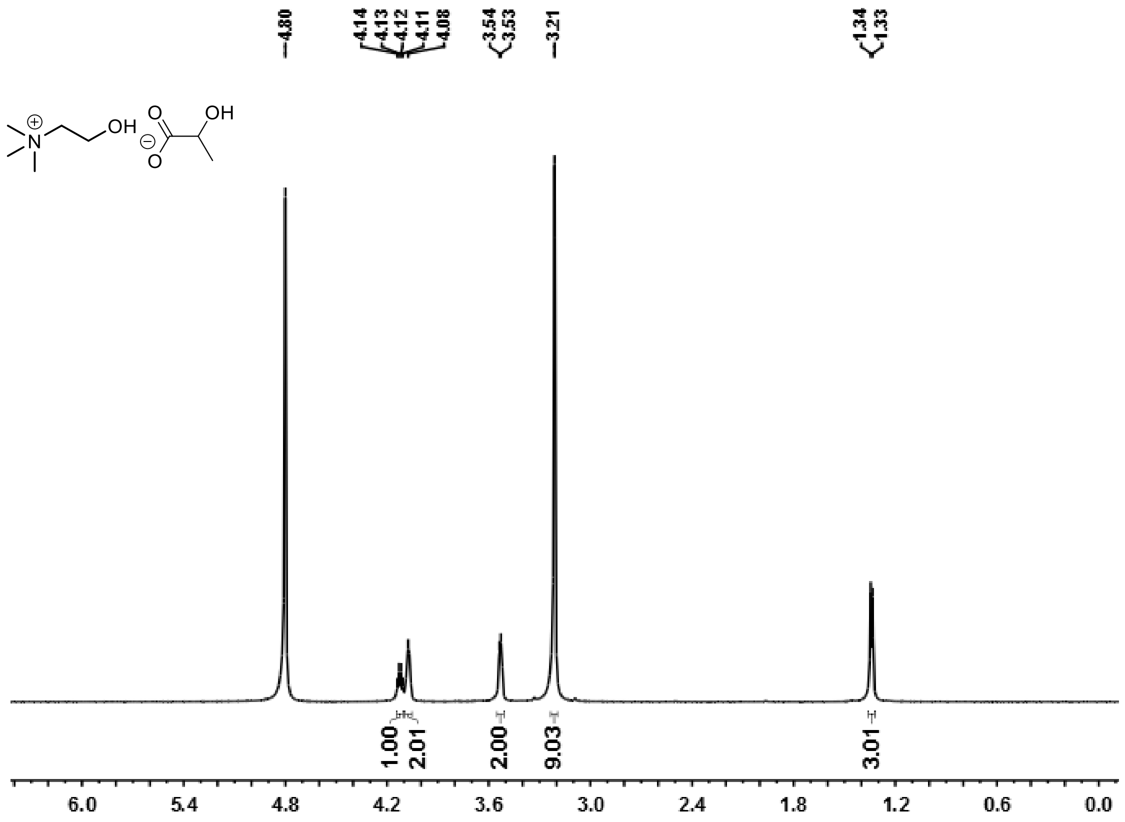
4.80
4.14
4.13
4.12
4.11
4.08
3.54
3.53
3.21
1.34
1.33
OH
O
OH
O
1.00
2.01
2.00
9.03
3.01
6.0
5.4
4.8
4.2
3.6
3.0
2.4
1.8
1.2
0.6
0.0

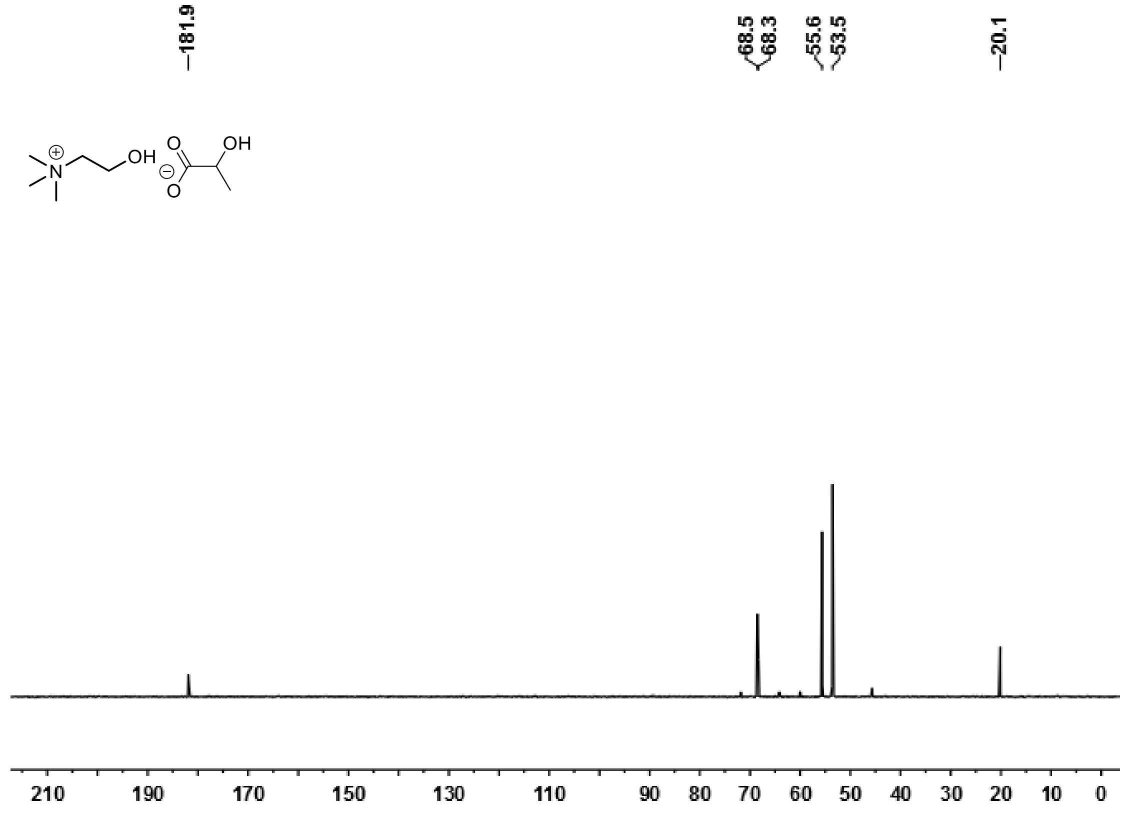
181.9
68.5
68.3
55.6
53.5
20.1
OH
O
OH
O
210
190
170
150
130
110
90
80
70
60
50
40
30
20
10
0

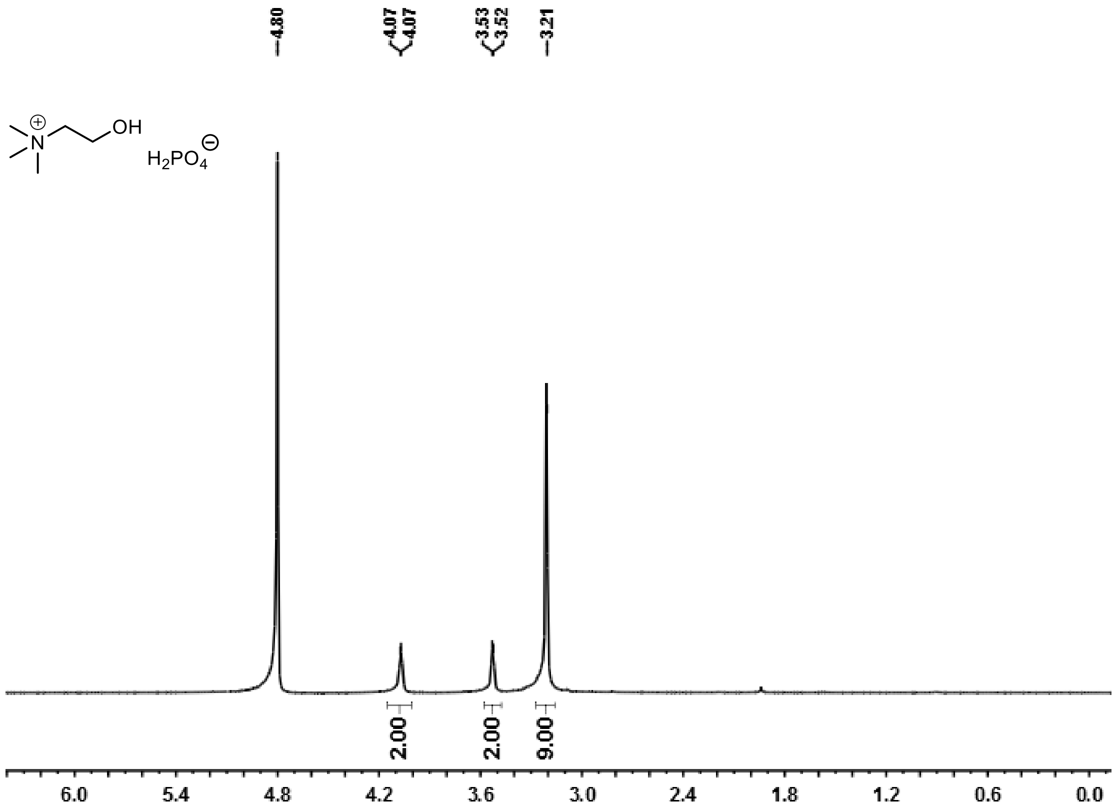

4.80
4.07
4.07
3.53
3.52
3.21
OH
H_2PO_4
2.00
2.00
9.00
6.0 5.4 4.8 4.2 3.6 3.0 2.4 1.8 1.2 0.6 0.0

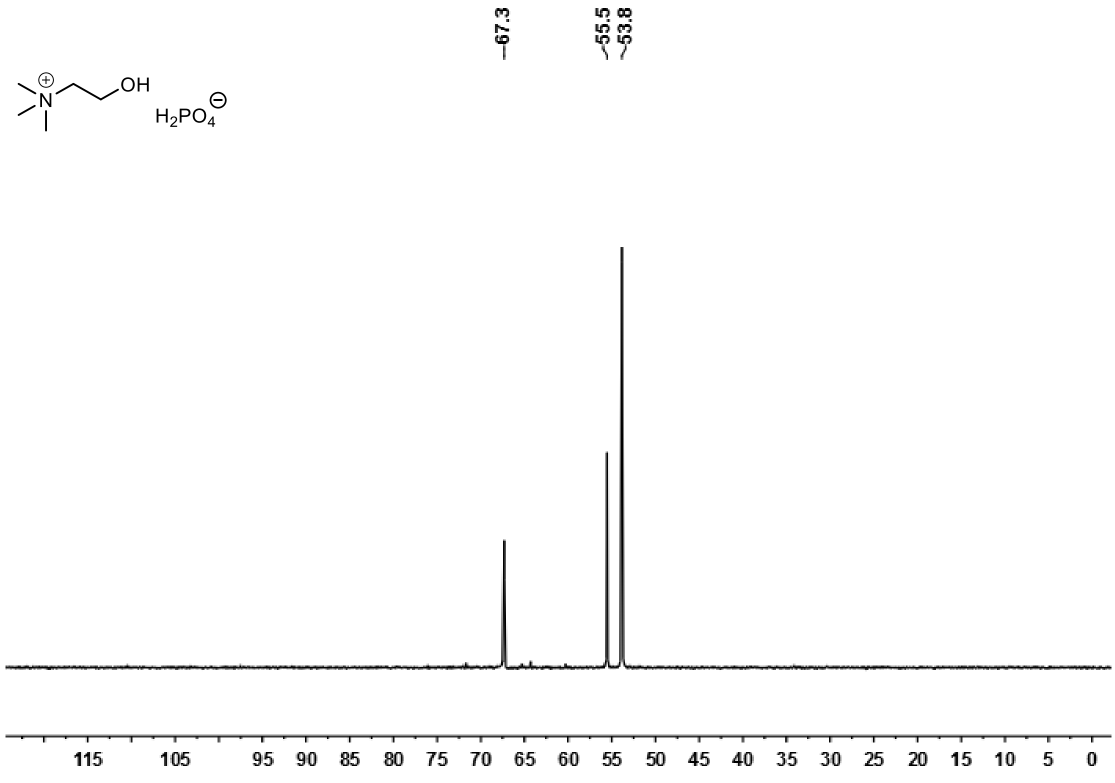

67.3
55.5
53.8
OH
H_2PO_4
115 105 95 90 85 80 75 70 65 60 55 50 45 40 35 30 25 20 15 10 5 0

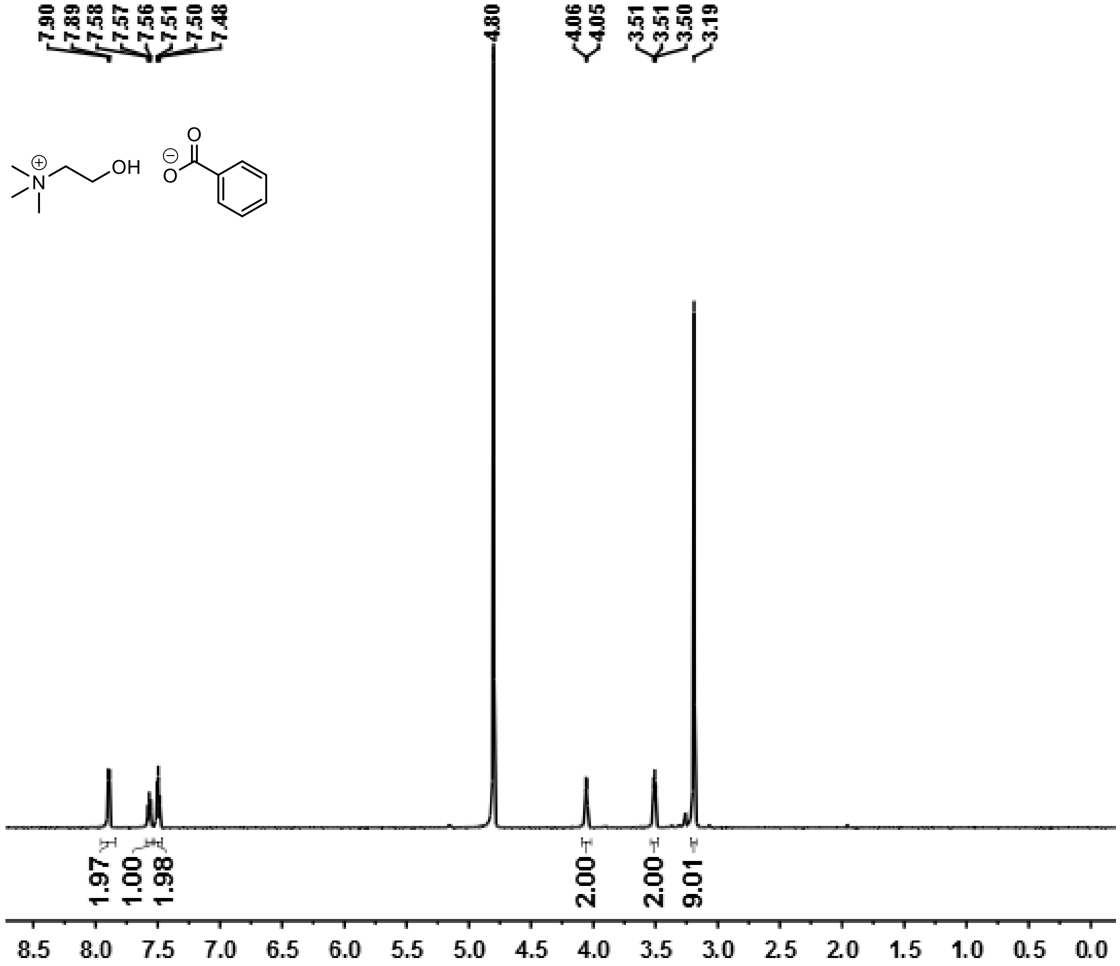
7.90
7.89
7.58
7.57
7.56
7.51
7.50
7.48
4.80
4.06
4.05
3.51
3.51
3.50
3.19
OH
O
O
N
1.97
1.00
1.98
2.00
2.00
9.01
8.5 8.0 7.5 7.0 6.5 6.0 5.5 5.0 4.5 4.0 3.5 3.0 2.5 2.0 1.5 1.0 0.5 0.0

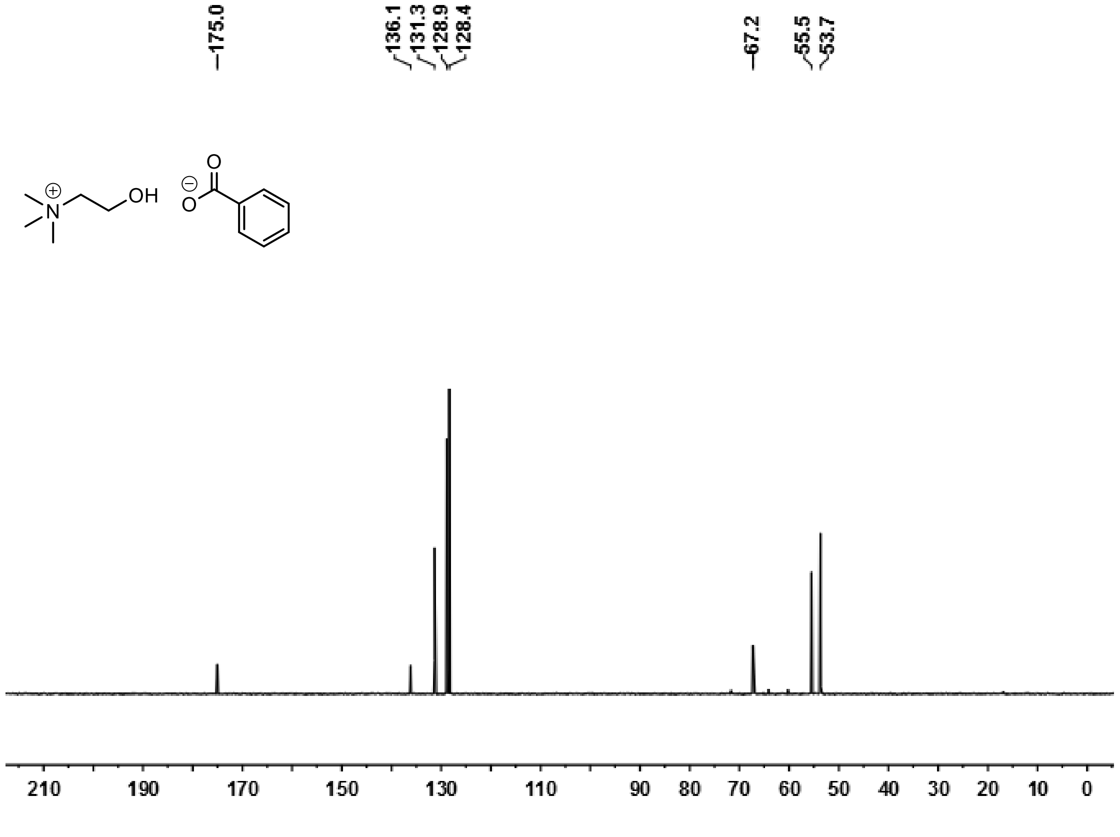
175.0
136.1
131.3
128.9
128.4
67.2
55.5
53.7
OH
O
O
N
210 190 170 150 130 110 90 80 70 60 50 40 30 20 10 0

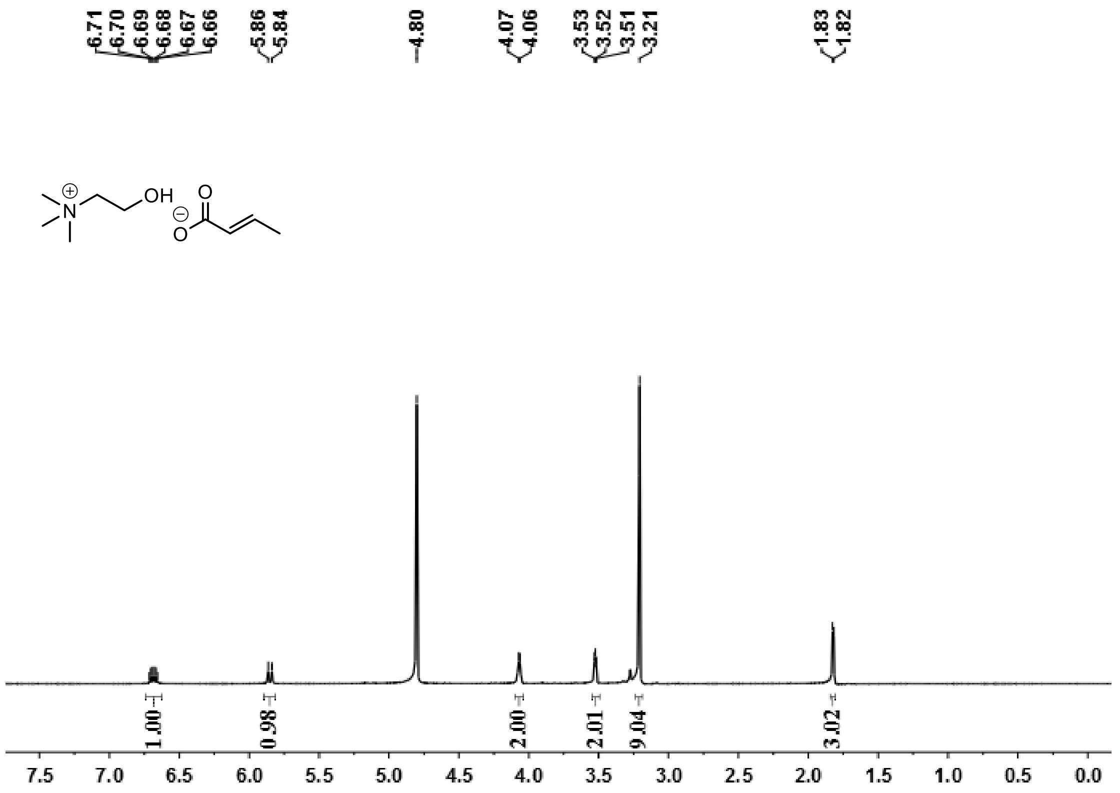
6.71
6.70
6.69
6.68
6.67
6.66
5.86
5.84
4.80
4.07
4.06
3.53
3.52
3.51
3.21
1.83
1.82
OH
O
O
1.00
0.98
2.00
2.01
9.04
3.02
7.5
7.0
6.5
6.0
5.5
5.0
4.5
4.0
3.5
3.0
2.5
2.0
1.5
1.0
0.5
0.0

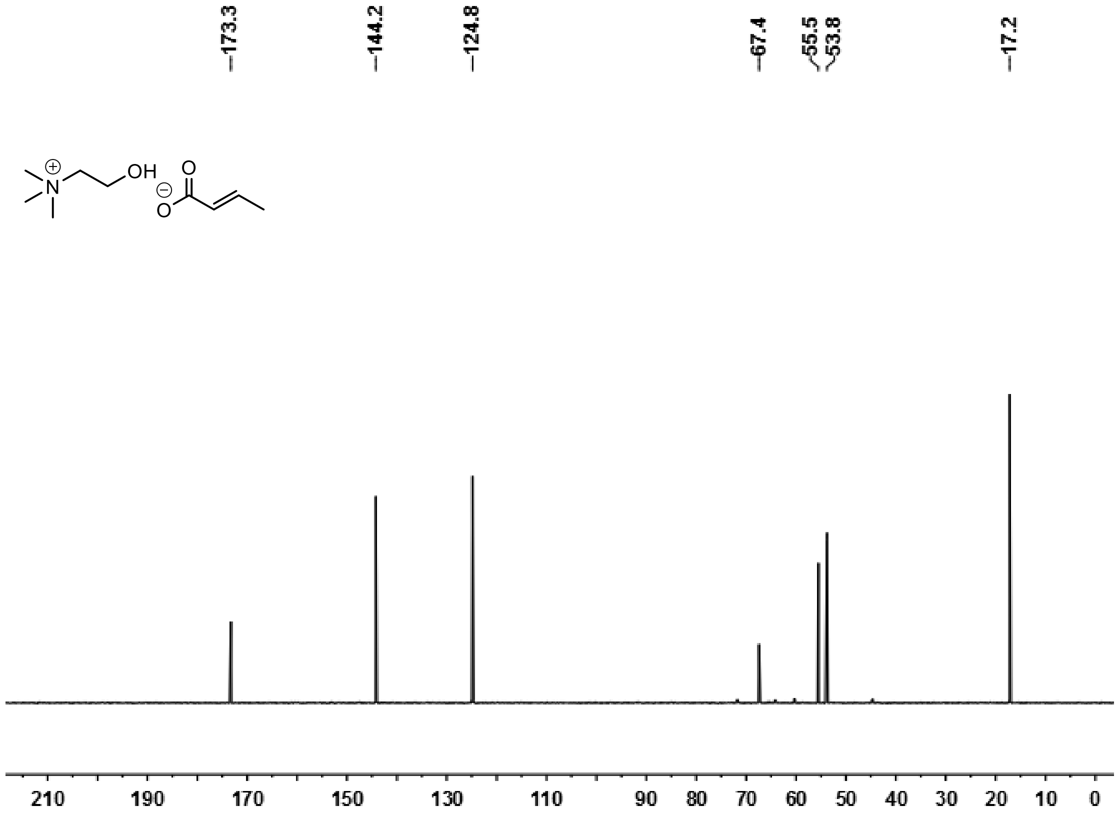
173.3
144.2
124.8
67.4
55.5
53.8
17.2
OH
O
O
210
190
170
150
130
110
90
80
70
60
50
40
30
20
10
0

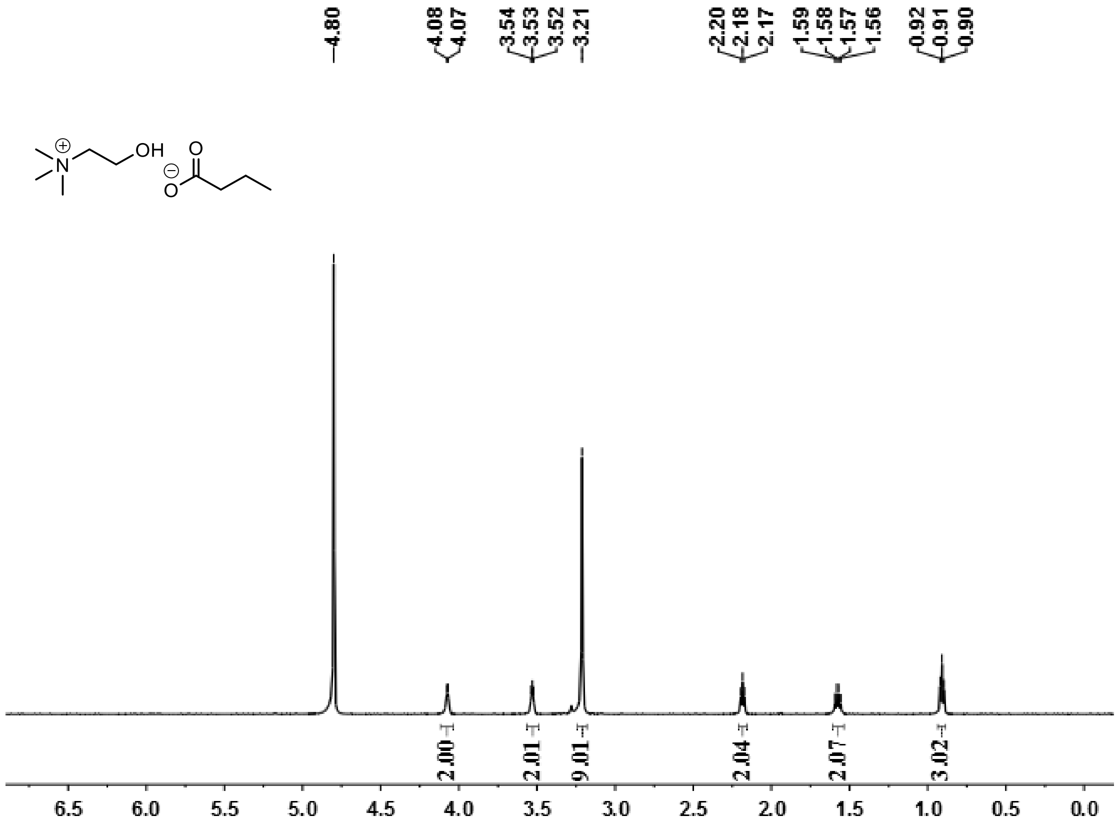
4.80
4.08
4.07
3.54
3.53
3.52
3.21
2.20
2.18
2.17
1.59
1.58
1.57
1.56
0.92
0.91
0.90
OH
O
N
O
2.00
2.01
9.01
2.04
2.07
3.02
6.5 6.0 5.5 5.0 4.5 4.0 3.5 3.0 2.5 2.0 1.5 1.0 0.5 0.0

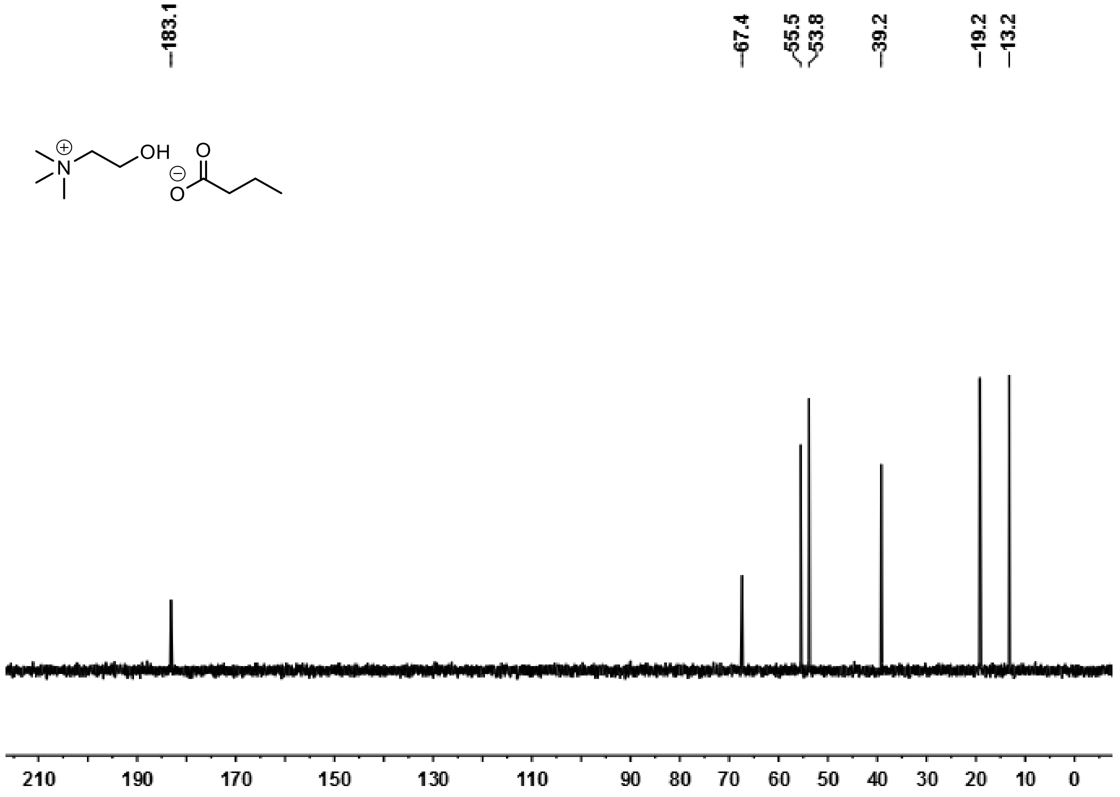
183.1
67.4
55.5
53.8
39.2
19.2
13.2
OH
O
N
O
210 190 170 150 130 110 90 80 70 60 50 40 30 20 10 0

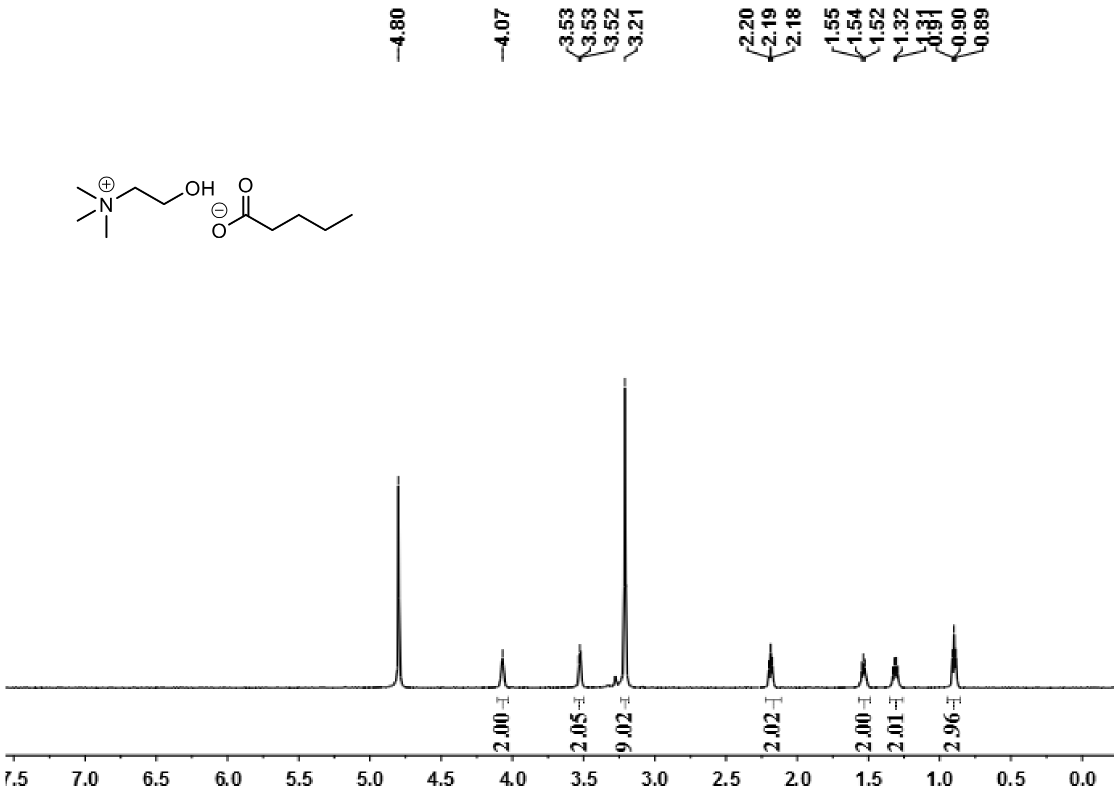
4.80
4.07
3.53
3.53
3.52
3.21
2.20
2.19
2.18
1.55
1.54
1.52
1.32
1.31
0.91
0.90
0.89
2.00
2.05
9.02
2.02
2.00
2.01
2.96
OH
O
N
O
7.5
7.0
6.5
6.0
5.5
5.0
4.5
4.0
3.5
3.0
2.5
2.0
1.5
1.0
0.5
0.0

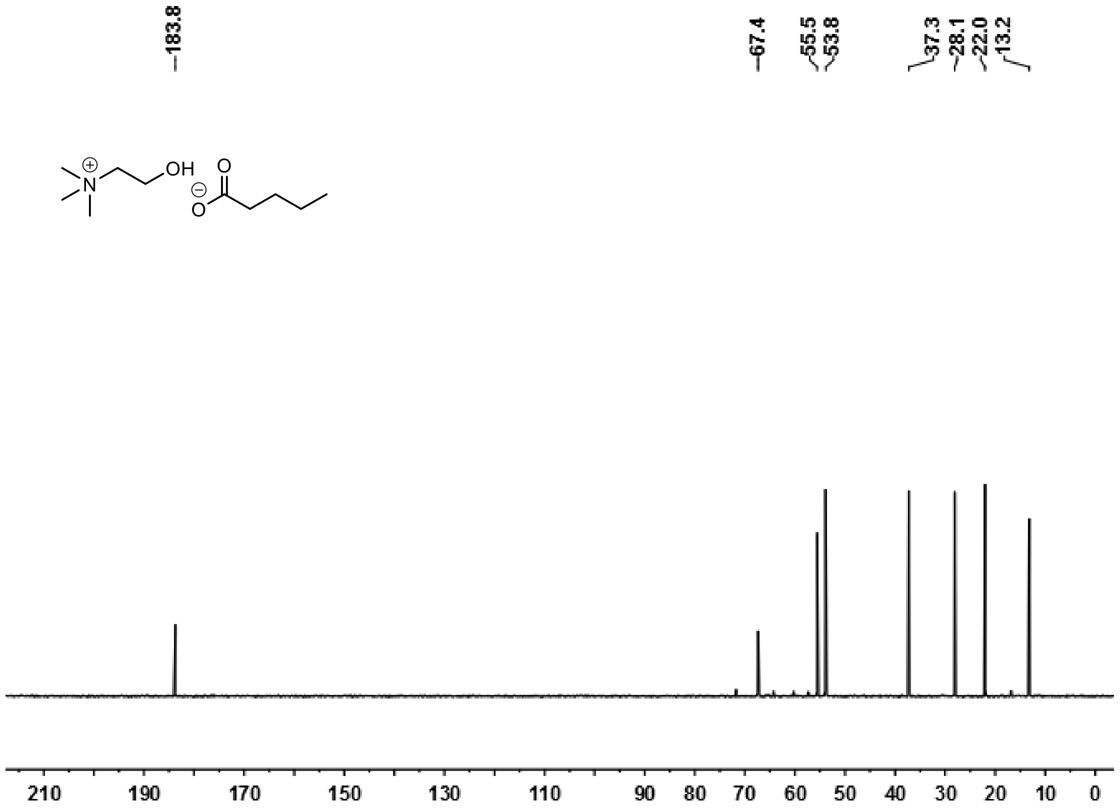
183.8
67.4
55.5
53.8
37.3
28.1
22.0
13.2
OH
O
N
O
210
190
170
150
130
110
90
80
70
60
50
40
30
20
10
0

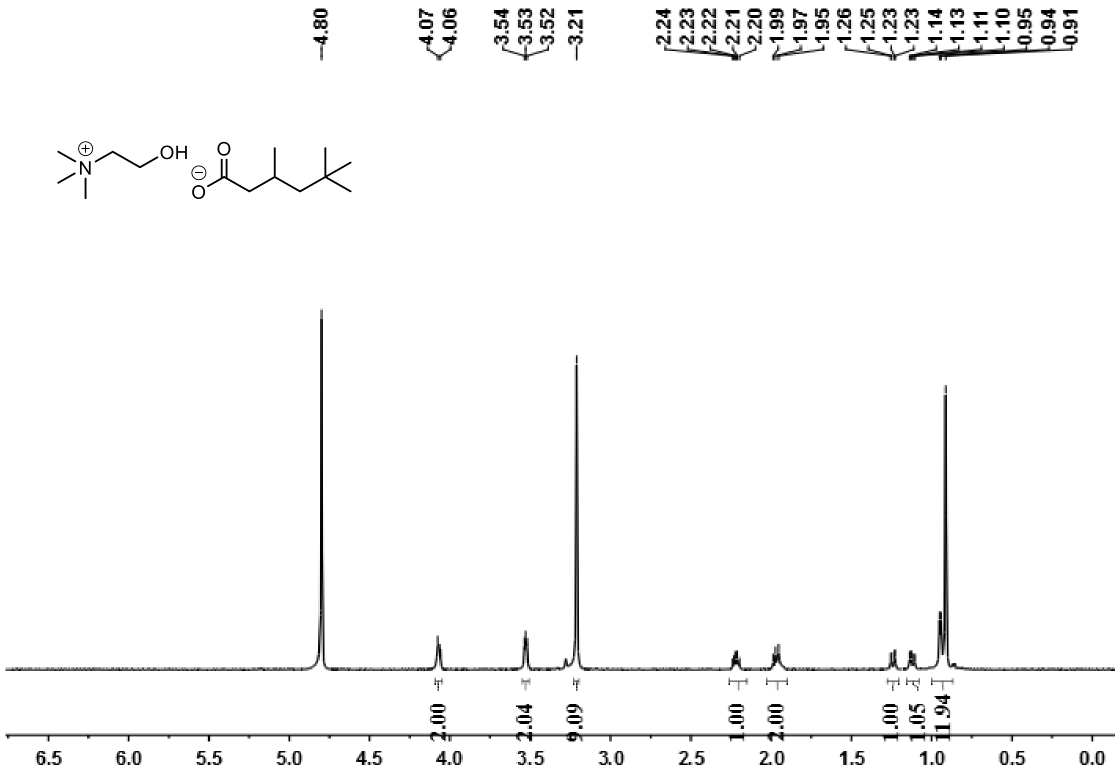
4.80
4.07 4.06
3.54 3.53 3.52
3.21
2.24 2.23 2.22 2.21 2.20 1.99 1.97 1.95 1.26 1.25 1.23 1.23 1.14 1.13 1.11 1.10 0.95 0.94 0.91
OH
O
O
N
2.00 2.04 9.09 1.00 2.00 1.00 1.05 11.94
6.5 6.0 5.5 5.0 4.5 4.0 3.5 3.0 2.5 2.0 1.5 1.0 0.5 0.0

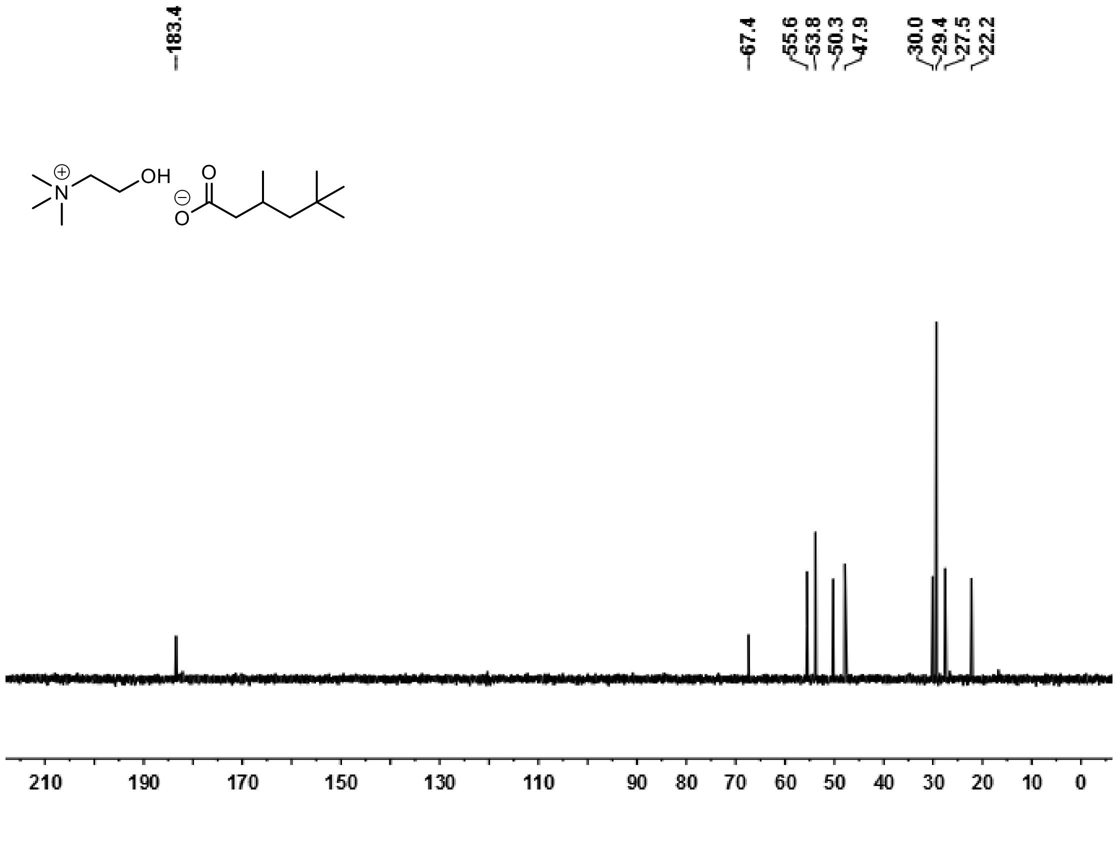
183.4
67.4
55.6 53.8 50.3 47.9
30.0 29.4 27.5 22.2
OH
O
O
N
210 190 170 150 130 110 90 80 70 60 50 40 30 20 10 0

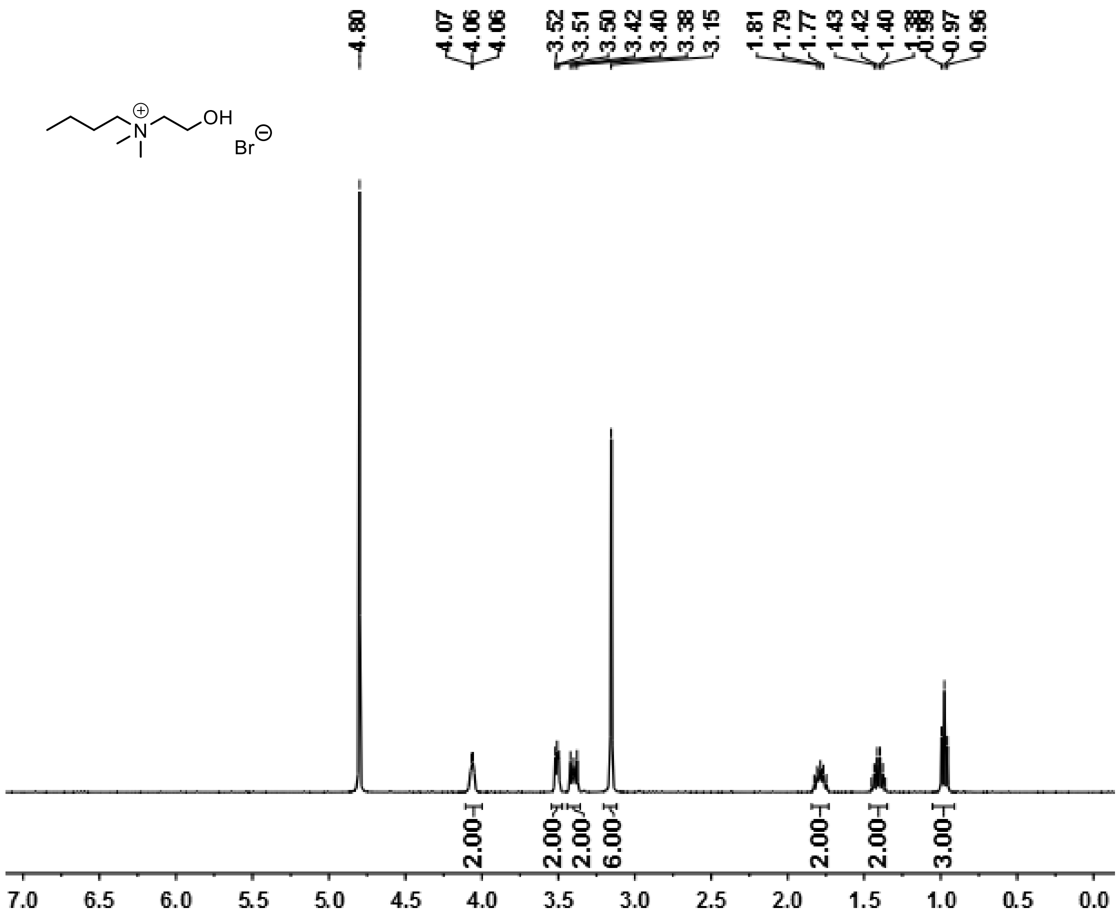
4.80
4.07 4.06 4.06
3.52 3.51 3.50 3.42 3.40 3.38 3.15
1.81 1.79 1.77 1.43 1.42 1.40 1.38
0.99 0.97 0.96
OH
Br
2.00 2.00 2.00 6.00 2.00 2.00 3.00
7.0 6.5 6.0 5.5 5.0 4.5 4.0 3.5 3.0 2.5 2.0 1.5 1.0 0.5 0.0

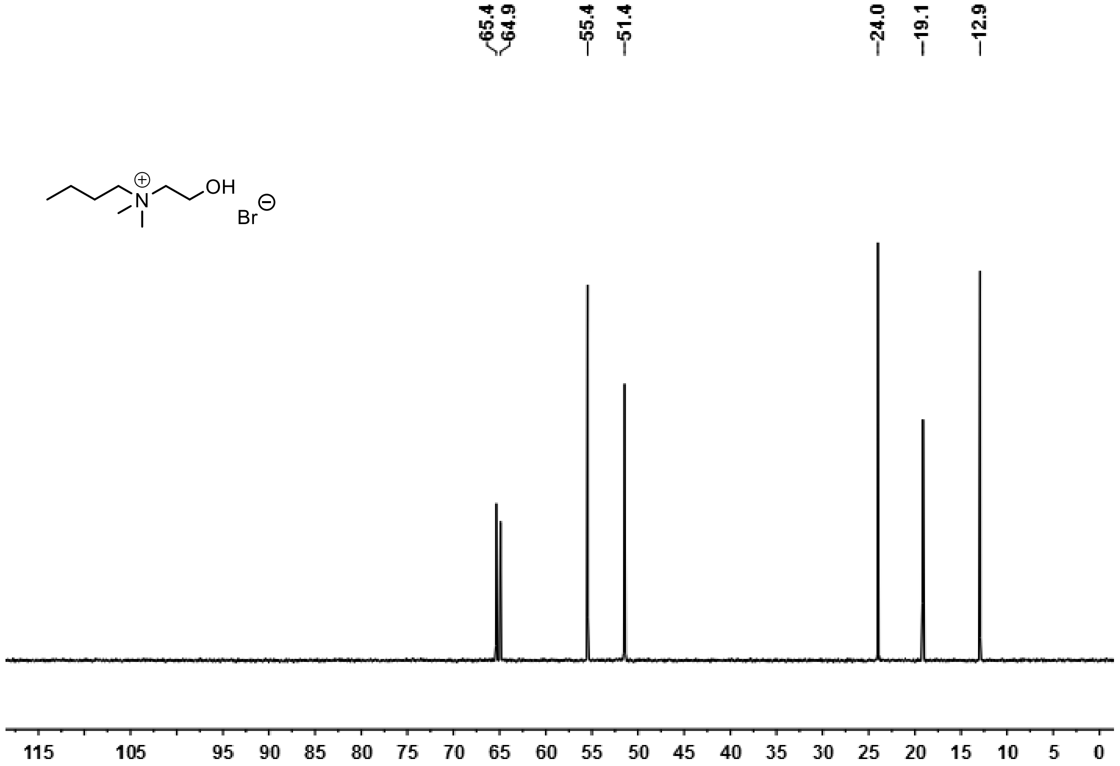
65.4 64.9 55.4 51.4 24.0 19.1 12.9
OH
Br
115 105 95 90 85 80 75 70 65 60 55 50 45 40 35 30 25 20 15 10 5 0

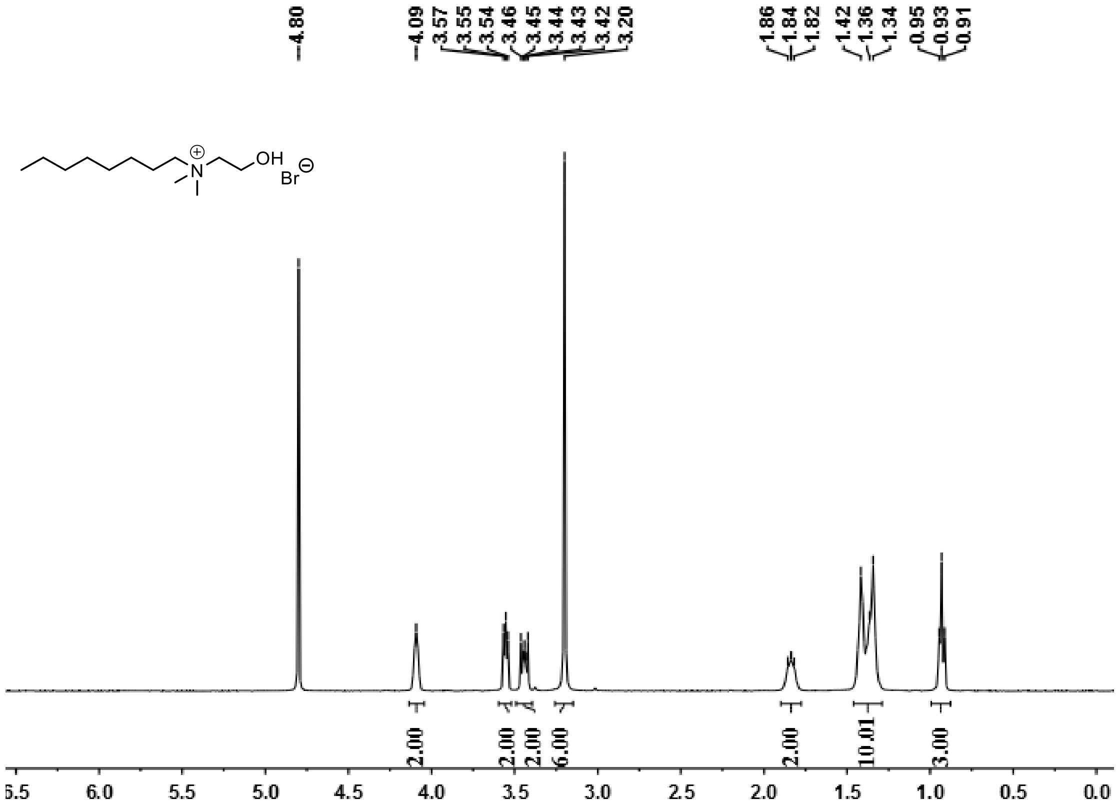

4.80
4.09
3.57
3.55
3.54
3.46
3.45
3.44
3.43
3.42
3.20
1.86
1.84
1.82
1.42
1.36
1.34
0.95
0.93
0.91
OH
N
Br
2.00
2.00
2.00
6.00
2.00
10.01
3.00
6.0
5.5
5.0
4.5
4.0
3.5
3.0
2.5
2.0
1.5
1.0
0.5
0.0

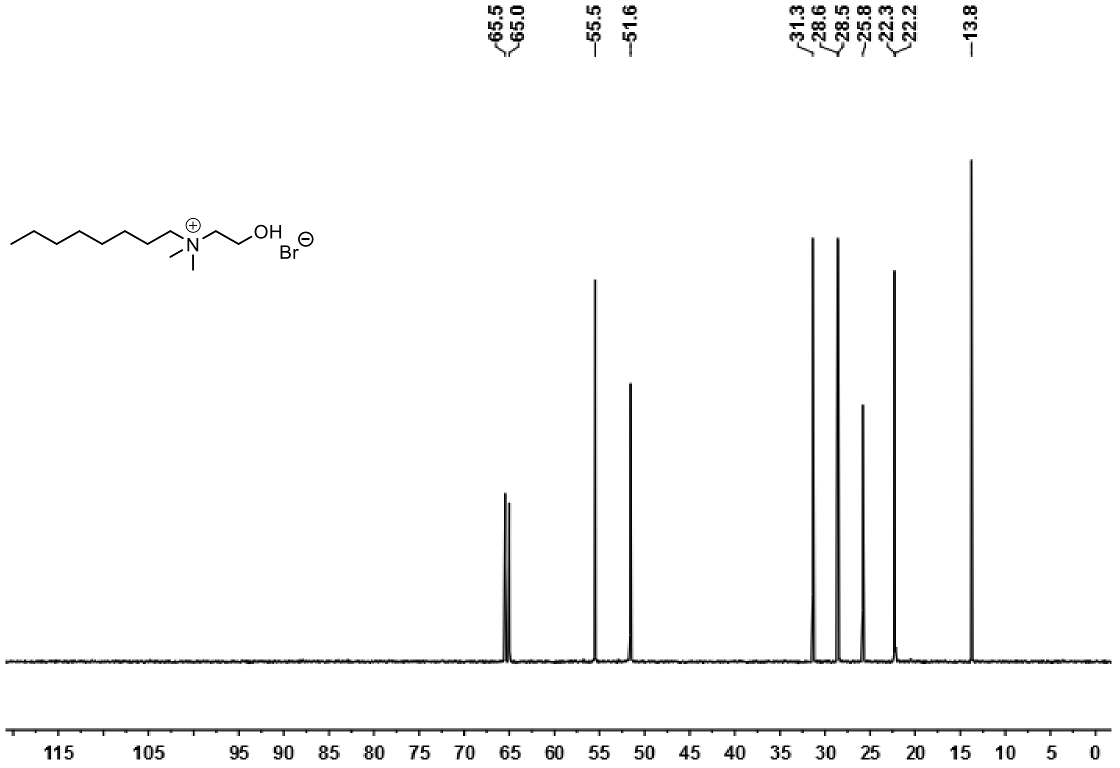

65.5
65.0
55.5
51.6
31.3
28.6
28.5
25.8
22.3
22.2
13.8
OH
N
Br
115
105
95
90
85
80
75
70
65
60
55
50
45
40
35
30
25
20
15
10
5
0

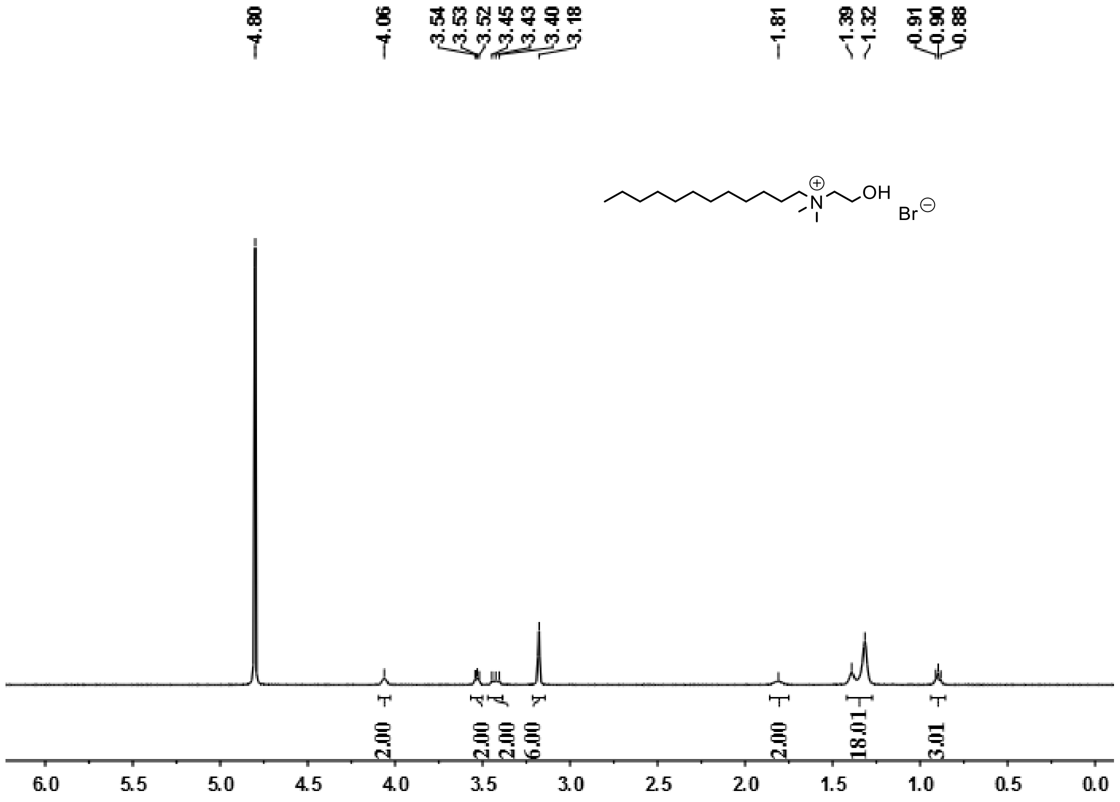
4.80
4.06
3.54
3.53
3.52
3.45
3.43
3.40
3.18
1.81
1.39
1.32
0.91
0.90
0.88
OH
Br
2.00
2.00
2.00
6.00
2.00
18.01
3.01
6.0
5.5
5.0
4.5
4.0
3.5
3.0
2.5
2.0
1.5
1.0
0.5
0.0

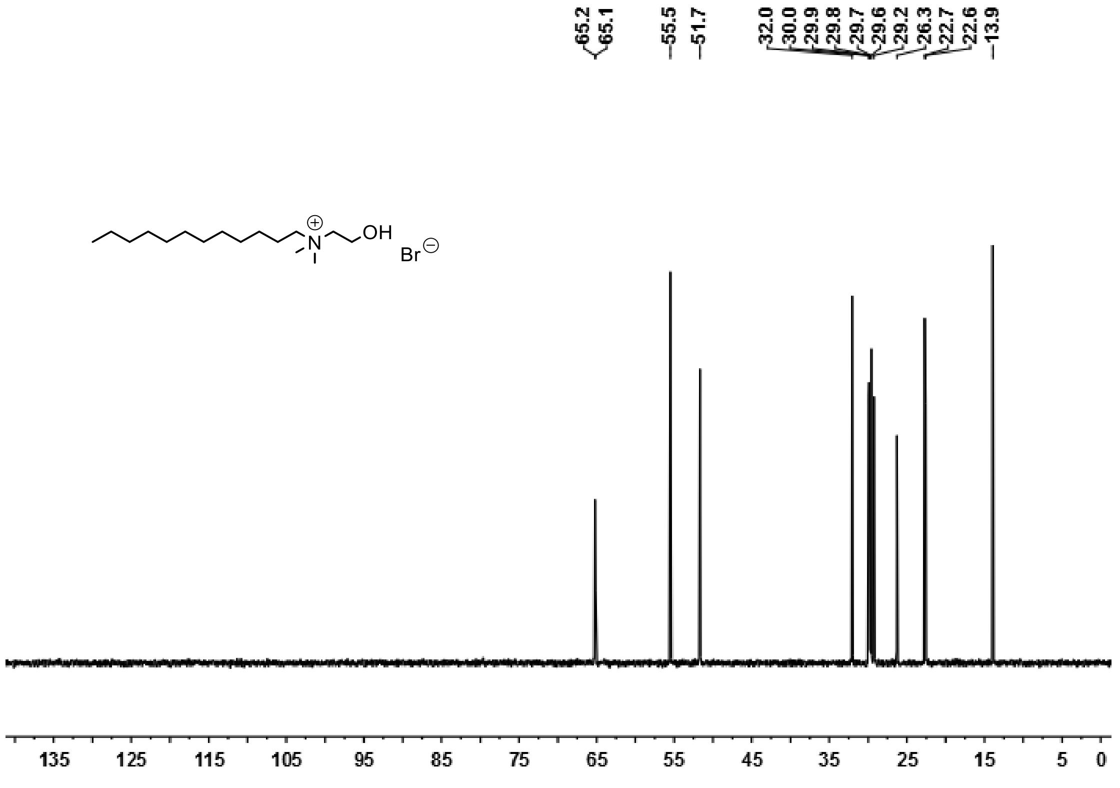
65.2
65.1
55.5
51.7
32.0
30.0
29.9
29.8
29.7
29.6
29.2
26.3
22.7
22.6
13.9
OH
Br
135
125
115
105
95
85
75
65
55
45
35
25
15
5
0

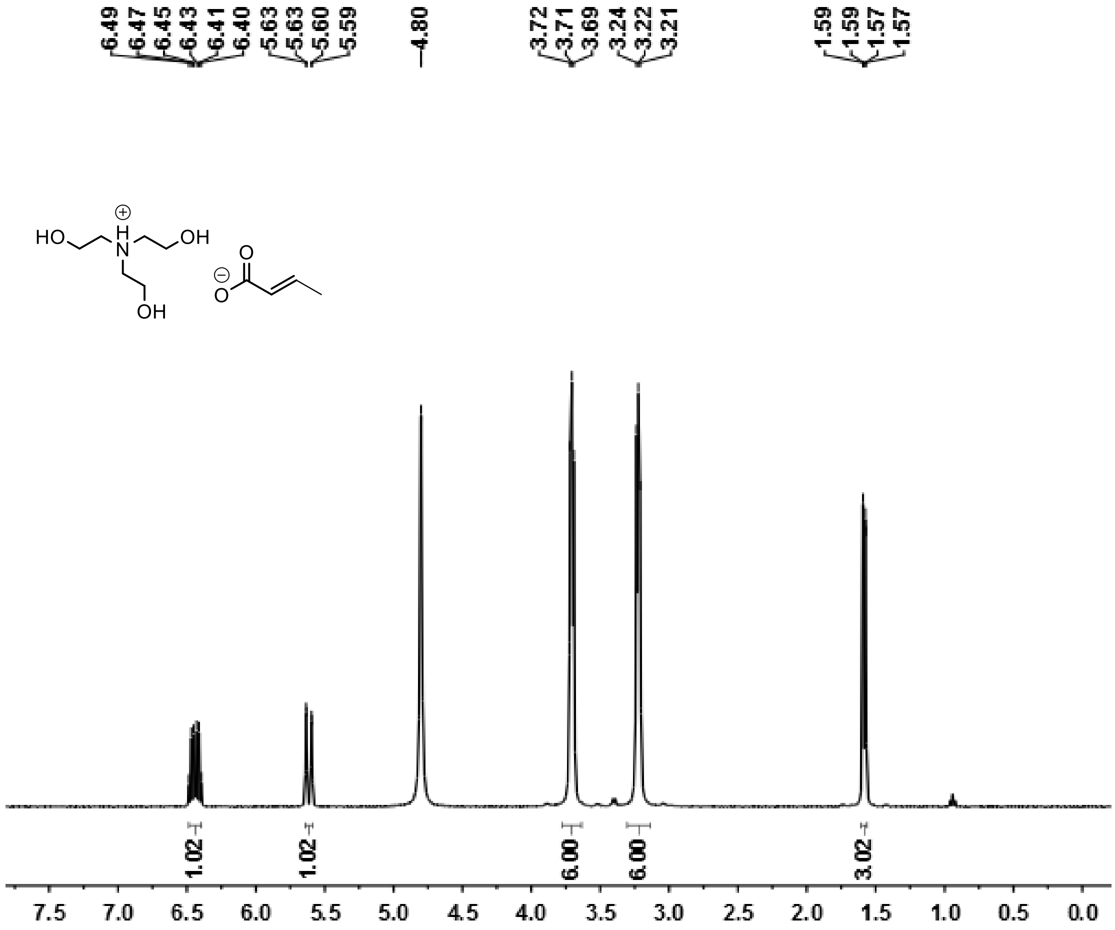
6.49 6.47 6.45 6.43 6.41 6.40 5.63 5.63 5.60 5.59
4.80
3.72 3.71 3.69 3.24 3.22 3.21
1.59 1.59 1.57 1.57
HO OH N H ⊕ OH
⊖ O O
1.02 1.02 6.00 6.00 3.02
7.5 7.0 6.5 6.0 5.5 5.0 4.5 4.0 3.5 3.0 2.5 2.0 1.5 1.0 0.5 0.0

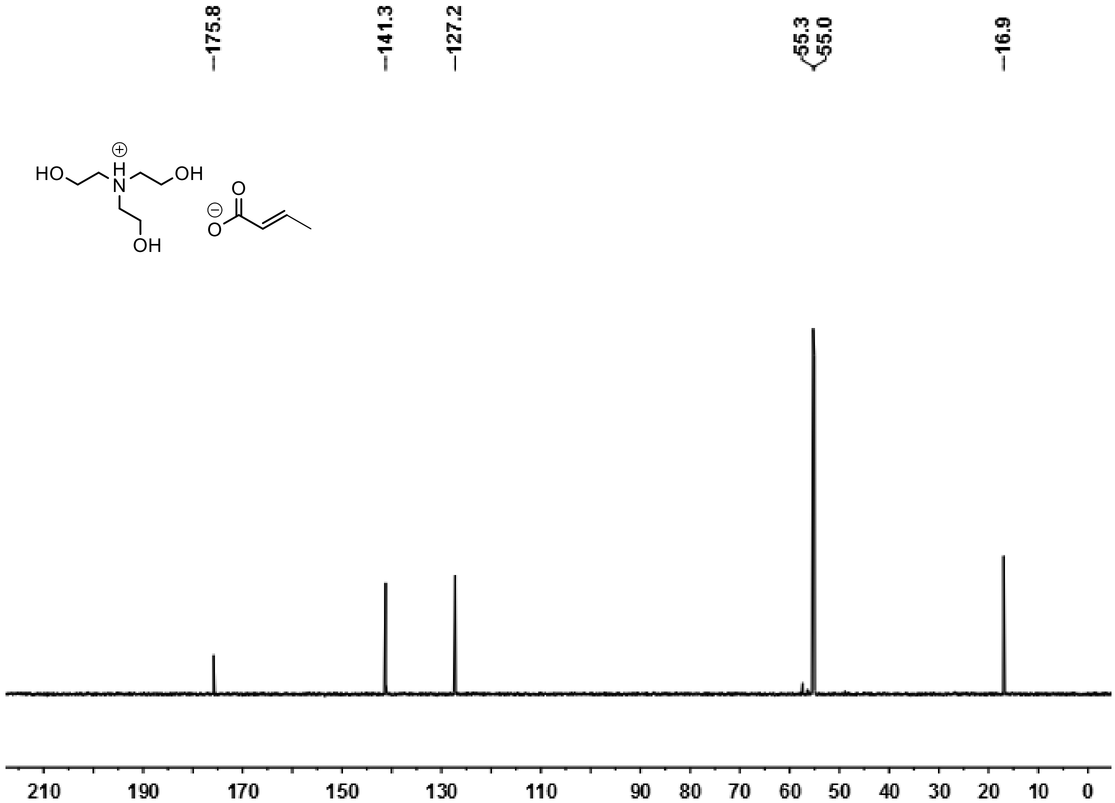
175.8
141.3
127.2
55.3 55.0
16.9
HO OH N H ⊕ OH
⊖ O O
210 190 170 150 130 110 90 80 70 60 50 40 30 20 10 0

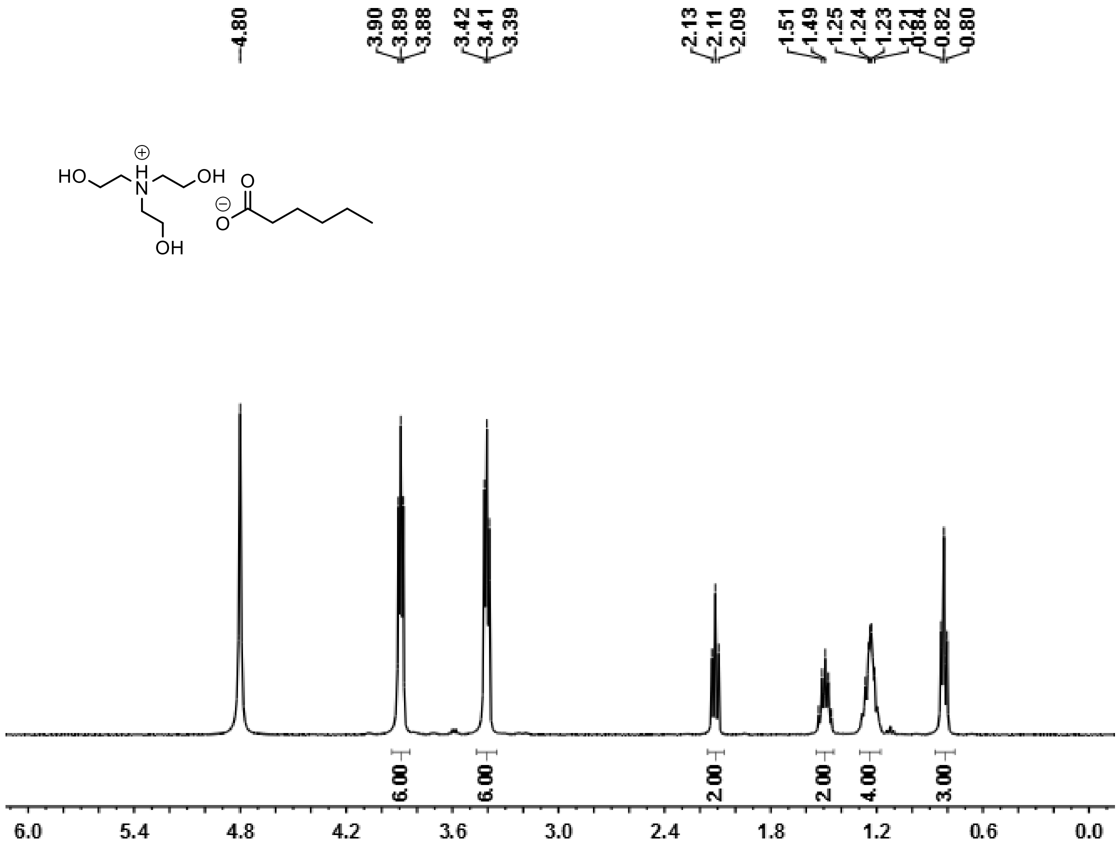
4.80
3.90
3.89
3.88
3.42
3.41
3.39
2.13
2.11
2.09
1.51
1.49
1.25
1.24
1.23
1.21
0.84
0.82
0.80
HO
OH
OH
6.00
6.00
2.00
2.00
4.00
3.00
6.0 5.4 4.8 4.2 3.6 3.0 2.4 1.8 1.2 0.6 0.0

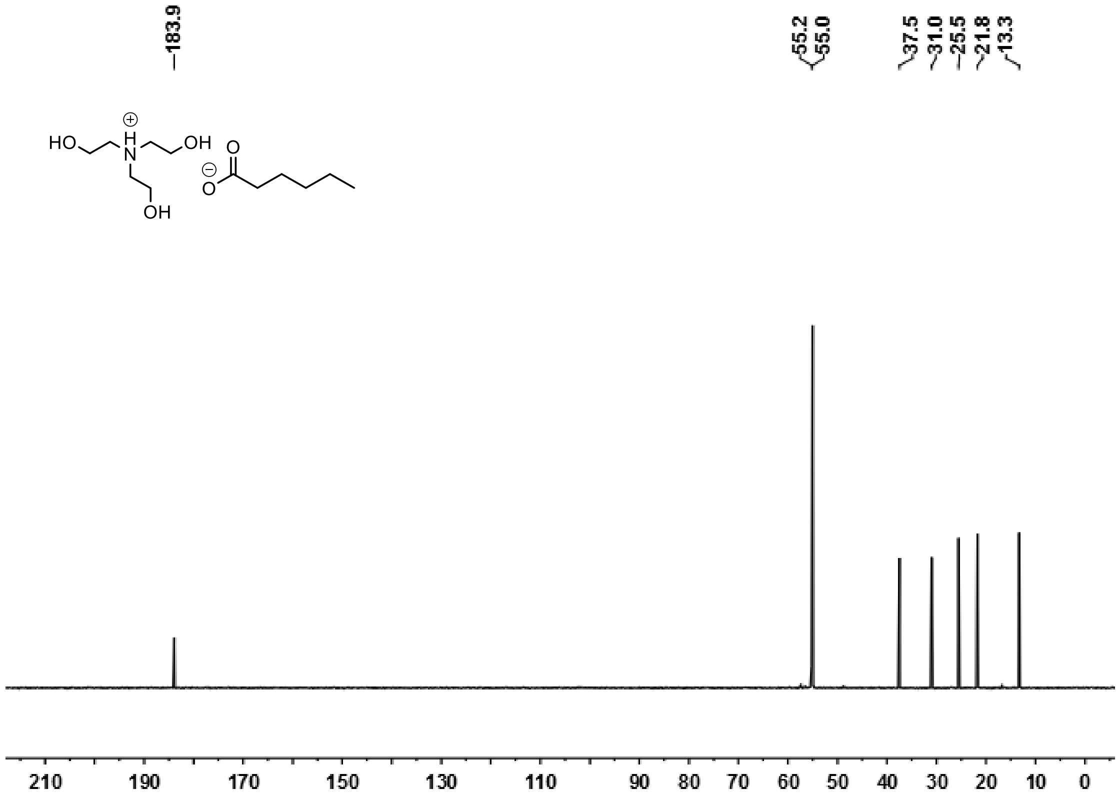
183.9
55.2
55.0
37.5
31.0
25.5
21.8
13.3
HO
OH
OH
210 190 170 150 130 110 90 80 70 60 50 40 30 20 10 0

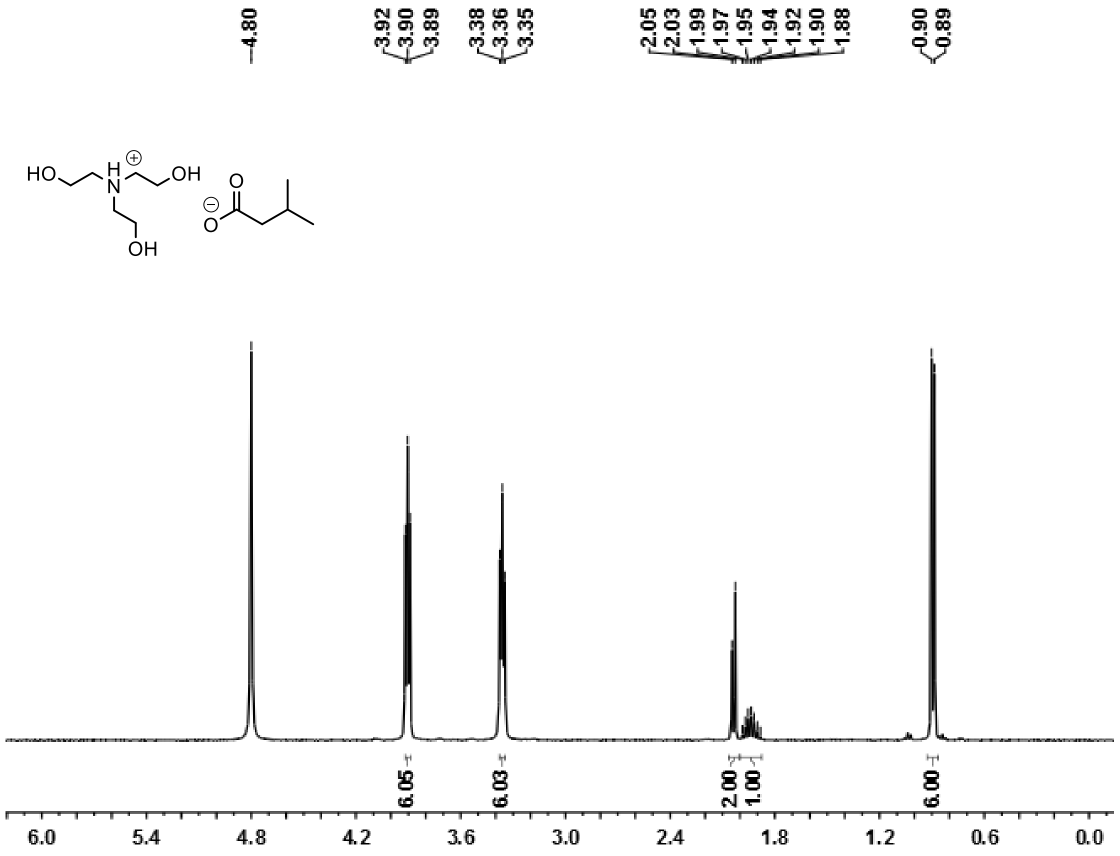

4.80
3.92
3.90
3.89
3.38
3.36
3.35
2.05
2.03
1.99
1.97
1.95
1.94
1.92
1.90
1.88
0.90
0.89
HO
OH
OH
N
H
O
O
6.05
6.03
2.00
1.00
6.00
6.0
5.4
4.8
4.2
3.6
3.0
2.4
1.8
1.2
0.6
0.0

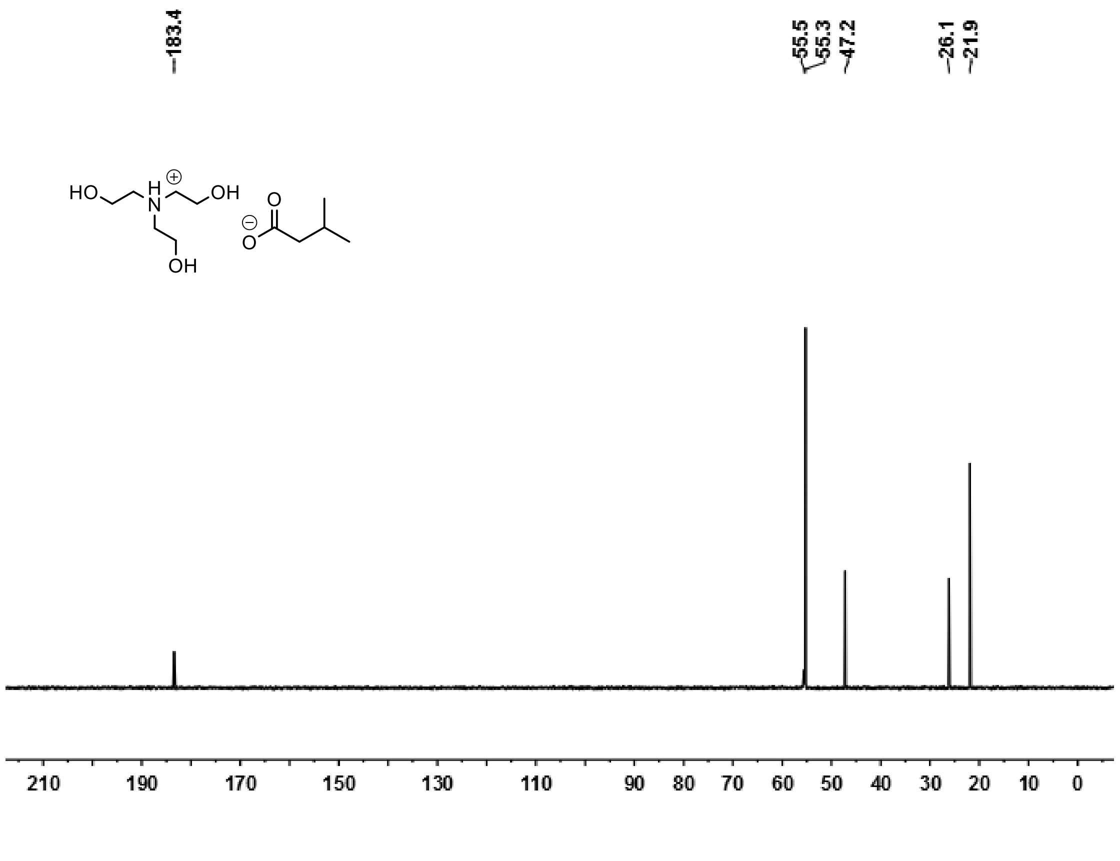

183.4
55.5
55.3
47.2
26.1
21.9
HO
OH
OH
N
H
O
O
210
190
170
150
130
110
90
80
70
60
50
40
30
20
10
0

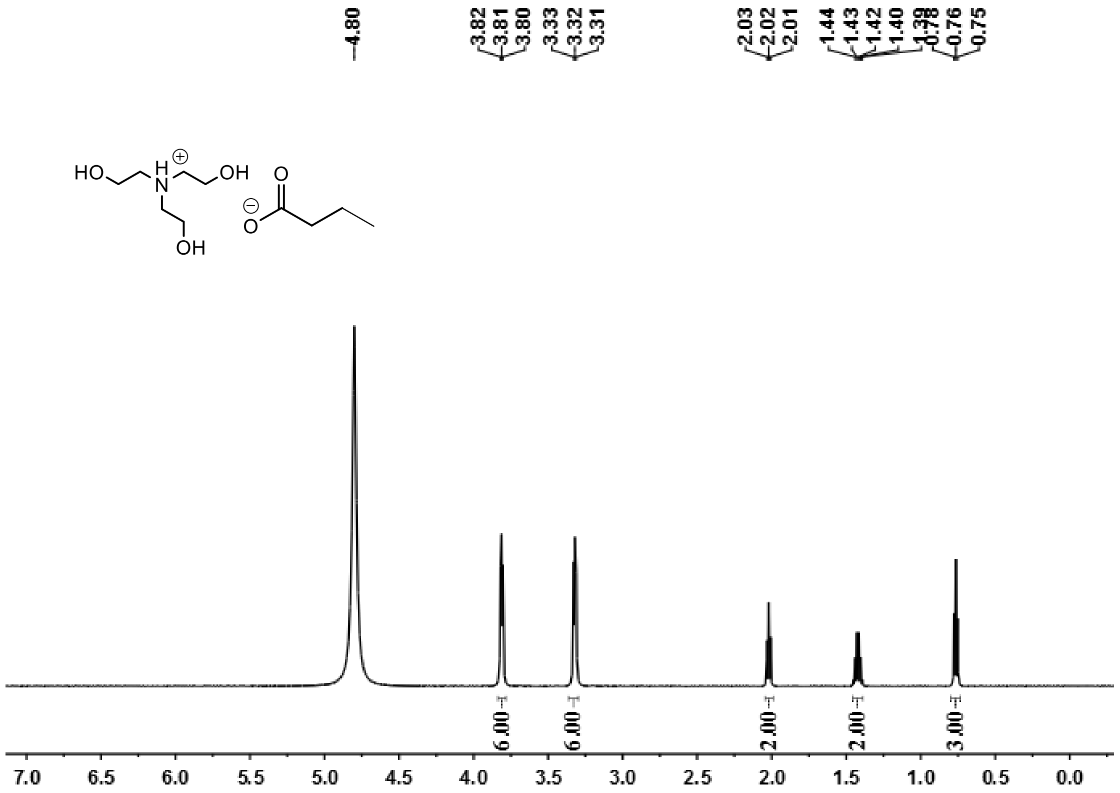

4.80
3.82
3.81
3.80
3.33
3.32
3.31
2.03
2.02
2.01
1.44
1.43
1.42
1.40
1.39
0.78
0.76
0.75
HO
OH
OH
O
6.00
6.00
2.00
2.00
3.00
7.0
6.5
6.0
5.5
5.0
4.5
4.0
3.5
3.0
2.5
2.0
1.5
1.0
0.5
0.0

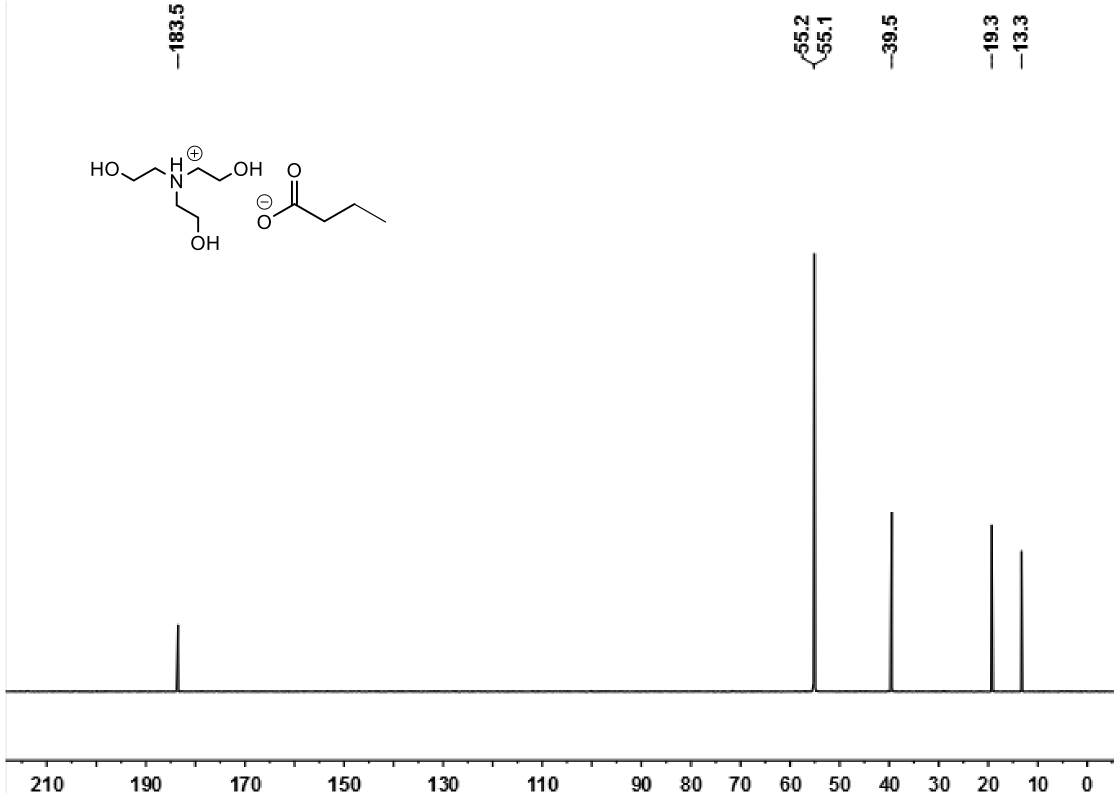

183.5
55.2
55.1
39.5
19.3
13.3
HO
OH
OH
O
210
190
170
150
130
110
90
80
70
60
50
40
30
20
10
0

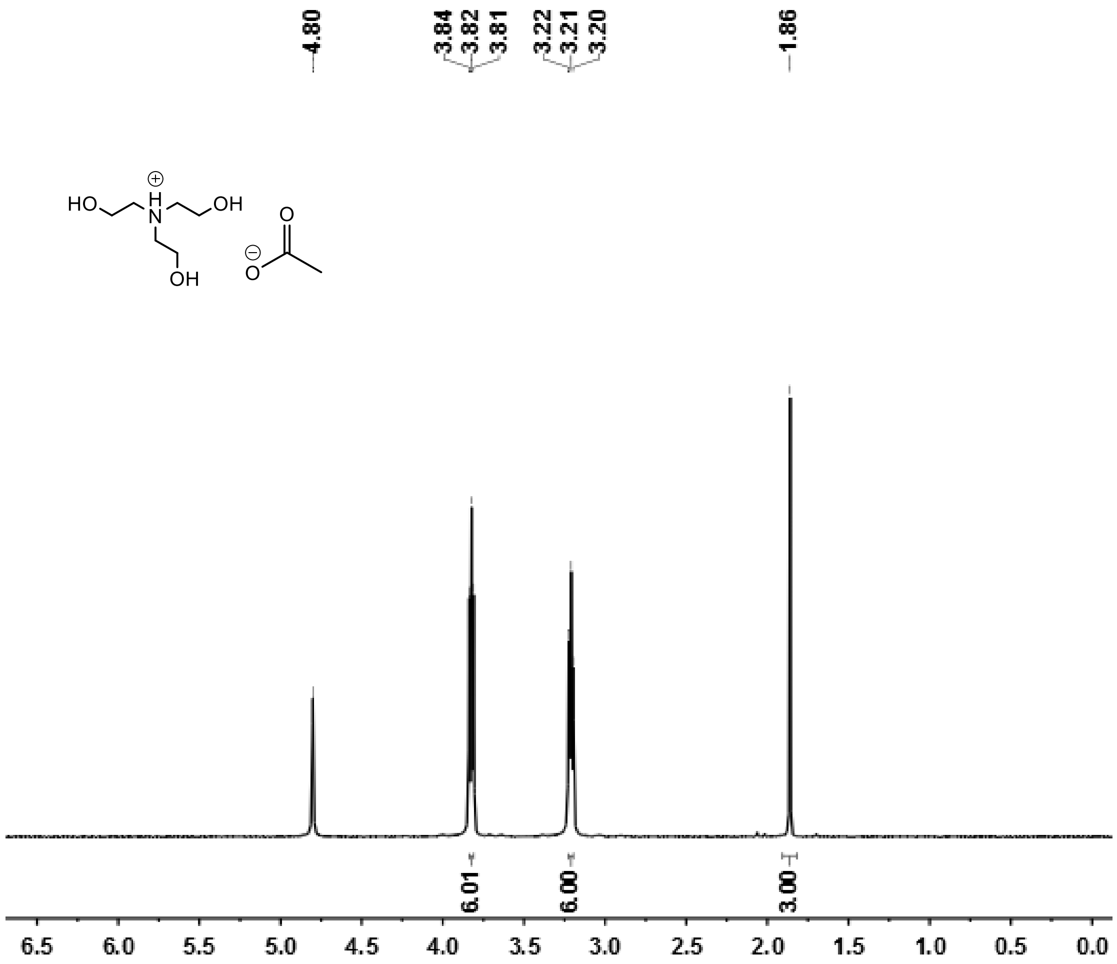
4.80
3.84
3.82
3.81
3.22
3.21
3.20
1.86
HO
OH
OH
O
O
6.01
6.00
3.00
6.5 6.0 5.5 5.0 4.5 4.0 3.5 3.0 2.5 2.0 1.5 1.0 0.5 0.0

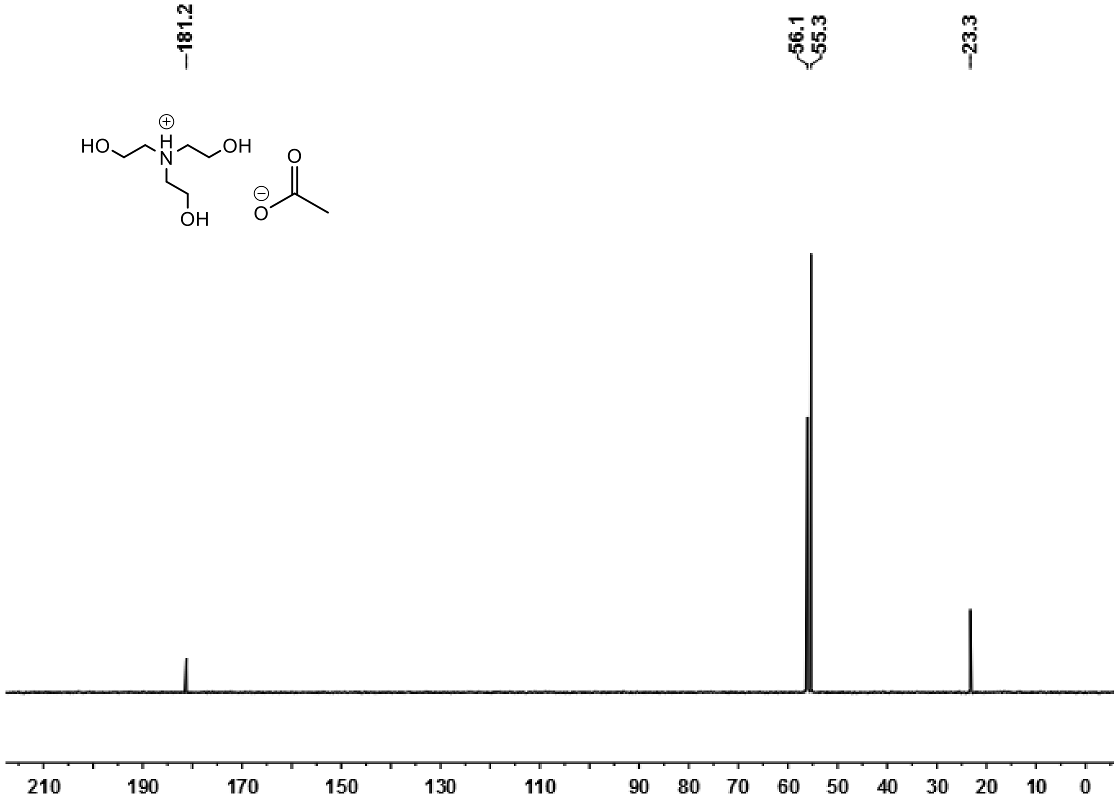
181.2
56.1
55.3
23.3
HO
OH
OH
O
O
210 190 170 150 130 110 90 80 70 60 50 40 30 20 10 0

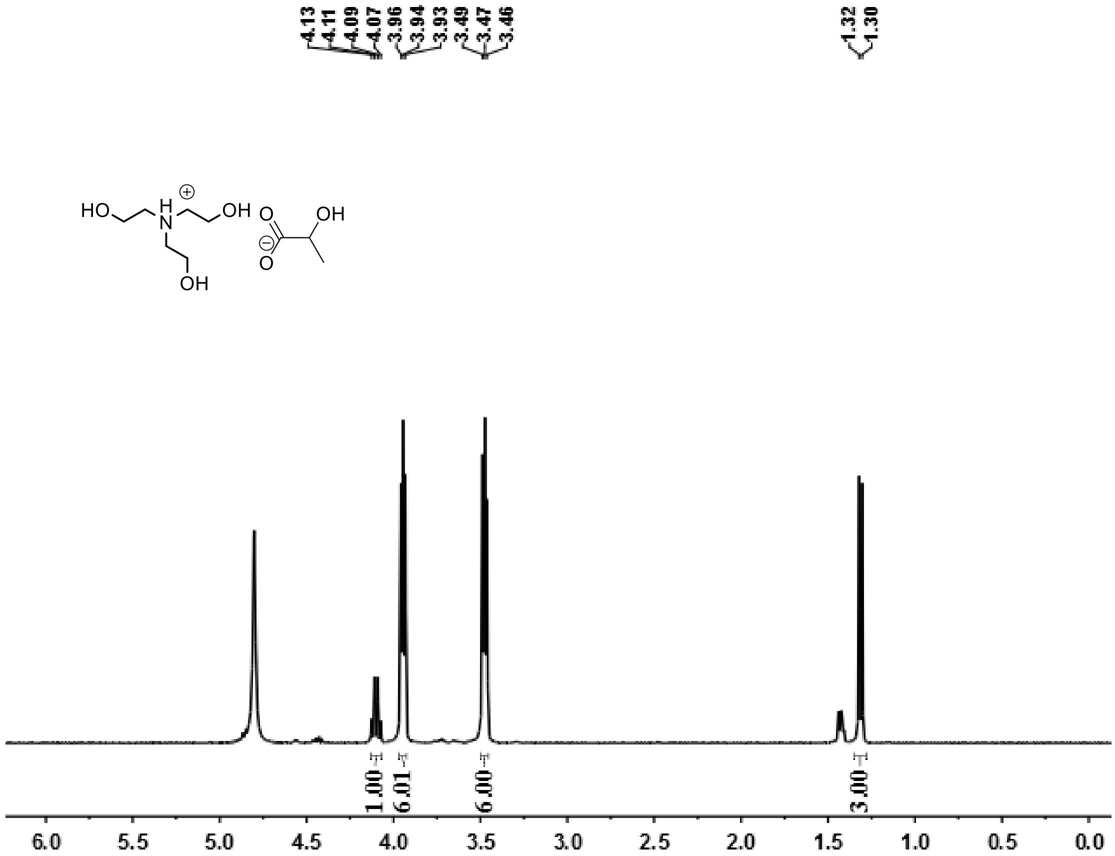
4.13
4.11
4.09
4.07
3.96
3.94
3.93
3.49
3.47
3.46
1.32
1.30
HO
H
N
OH
OH
O
OH
O
1.00
6.01
6.00
3.00
6.0
5.5
5.0
4.5
4.0
3.5
3.0
2.5
2.0
1.5
1.0
0.5
0.0

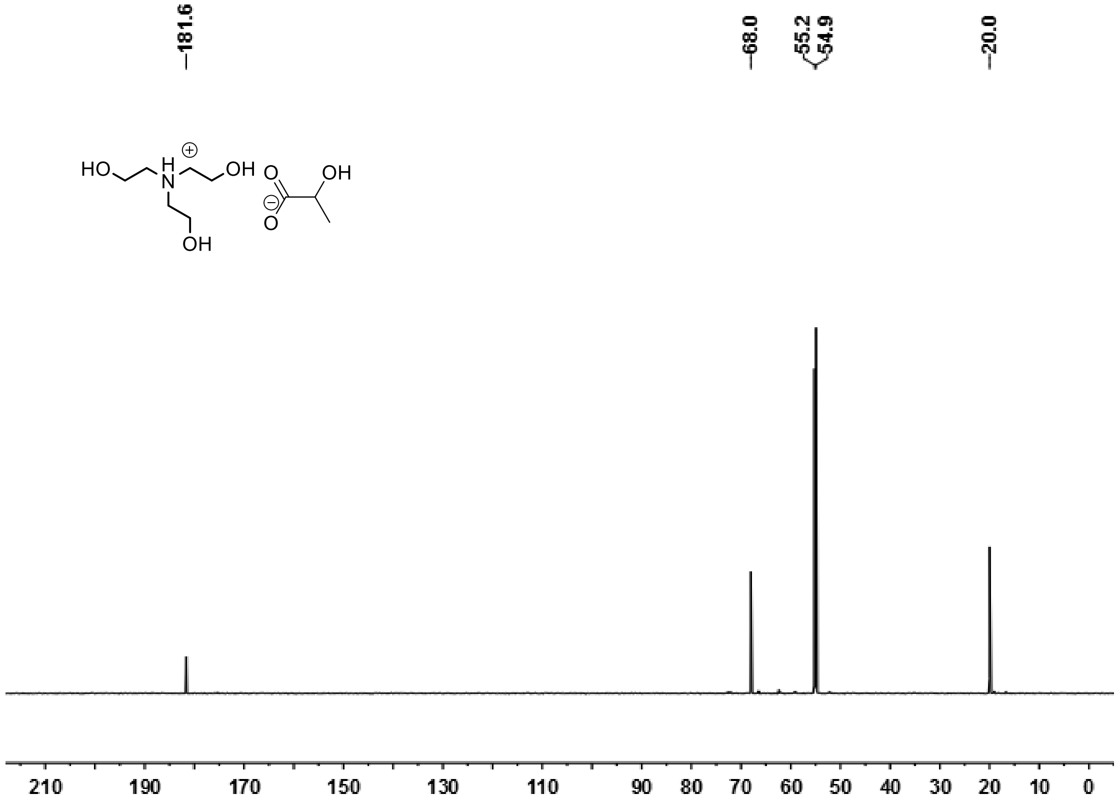
181.6
68.0
55.2
54.9
20.0
HO
H
N
OH
OH
O
OH
O
210
190
170
150
130
110
90
80
70
60
50
40
30
20
10
0

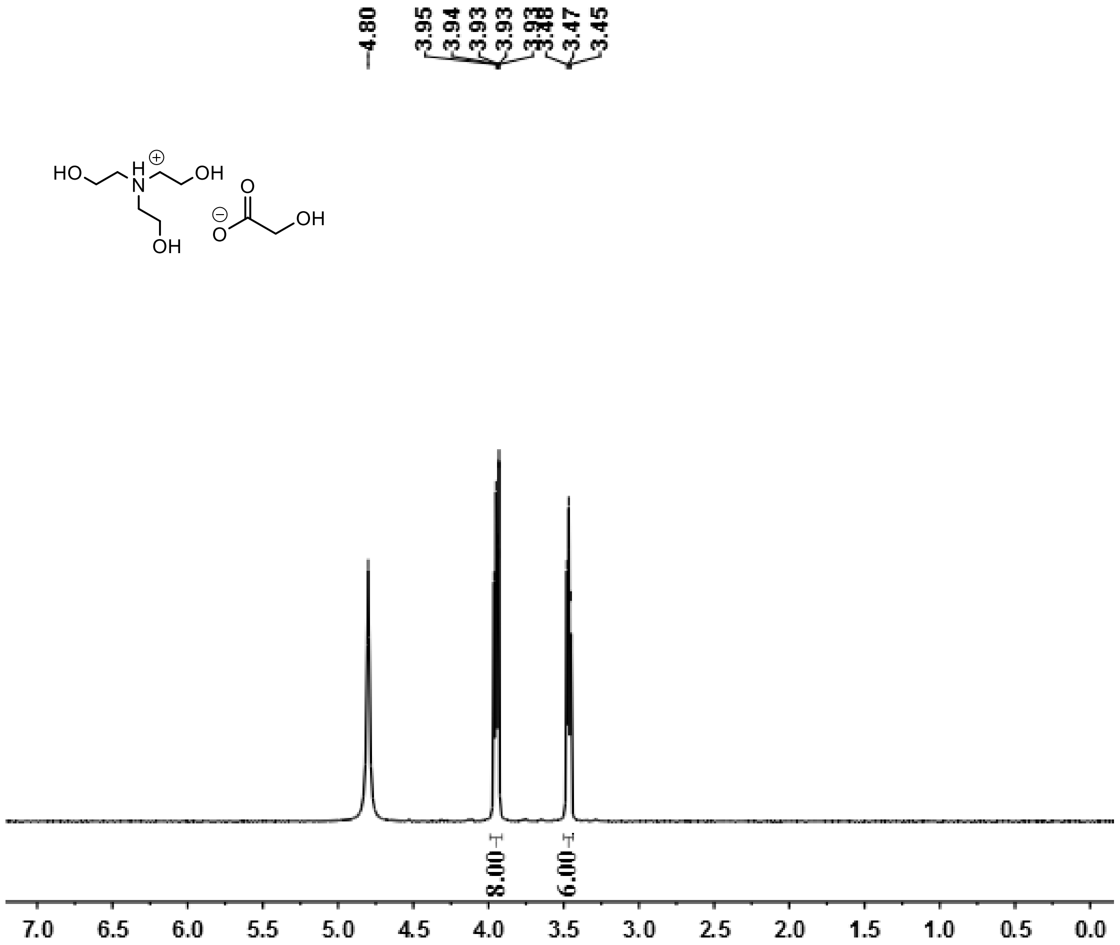
4.80
3.95
3.94
3.93
3.93
3.93
3.48
3.47
3.45
HO
H
N
OH
O
O
OH
OH
8.00
6.00
7.0 6.5 6.0 5.5 5.0 4.5 4.0 3.5 3.0 2.5 2.0 1.5 1.0 0.5 0.0

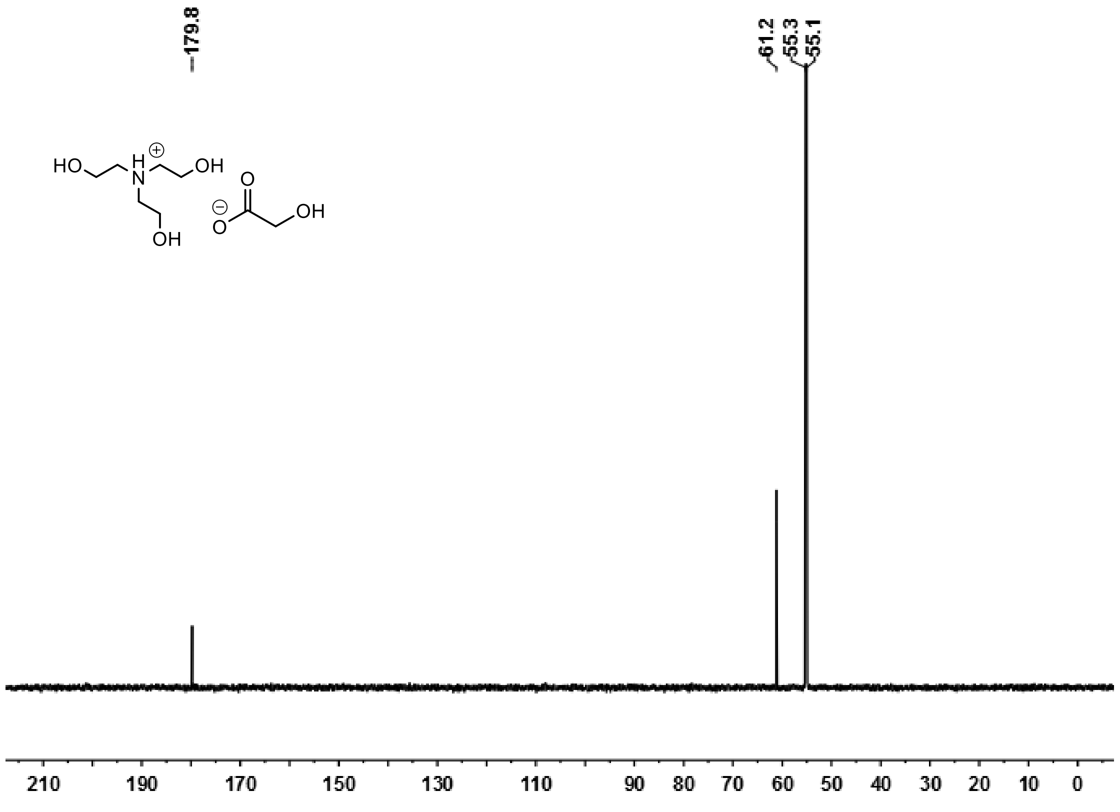
179.8
61.2
55.3
55.1
HO
H
N
OH
O
O
OH
OH
210 190 170 150 130 110 90 80 70 60 50 40 30 20 10 0

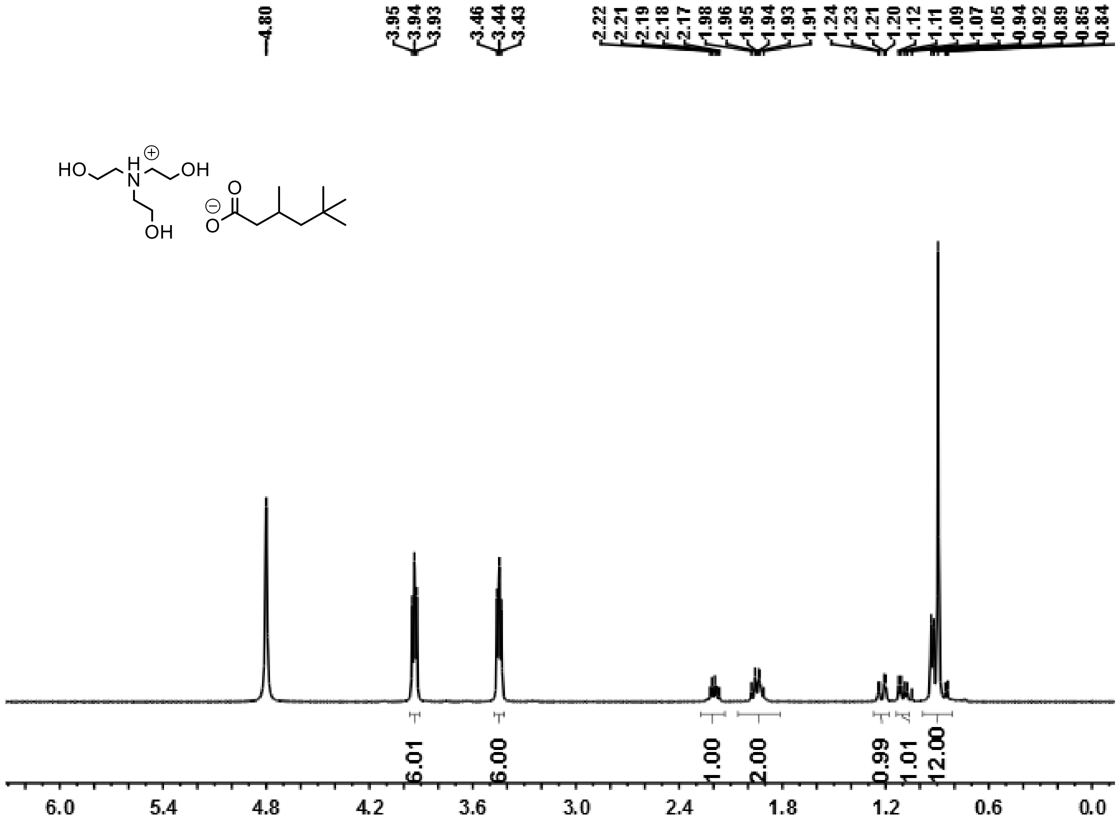
4.80
3.95
3.94
3.93
3.46
3.44
3.43
2.22
2.21
2.19
2.18
2.17
1.98
1.96
1.95
1.94
1.93
1.91
1.24
1.23
1.21
1.20
1.12
1.11
1.09
1.07
1.05
0.94
0.92
0.89
0.85
0.84
HO
OH
OH
O
O
6.01
6.00
1.00
2.00
0.99
1.01
12.00
6.0
5.4
4.8
4.2
3.6
3.0
2.4
1.8
1.2
0.6
0.0

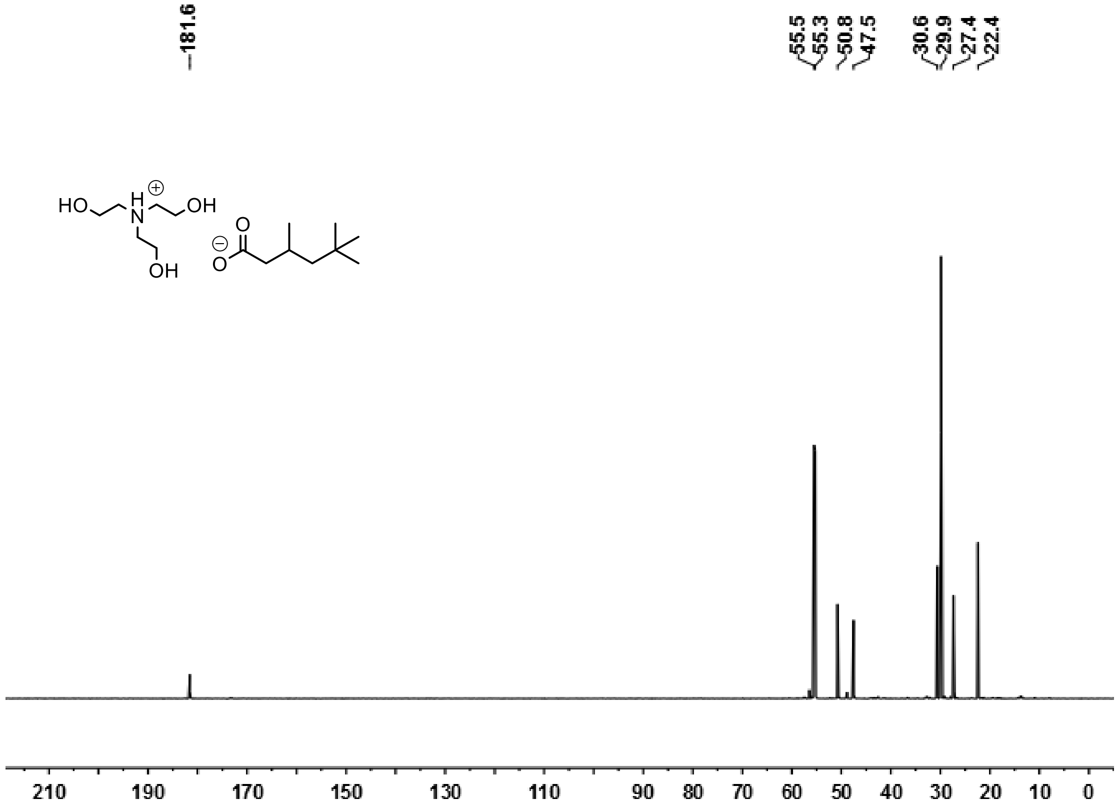
181.6
55.5
55.3
50.8
47.5
30.6
29.9
27.4
22.4
HO
OH
OH
O
O
210
190
170
150
130
110
90
80
70
60
50
40
30
20
10
0

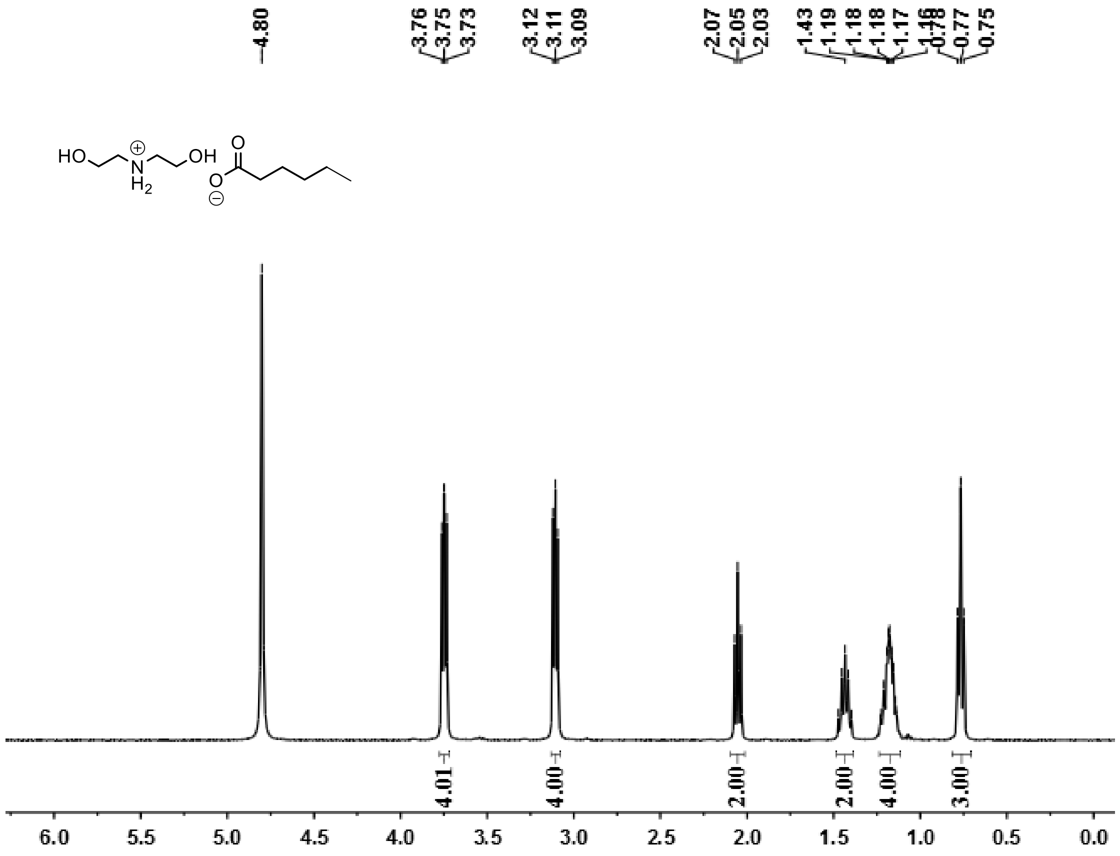
4.80
3.76
3.75
3.73
3.12
3.11
3.09
2.07
2.05
2.03
1.43
1.19
1.18
1.18
1.17
1.16
0.78
0.77
0.75
HO
N
H2
OH
O
O
4.01
4.00
2.00
2.00
4.00
3.00
6.0
5.5
5.0
4.5
4.0
3.5
3.0
2.5
2.0
1.5
1.0
0.5
0.0

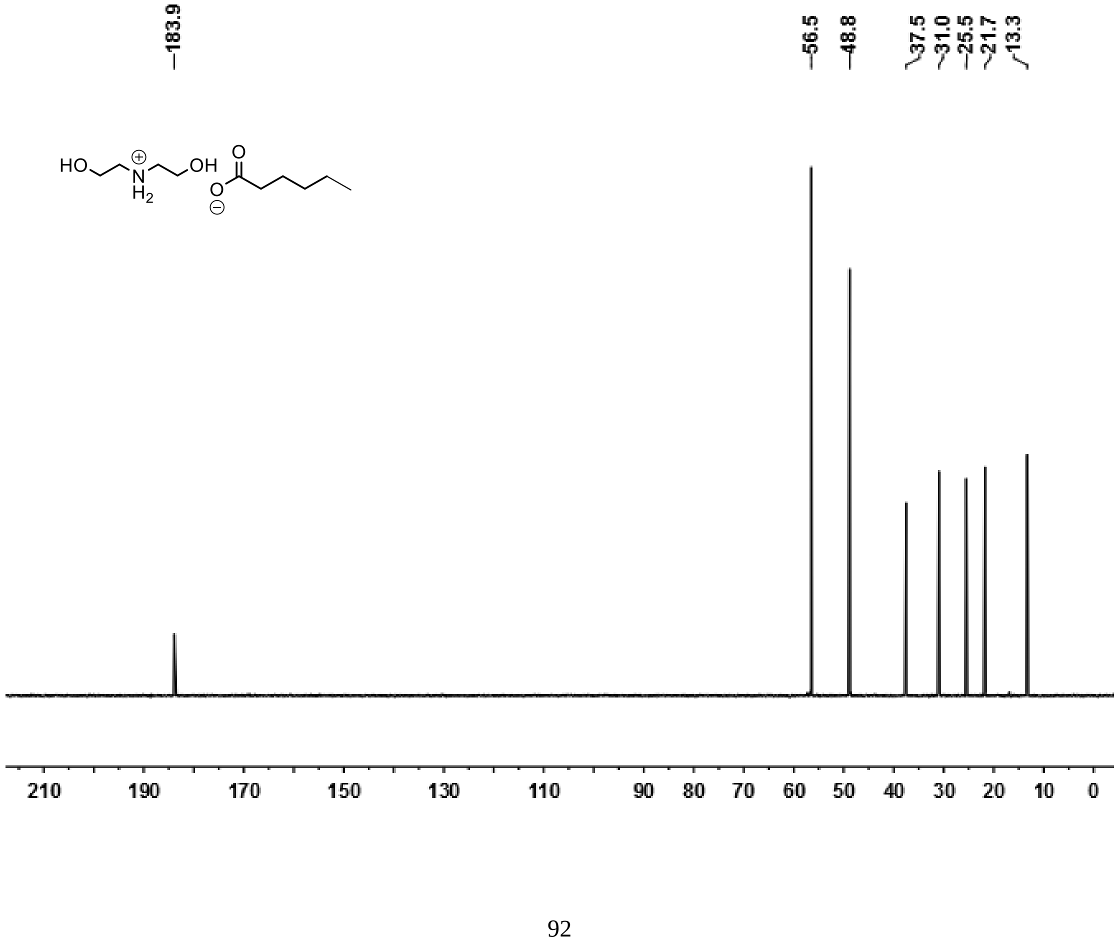
183.9
56.5
48.8
37.5
31.0
25.5
21.7
13.3
HO
N
H2
OH
O
O
210
190
170
150
130
110
90
80
70
60
50
40
30
20
10
0

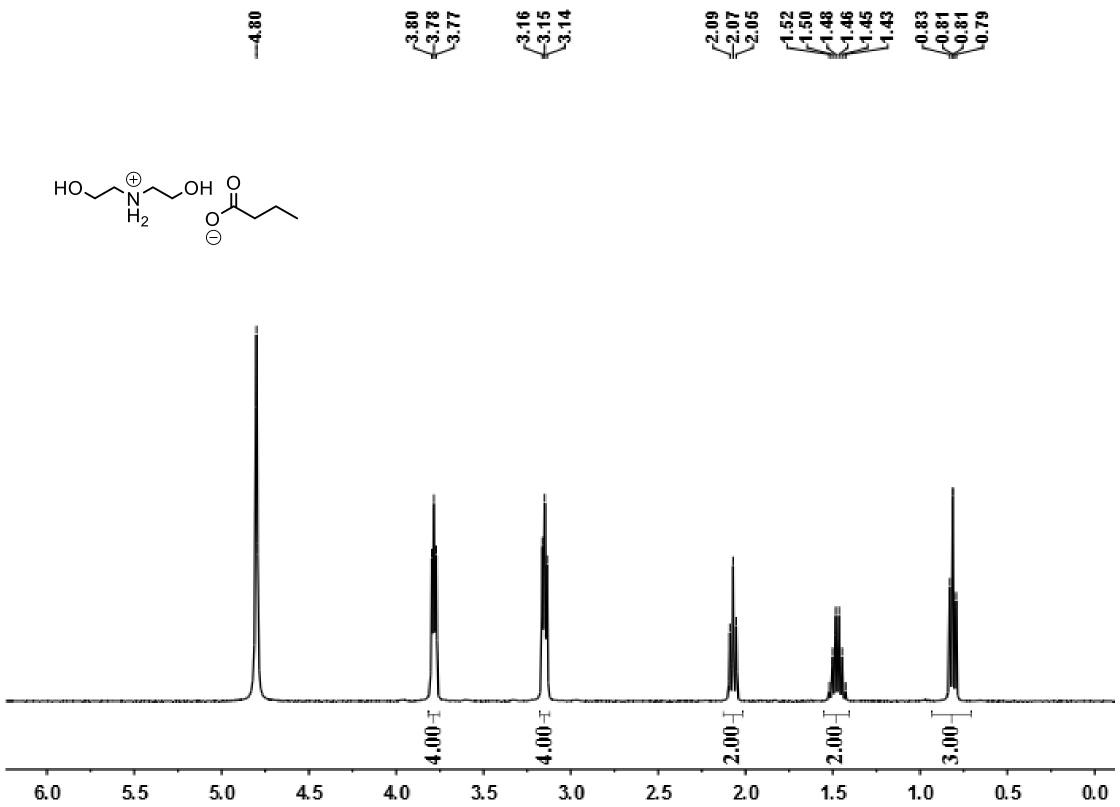
4.80
3.80
3.78
3.77
3.16
3.15
3.14
2.09
2.07
2.05
1.52
1.50
1.48
1.46
1.45
1.43
0.83
0.81
0.81
0.79
HO
N
H2
OH
O
O
4.00
4.00
2.00
2.00
3.00
6.0 5.5 5.0 4.5 4.0 3.5 3.0 2.5 2.0 1.5 1.0 0.5 0.0

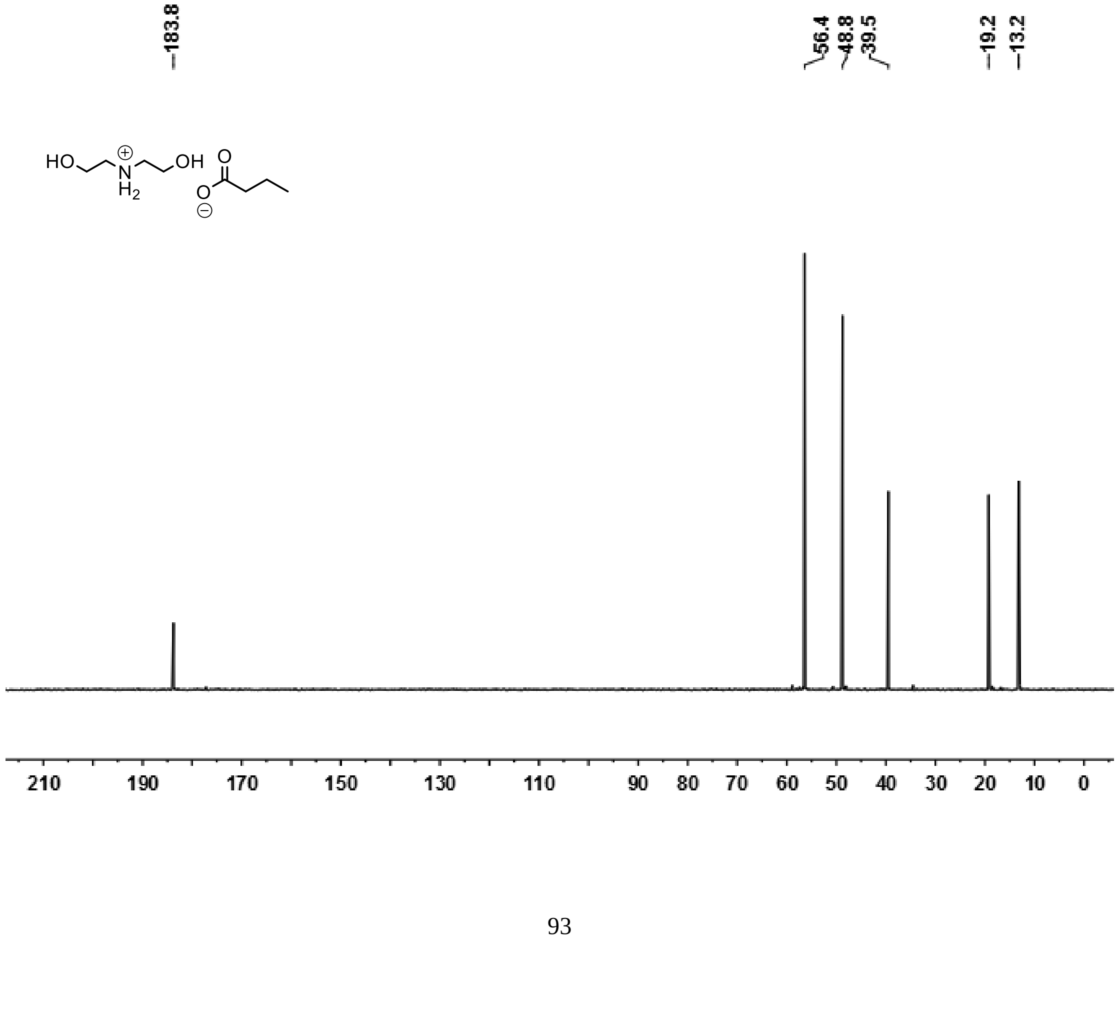
183.8
56.4
48.8
39.5
19.2
13.2
HO
N
H2
OH
O
O
210 190 170 150 130 110 90 80 70 60 50 40 30 20 10 0

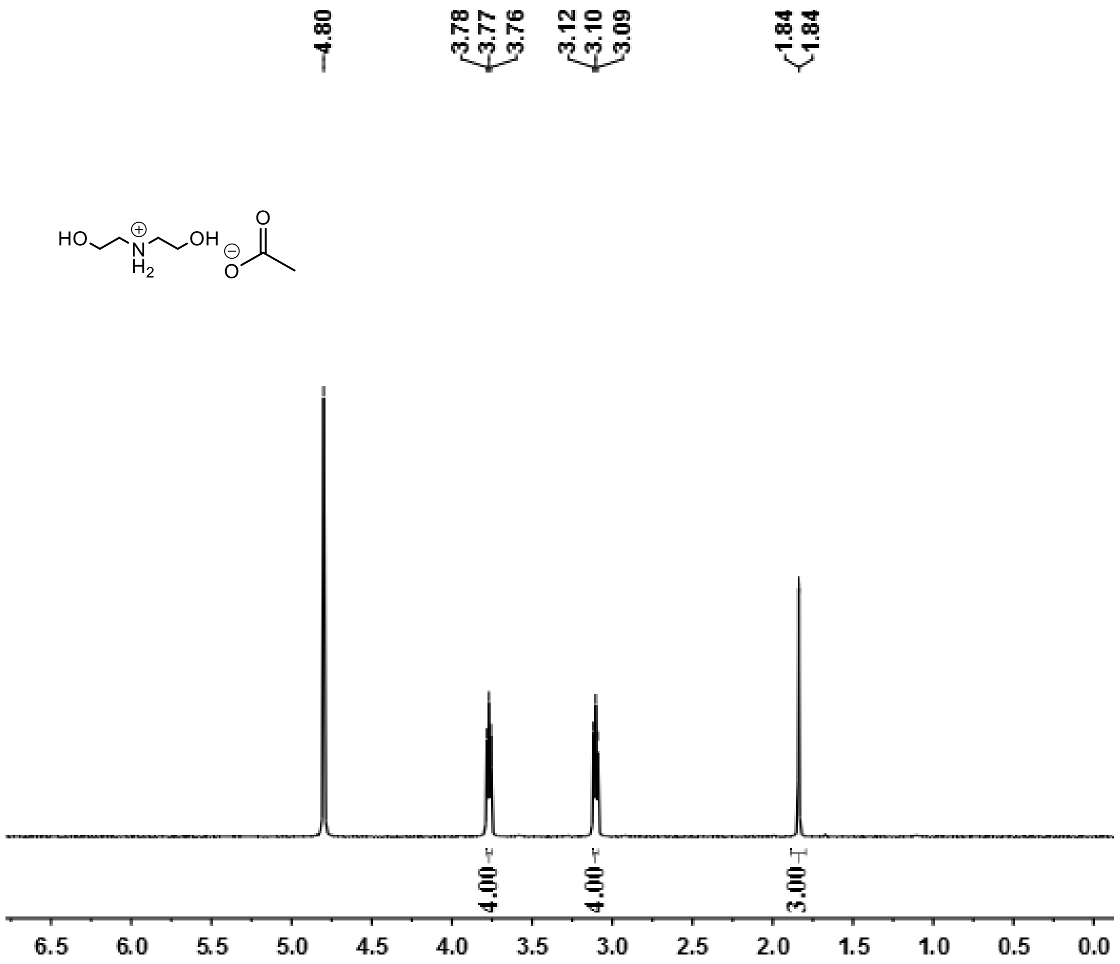
4.80
3.78
3.77
3.76
3.12
3.10
3.09
1.84
1.84
HO
OH
N
H2
O
O
4.00
4.00
3.00
6.5 6.0 5.5 5.0 4.5 4.0 3.5 3.0 2.5 2.0 1.5 1.0 0.5 0.0

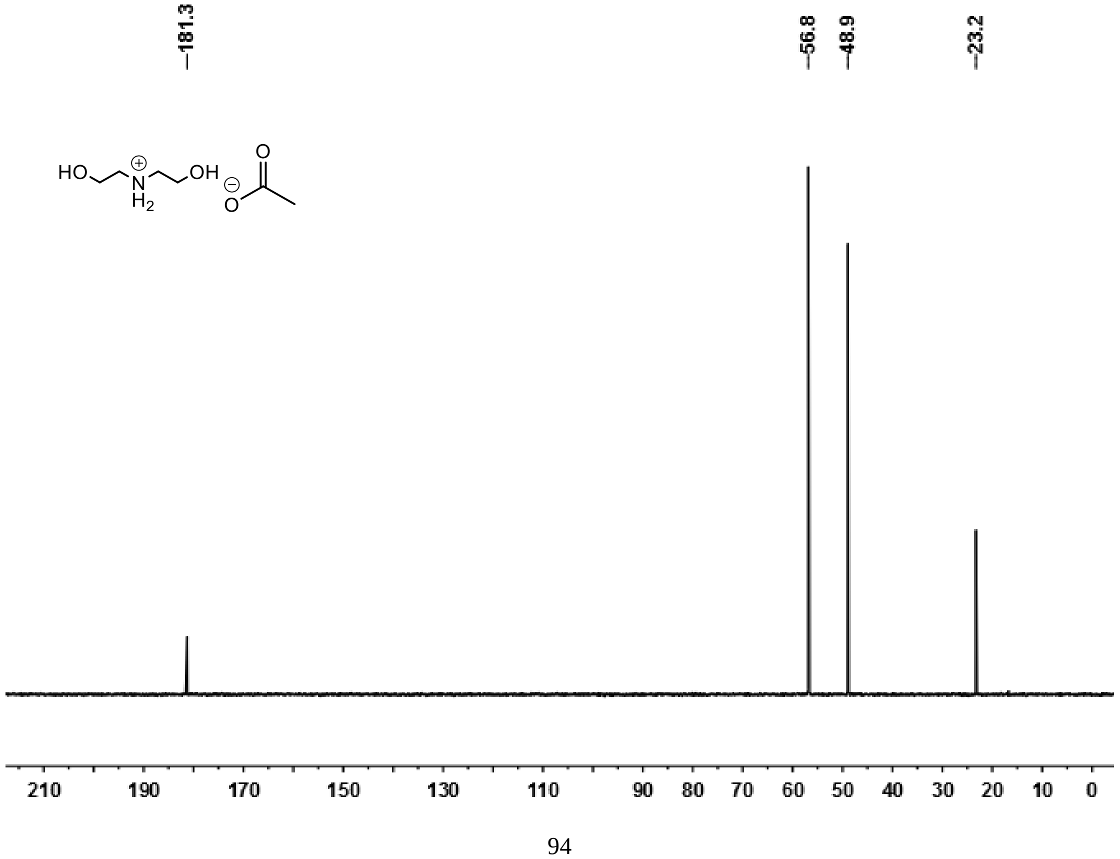
181.3
56.8
48.9
23.2
HO
OH
N
H2
O
O
210 190 170 150 130 110 90 80 70 60 50 40 30 20 10 0

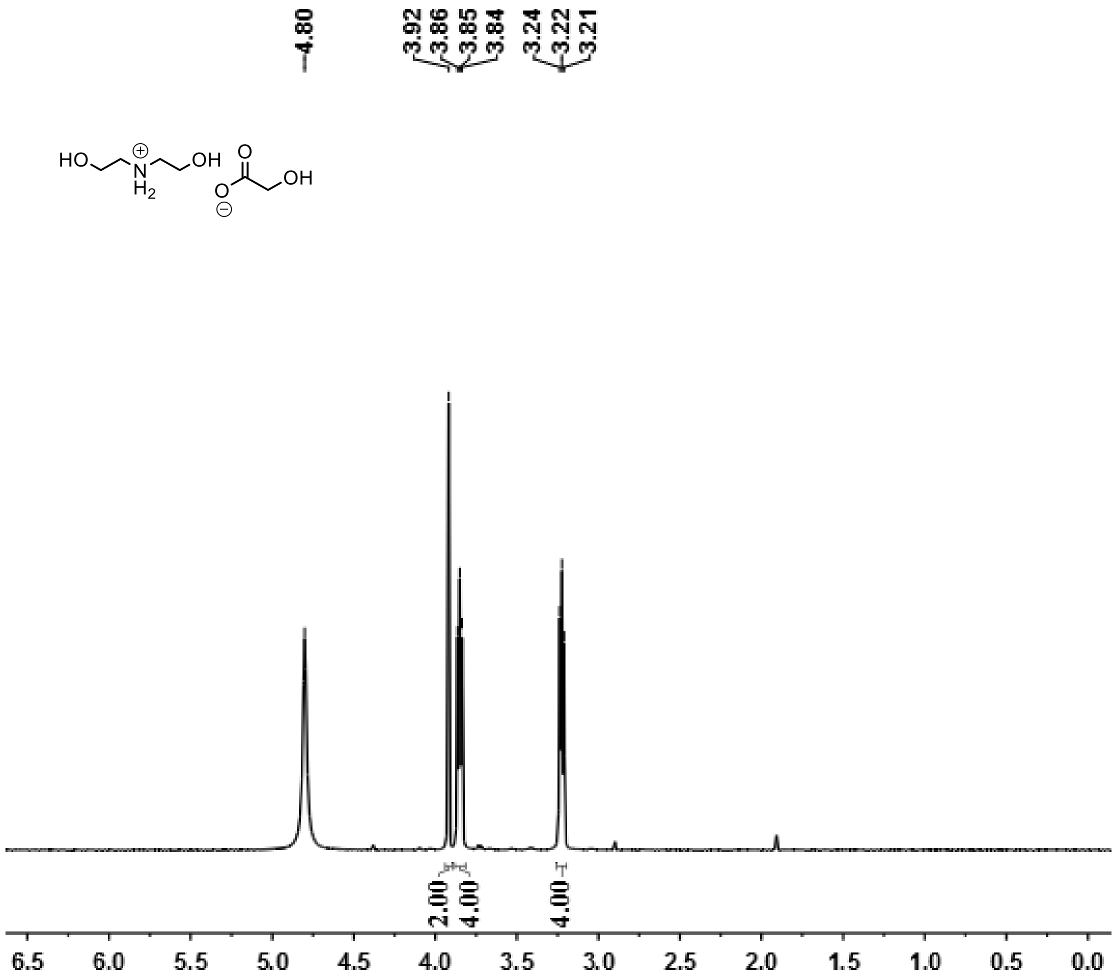
4.80
3.92
3.86
3.85
3.84
3.24
3.22
3.21
HO
N
H2
OH
O
O
OH
2.00
4.00
4.00
6.5 6.0 5.5 5.0 4.5 4.0 3.5 3.0 2.5 2.0 1.5 1.0 0.5 0.0

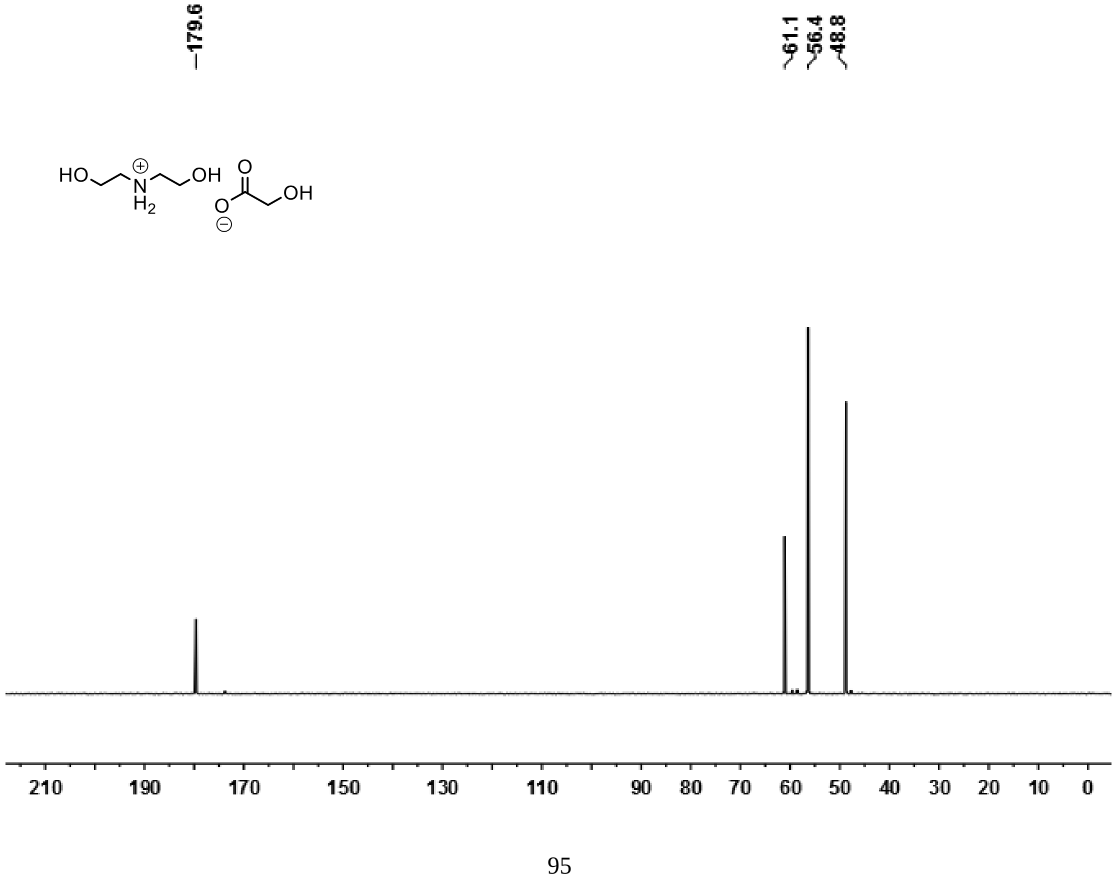
179.6
61.1
56.4
48.8
HO
N
H2
OH
O
O
OH
210 190 170 150 130 110 90 80 70 60 50 40 30 20 10 0

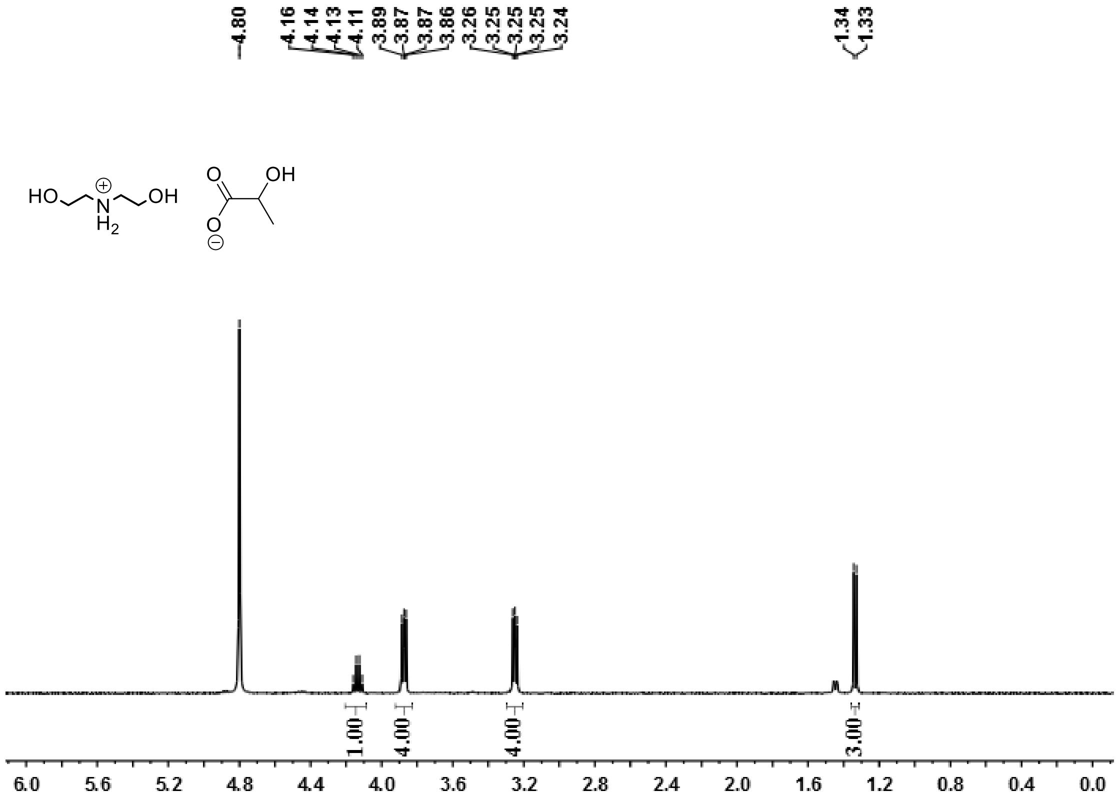
4.80
4.16
4.14
4.13
4.11
3.89
3.87
3.87
3.86
3.26
3.25
3.25
3.25
3.24
1.34
1.33
1.00
4.00
4.00
3.00
6.0 5.6 5.2 4.8 4.4 4.0 3.6 3.2 2.8 2.4 2.0 1.6 1.2 0.8 0.4 0.0

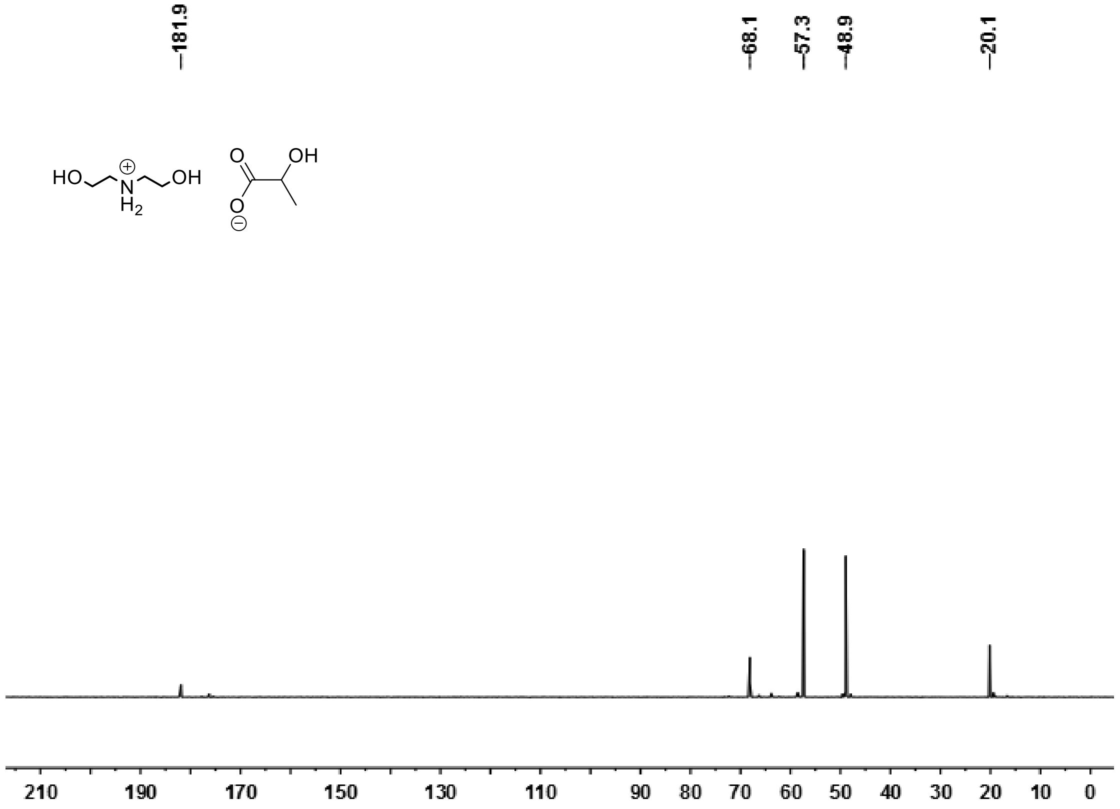
181.9
68.1
57.3
48.9
20.1
210 190 170 150 130 110 90 80 70 60 50 40 30 20 10 0

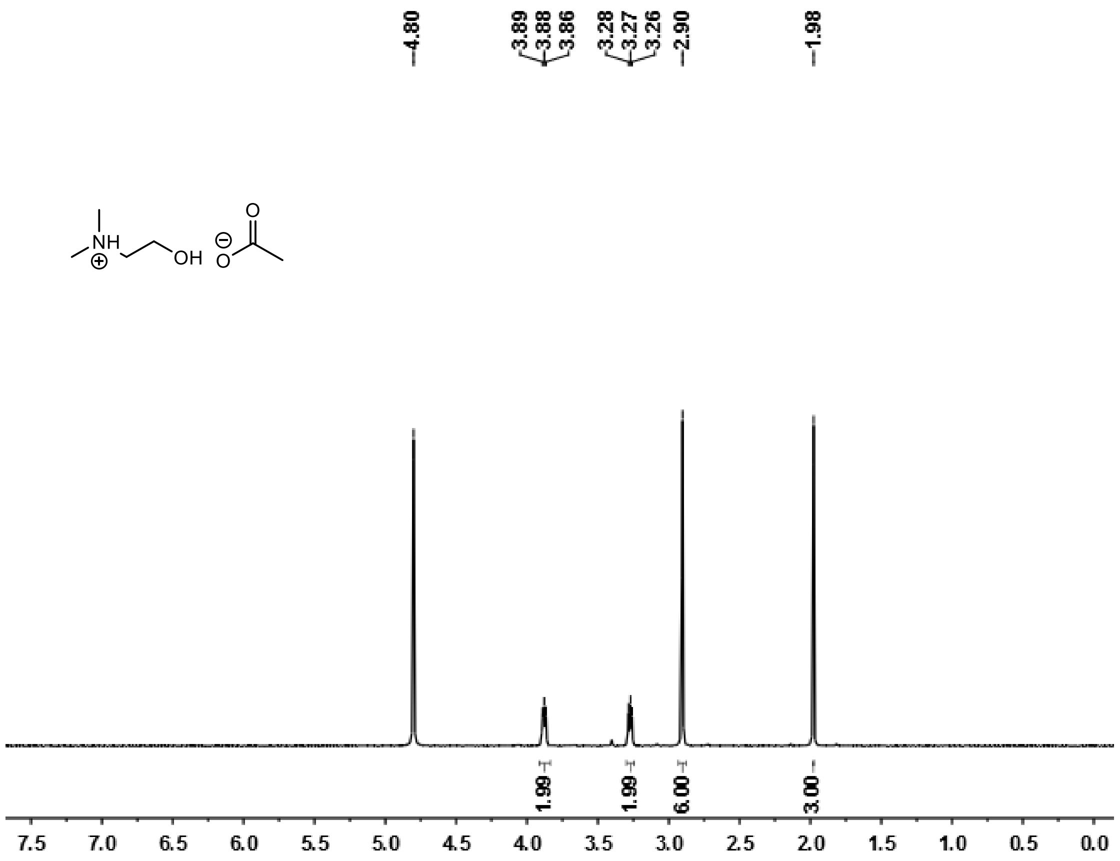
4.80
3.89
3.88
3.86
3.28
3.27
3.26
2.90
1.98
O
NH
OH
O
1.99
1.99
6.00
3.00
7.5 7.0 6.5 6.0 5.5 5.0 4.5 4.0 3.5 3.0 2.5 2.0 1.5 1.0 0.5 0.0

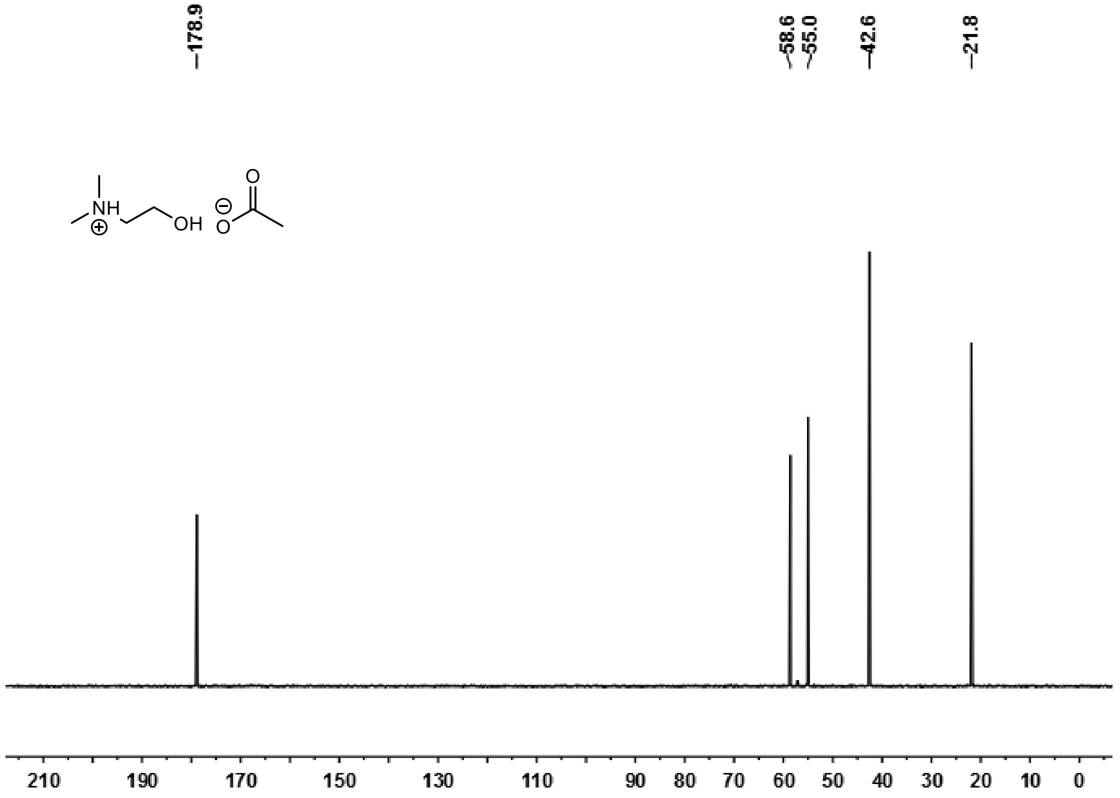
178.9
58.6
55.0
42.6
21.8
O
NH
OH
O
210 190 170 150 130 110 90 80 70 60 50 40 30 20 10 0

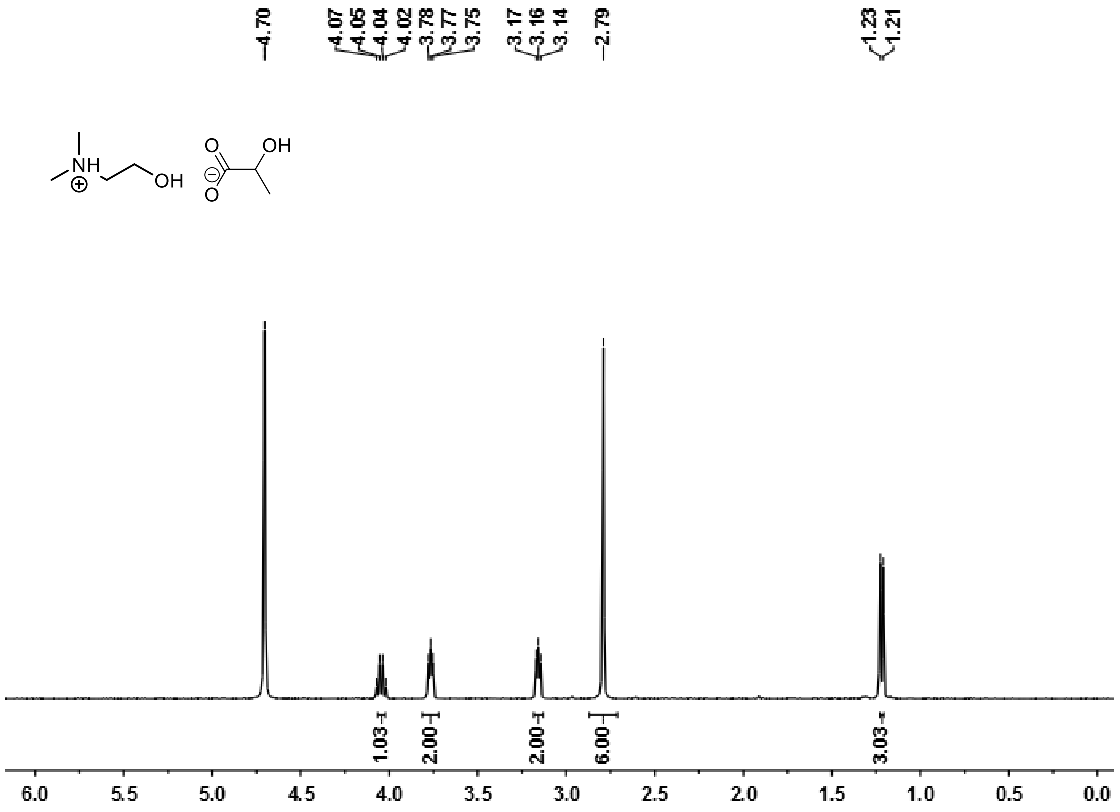
4.70
4.07
4.05
4.04
4.02
3.78
3.77
3.75
3.17
3.16
3.14
2.79
1.23
1.21
NH
OH
O
OH
O
1.03
2.00
2.00
6.00
3.03
6.0
5.5
5.0
4.5
4.0
3.5
3.0
2.5
2.0
1.5
1.0
0.5
0.0

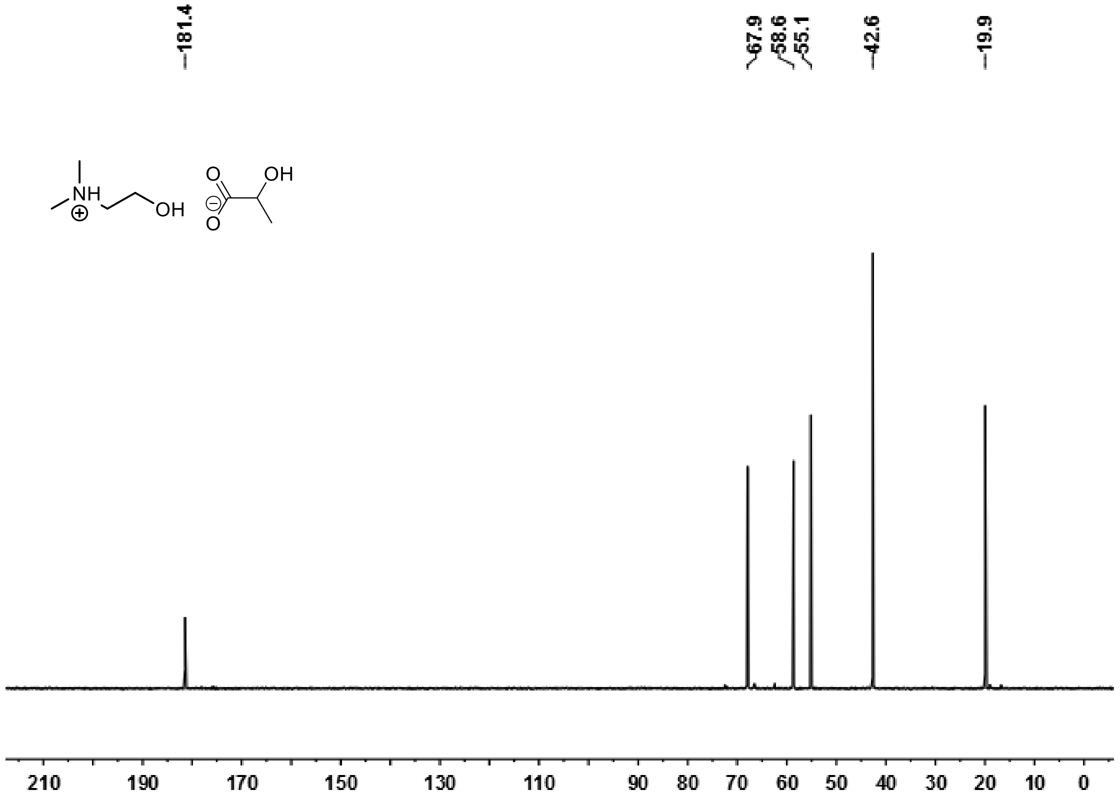
181.4
67.9
58.6
55.1
42.6
19.9
NH
OH
O
OH
O
210
190
170
150
130
110
90
80
70
60
50
40
30
20
10
0

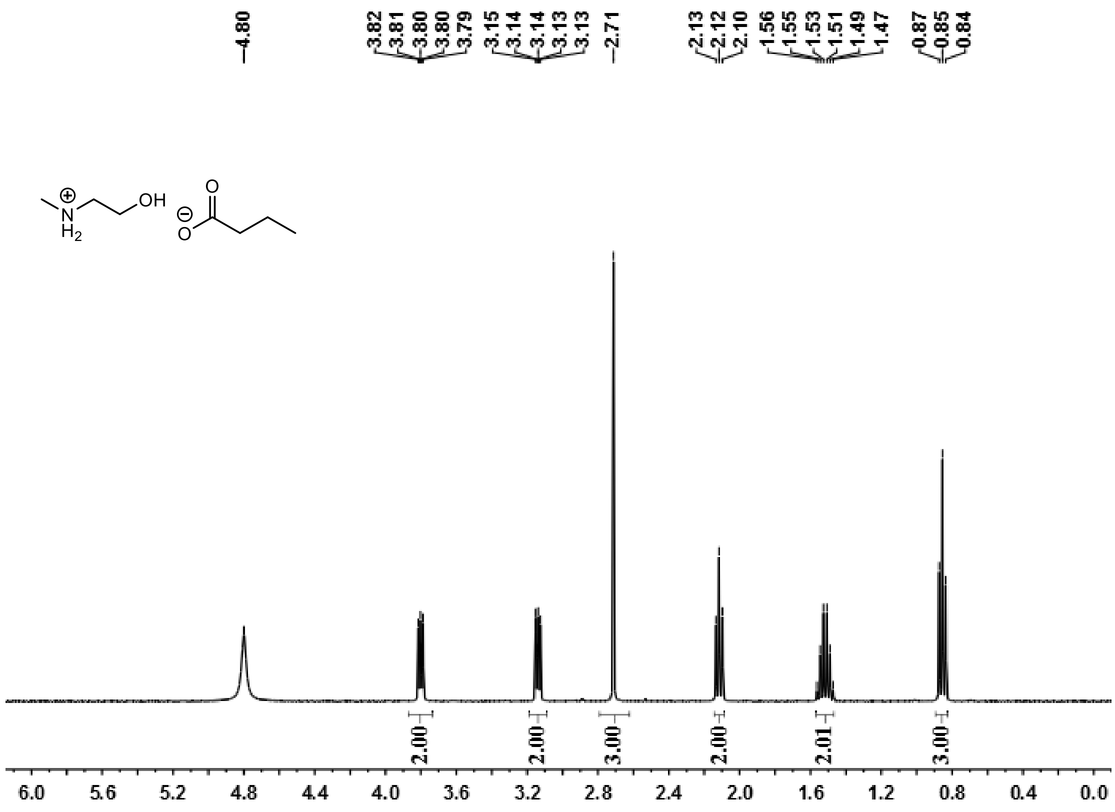
4.80
3.82
3.81
3.80
3.80
3.79
3.15
3.14
3.14
3.13
3.13
2.71
2.13
2.12
2.10
1.56
1.55
1.53
1.51
1.49
1.47
0.87
0.85
0.84
OH
N
H2
O
O
2.00
2.00
3.00
2.00
2.01
3.00
6.0
5.6
5.2
4.8
4.4
4.0
3.6
3.2
2.8
2.4
2.0
1.6
1.2
0.8
0.4
0.0

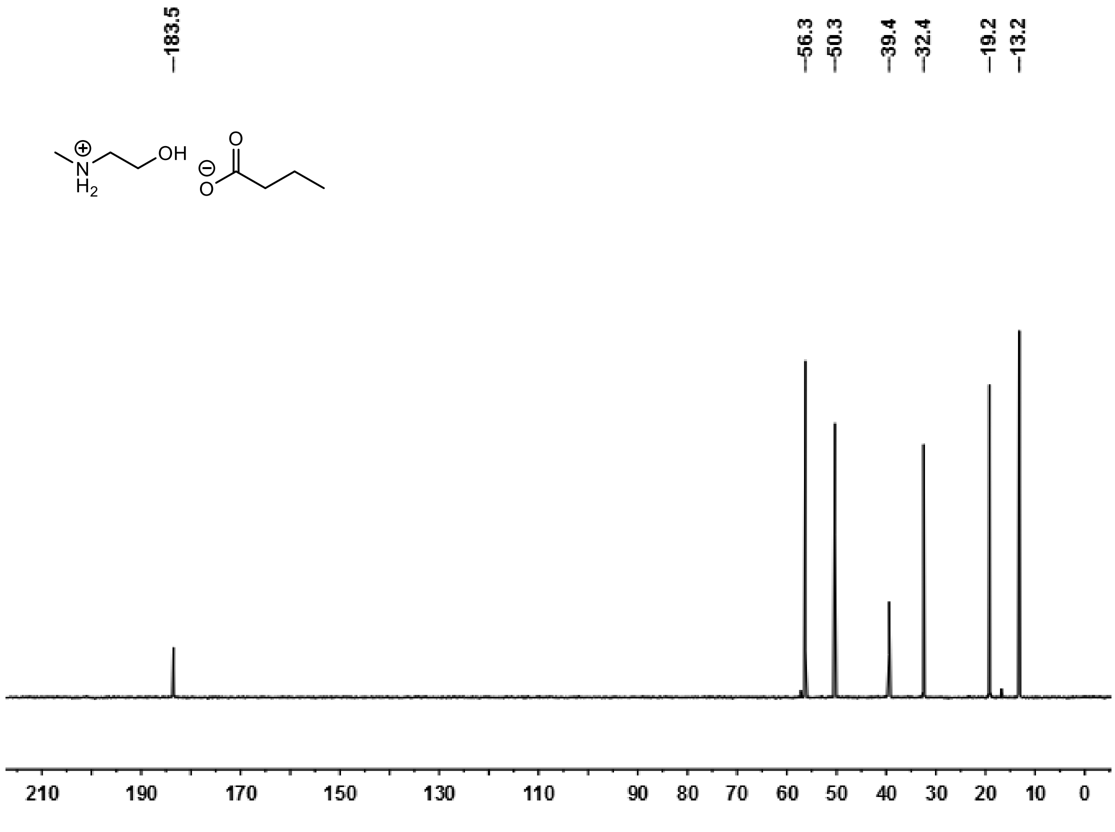
183.5
56.3
50.3
39.4
32.4
19.2
13.2
OH
N
H2
O
O
210
190
170
150
130
110
90
80
70
60
50
40
30
20
10
0

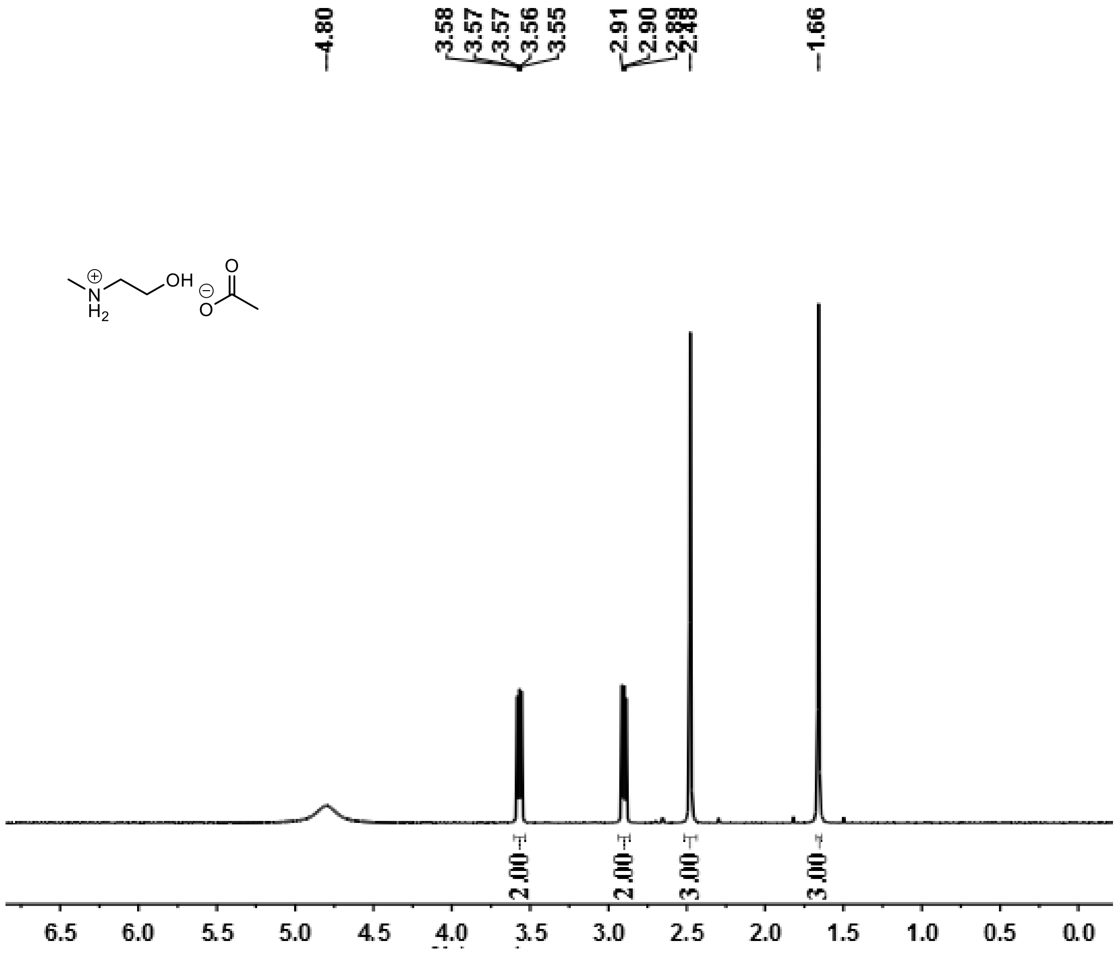
4.80
3.58
3.57
3.57
3.56
3.55
2.91
2.90
2.89
2.48
1.66
OH
O
O
H2
2.00
2.00
3.00
3.00
6.5 6.0 5.5 5.0 4.5 4.0 3.5 3.0 2.5 2.0 1.5 1.0 0.5 0.0

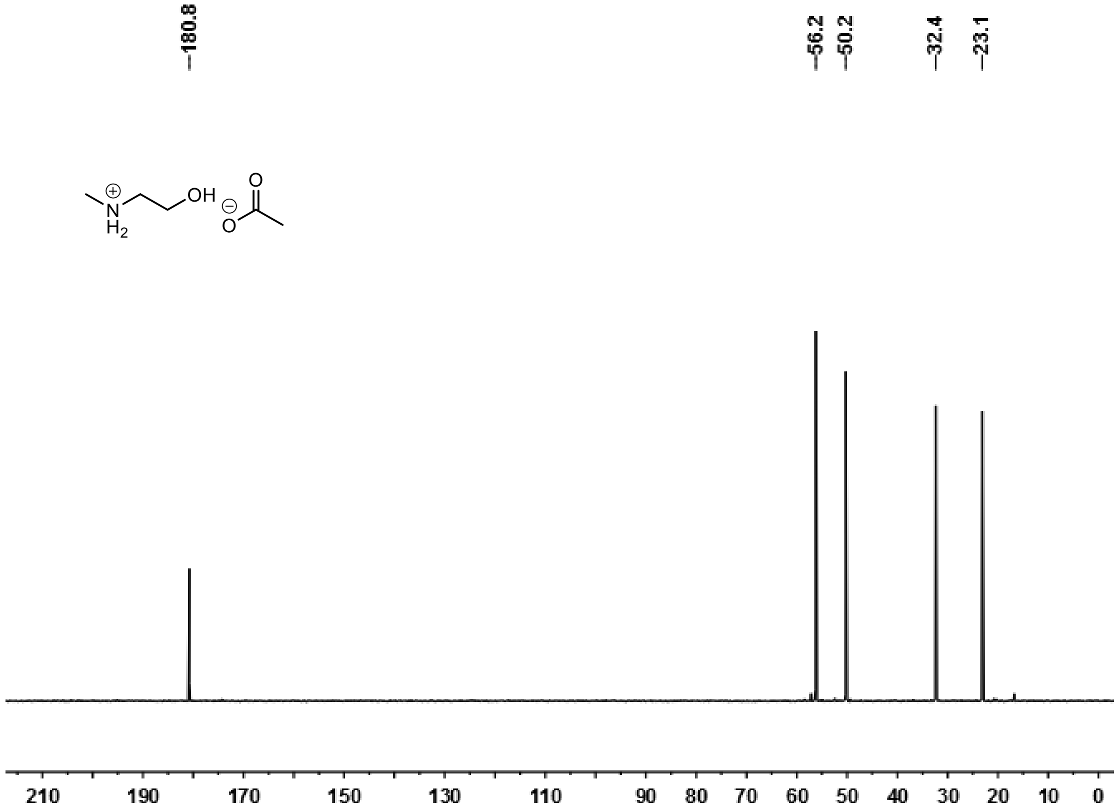
180.8
56.2
50.2
32.4
23.1
OH
O
O
H2
210 190 170 150 130 110 90 80 70 60 50 40 30 20 10 0

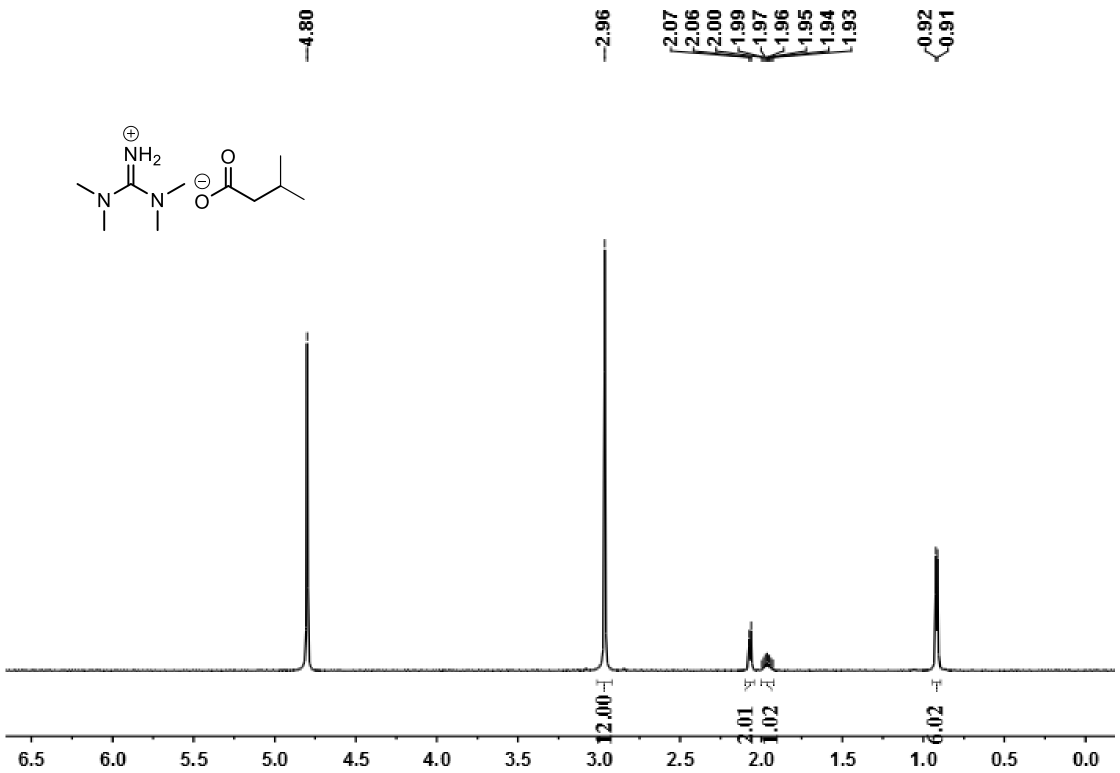
4.80
2.96
2.07
2.06
2.00
1.99
1.97
1.96
1.95
1.94
1.93
0.92
0.91
NH2
O
N
N
O
12.00
2.01
1.02
6.02
6.5
6.0
5.5
5.0
4.5
4.0
3.5
3.0
2.5
2.0
1.5
1.0
0.5
0.0

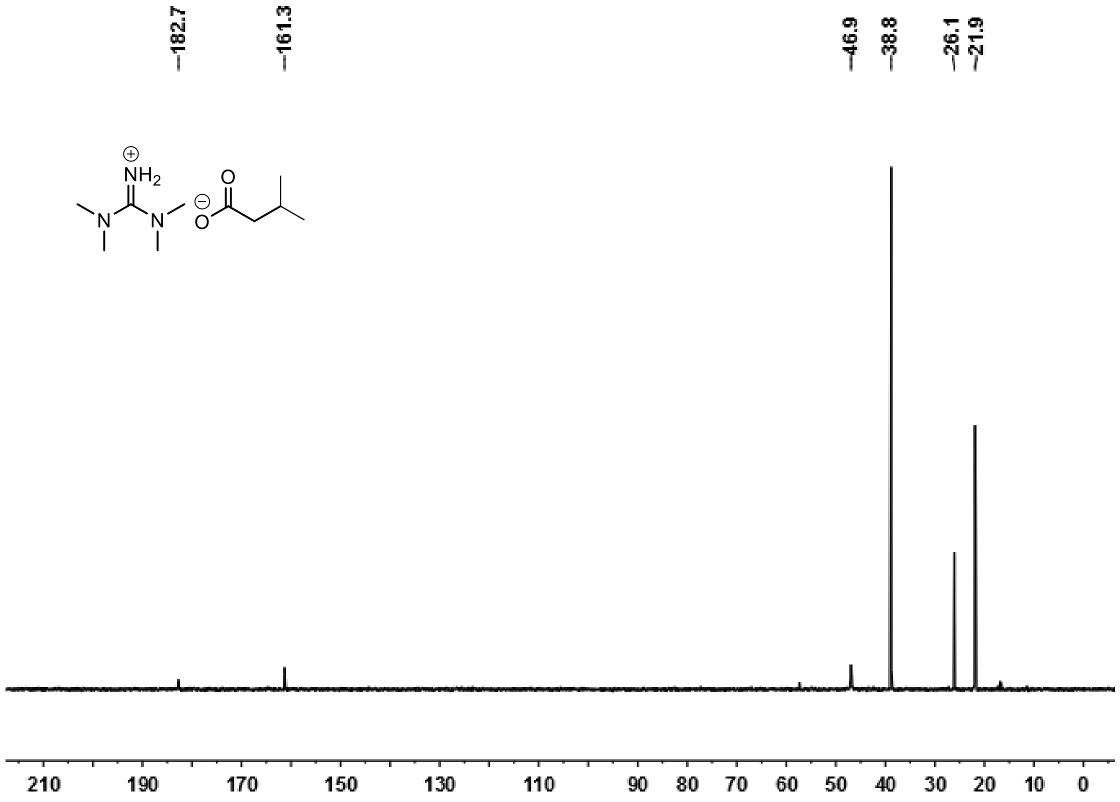
182.7
161.3
46.9
38.8
26.1
21.9
NH2
O
N
N
O
210
190
170
150
130
110
90
80
70
60
50
40
30
20
10
0

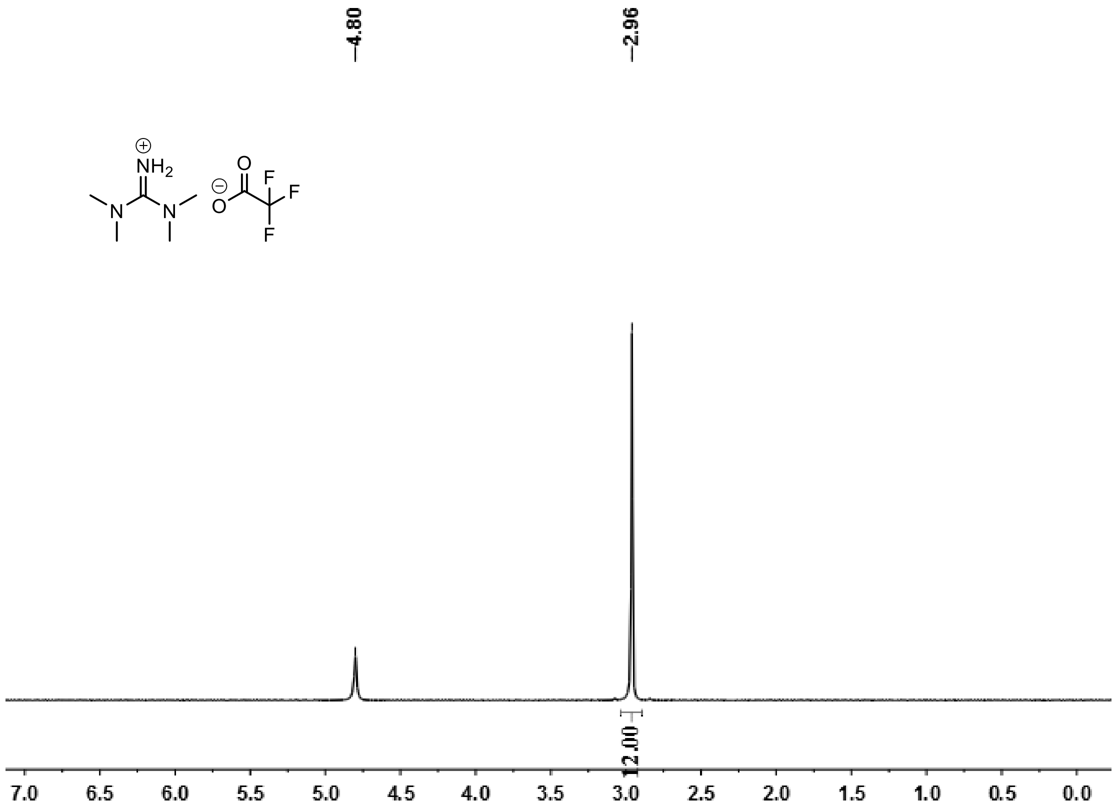

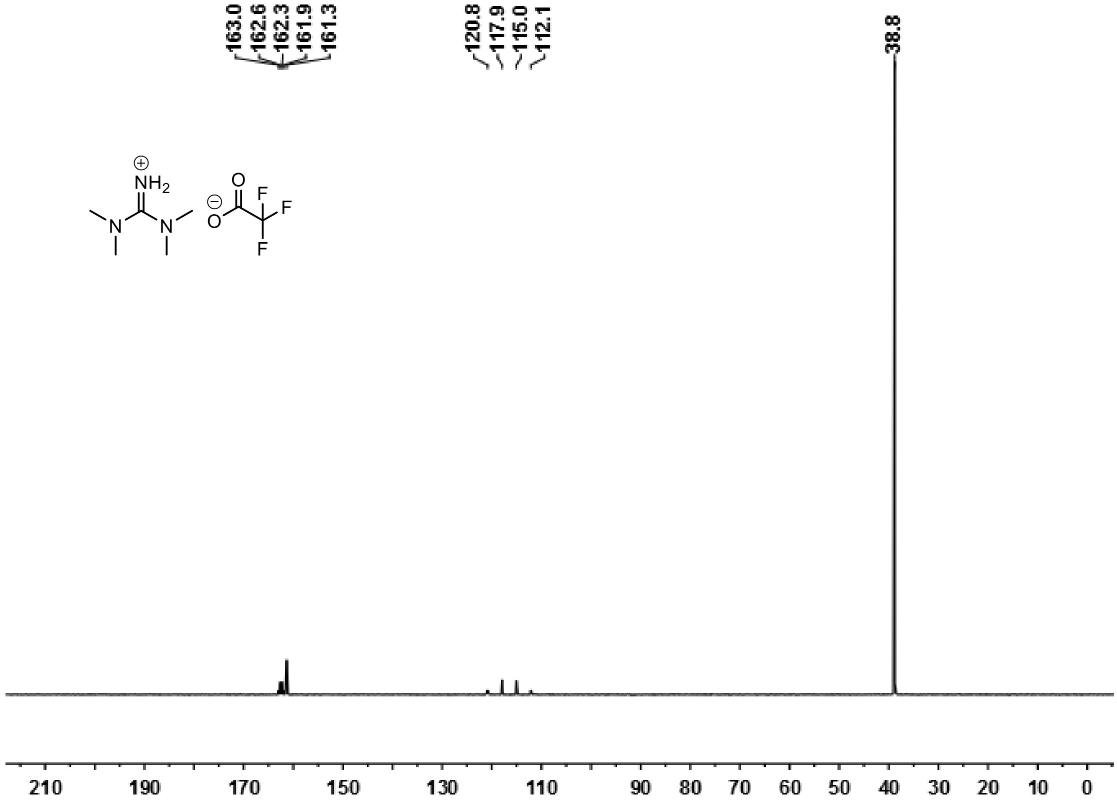

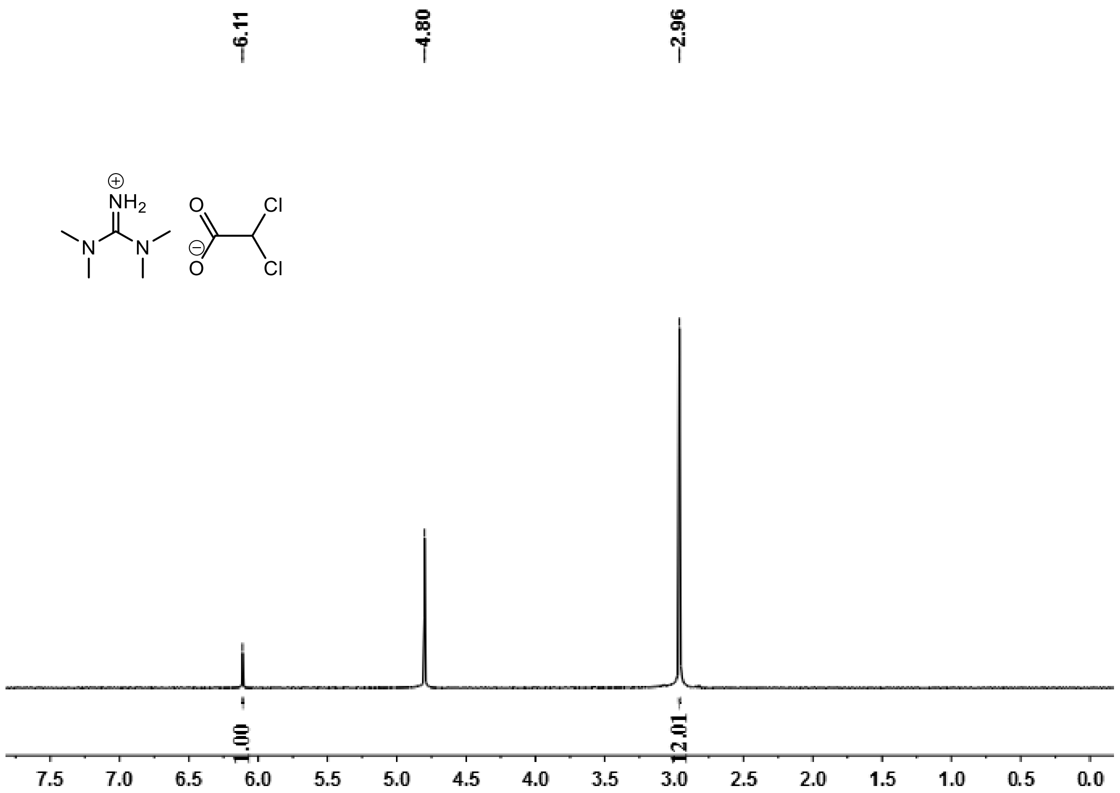
6.11
4.80
2.96
NH2
O
Cl
N
N
O
Cl
1.00
12.01
7.5 7.0 6.5 6.0 5.5 5.0 4.5 4.0 3.5 3.0 2.5 2.0 1.5 1.0 0.5 0.0

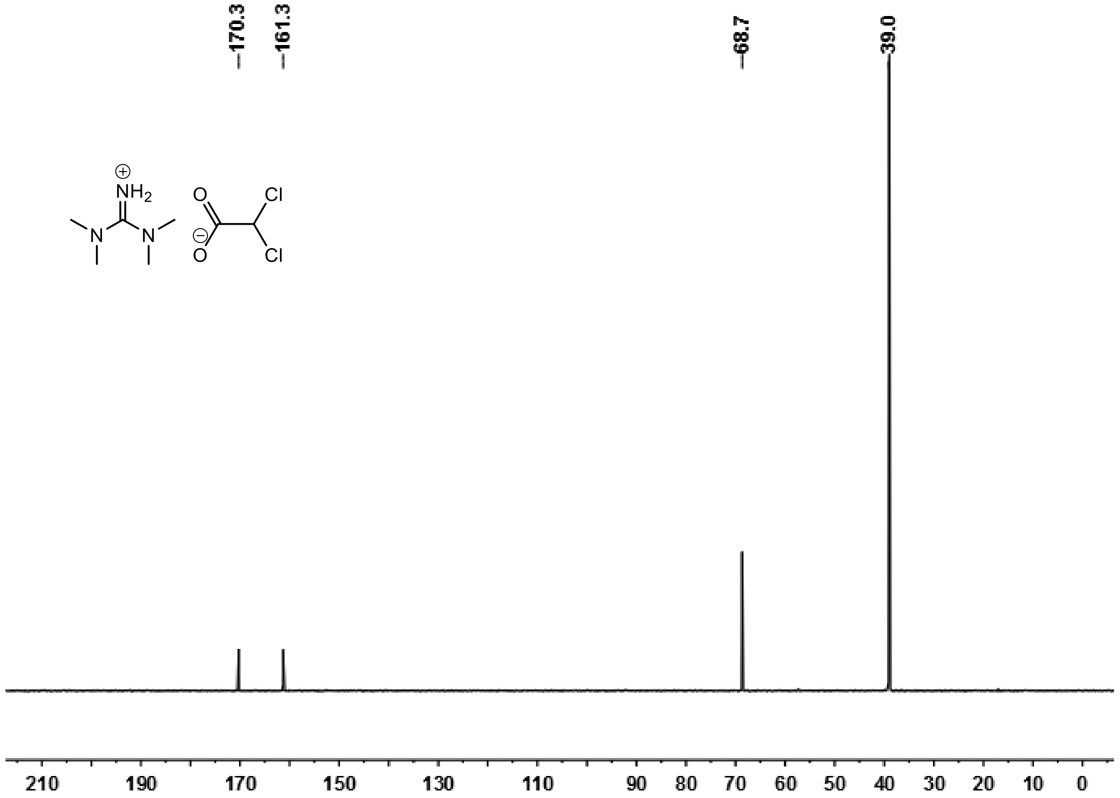
170.3
161.3
68.7
39.0
NH2
O
Cl
N
N
O
Cl
210 190 170 150 130 110 90 80 70 60 50 40 30 20 10 0

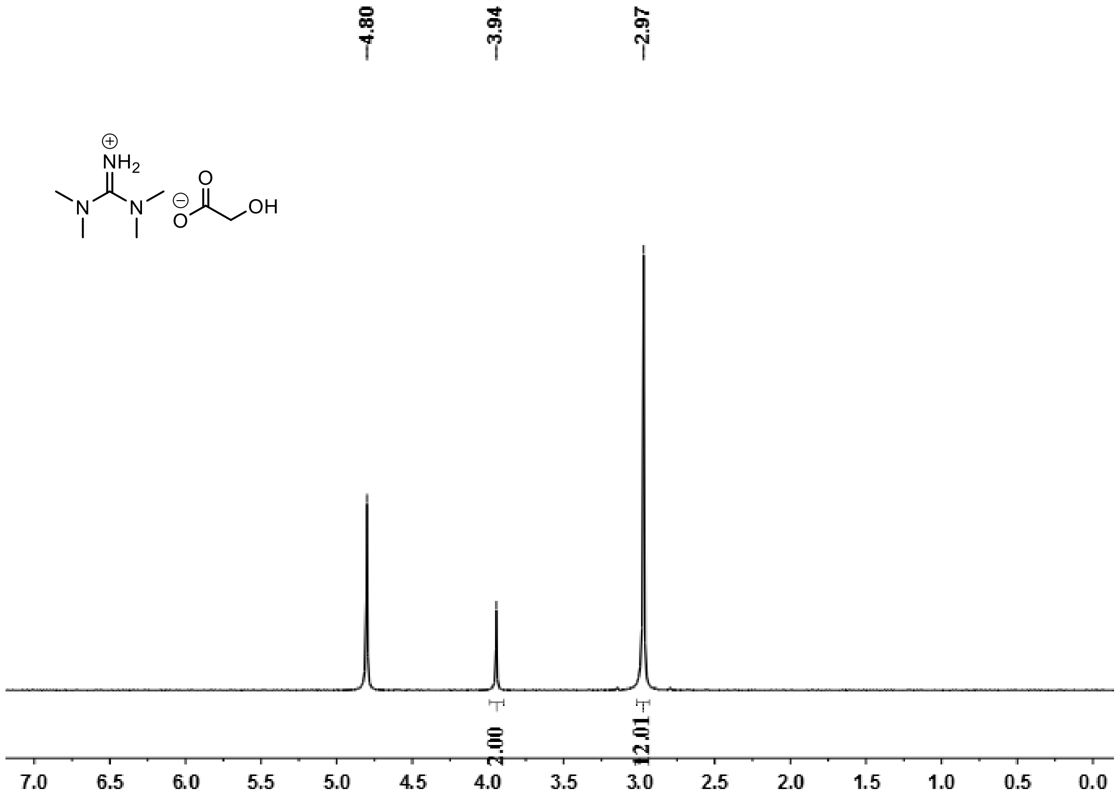
4.80
3.94
2.97
NH2
N
N
O
O
OH
2.00
12.01
7.0 6.5 6.0 5.5 5.0 4.5 4.0 3.5 3.0 2.5 2.0 1.5 1.0 0.5 0.0

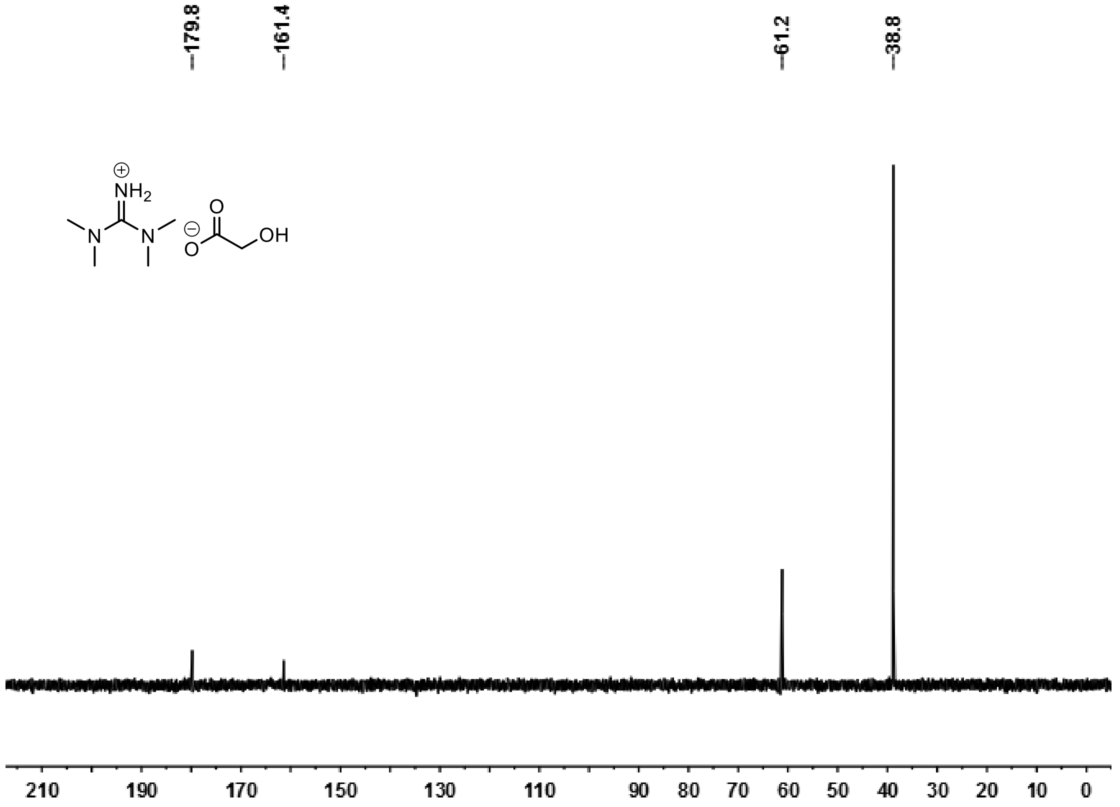
179.8
161.4
61.2
38.8
NH2
N
N
O
O
OH
210 190 170 150 130 110 90 80 70 60 50 40 30 20 10 0

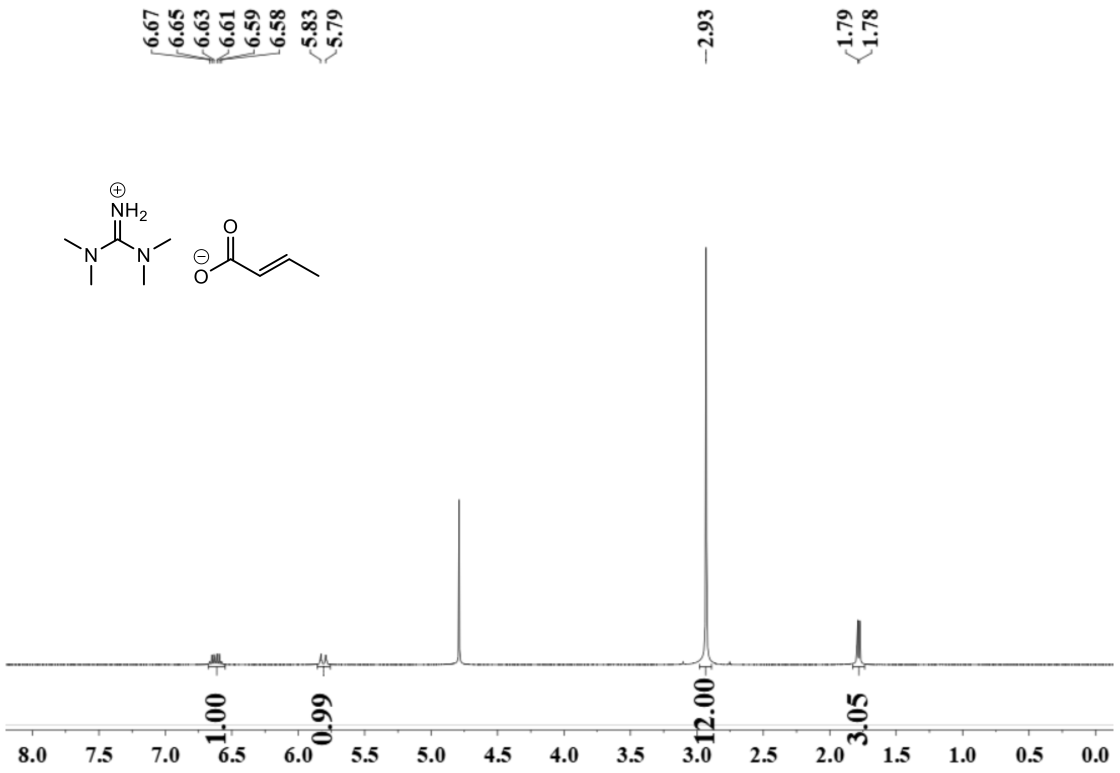
6.67 6.65 6.63 6.61 6.59 6.58 5.83 5.79
2.93
1.79 1.78
NH2
N N
O
O
1.00 0.99 12.00 3.05
8.0 7.5 7.0 6.5 6.0 5.5 5.0 4.5 4.0 3.5 3.0 2.5 2.0 1.5 1.0 0.5 0.0

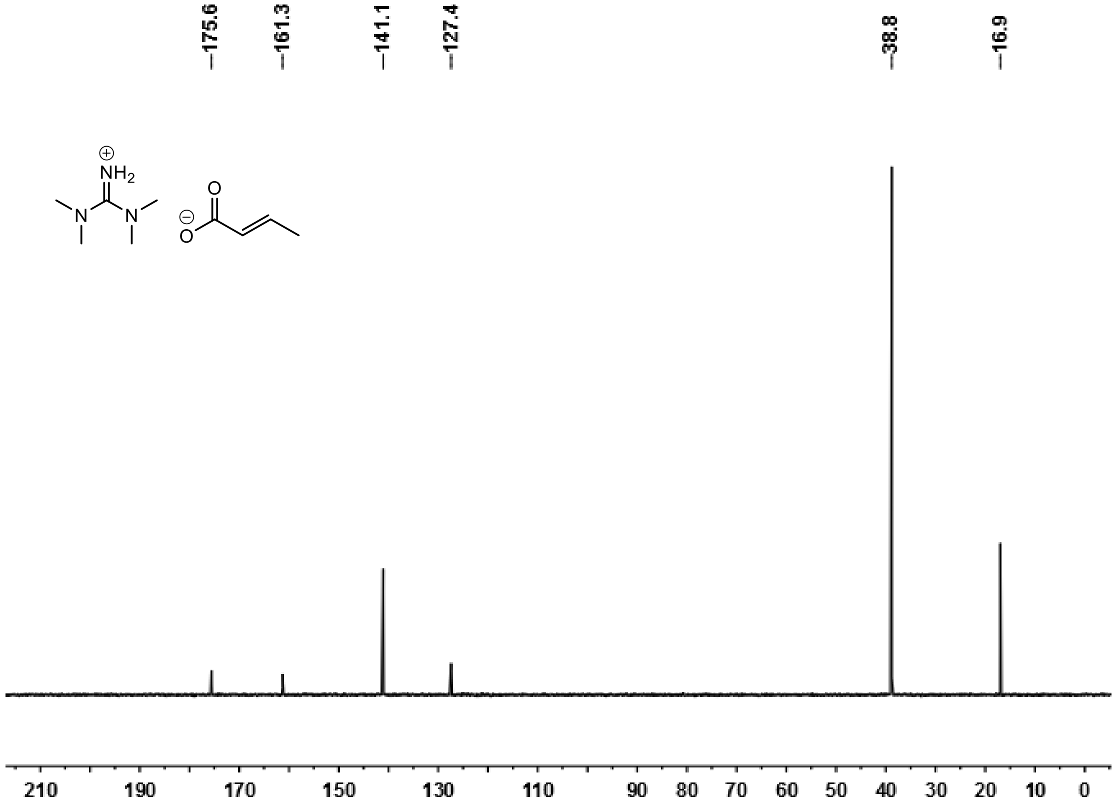
175.6 161.3 141.1 127.4 38.8 16.9
NH2
N N
O
O
210 190 170 150 130 110 90 80 70 60 50 40 30 20 10 0

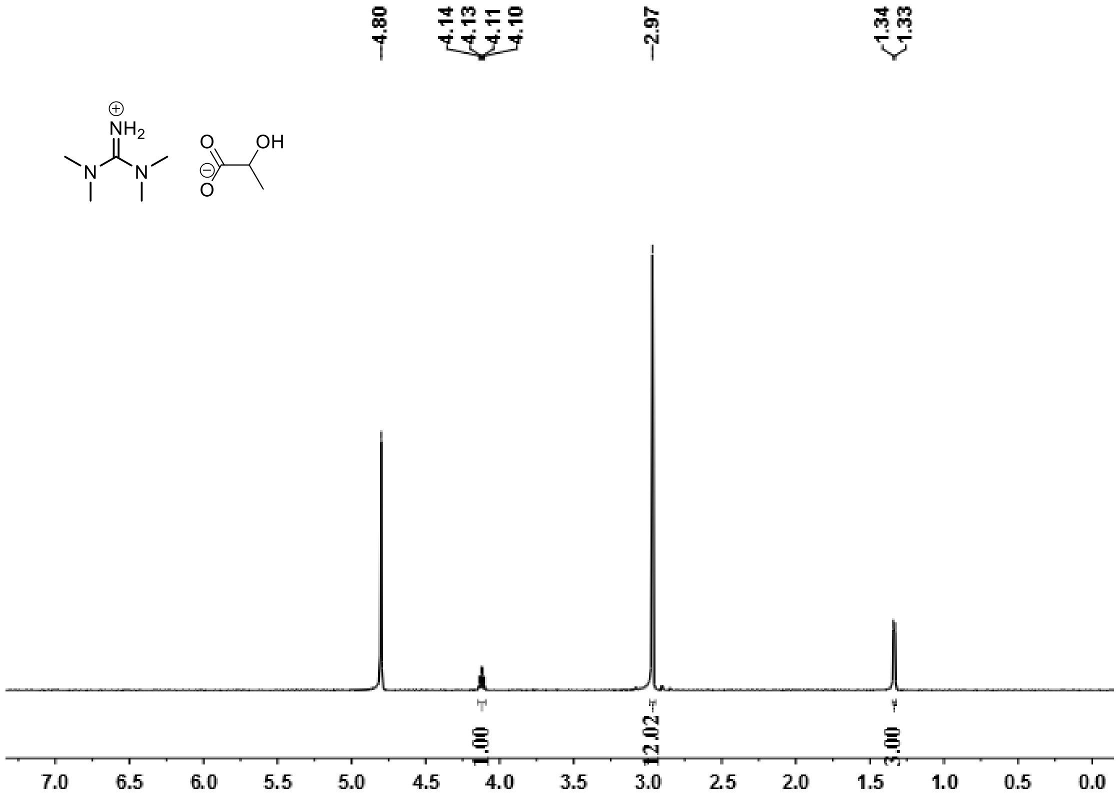
4.80
4.14
4.13
4.11
4.10
2.97
1.34
1.33
NH2
N
N
O
OH
O
1.00
12.02
3.00
7.0 6.5 6.0 5.5 5.0 4.5 4.0 3.5 3.0 2.5 2.0 1.5 1.0 0.5 0.0

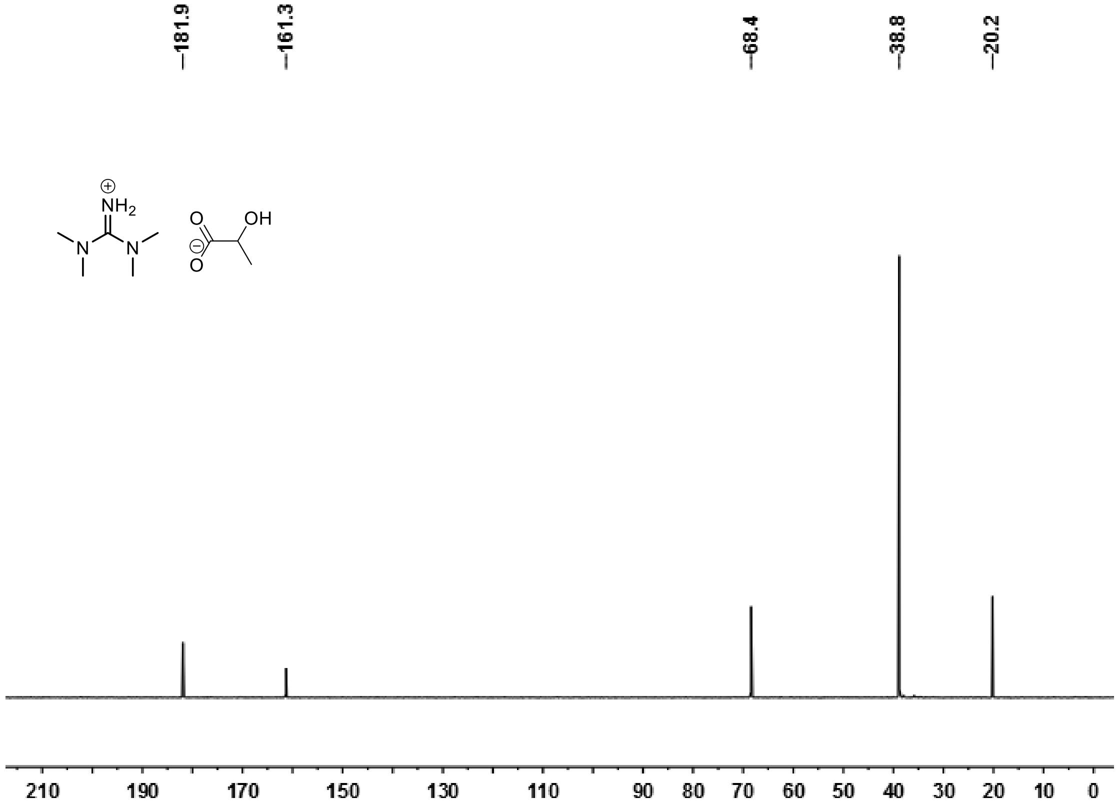
181.9
161.3
68.4
38.8
20.2
NH2
N
N
O
OH
O
210 190 170 150 130 110 90 80 70 60 50 40 30 20 10 0

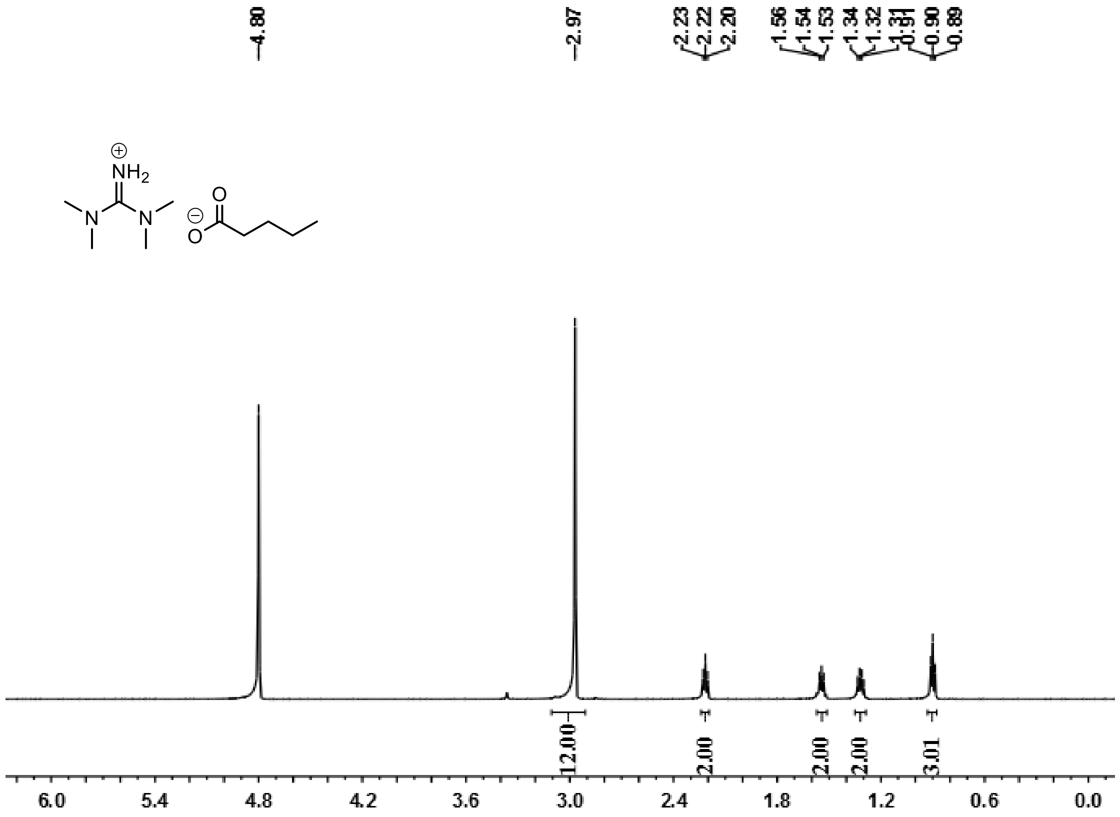

4.80
2.97
2.23
2.22
2.20
1.56
1.54
1.53
1.34
1.32
1.31
0.91
0.90
0.89
NH2
O
N
N
O
12.00
2.00
2.00
2.00
3.01
6.0
5.4
4.8
4.2
3.6
3.0
2.4
1.8
1.2
0.6
0.0

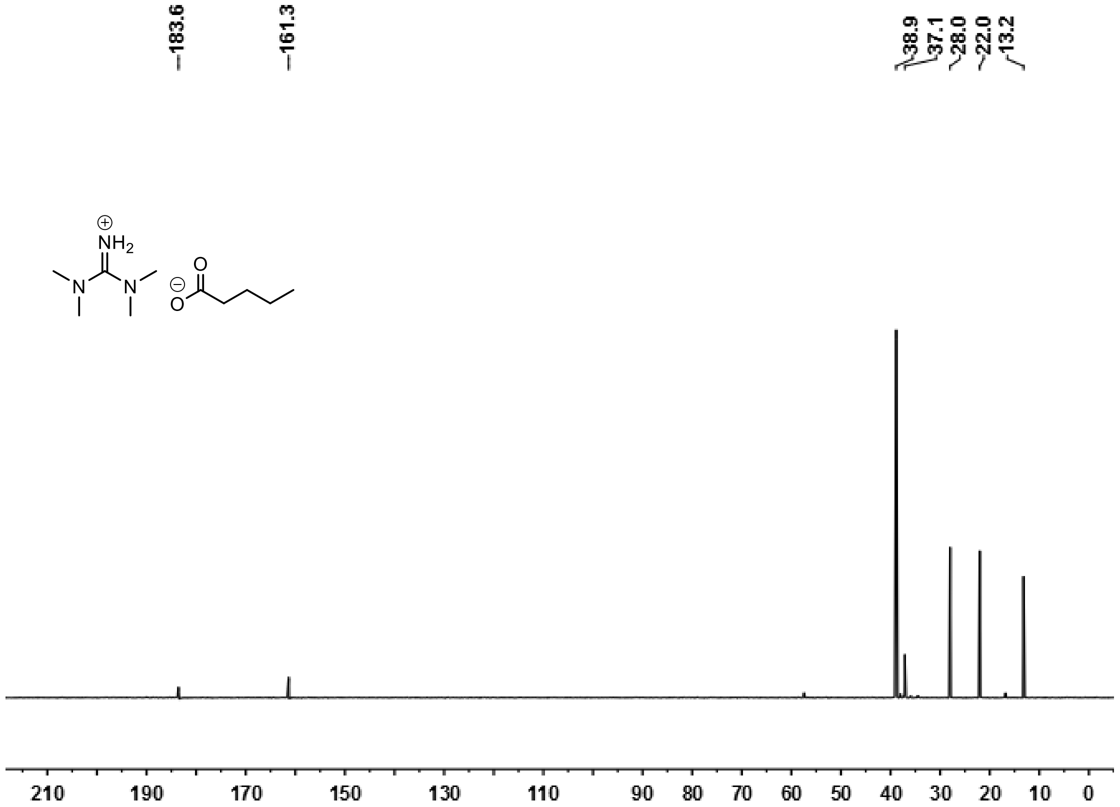

183.6
161.3
38.9
37.1
28.0
22.0
13.2
NH2
O
N
N
O
210
190
170
150
130
110
90
80
70
60
50
40
30
20
10
0

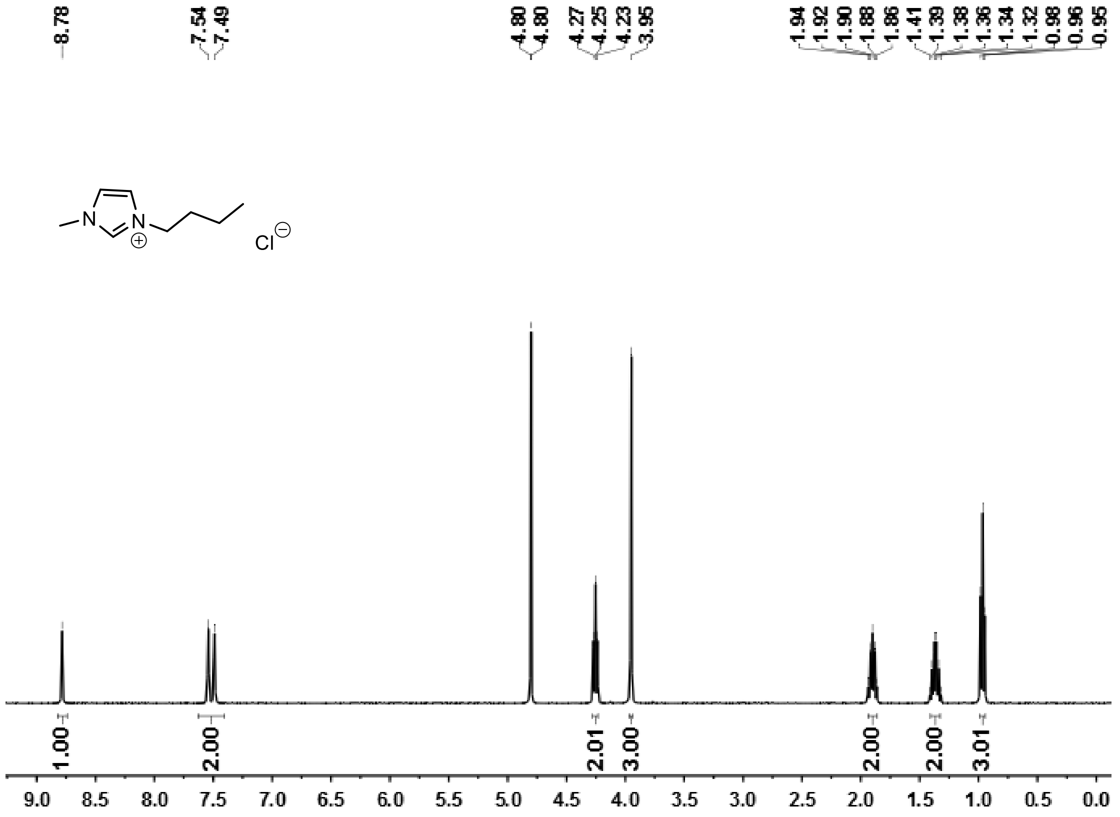
8.78
7.54 7.49
4.80 4.80
4.27 4.25 4.23 3.95
1.94 1.92 1.90 1.88 1.86 1.41 1.39 1.38 1.36 1.34 1.32 0.98 0.96 0.95
N
N
Cl
1.00
2.00
2.01
3.00
2.00
2.00
3.01
9.0 8.5 8.0 7.5 7.0 6.5 6.0 5.5 5.0 4.5 4.0 3.5 3.0 2.5 2.0 1.5 1.0 0.5 0.0

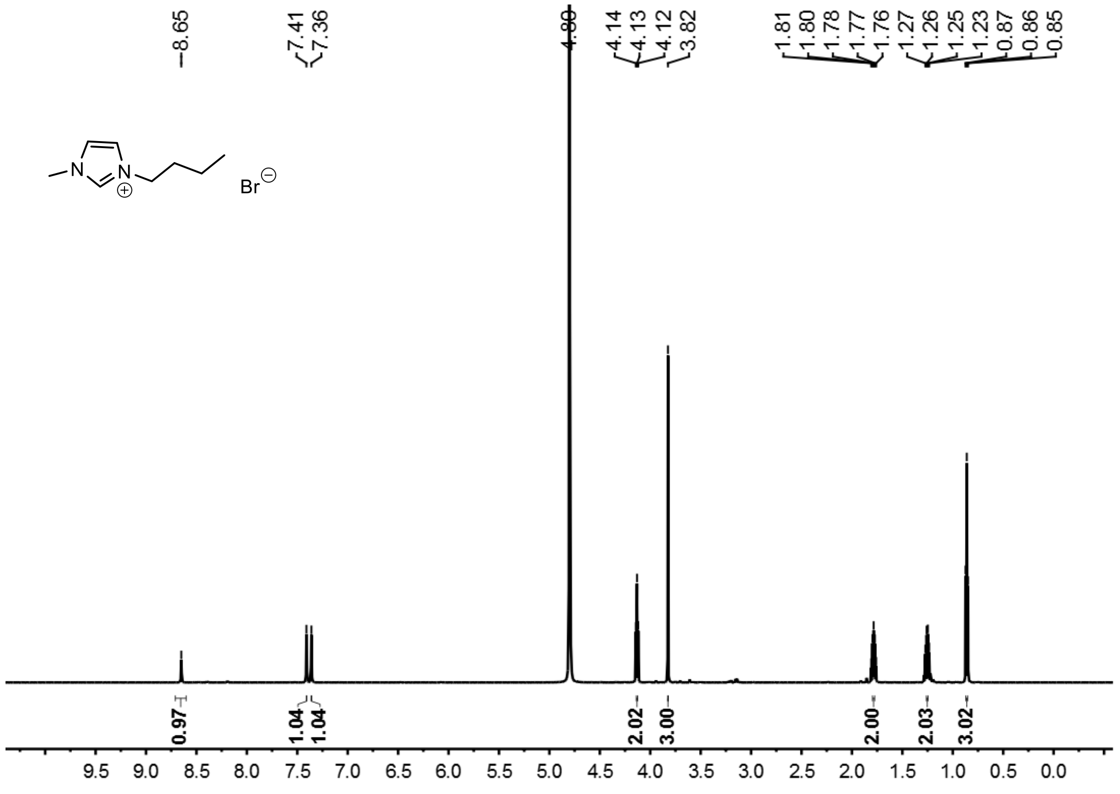
8.65
7.41 7.36
4.80
4.14 4.13 4.12 3.82
1.81 1.80 1.78 1.77 1.76 1.27 1.26 1.25 1.23 0.87 0.86 0.85
N
N
Br
0.97
1.04 1.04
2.02
3.00
2.00
2.03
3.02
9.5 9.0 8.5 8.0 7.5 7.0 6.5 6.0 5.5 5.0 4.5 4.0 3.5 3.0 2.5 2.0 1.5 1.0 0.5 0.0

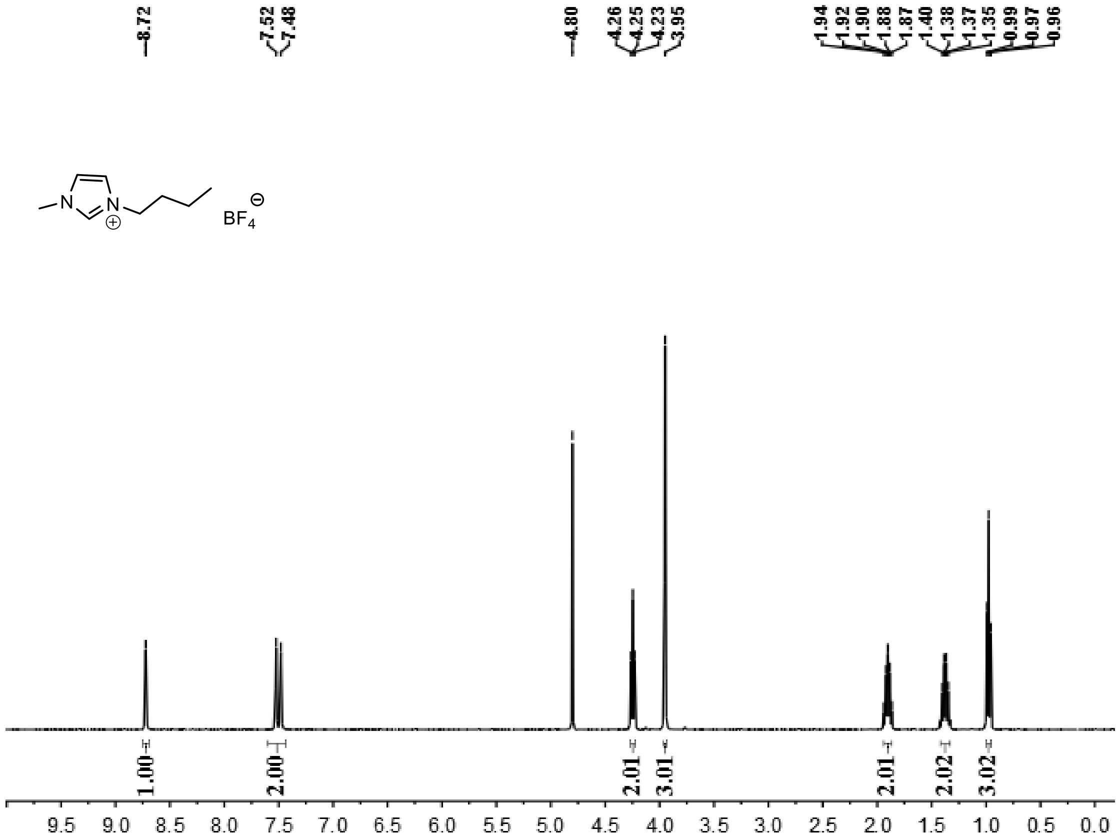
8.72
7.52
7.48
4.80
4.26
4.25
4.23
3.95
1.94
1.92
1.90
1.88
1.87
1.40
1.38
1.37
1.35
0.99
0.97
0.96
N
N
BF4
1.00
2.00
2.01
3.01
2.01
2.02
3.02
9.5 9.0 8.5 8.0 7.5 7.0 6.5 6.0 5.5 5.0 4.5 4.0 3.5 3.0 2.5 2.0 1.5 1.0 0.5 0.0

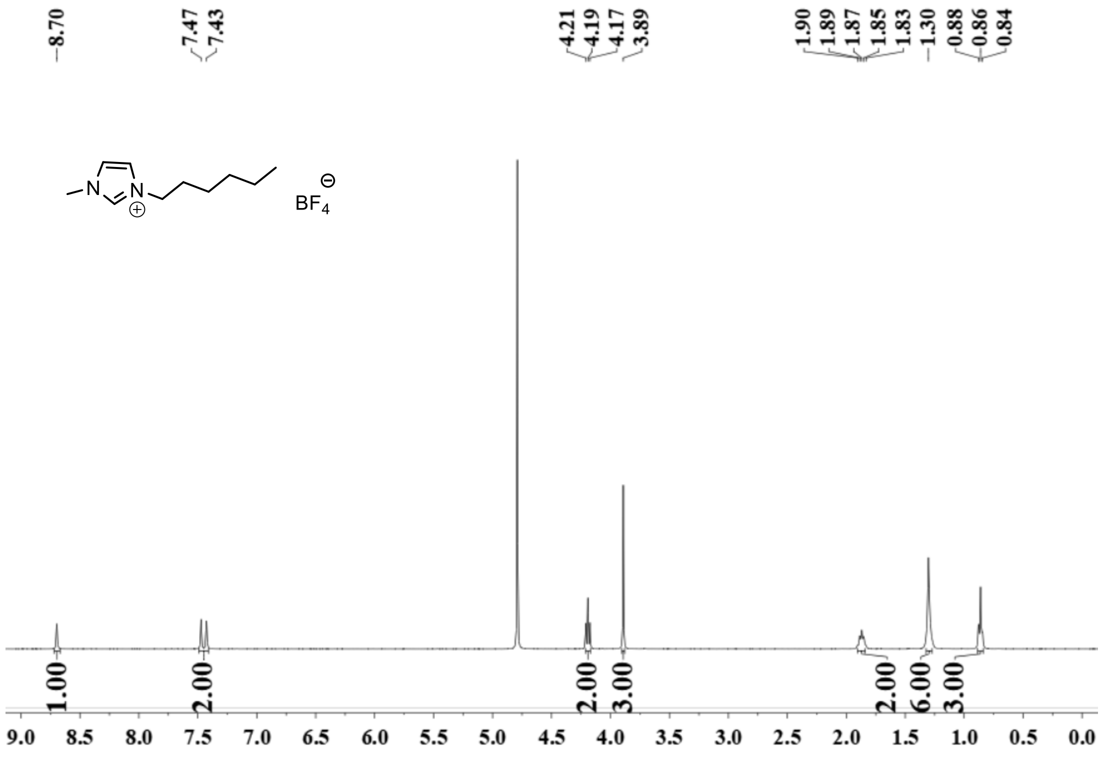
8.70
7.47
7.43
4.21
4.19
4.17
3.89
1.90
1.89
1.87
1.85
1.83
1.30
0.88
0.86
0.84
N
N
BF4
1.00
2.00
2.00
3.00
2.00
6.00
3.00
9.0 8.5 8.0 7.5 7.0 6.5 6.0 5.5 5.0 4.5 4.0 3.5 3.0 2.5 2.0 1.5 1.0 0.5 0.0

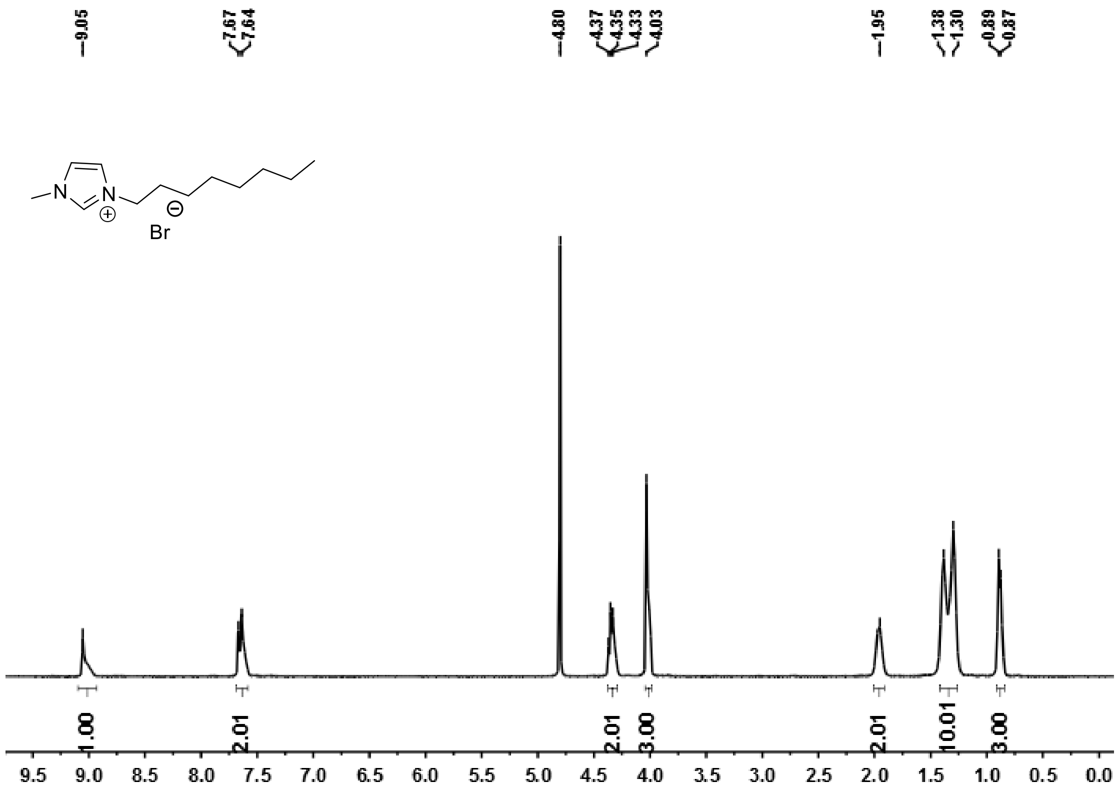
9.05
7.67
7.64
4.80
4.37
4.35
4.33
4.03
1.95
1.38
1.30
0.89
0.87
N
N
Br
1.00
2.01
2.01
3.00
2.01
10.01
3.00
9.5 9.0 8.5 8.0 7.5 7.0 6.5 6.0 5.5 5.0 4.5 4.0 3.5 3.0 2.5 2.0 1.5 1.0 0.5 0.0

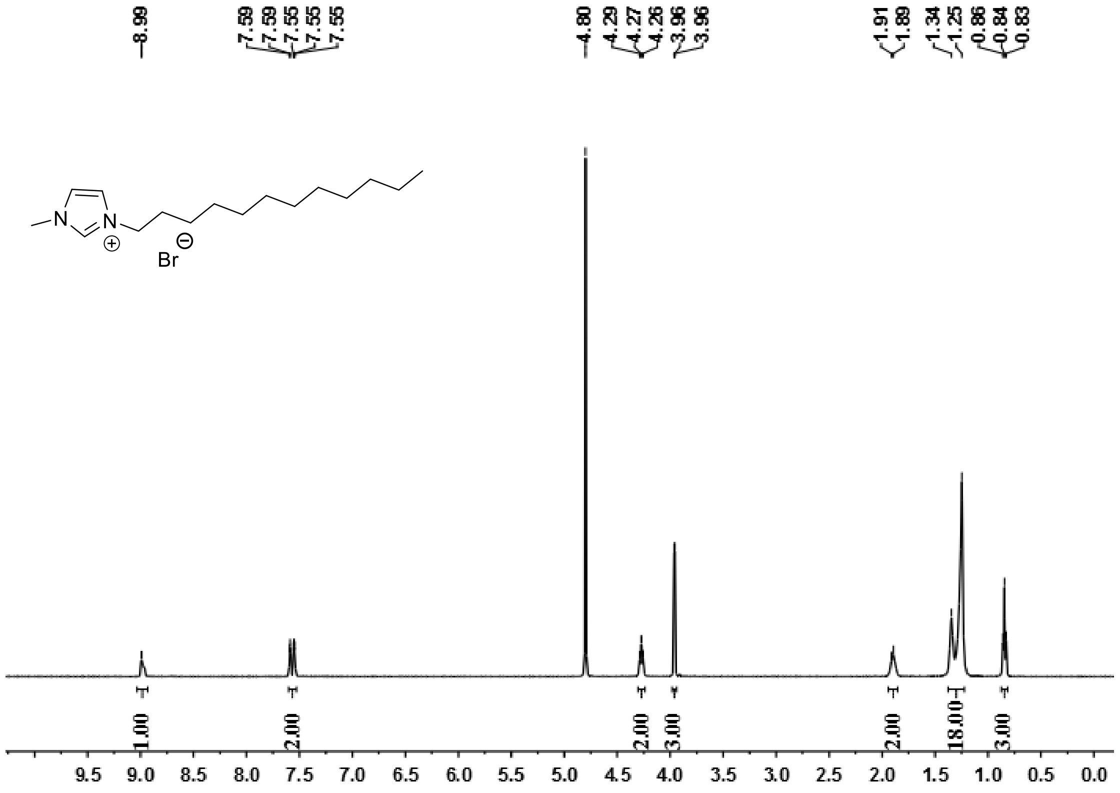
8.99
7.59
7.58
7.55
7.55
7.55
4.80
4.29
4.27
4.26
3.96
3.96
1.91
1.89
1.34
1.25
0.86
0.84
0.83
N
N
Br
1.00
2.00
2.00
3.00
2.00
18.00
3.00
9.5 9.0 8.5 8.0 7.5 7.0 6.5 6.0 5.5 5.0 4.5 4.0 3.5 3.0 2.5 2.0 1.5 1.0 0.5 0.0

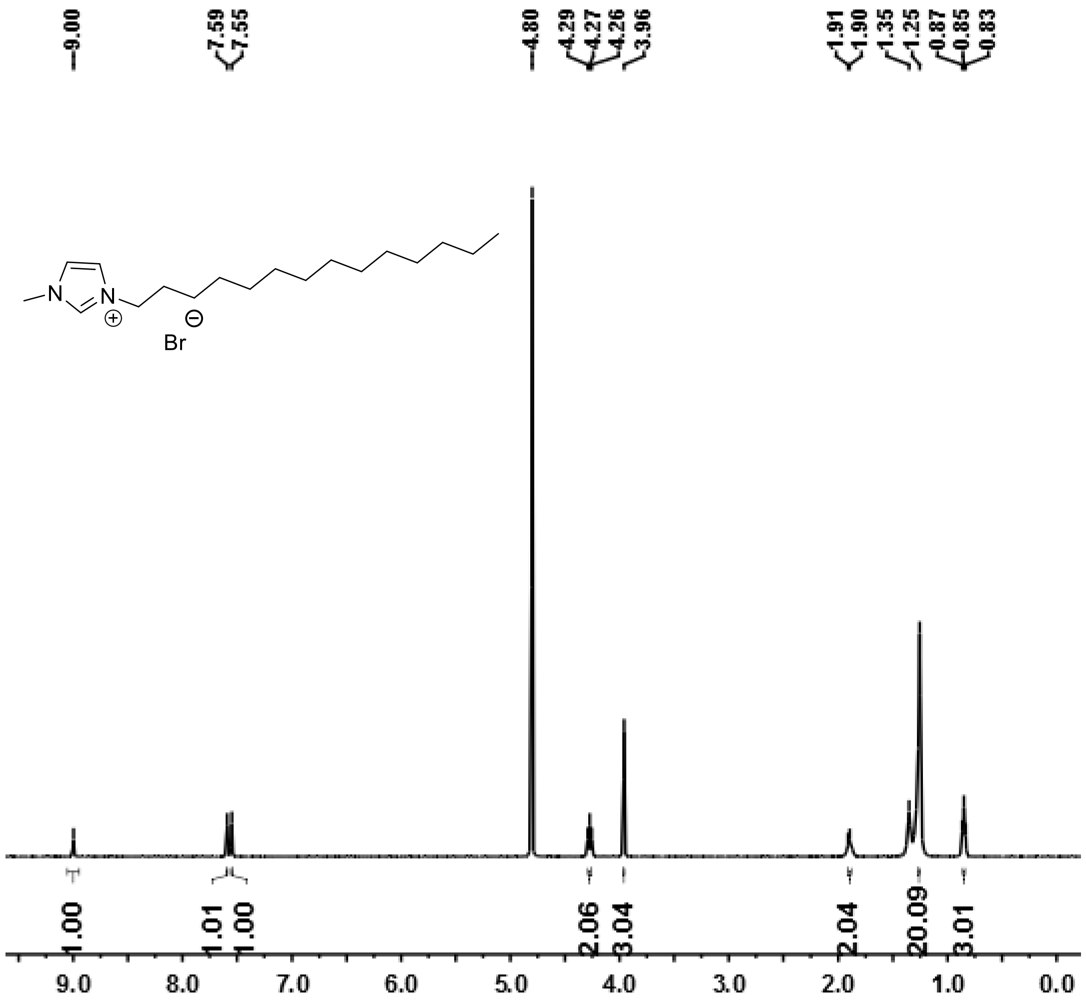

Supplementary References:

1. Zhu, A.; Li, L.; Zhang, C.; Shen, Y.; Tang, M.; Bai, L.; Du, C.; Zhang, S.; Wang. J. An integrated high-throughput strategy enables the discovery of multifunctional ionic liquids for sustainable chemical processes. *Green Chem.*, **2019**, 21, 307-313.

2. Zhu, A.; Wang, J.; Liu R. A volumetric and viscosity study for the binary mixtures of 1-hexyl-3-methylimidazolium tetrafluoroborate with some molecular solvents. *J. Chem. Thermodyn.*, **2011**, 43, 796-799.

3. Zhu, A.; Wang, J.; Han, L.; Fan, M. Measurements and correlation of viscosities and conductivities for the mixtures of imidazolium ionic liquids with molecular solutes. *Chem. Eng. J.*, **2009**, 147, 27-35

4. Stark, A.; Behrend, P.; Braun, O.; Müller, A.; Ranke, J.; Ondruschka, B.; Jastorff, B. Purity specification methods for ionic liquids. *Green Chem.*, **2008**, *10*, 1152-1161

5. Wang, J.; Zhu, A.; Zhao, Y.; Zhuo, K. Excess Molar Volumes and Excess Logarithm Viscosities for Binary Mixtures of the Ionic Liquid 1-Butyl-3-methylimidazolium Hexaflurophosphate

with Some Organic Compounds. *J. Solution Chem.*, **2005**, 34, 585-596
6. Huddleston, J. G.; Visser, A. E.; Reichert, W. M.; Willauer, H. D.; Broker, G. A.; Rogers, R. D. Characterization and comparison of hydrophilic and hydrophobic room temperature ionic liquids incorporating the imidazolium cation. *Green Chem.*, **2001**, *3,* 156-164.
7. Varma, R. S.; Namboodiri, V. V. An expeditious solvent-free route to ionic liquids using microwaves. *Chem. Commun.*, **2001**, 643-644.
8. María, G. S.; José, J. A.; L. B. MartínÁvalosbJosé LuisJiménezb IgnacioLópez, Silvero, G.; Arevalo, M. J.; Bravo, J. L.; Avalos, M.; Jimenez, J. L.; Lopez, I. An in-depth look at the effect of Lewis acid catalysts on Diels–Alder cycloadditions in ionic liquids. *Tetrahedron*, **2005**, 61, 7105-7111.
9. Baltazar, Q. Q.; Chandawalla, J.; Sawyer, K.; Anderson, J. L.; Interfacial and micellar properties of imidazolium-based monocationic and dicationic ionic liquids. *J. Colloids Surf., A*, **2007**, 302, 150-156.
10. Guzman-Lucero, D.; Flores, P.; Rojo, T.; Martı´nez-Palou, R. Ionic Liquids as Demulsifiers of Water-in-Crude Oil Emulsions: Study of the Microwave Effect. *Energ. Fuel*. **2010**, 24, 3610-3615
11. Mindt, T. L.; Schweinsberg, C.; Hagenbach, L. B. A.; Abram, U.; Tourwé, D.; Garcia-Garayoa, E.; Schibli, R. A click approach to structurally diverse conjugates containing a central di-1,2,3-triazole metal chelate. *ChemMedChem*, **2009**, 4, 529-539.
12. Chan, T. R.; Hilgraf, R.; Sharpless, K. B.; Fokin, V. V. Polytriazoles as copper(I)-stablizing ligands in catalysis. *Org. Lett.* **2004**, 6, 2853-2855.
13. Hong, V.; Presolski, S. I., Ma Celia, Finn, M. G. Analysis and optimization of copper-catalyzed azide-alkyne cycloaddition for bioconjugation. *Angew. Chem. Int. Ed.*, **2009**, 48, 9879-9883.
14. Kuhn, I.; Kellenberger, E.; Cakir-Kiefer, C.; Muller-Steffner, H.; Schuber, F. Probing the catalytic mechanism of bovine CD38/NAD^+glycohydrolase by site directed mutagenesis of key active site residues. *Biochimica & Biophysica Acta.*, **2014**, 1844, 1317-1331.
