# Supplementary material for: Biomimetic α-selective ribosylation enables two-step modular synthesis of biologically important ADP-ribosylated peptides
Source: Nat Commun. 2020 Nov 5;11:5600. doi: 10.1038/s41467-020-19409-1 (PMC7645758; doi:10.1038/s41467-020-19409-1)
Supplement: Supplementary file 3 — Reporting Summary [file 41467_2020_19409_MOESM3_ESM.pdf]

nature research

Corresponding author(s): Lingjun Li, Xiang David Li

Last updated by author(s): Sep 22, 2020

Reporting Summary

Nature Research wishes to improve the reproducibility of the work that we publish. This form provides structure for consistency and transparency in reporting. For further information on Nature Research policies, see our Editorial Policies and the Editorial Policy Checklist.

Statistics

For all statistical analyses, confirm that the following items are present in the figure legend, table legend, main text, or Methods section.

n/a	Confirmed	
[ ]	[x]	The exact sample size (n) for each experimental group/condition, given as a discrete number and unit of measurement
[ ]	[x]	A statement on whether measurements were taken from distinct samples or whether the same sample was measured repeatedly
[x]	[ ]	The statistical test(s) used AND whether they are one- or two-sided<br>*Only common tests should be described solely by name; describe more complex techniques in the Methods section.*
[x]	[ ]	A description of all covariates tested
[x]	[ ]	A description of any assumptions or corrections, such as tests of normality and adjustment for multiple comparisons
[ ]	[x]	A full description of the statistical parameters including central tendency (e.g. means) or other basic estimates (e.g. regression coefficient) AND variation (e.g. standard deviation) or associated estimates of uncertainty (e.g. confidence intervals)
[x]	[ ]	For null hypothesis testing, the test statistic (e.g. F, t, r) with confidence intervals, effect sizes, degrees of freedom and P value noted<br>*Give P values as exact values whenever suitable.*
[x]	[ ]	For Bayesian analysis, information on the choice of priors and Markov chain Monte Carlo settings
[x]	[ ]	For hierarchical and complex designs, identification of the appropriate level for tests and full reporting of outcomes
[x]	[ ]	Estimates of effect sizes (e.g. Cohen's d, Pearson's r), indicating how they were calculated

Our web collection on statistics for biologists contains articles on many of the points above.

Software and code

Policy information about availability of computer code

Data collection	Thermal analysis of ionic liquids was performed on a Netzsch STA 449 F5 with NETZSCH Proteus-Thermal Analysis-Version 6.1.0 software. The HPLC was performed on a Thermo SIENTIFIC Ultimate 3000 with Thermo ScientificTM DionexTM ChromeleonTM 7.2 SR5 software.
Data analysis	All images were processed by Windows ImageJ bundled with 64-bit Java 1.8.0_172 software (National Institutes of Health), and contrast was adjusted appropriately. IC50 and dissociation constants Kd were fit with Origin 7.0 software package (OriginLab). The data analysis in Fig 2d, supplementary Figure 2 to 7 were conducted by Prism 8 (GraphPad) software. The data analysis of DSC/TGA were conducted by NETZSCH Proteus-Thermal Analysis-Version 6.1.0 software.

For manuscripts utilizing custom algorithms or software that are central to the research but not yet described in published literature, software must be made available to editors and reviewers. We strongly encourage code deposition in a community repository (e.g. GitHub). See the Nature Research guidelines for submitting code & software for further information.

Data

Policy information about availability of data

All manuscripts must include a data availability statement. This statement should provide the following information, where applicable:

- Accession codes, unique identifiers, or web links for publicly available datasets
- A list of figures that have associated raw data
- A description of any restrictions on data availability

The data supporting the findings of this work are available within the article and its Supplementary Information files. Raw data underlying Fig 2d, supplementary figures 2-7 and uncropped versions of gels and blots presented in the fig.4 are available in figshare (doi:10.6084/m9.figshare.12375032). All data are available from the authors upon reasonable request. Source data are provided with this paper.

Field-specific reporting

Please select the one below that is the best fit for your research. If you are not sure, read the appropriate sections before making your selection.

[x] Life sciences   [ ] Behavioural & social sciences   [ ] Ecological, evolutionary & environmental sciences

For a reference copy of the document with all sections, see nature.com/documents/nr-reporting-summary-flat.pdf

Life sciences study design

All studies must disclose on these points even when the disclosure is negative.

Sample size	No statistical method was used to pre-determine sample sizes. Samples sizes for each experiment listed in Figure 4, Supplementary Figures 10 and 11 were based on our and others' previous publications (X. Li, T. M. Kapoor, J. Am. Chem. Soc. 2010, 132, 2504-2505; P. M. Moyle, T. W. Muir, J. Am. Chem. Soc. 2010, 132, 15878-15880)
Data exclusions	No data were excluded.
Replication	Results of Figures 4a, 4c, 4e, 4f, 4g, Supplementary Figures 10a, 10b were independently repeated for 3 times. Results of Figures 4b and 4d were independently repeated for 2 times. All replicates were successful.
Randomization	No randomization were applied in this study since the samples are prepared with defined conditions including protein and chemical identities, chemical concentrations, competitor concentrations, temperatures, durations. All samples were analyzed equally with no sub-sampling.
Blinding	Blinding is not relevant to this study since no group allocation was required.

Reporting for specific materials, systems and methods

We require information from authors about some types of materials, experimental systems and methods used in many studies. Here, indicate whether each material, system or method listed is relevant to your study. If you are not sure if a list item applies to your research, read the appropriate section before selecting a response.

Materials & experimental systems

n/a	Involved in the study	
[ ]	[x]	Antibodies
[ ]	[x]	Eukaryotic cell lines
[x]	[ ]	Palaeontology and archaeology
[x]	[ ]	Animals and other organisms
[x]	[ ]	Human research participants
[x]	[ ]	Clinical data
[x]	[ ]	Dual use research of concern

Methods

n/a	Involved in the study	
[x]	[ ]	ChIP-seq
[x]	[ ]	Flow cytometry
[x]	[ ]	MRI-based neuroimaging

Antibodies

Antibodies used	Antibodies used in this study were macroH2A1.1 antibody (D5F6N, Cell Signaling Technology, #12455), PARP9 antibody (ab53796, Abcam), anti-poly(ADP-ribose) polymer antibody (ab14459, Abcam), goat anti-rabbit-HRP conjugated secondary antibody (sc-2004, Santa Cruz), donkey anti-mouse-HRP conjugated secondary antibody (sc-2314, Santa Cruz)
Validation	The applications of the antibodies used in this study have been validated by the manufactures based on their on-line statements. Specifically:<br>For macroH2A1.1 antibody, see https://www.cellsignal.com/products/primary-antibodies/macroh2a1-1-d5f6n-rabbit-mab/12455?Ntk=Products&Ntt=12455<br>For PARP9 antibody, see https://www.abcam.com/parp9-antibody-ab53796.html<br>For anti-poly(ADP-ribose) polymer antibody, see https://www.abcam.com/poly-adp-ribose-polymer-antibody-10h-ab14459.html<br>For goat anti-rabbit-HRP conjugated secondary antibody, see https://www.scbt.com/p/goat-anti-rabbit-igg-hrp?productCanUrl=goat-anti-rabbit-igg-hrp&_requestid=1455301<br>For donkey anti-mouse-HRP conjugated secondary antibody, see https://www.scbt.com/p/donkey-anti-mouse-igg-hrp.

Eukaryotic cell lines

Policy information about cell lines

Cell line source(s)	HeLa S3 cells were purchased from American Type Culture Collection (ATCC)

Authentication	All the cell lines were tested for authentication by short tandem repeat (STR) profiling.
Mycoplasma contamination	All the cell lines were routinely tested for mycoplasma, showing mycoplasma-negative results.
Commonly misidentified lines (See ICLAC register)	No commonly misidentified cell lines were used.
