# Supplementary figures and images for: Biomimetic α-selective ribosylation enables two-step modular synthesis of biologically important ADP-ribosylated peptides
Source: Nat Commun. 2020 Nov 5;11:5600. doi: 10.1038/s41467-020-19409-1 (PMC7645758; doi:10.1038/s41467-020-19409-1)

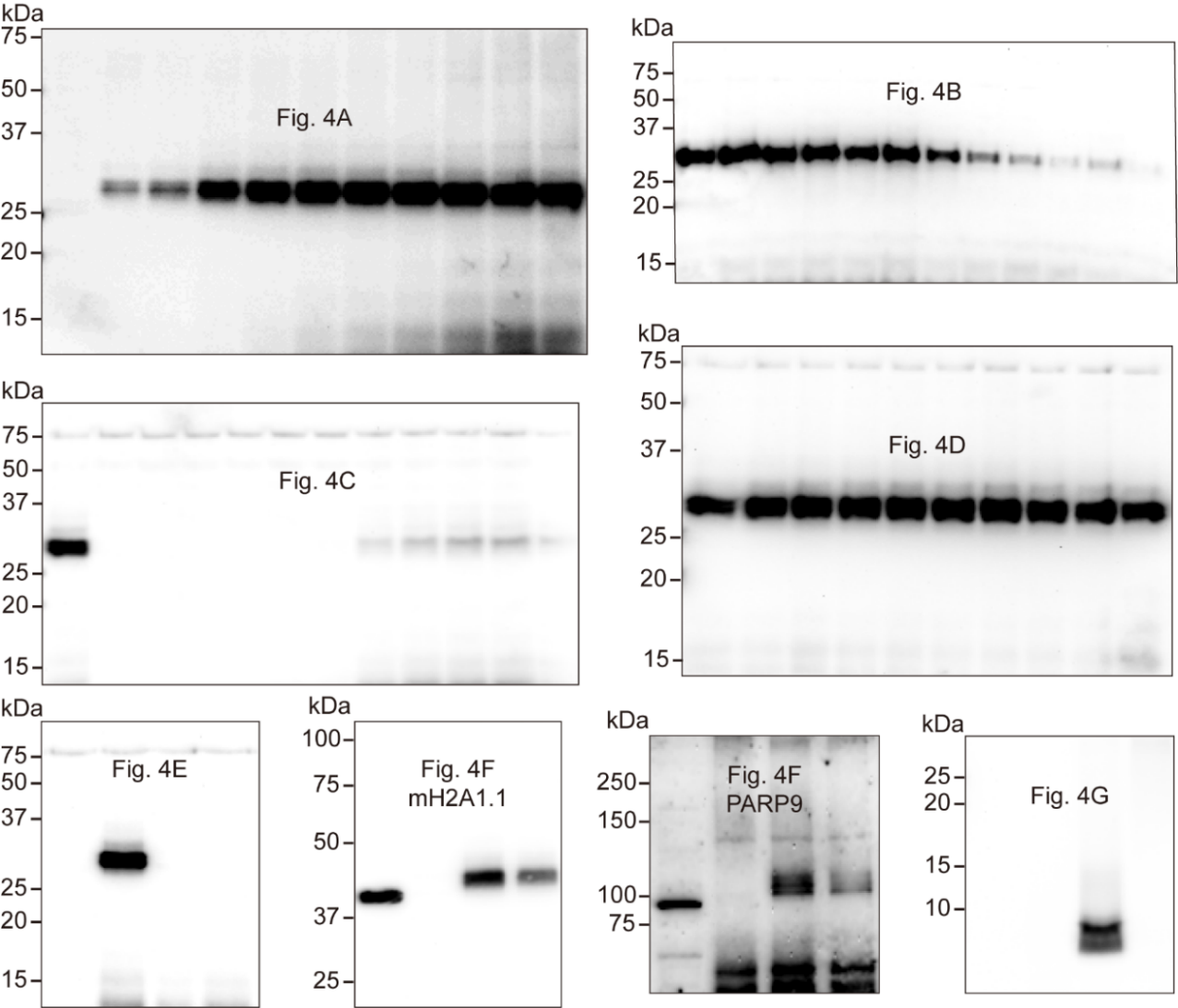


Uncropped blots of Fig. 4.

Supplement: Supplementary file 4 — Source Data [file 41467_2020_19409_MOESM4_ESM.zip › Source Data-9-26/Uncropped blots of Fig. 4.docx]
